# Human mesenchymal stromal cells broadly modulate high glucose-induced inflammatory responses of renal proximal tubular cell monolayers

**DOI:** 10.1186/s13287-019-1424-5

**Published:** 2019-11-19

**Authors:** Md Nahidul Islam, Tomás P. Griffin, Elizabeth Sander, Stephanie Rocks, Junaid Qazi, Joana Cabral, Jasmin McCaul, Tara McMorrow, Matthew D. Griffin

**Affiliations:** 1 Regenerative Medicine Institute (REMEDI) at CÚRAM Centre for Research in Medical Devices, School of Medicine, National University of Ireland Galway, Galway, REMEDI, Biomedical Sciences, Corrib Village, Dangan, Galway, H91 TK33 Ireland; 2 Centre for Endocrinology, Diabetes and Metabolism, Galway University Hospitals, Galway, Ireland; 3 School of Biomolecular and Biomedical Science, Conway Institute, University College Dublin, Dublin, Ireland

## Abstract

**Background:**

Renal proximal tubular epithelial cells (RPTEC) are dysfunctional in diabetic kidney disease (DKD). Mesenchymal stromal cells (MSC) may modulate DKD pathogenesis through anti-inflammatory mediators. This study aimed to investigate the pro-inflammatory effect of extended exposure to high glucose (HG) concentration on stable RPTEC monolayers and the influence of MSC on this response.

**Methods:**

Morphologically stable human RPTEC/TERT1 cell monolayers were exposed to 5 mM and 30 mM (HG) *D-glucose* or to 5 mM *D-glucose* + 25 mM *D-mannitol* (MAN) for 5 days with sequential immunoassays of supernatants and end-point transcriptomic analysis by RNA sequencing. Under the same conditions, MSC-conditioned media (MSC-CM) or MSC-containing transwells were added for days 4–5. Effects of CM from HG- and MAN-exposed RPTEC/MSC co-cultures on cytokine secretion by monocyte-derived macrophages were determined.

**Results:**

After 72–80 h, HG resulted in increased RPTEC/TERT1 release of interleukin (IL)-6, IL-8, monocyte chemoattractant protein (MCP)-1 and neutrophil gelatinase-associated lipocalin (NGAL). The HG pro-inflammatory effect was attenuated by concentrated (10×) MSC-CM and, to a greater extent, by MSC transwell co-culture. Bioinformatics analysis of RNA sequencing data confirmed a predominant effect of HG on inflammation-related mediators and biological processes/KEGG pathways in RPTEC/TERT1 stable monolayers as well as the non-contact-dependent anti-inflammatory effect of MSC. Finally, CM from HG-exposed RPTEC/MSC transwell co-cultures was associated with attenuated secretion of inflammatory mediators by macrophages compared to CM from HG-stimulated RPTEC alone.

**Conclusions:**

Stable RPTEC monolayers demonstrate delayed pro-inflammatory response to HG that is attenuated by close proximity to human MSC. In DKD, this MSC effect has potential to modulate hyperglycemia-associated RPTEC/macrophage cross-talk.

## Background

Diabetic kidney disease (DKD) is the leading cause of end stage renal disease (ESRD) worldwide [1]. The complex pro-inflammatory milieu of hyperglycaemia, reactive oxygen species (ROS), advanced glycation end products (AGE) and angiotensin-II contributes to activation of transcription factors, growth factors, inflammatory cytokines and chemokines that mediate glomerular, microvascular and tubulo-interstitial injury—eventually leading to progression to ESRD and to the increased cardiovascular mortality associated with DKD [2–4].

A substantial body of research evidence documents the links between chronic inflammation and the development and progression of DKD [5–8]. Hyperglycaemia induces cytokine production by macrophages and other immune cells which may serve both as drivers and predictive biomarkers for progressive loss of renal function [7, 9, 10]. For example, circulating concentration of monocyte chemoattractant protein-1 (MCP-1/CCL2) has been shown to correlate with the degree of interstitial macrophage infiltration in human DKD while, experimentally, inhibition of MCP-1 in models of diabetes mellitus (DM) ameliorates renal injury [11–13]. Hyperglycaemia upregulates MCP-1 production by kidney tubular epithelial cells, leading to infiltration of monocytes into the kidneys where they may subsequently become differentiated into inflammatory macrophages [14, 15]. This is further associated with localised release of pro-inflammatory cytokines such as interleukin (IL)-1β, IL-6 and tumour necrosis factor-alpha (TNFα) [15, 16].

The renal proximal tubular epithelial cell (RPTEC) is a significant target for the adverse effects of chronic hyperglycaemia. Excessive glucose in the glomerular filtrate drives increased glucose reabsorption in the proximal tubules and activates a range of maladaptive pathways within RPTEC that contribute to the DKD pathogenesis [17–22]. As DKD progresses, secondary mediators including growth factors, angiotensin-II and AGE activate inflammatory signalling pathways to further increase ROS production, inflammation, tubular cell hypertrophy and interstitial fibrosis [17–19, 21]. These insights highlight RPTEC as a potentially important therapeutic target in DKD, and in vitro studies involving cultured RPTEC-like cells provide a valuable test-bed for identifying and evaluating novel interventional strategies [21, 23]. Among the in vitro tools available, RPTEC/TERT1 is an immortalised RPTEC cell line generated by overexpression of human telomerase reverse transcriptase (hTERT) [24]. Recent studies have highlighted the potential advantages of RPTEC/TERT1 stable monolayer cultures over other cell lines for modelling renal proximal tubular function and responses [25–27].

Macrophages are key mediators of intra-renal inflammation in DM, being an important source of pro-inflammatory factors including IL-1, TNFα, IL-6 and ROS [28]. In vivo, macrophage infiltration and activation within the kidneys of diabetic animals as well as other models of renal injury has been shown to contribute significantly to increased production of chemokines, interstitial fibrosis and increased serum creatinine and proteinuria [29–32]. Combined with the direct effects of chronic hyperglycaemia to induce pro-inflammatory responses in RPTEC, these studies indicate that crosstalk between RPTEC and interstitial macrophages within the kidney represents a key pathological axis in the development and progression of DKD.

Currently, a limited number of therapies are available that specifically target the development and progression of DKD. Novel interventions that modulate multiple inflammatory pathways as well as promote repair of tubule-interstitial injury could well complement conventional drug classes that predominantly address maladaptive glomerular pathophysiology in DKD. Relevant to this, interventions to inhibit pro-inflammatory cross-talk between RPTEC and macrophages represent an attractive strategy. Mesenchymal stem/stromal cell (MSC) therapy is a potential therapeutic option for diverse inflammatory disease pathologies [2, 33, 34]. In their physiological, perivascular niches in the bone marrow and other tissues, MSC have critical roles in immunomodulation and self-renewal [2, 34, 35]. Recent studies in animal models of DKD indicate that systemic administration of MSC ameliorates DM-associated albuminuria and renal pathological abnormalities in a paracrine manner through immunomodulatory and anti-apoptotic effects [36–39]. The potential clinical translation of MSC therapy for progressive DKD has reached the stage of early-phase clinical trials but the precise mechanisms of action of MSC remain incompletely characterised.

In this study, we aimed to determine the immunological consequences of prolonged exposure of RPTEC/TERT1 stable monolayers to high concentrations of glucose and to investigate the modulatory effects of culture-expanded human MSC and their soluble products on high glucose (HG)-induced RPTEC inflammatory response and the resulting RPTEC/macrophage cross-talk.

## Methods

### RPTEC/TERT1 cell culture and treatments

RPTEC/TERT1 (human renal proximal tubular epithelial cell line from the American Tissue Culture Collection) were cultured in 24-well flat-bottom plates (Sarstedt, Numbrecht, Germany) in Dulbecco’s modified Eagle’s medium (DMEM) (Gibco, Grand Island, NY, USA) and Ham’s F-12 medium (Gibco) at 1:1 supplemented with ITS (Sigma Aldrich, St. Louis, MO, USA) containing 10 μg/ml insulin, 5.5 μg/ml transferrin and 5 ng/ml sodium selenite; 10 ng/ml epidermal growth factor (Sigma); 36 ng/ml hydrocortisone (Sigma); 2 mM l-glutamine (Gibco) and 100 U/ml penicillin and 100 μg/ml streptomycin (Gibco) and maintained at 37 °C, 5% CO_2_ in a humidified tissue culture incubator. For stabilisation of the monolayer, 27,500 cells/cm^2^ were plated, cultured for 6 days to 100% confluency then allowed to form stable monolayers for a further 6 days before use in individual experiments. The medium was replaced every 2 days. For “high glucose (HG)” and “mannitol osmotic control (MAN)” culture conditions, the medium was additionally supplemented with 25 mM *D-glucose* (Sigma) or 25 mM D-mannitol (Sigma) respectively was added at day 12 and maintained for a further 4–5 days. In some experiments, the medium was also supplemented with 100 μg/ml human serum albumin (Sigma), or 1 ng/ml IL-1β (Peprotech EC Ltd., NJ, USA) and 20 ng/ml TNFα (Peprotech) for the final 5 or 2 days of culture respectively. Phase-contrast microscopy and image capture of cultured cells were performed at intervals using an Olympus-IX71 inverted microscope (Tokyo, Japan). Osmolality of the cell culture supernatants from CTRL, HG and MAN conditions was measured in the Clinical Biochemistry Laboratory, Galway University Hospitals.

### Culture of mesenchymal stromal cells and control cells

Cryopreserved human bone marrow-derived MSC (BM-MSC) from two healthy donors were cultured in MEM-Alpha media (Gibco) supplemented with 10% extracellular vesicle (EV)-free heat-inactivated foetal calf serum (FCS) (Gibco), 1% penicillin/streptomycin (Gibco) and 1 ng/ml fibroblast growth factor (R&D Systems, Minneapolis, MN, USA). EV-free FCS was prepared by ultracentrifugation of FCS at 100,000×*g* (Sorvall 100SE Ultra Centrifuge) for 18 h and subsequent collection of supernatants. Culture of human corneal endothelial cells (HCEC) was carried out in DMEM supplemented with 10% FCS (Gibco) and 1% penicillin/streptomycin (Gibco). Conditioned media were prepared as described in Additional file 1: Supplementary Methods. In the case of the MSC-derived CM, this was further divided into non-manipulated CM (“MSC-CM (Whole)”) and MSC-CM from which the MSC-derived EV were depleted by ultracentrifugation for 18 h as 100,000×*g* (“MSC-CM (-EV)”).

### Indirect co-culture of RPTEC/TERT1 cells and mesenchymal stromal cells

RPTEC/TERT1 cells were plated at 27,500 cells/cm^2^ in six-well tissue culture plates and were cultured for 12 days to form stable monolayers. The cells were then cultured in medium additionally supplemented with 25 mM D-glucose (Sigma) or D-mannitol (Sigma) for a further 5 days as described above. Human BM-MSC were separately seeded at 10,000 cells/cm^2^ into transwell inserts (ThinCert™, Greiner Bio-One, Kremsmünster, Austria) for 3 days in MSC culture medium. On day 15 of RPTEC/TERT1 cell culture, the BM-MSC-containing inserts were placed on top of individual RPTEC/TERT1 monolayer-containing wells and these co-cultures were maintained for a further 2 days, following which the RPTEC/TERT1 cell pellets and supernatants were collected for protein analysis and enzyme-linked immunosorbent assays (ELISA) respectively. Transwell co-cultures of RPTEC/TERT1 cells and HCEC were carried out by the same protocol.

### Enzyme-linked immunosorbent assays

Assay kits for Interleukin-1β (IL-1β), IL-6, IL-8, TNFα, IL-10, MCP-1 and neutrophil gelatinase-associated lipocalin (NGAL) (R&D Systems, MN, USA) were used to perform ELISAs of culture supernatants according to the manufacturer’s instructions (see Additional file 1: Supplementary Methods for a detailed protocol).

### Flow cytometry

Viability of RPTEC/TERT1 cells was determined by propidium iodide (PI) (Molecular Probes, Oregon, USA) staining. In brief, following trypsinization and centrifugation, cell pellets were re-suspended in culture medium and incubated for 15 min at 37 °C to restore membrane integrity. The cell suspensions were then washed and re-suspended in FACS buffer containing 2% FCS (Gibco) and 0.05% NaN_2_ (Sigma) in PBS (Sigma). Cells were transferred as 100-μl aliquots into 5-ml polystyrene FACS tubes (Sarstedt). Finally, PI solution was added to final concentration of 1 μg/ml and the samples were analysed on an Accuri-C6 flow cytometer (Becton Dickinson, USA) using CFlow software.

### Western blotting

Immunoblots of RPTEC-derived protein lysates for NF-κB p65, phospho-NF-kB p65 (pP65), p38 MAPK, phospho-p38 MAPK (pP38MAPK), p44/42 MAPK (Erk1/2), phospho-p44/42 MAPK (pErk1/2; Thr202/Tyr204), STAT1, phospho-Stat1 (pSTAT1; Tyr701), protein kinase-C alpha (PKCα), phospho-PKCα/β II (pPKCα; Thr638/641) and PPAR-γ were performed using reagents and procedures described in Additional file 1**:** Supplementary Methods.

### RNA isolation and quantification

Total RNA was isolated from RPTEC/TERT1 cells using TRIzol/Chloroform method and by RNEasy Midi Kit (Qiagen, Hilden, Germany). A detailed protocol for RNA isolation by TRIzol method is provided in Additional file 1: Supplementary Methods. For samples prepared using RNEasy Midi Kit (Qiagen), the manufacturer’s recommended protocol was followed. The quality and integrity of all RNA samples were measured by Bioanalyzer-2100 using RNA 6000 Pico kit (Agilent Technologies) according to the manufacturer’s recommended protocol.

### RNA sequencing and bioinformatics analysis

High-throughput RNA sequencing (RNA-seq) was performed by BGI Genomics Service (Hong Kong) using BGISEQ-500, and Bioinformatics analyses of the resulting transcriptional profiles were performed using a suite of software packages including RSEM (quantitation of gene expression level), Cluster and Java Treeview Cluster (clustering analysis of gene expressions), Medusa (protein-protein interactions), WCGNA and Cytoscape (gene co-expression network analysis). Only RNA samples with RNA integrity number (RIN) ≥ 7.0 were subjected to RNA-seq. In brief, following fragmentation of mRNA and subsequent reverse transcription and amplification, a sequencing library was prepared. Nucleotide sequence of the fragments was determined and high-quality reads were aligned to the reference genomic sequence. Fragments that matched the genomic sequence were assigned to a specific position of a specific chromosome in the genome; thereby, the gene fragments could be linked to a specific gene. The number of reads per gene were counted and normalised. The criteria for designation of differentially expressed genes (DEG) were > 1.5 absolute fold-change and statistical significance (*p* < 0.05) among experimental conditions. RNA-seq data analyses involved plotting data using principal component analysis and KEGG pathway enrichment analysis.

### Quantitative, reverse transcription polymerase chain reaction (qRT-PCR)

For qRT-PCR, the Luna Universal Probe One Step RT-qPCR kit (New England BioLabs, MA, USA) was used according to the manufacturer’s instructions. Reactions consisted of 50 ng RNA samples, mastermix, nuclease-free water, enzyme and primer/probe in a final volume of 10 μl. Individual target specific primers (both forward and reverse; for IL-6, MCP-1, IL-1β, IL-8, TNFα, NGAL and RPLP0) and TaqMan probes (for quantitation) were purchased from Integrated DNA Technologies (Coralville, Iowa, USA). The primer sequences are listed in Additional file 2: Table S1. The reactions were performed on Step-One Plus PCR instrument (Applied Biosystems, Waltham, MA, USA). Mean Ct values were used to calculate the fold changes in the expression of different target genes (for IL-6, MCP-1, IL-1β, IL-8, TNFα and NGAL) in treatment groups vs. control as determined relative to the housekeeping gene RPLP0 using the 2^–∆∆Ct^ method.

### Culture of primary human macrophages and exposure to conditioned media

Human peripheral blood mononuclear cells (PBMCs) were prepared and cultured overnight using a standard protocol (described in detail in Additional file 1: Supplementary Methods). Plastic-adherent PBMCs were cultured in 24-well plates (Sarstedt) at a density of 22,500 cells/cm^2^ in macrophage medium containing 20 ng/ml granulocyte macrophage colony stimulating factor (GM-CSF, Peprotech). Medium was replaced every 3 days. After 9 days, the medium was replaced with conditioned medium (CM) from the RPTEC/TERT1 co-culture experiments with addition of GM-CSF to a final concentration of 20 ng/ml. To some wells, 100 ng/ml interferon gamma (IFNγ, Peprotech), 100 ng/ml TNFα (Peprotech) and 50 ng/ml LPS (Sigma) were added to provide a positive control for pro-inflammatory stimulation. After 24 h, the CM was removed, the macrophages were washed with PBS and fresh macrophage medium containing GM-CSF was added. Finally, after an additional 24 h of culture, the supernatants were collected for subsequent analysis by ELISA.

*Statistical analysis* was performed using GraphPad Prism version 6.0. Paired- or unpaired Student’s *t* test, non-parametric multiple *t* test and one- or two-way ANOVA were used for analysis of individual experiments as appropriate. Details of statistical analyses performed for specific experiments are provided in individual figure legends. For all statistical analyses, the threshold for significance was < 0.05. Experiments were performed at least three times unless otherwise stated in the figure legends.

## Results

### Prolonged exposure to high glucose enhances inflammatory response of RPTEC/TERT1 monolayers

**Fig. 1:**
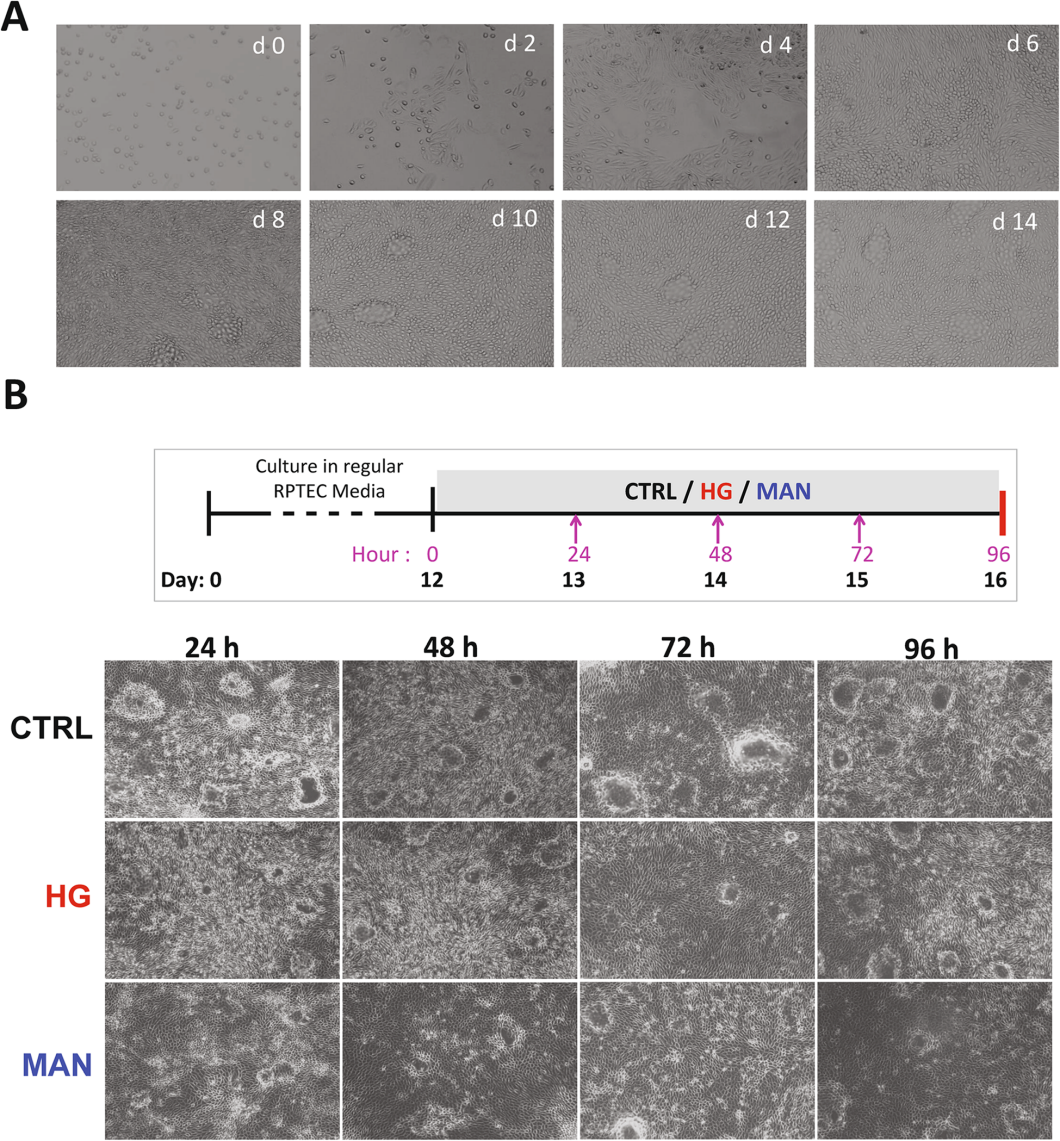
Morphology of RPTEC/TERT1 cell layers in culture. **a** Morphology at different time points before and after confluency using phase contrast microscopy at × 4 magnification. **b** Morphology of cell layers during basal medium (CTRL), HG and MAN culture conditions beginning at day 12 for four consecutive days using phase-contrast microscopy at × 4 magnification

Based on previously published characterizations of the RPTEC/TERT1 cell line by us and others [25–27], culture conditions were established under which RPTEC-TERT1 cells, seeded at an optimised initial density (Additional file 3: Figure S1A), reached confluence by day 6 and formed stable monolayers by day 12. As shown in Fig. 1a, seeded cells progressed from sub-confluent, spindle-shaped cells to confluent layers of tightly packed cells of cobblestone appearance between days 0 and 6 of culture then developed frequent dome-shaped protuberances between days 8 and 12. After 12 days of culture, RPTEC/TERT1 monolayers were cultured for an additional 4 days under RM, HG or MAN conditions. As shown in Fig. 1b, RPTEC/TERT1 monolayers continued to show a confluent, cobblestone appearance during this time period and no morphological differences were observed among the culture conditions. The osmolalities of HG and MAN culture media were confirmed to be higher than that of the basal medium (HG = 360 ± 10 mosm/ml; MAN = 366 ± 9 mosm/ml; CTRL = 339 ± 3 mosm/ml).

**Fig. 2:**
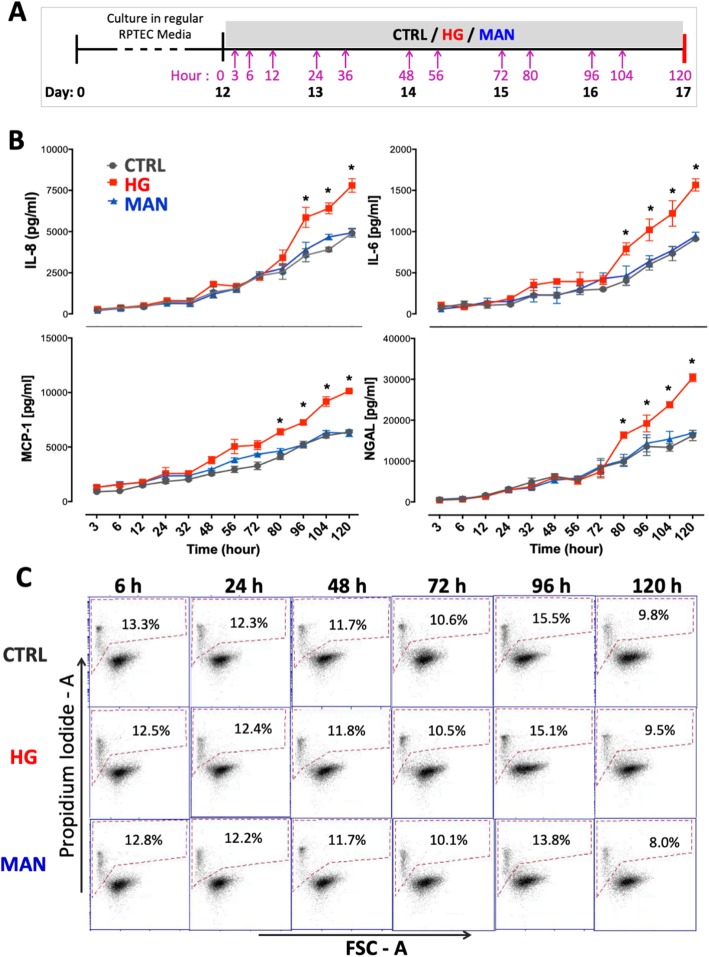
High glucose exposure increases the inflammatory response of RPTEC/TERT1 cell stable monolayers: **a** Schematic diagram of the experimental protocol. **b** Concentrations of inflammatory biomarkers IL-8 (top left), IL-6 (top right), MCP-1 (bottom left) and tubular injury marker NGAL (bottom right) in culture supernatants of RPTEC/TERT1 cell stable monolayers between 3 and 120 h following exposure to medium containing normal (5 mM) glucose (CTRL, grey line), high (30 nM) glucose (HG, red line) and 5 mM glucose + 25 mM mannitol (MAN, blue line). All results are presented as mean ± SD. Statistical analyses were performed by one-way ANOVA. *p < 0.05. **c** Representative examples of flow cytometric analysis of RPTEC/TERT1 cell viability by propidium iodide exclusion at various time-points following exposure to the three culture conditions. Percentages of dead cells (PI^+ve^) are shown for each condition

The influence of high glucose (HG) exposure for a further 5 days (120 h) on secretion of inflammation-related soluble factors (IL-6, IL-8 and MCP-1) and tubular injury-related marker NGAL into the culture medium was then compared to that of normal-glucose (CTRL) and mannitol osmotic control (MAN) culture conditions (Fig. 2a, b). As shown, increased secretion of all four analytes was observed under HG but not MAN conditions relative to CTRL between 80 and 120 h of culture. The HG-induced increases in inflammatory factors were not associated with increased cell death (Fig. 2c). As further evidence of the relevance of the culture system to diabetic conditions, it was verified that the exposure of RPTEC/TERT1 monolayers to other relevant stimuli—human serum albumin (HSA) and IL-1β—known to exert pro-inflammatory effects on proximal tubular epithelial cells, resulted in increased secretion of IL-6, IL-8, MCP-1 and NGAL that was additive to the induction associated with HG culture (see Additional file 4: Figure S2 and Additional file 5: Figure S3). Neither HSA nor IL-1β were associated with increased cell death during culture (data not shown). Western blotting of RPTEC/TERT1 monolayer-derived protein lysates collected at 24, 48 and 96 h following exposure to CTRL, HG and MAN conditions showed evidence of activation of multiple potentially pro-inflammatory intracellular signalling pathways (NF-kB, p38 and ERK1/2 MAPK and PKCα). However, the level of activation, based on abundance of phosphorylated pathway components, was no greater for HG compared to the control conditions (see Additional file 6: Figure S4A and Additional file 7: Figure S4B). It was concluded that prolonged exposure of mature RPTEC/TERT1 cell monolayers to HG results in increased release of pro-inflammatory mediators that is not driven by sustained hyperactivity of the individual signalling pathways examined and is not associated with overt loss of viability, gross morphological changes or dedifferentiation to a non-epithelial phenotype.

### Conditioned medium from human mesenchymal stromal cells inhibits the high glucose-induced inflammatory response of RPTEC/TERT1 monolayers

**Fig. 3:**
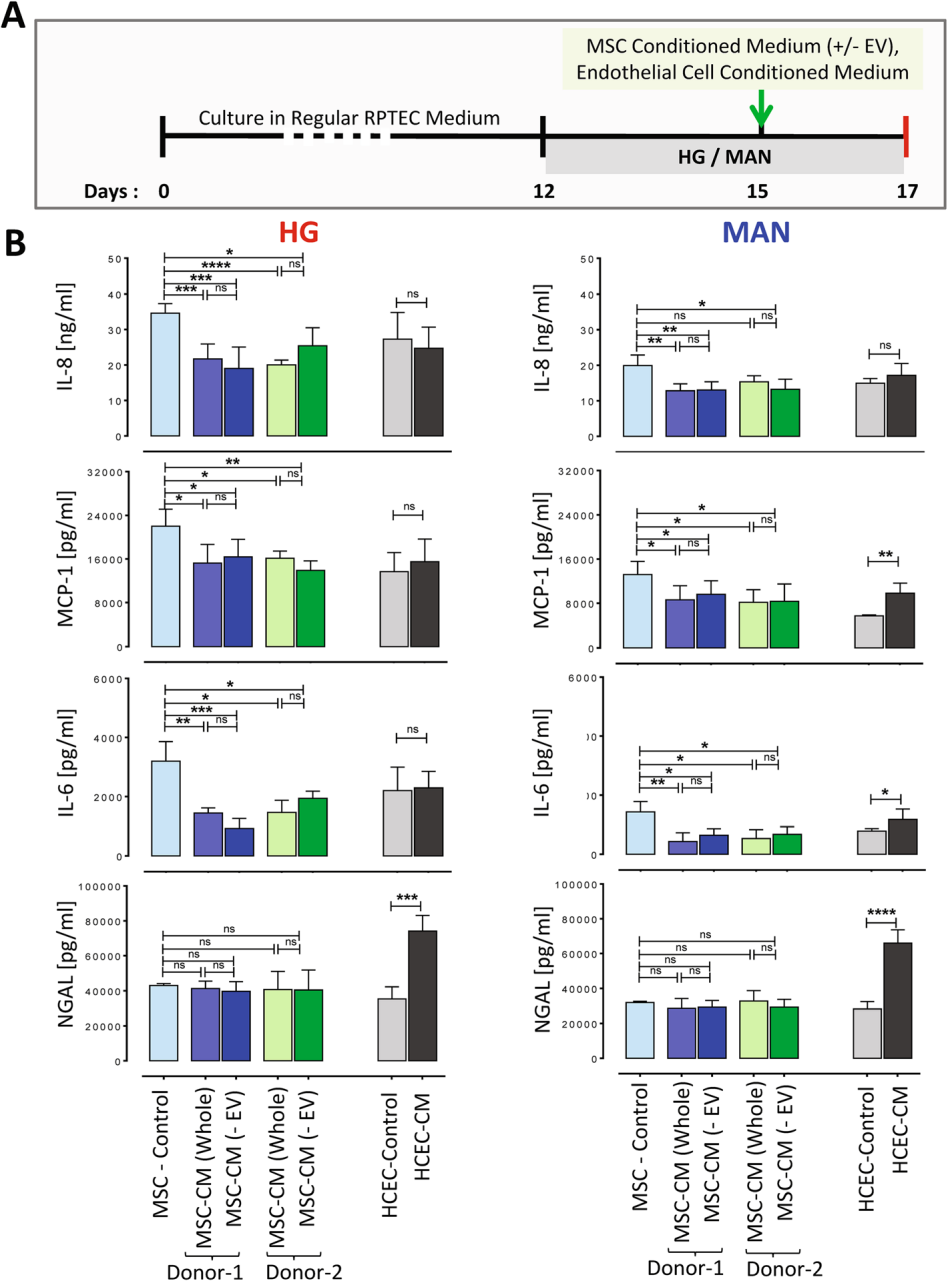
Total and extracellular vesicle-depleted conditioned media from human MSC but not HCEC inhibit high glucose-induced secretion of inflammatory cytokines by RPTEC/TERT1 cell stable monolayers*.* **a** Schematic diagram of the experimental protocol (+/− EV = without and with extracellular vesicle depletion). **b** Graphs depicting the concentrations of IL-8, IL-6, MCP-1 and NGAL in the supernatants from RPTEC/TERT1 cell monolayers cultured without (control) and with various conditioned media (CM) during the final 2 days of 5-day cultures under high glucose (HG, left graphs) and mannitol (MAN, right graphs) conditions. All results are expressed as mean ± SD of *n* = 6 technical replicates for each condition. For cultures containing human (h) BM-MSC-derived CM, results are shown for MSC from two different donors [Donor 1 (blue) and Donor 2 (green)]. MSC-control = Unconditioned MSC medium. MSC-CM (whole) = Non-extracellular vesicle (EV) depleted MSC-CM. MSC-CM (-EV) = Extracellular vesicle (EV) depleted MSC-CM. HCEC-control = Unconditioned human corneal endothelial cell medium. HCEC-CM = CM from human corneal endothelial cells. Statistical analyses performed by one-way ANOVA (for hBM-MSC versus control) and unpaired Student’s *t* test (for endothelial cell versus control). **** *p* < 0.0001, *** *p* < 0.001, * *p* < 0.05, ns = not significant

Early-passage human BM-MSC from healthy adult donors were shown to have expected surface marker profiles and osteogenic and adipogenic differentiation potential (see Additional file 8: Figure S5). To determine whether MSC-derived soluble products suppress RPTEC/TERT1 inflammatory responses, 10×-concentrated MSC-CM with and without depletion of extracellular vesicles (EVs) was added at 20% volume to RPTEC/TERT1 monolayers for the final 48 h of 5-day cultures in HG and MAN conditions (Fig. 3). Conditioned media of BM-MSC from two different donors were used. As shown, both MSC-CM preparations resulted in reduced secretion of IL-8, IL-6 and MCP-1 but not NGAL under HG and MAN conditions. Of note, the suppressive effect of MSC-CM on pro-inflammatory cytokines and chemokines was similar for CM preparations with and without EV depletion, suggesting that the effect was unlikely to be mediated by MSC-EV. In contrast, addition of CM from a non-MSC cell line (HCEC) resulted either in no reduction or in increased release of IL-6, IL-8, MCP-1 and NGAL by RPTEC/TERT1 monolayers.

### Indirect co-culture of human mesenchymal stromal cells causes a more potent inhibition of high glucose-induced inflammatory response of RPTEC/TERT1

**Fig. 4:**
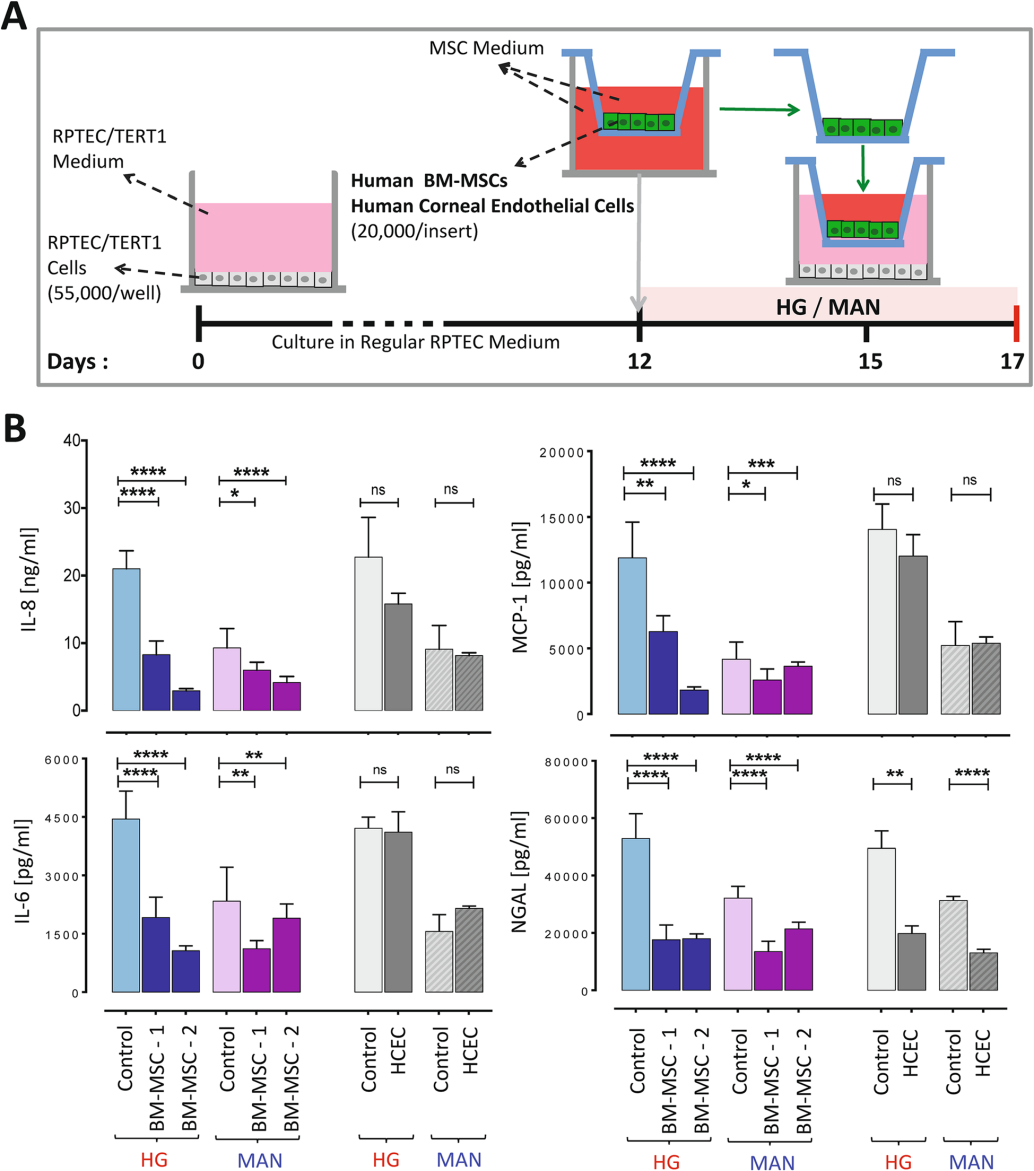
Indirect co-culture with human MSC results in potent inhibition of high glucose-induced secretion of inflammatory cytokines by RPTEC/TERT1 cell stable monolayers. **a** Schematic diagram of the experimental protocol. **b** Graphs depicting the concentrations of IL-8, IL-6, MCP-1 and NGAL in the supernatants from RPTEC/TERT1 cell monolayers cultured without (control) and with (cells) various transwell co-cultures during the final 2 days of 5-day cultures under high glucose (*D-glucose*, blue) and mannitol (D-mannitol, purple) conditions. Results are shown for co-cultures carried out with human bone marrow MSC from two different donors (BM-MSC-1 and BM-MSC-2) and with a non-MSC control cell line (HCEC, grey). All results are expressed as mean ± SD of *n* = 6 technical replicates for each condition. Statistical analyses performed by one-way ANOVA (for BM-MSC versus control) and unpaired Student’s *t* test (for HCEC versus control). ****p < 0.0001, ***p < 0.001, **p < 0.01, *p < 0.05, ns = not significant

Next, it was determined, using a transwell co-culture system, whether indirect contact of BM-MSC diminishes the RPTEC/TERT1 inflammatory response to HG and control culture conditions. As shown in Fig. 4, indirect contact with BM-MSC from two different donors (but not indirect contact with HCEC) for the final 2 days of a 5-day culture resulted in potent reductions of secretion of IL-8, IL-6 and MCP-1 by HG-exposed RPTEC/TERT1 cell monolayers as well as more modest reductions under MAN-exposed conditions. In contrast to the effect of MSC-CM addition, transwell co-culture with MSC (as well as with HCEC) resulted in potent reduction of NGAL release by the monolayers under HG and, to a lesser extent, MAN conditions.

### Prolonged exposure of RPTEC/TERT1 monolayers to high glucose concentration is associated with widespread transcriptional modifications that are modulated by indirect co-culture with MSC

**Fig. 5:**
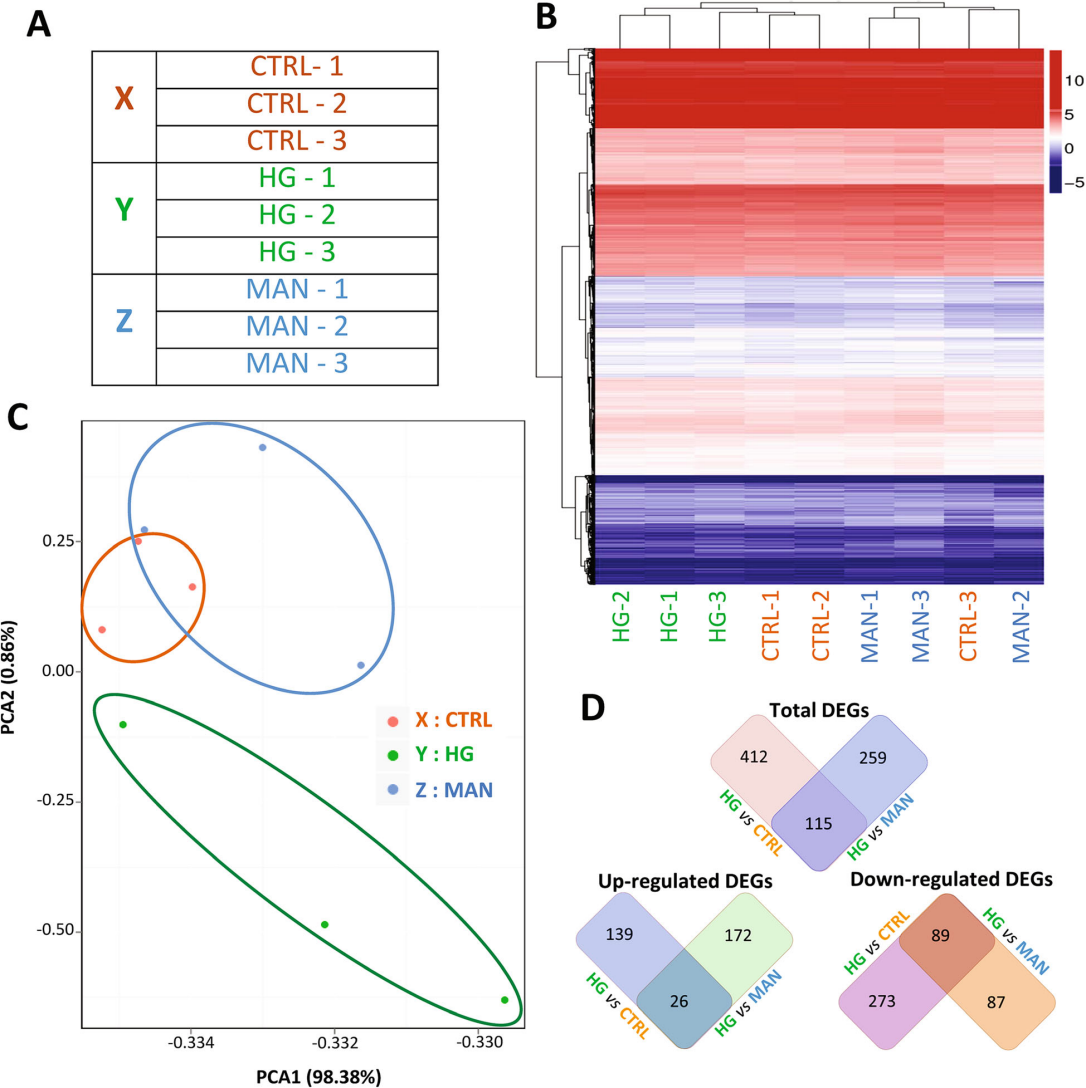
High-level bioinformatics analyses of RNA-seq transcriptional profiling of RPTEC/TERT1 monolayers following 5-day exposure to control, high glucose and mannitol culture conditions: **a** Table summarising the samples subjected to RNA-seq. **b** Unsupervised hierarchical clustering of the RNA-seq profiles of samples (nomenclature as per Fig. 4a). **c** Scatter plot of principal component analysis of the RNA-seq profiles. Each dot represents one sample (nomenclature as per Fig. 4a). **d** Figures illustrating the numbers of total upregulated and downregulated DEGs for high glucose (HG) vs. normal glucose control (CTRL) culture conditions and high glucose vs. mannitol control (MAN) culture conditions. Overlapping segments indicate the numbers of DEGs that were common to both comparisons

To more broadly characterise the effect of prolonged HG exposure, RNA-seq was performed on triplicate samples of mature RPTEC/TERT1 monolayers following 5-day culture in CTRL, HG and MAN culture conditions (Fig. 5a). For this analysis, hierarchical clustering and principal component analyses demonstrated partial overlap between CTRL and MAN transcriptional profiles with separation of the HG profile from both controls (Fig. 5b, c). In total, 527 and 374 genes were differentially expressed in HG-exposed compared with CTRL- and MAN-exposed RPTEC/TERT1 monolayers respectively, with 115 (26 upregulated and 89 downregulated) DEGs being common to both comparisons (Fig. 5d). In contrast, only 314 DEG were identified for MAN compared to CTRL samples. Full lists of the DEGs from these comparisons are provided in Additional file 9: Table S2, Additional file 10: Table S3 and Additional file 11: Table S4. Biological process and pathway enrichment analysis of the DEGs from HG vs. MAN comparison indicated a predominance of processes and pathways related to inflammation and infection (see Additional file 12: Figure S6). Prominent among the modulated pathways were TNF-signalling pathway, cytokine-cytokine receptor interaction and NOD-like receptor signalling pathway. Among 15 DEGs within the TNF signalling pathway, multiple inflammation-related transcripts were present including IL1-β, TNF, IL-6, L-selectin, colony stimulating factor 2 (CSF-2), IL-8, C-C motif chemokine ligand 2 (CCL2; also called MCP-1), lymphotoxin beta (LTB) and cellular inhibitor of apoptosis 1 and 2 (cIAP1/2) (see Additional file 13: Figure S5).

**Fig. 6:**
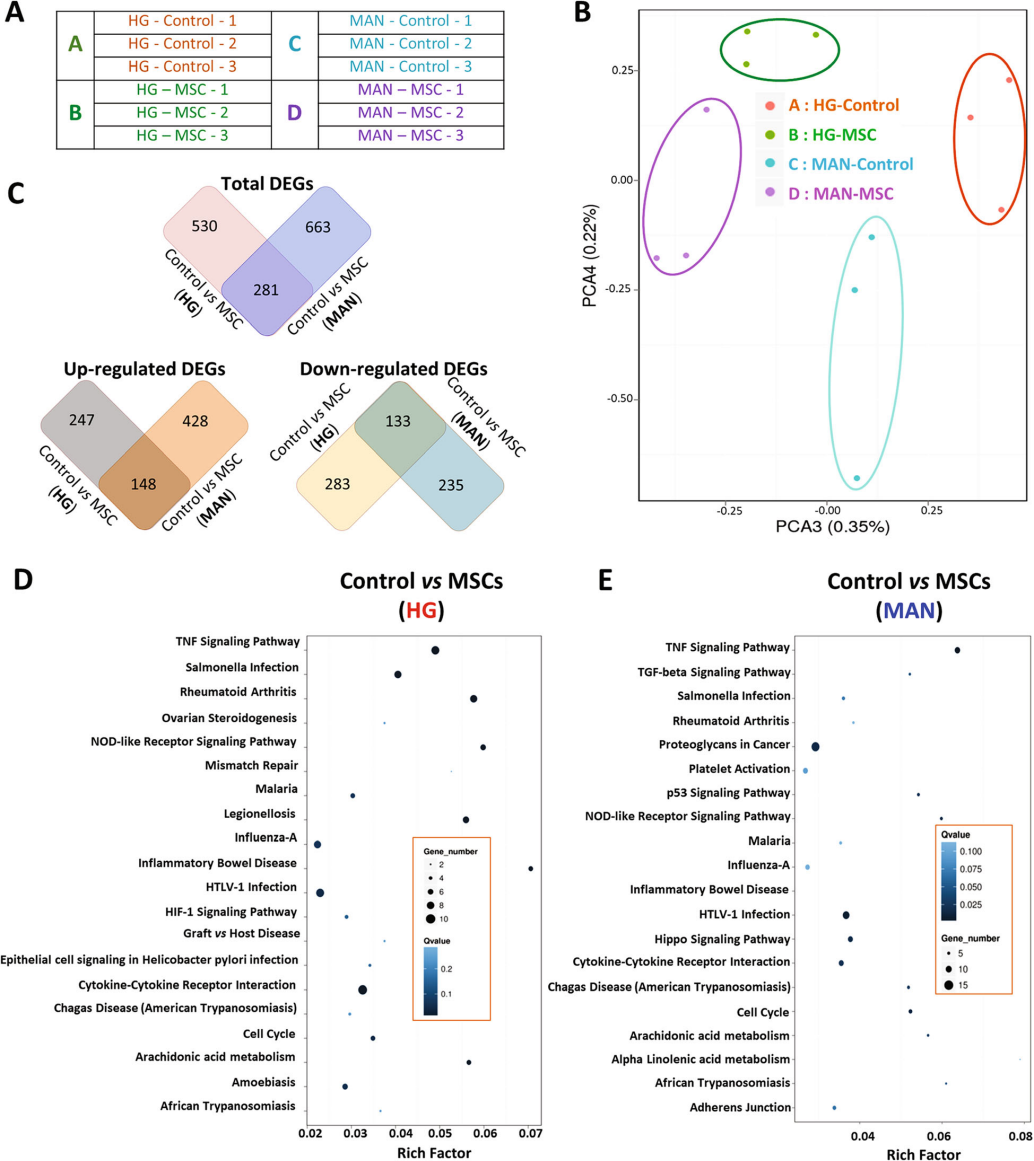
Bioinformatics analyses of RNA-seq transcriptional profiling of RPTEC/TERT1 monolayers following 5-day exposure to high glucose (HG) and mannitol (MAN) culture conditions in the presence or absence of MSC-containing transwells*:* **a** Table summarising the non-MSC- (control) and MSC-co-cultured (MSC) samples subjected to RNA-seq. **b** Scatter plot of principal component analysis of the RNA-seq profiles. Each dot represents one sample (nomenclature as per Fig. 5a). **c** Figures illustrating the numbers of total upregulated and downregulated DEGs for control vs. MSC-co-cultured RPTEC/TERT1 cells under high glucose (HG) and mannitol (MAN) culture conditions. Overlapping segments indicate the numbers of DEGs that were common to both comparisons. **d**, **e** Scatter graphs indicating the enrichment factors and number of DEGs of the top 20 enriched pathways for control vs. MSC-co-cultured RPTEC/TERT1 monolayers under HG (**d**) and MAN (**e**) conditions based on KEGG pathway enrichment analysis

Next, a similar RNA-seq analysis was carried out on triplicates of RPTEC/TERT1 monolayers following 5-day exposure to HG and MAN conditions in the presence or absence of indirect (transwell) MSC co-culture for the final 2 days (Fig. 6a). As shown in Fig. 6b, principal component analyses demonstrated distinctive transcriptional profiles for the four conditions—indicating broad modulatory effects of indirect MSC co-culture on RPTEC/TERT1 gene expression under both HG and osmotic control conditions. In total, 811 genes were differentially expressed in MSC-co-cultured vs. non-MSC-(control)-cultured RPTEC/TERT1 monolayers under HG condition of which 395 were upregulated and 416 were downregulated. For the MAN condition, 916 genes were differentially expressed between non-MSC (control)-co-cultured vs. MSC-co-cultured cells of which 576 were upregulated and 368 were downregulated. A total of 281 genes (148 upregulated and 133 downregulated) were modulated by MSC co-culture under both HG and MAN conditions (Fig. 6c). Among the latter were multiple transcripts of relevance to inflammatory response including those encoding CCL2/MCP-1, chemokine (C-X-C motif) ligand 2 (CXCL2), enolase-2, interleukin 2 receptor subunit gamma (IL2RG), IL1-β, prostaglandin-endoperoxide synthase 1 (PTGS1), lipocalin 2 (LCN2; also called NGAL), S100 calcium binding protein A14 (S100A14), L-selectin (SELL) and superoxide dismutase 2 (SOD2) (Full lists of the DEGs from these comparisons are provided in Additional file 14: Table S5 and Additional file 15: Table S6).

**Fig. 7:**
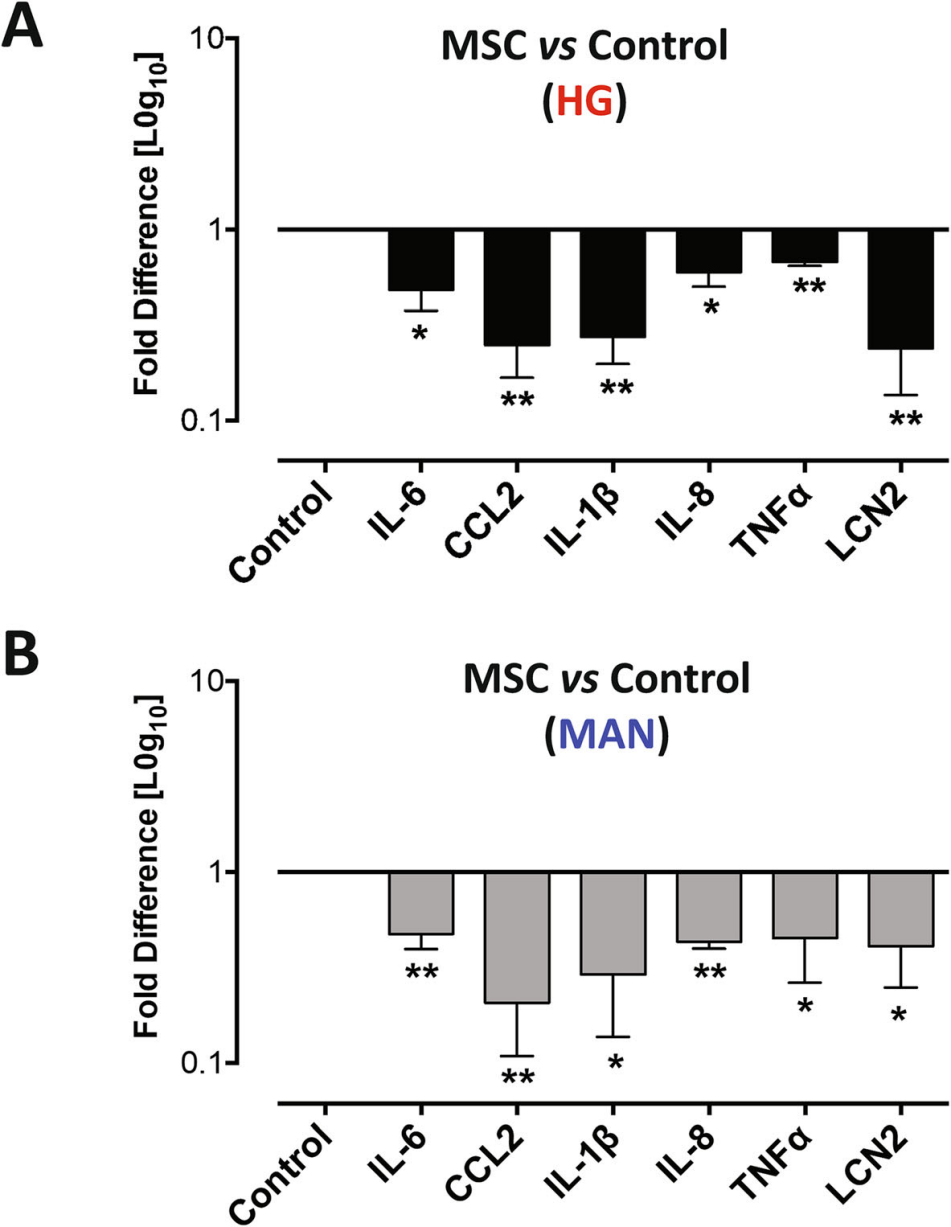
Validation of the effect of BM-MSC on RPTEC-TERT1 expression of inflammation-related transcripts. Graphical representations of qRT-PCR analysis of the relative expression of RNA transcripts for IL-6, MCP-1 (CCL2), IL-1β, IL-8, TNFα and NGAL (LCN2) in RPTEC/TERT1 cell monolayers cultured for 5 days in high glucose (HG) (**a**) or mannitol (MAN) (**b**) in the presence of BM-MSC-containing transwells (MSC) or no cells (control) for the final 2 days of culture. Results are expressed as mean ± SD for *n* = 3 samples per condition of the fold difference for MSC vs. control as determined relative to the housekeeping gene RPLP0 using the 2^–∆∆Ct^ method. Statistical analysis by Student’s unpaired *t* test, **p < 0.01, *p < 0.05, ns = not significant

Biological process (presented in Additional file 16: Figure S8) and pathway enrichment (Fig. 6d, e) analyses of the control-co-cultured vs. MSC-co-cultured RNA-seq profiles under HG and MAN conditions indicated prominent modulation of a range of inflammation-related responses. Notable among the pathways that were significantly modulated by indirect MSC co-culture in the presence of HG were TNF-signalling pathway, cytokine-cytokine receptor interaction, NOD-like receptor signalling pathway and arachidonic acid metabolism. Transcripts involved in these pathways included those encoding IL1β, TNF, interleukin-6 (IL-6), IL18R, SELL, CSF2, TNF Alpha Induced Protein 3 (TNFAIP3), IL-8, CCL2, CXCL2, LTB and cIAP1/2 (Additional file 17: Figure S9 provides a representative example). Quantitative RT-PCR analysis confirmed the RNA-seq results for 5 inflammation-related transcripts (Fig. 7).

Overall, it was concluded from the transcriptional profiling analyses of RPTEC/TERT1 monolayers that prolonged exposure to HG results in enhanced expression/activity of a wide range of genes and pathways associated with immune response and inflammation and that proximity to viable human BM-MSC for 48 h exert a broad inhibitory effect on many of these inflammatory pathways—likely through the production of soluble mediators. Furthermore, the anti-inflammatory effect of MSC on proximal tubular epithelial monolayers is not exclusive to a HG-induced pro-inflammatory response, as it was also evident under MAN (osmotic control) conditions.

### Indirect mesenchymal stromal cell contact modulates pro-inflammatory cross-talk between high glucose-exposed RPTEC/TERT1 cell monolayers and human macrophages

**Fig. 8:**
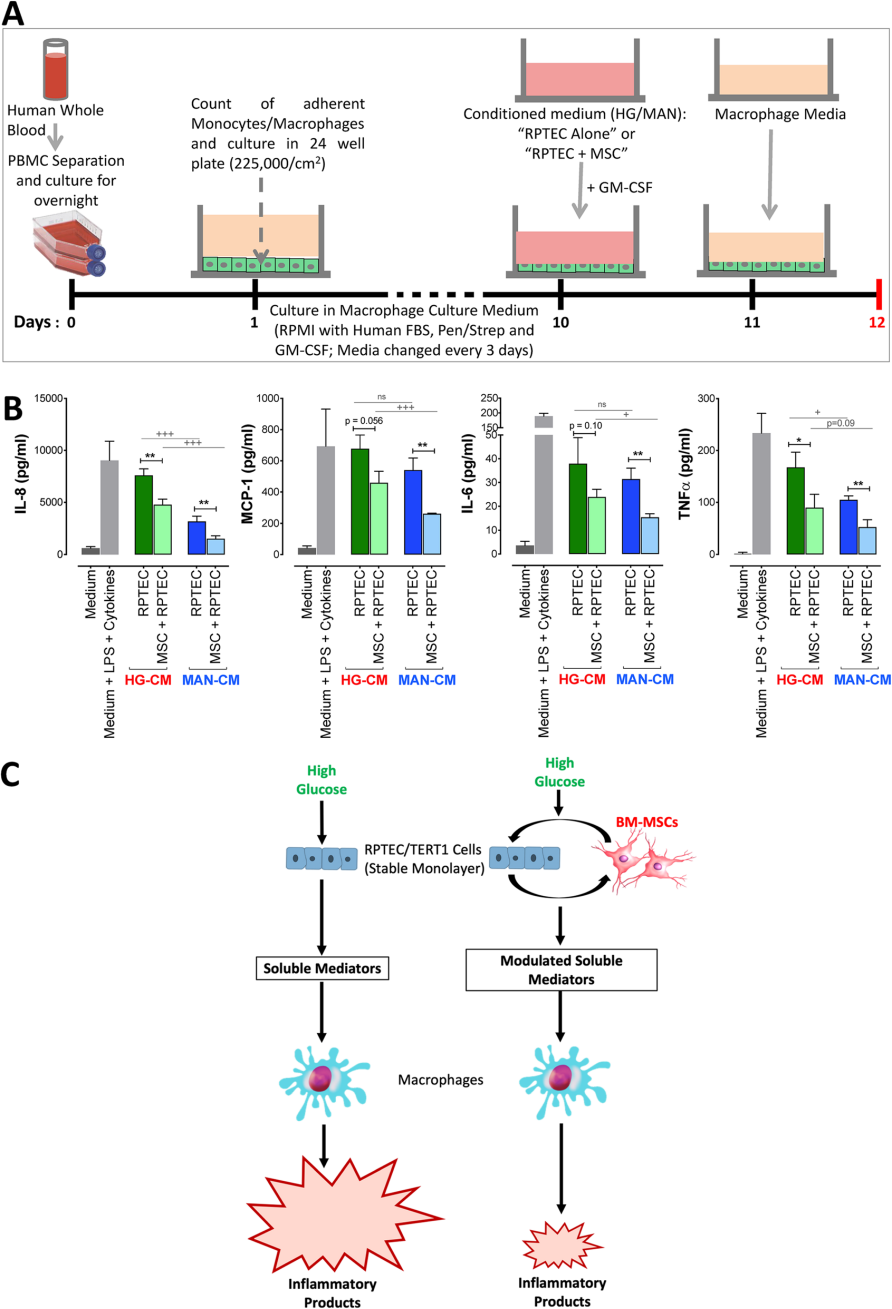
Indirect mesenchymal stromal cell contact modulates pro-inflammatory cross-talk between high glucose-exposed RPTEC/TERT1 cell monolayers and human macrophages*.* **a** Schematic diagram of the experimental protocol. **b** Graphs depicting the concentrations of IL-8, MCP-1, TNFα and IL-6 in the supernatants from monocyte-derived macrophages cultured with macrophage medium alone (medium), macrophage medium containing a stimulatory cocktail of LPS, IFNγ and TNFα (medium + M1 stimuli) and conditioned media from 48 h cultures of RPTEC/TERT1 cells alone (RPTEC) or RPTEC/TERT1 cells co-cultured with MSC-containing transwells (MSC + RPTEC) under high glucose (HG) and mannitol (MAN) conditions. Results for macrophages exposed to conditioned media from high glucose (HG) condition are represented in green and those from mannitol (MAN) are represented in blue bars. Results are expressed as mean ± SD of *n* = 3 technical replicates for each condition. Statistical analyses performed unpaired Student’s *t* test (* for MSC versus control; + for HG versus MAN). ****/++++*p* < 0.0001, ***/+++*p* < 0.001, **/++*p* < 0.01, */+*p* < 0.05, ns = not significant. **c** Schematic representation of the effect of high glucose on RPTEC, the subsequent inflammatory response of macrophages (left) and the modulatory effect of indirect contact between RPTEC and MSC on the downstream macrophage response (right)

Finally, we sought to determine whether soluble products released during RPTEC/TERT1-MSC co-cultures under HG or MAN conditions exert anti-inflammatory effects on human macrophages. An experiment was carried out in which in vitro-differentiated, human monocyte-derived macrophages were cultured for 24 h in the presence of CM from RPTEC/TERT1 monolayers that had been exposed for 5 days to HG or MAN conditions in the presence or absence of BM-MSC-containing transwells for the final 2 days (Fig. 8a). The CM-exposed macrophages were then washed and allowed to remain in culture in macrophage medium alone for a further 24 h, following which production of pro-inflammatory cytokines and chemokines was quantified by ELISA of the macrophage culture supernatants. Macrophages cultured for 24 h in the absence and presence of a stimulatory cocktail of LPS, IFNγ and TNFα served as additional controls. As shown in Fig. 8b, primary human macrophages showed significant elevation in the levels of those cytokines/chemokines when exposed for 24 h to CM from RPTEC/TERT1 cells under HG compared to MAN condition. When macrophages were exposed to CM from the equivalent RPTEC/TERT1-MSC co-cultures, reduced secretion of IL-8, MCP-1, IL-6 and TNFα was observed—being most significant for IL-8 and TNFα. The magnitude of induction of IL-8, MCP-1 and TNFα following macrophage exposure to HG-CM from RPTEC alone was comparable to that induced by stimulation with LPS, IFNγ and TNFα. The results of qRT-PCR performed on RNA extracted from macrophages at the end of the experiment were inconclusive due to variability across technical replicates (*data not shown*). It was not possible, therefore, to confirm whether reduced macrophage secretion of inflammatory mediators was mediated specifically through suppression of transcription. A conceptual model for MSC modulation of HG-induced RPTEC/macrophage cross-talk based on the experimental results of the study is shown in Fig. 8c.

## Discussion

A growing body of evidence indicates that RPTEC play an important role in the pathogenesis of DKD [17, 21, 25, 39–41]. In vitro observations using immortalised cell lines such as HK-2 provide a valuable, simplified system in which to explore specific effects of the diabetic milieu on renal epithelial cell biology but have limitations when comparing with primary RPTEC. Recent studies suggest that stable monolayers of RPTEC/TERT1 cells provide superior phenotypic and functional comparability to primary tubular epithelium [24–27]. In our hands, in keeping with previous reports [24–27], RPTEC/TERT1 cells formed stable monolayers after 12 days with an epithelial-like, cobblestone morphology which remained stable following a further 5-day exposure to HG or MAN. In this system, we observed that prolonged HG exposure induced a heightened inflammatory phenotype characterised by progressively increasing secretion of IL-6, IL-8 and MCP-1 along with greater release of the tubular injury biomarker NGAL. For IL-6 and MCP-1, our observations are comparable to those of Tang et al. in confluent, growth-arrested primary RPTEC, albeit only becoming evident compared to normal glucose and MAN controls following a longer exposure time [42]. Importantly, although RNA-seq analysis revealed a range of other gene expression changes, we did not observe overt toxicity or evidence of loss of epithelial-like morphology of the monolayers during the period of exposure to HG or MAN.

Consistent with the HG-induced increase in IL-6 secretion by RPTEC/TERT1 monolayers, patients with DKD have increased renal IL-6 expression which correlates with kidney hypertrophy and albuminuria [43, 44] and increases with stage of DKD [45]. Exposure to HG for 96 h or more also resulted in increased secretion of IL-8 by RPTEC/TERT1 cells. Of interest, Tashiro et al. reported increased levels of IL-8 in urine samples from the patients with early-stage DKD [46] while others observed increased IL-8 production by tubular epithelial cells via activation of NF-κB, ERK1/2 and STAT1 signalling in a glycated-albumin-induced diabetes model [47]. Similarly, NF-κB-dependent upregulation of MCP-1 in albumin-treated RPTEC and increased MCP-1 expression in kidney biopsy samples from patients with DKD have been reported [48, 49]. In a hyperglycaemic environment, increased MCP-1 production by mesangial and tubular epithelial cells has also been observed in vitro [14, 50]. Functionally, tubular cell-derived MCP-1 triggers infiltration of the interstitium by monocytes which, along with resident macrophages, secrete additional pro-inflammatory cytokines [16]. We also describe here a progressive increase in the release of NGAL, a comparatively new biomarker of DKD, following exposure of RPTEC/TERT1 stable monolayers to HG for 80 h or more. Of relevance, NGAL is elevated in serum and urine of DKD patients [51–53]. Furthermore, Nielsen et al. demonstrated an association between urinary NGAL and rate of eGFR decline with the implication that renal tubular cells represent the major source of the urinary NGAL release [52]. Taken together, these observations support the relevance of our findings in the RPTEC/TERT1 culture system to renal interstitial inflammatory events related to hyperglycaemia and diabetes.

Human bone marrow-derived MSC produce immunomodulatory and cytoprotective mediators that act in a paracrine manner on a range of target cells to downregulate the production of pro-inflammatory cytokines and inhibit inflammatory signalling pathways [36, 54–56]. In animal models of diabetes, hBM-MSC infusions reduced matrix deposition in the mesangium [54] and glomerulus [56]. Nagaishi et al. studied the effects of BM-MSC treatment in insulin-deficient and insulin-resistant DKD models and demonstrated the benefits following systemic administration of both cells and CM [37]. Similarly, Lv et al. reported reduced intra-renal expression of IL1-β, IL-6, TNFα and MCP-1 following intravenous injection of MSC in a streptozotocin-induced rat model of DKD [55]. The reduction in MCP-1 and other inflammatory cytokines was associated with decreased macrophage infiltration and reduced severity of renal structural injury. In keeping with these in vivo findings, our experiments to evaluate the effect of human BM-MSC on human RPTEC/TERT1 cells in the setting of prolonged HG exposure revealed anti-inflammatory effects both of MSC-CM and of indirect (transwell) co-culture of RPTEC-TERT1 cells with MSC—with the latter being more potent. To our knowledge, this is the first study to show the effect of MSC and their soluble products on RPTEC/TERT1 cells as an in vitro model of diabetic proximal tubulopathy. The results indicate that MSC have the potential to modulate RPTEC dysfunction in DKD either from a distant anatomical site or locally within the kidney. Perhaps surprisingly, MSC-CM depleted of EV had comparable anti-inflammatory effects to those of EV-containing CM, indicating that the paracrine anti-inflammatory effects were unlikely to be mediated by MSC-derived EV despite evidence that they may have reno-protective properties [57, 58]. Whether purified MSC-EV may have a distinct modulatory effect on HG-induced inflammatory responses of RPTEC/TERT1 monolayers and, if so, what mechanisms underlie such an effect remain an interesting question that merits further investigation.

Although knowledge of the mechanism of action of MSC in vivo is incomplete, the paracrine effect of a range of inducible factors is well established. Transcriptomic analysis of RPTEC/TERT1 cell monolayers demonstrated that prolonged HG exposure was associated with gene expression changes that were enriched for biological processes and signalling pathways of relevance to immune response and inflammation. We also observed differential gene expression between MAN and CTRL culture conditions that likely reflect the influence of increased osmolality on RPTEC-TERT1 monolayers. However, the findings that higher numbers of DEGs were identified for HG vs CTRL than for MAN vs CTRL and that the HG condition separated fully from CTRL and MAN on a principal component analysis of the RNA-seq data are in keeping with a distinct effect of HG rather than a non-specific effect of increased osmolality. Interestingly, under both HG and MAN conditions, the modulatory effects of indirect co-culture with MSC on the RPTEC-TERT1 monolayer transcriptome were also enriched for immune/inflammatory pathways—with the TNF signalling pathway being particularly prominent. Modulation of the HG-induced TNF signalling pathway following MSC co-culture included reversion toward control levels of the increased expression of genes related to leukocyte recruitment (CCL2/MCP-1, CXCL1, CXCL2, CXCL3), leukocyte activation (CSF-2), inflammatory cytokines (IL-1β, IL-6) and cell adhesion (E-Selectin). Results for several inflammatory mediators/products (IL-6, IL-8, CCL2/MCP-1 and LCN2/NGAL) were consistent across RNA-seq, qRT-PCR and ELISA. An overall implication of these results is that human MSC, delivered directly to the kidney or transmigrating to renal interstitial spaces in the setting of diabetes/hyperglycemia, are likely to initiate a paracrine interaction with RPTEC that potently downregulates chronic inflammatory signalling. Furthermore, we provide evidence that the modulatory effect of MSC on release of inflammatory mediators by RPTEC in the setting of HG may have downstream effects on the response of monocyte-derived macrophages which are known to infiltrate the kidney and mediate renal interstitial damage during DKD [31]. Inflammatory stimuli to RPTEC have been shown to result in cross-talk with intra-renal macrophages through a number of mechanisms including cytokine/chemokine secretion, transfer of bioactive molecules in extracellular vesicles, release of danger-associated molecular patterns and triggering of specific forms of necrotic cell death [59–61].

A relatively large body of literature exists in support of the potential benefits of MSC to slow the progression of DKD through anti-inflammatory mechanisms [2]. To date, however, only one early-phase clinical trial of a stromal cell therapy has been completed in human subjects with DKD. This demonstrated that, in relatively advanced DKD due to type 2 DM, intravenous injection of allogeneic bone marrow-derived Stro3^+^ mesenchymal precursor cells was safe up to 24 weeks post-administration and was associated with preliminary evidence of efficacy including decreased serum IL-6 compared to placebo [62].

In addition to the obvious caveat that experimental work conducted in vitro using a cell line will require validation in more physiologically relevant systems, some specific limitations of the study must be acknowledged. Firstly, while our culture conditions and experimental durations are quite comparable to those used by other investigators who have extensively characterised RPTEC/TERT1 cell epithelial monolayer formation [26], they cannot be said to be identical. Thus, the influence of variability in culture conditions on the physiological relevance of our experimental findings cannot be fully determined. Nonetheless, it is clear that, in our hands, the cells consistently generated typical, mature epithelial-like monolayers and that subsequent 4–5-day exposure of the monolayers to HG and MAN did not result in overt cellular toxicity or transformation to a non-epithelial phenotype. Lack of elucidation of a clear intracellular signalling mechanism to explain HG-induced changes in the expression and secretion of inflammatory mediators by RPTEC/TERT1 cell monolayers (and its suppression by factors released by MSC) is a second limitation. While involvement of alternative signalling pathways, of post-transcriptional/post-translation modification of the gene products or of altered intracellular trafficking/secretion of inflammatory mediators may explain the observations, it is also possible that experiments carried out at earlier or later time-points could have revealed increased activity of one or more predicted signalling pathways under HG conditions. Thirdly, while our experimental focus for the study was on HG-induced inflammatory response of RPTECT/TERT1 monolayers and its modulation by MSC, other potentially important aspects of the altered transcriptomic profiles revealed by RNA-seq have not been functionally validated and explored. By sharing the full lists of DEG we have identified under different experimental conditions, we anticipate that these data can be further exploited through deeper functional investigation by others in the field. Finally, a limitation of our experiments involving the transfer of conditioned media from RPTEC/MSC co-cultures to primary macrophages is that they do not exclude the possibility that MSC- and RPTEC/TERT1-derived soluble mediators act on macrophages independently of each other rather than as a result of a distinctive MSC/RPTEC cross-talk. Thus, additional experiments beyond the scope of the current study will be needed to dissect the individual mechanistic contributions of RPTEC and MSC on downstream activity of macrophages and to investigate the potency of MSC to regulate RPTEC signalling to macrophages across a range of pathogenic conditions.

## Conclusions

Our current study reveals a predominantly pro-inflammatory effect of prolonged HG exposure on stable monolayers of the RPTEC/TERT1 cell line during a 5-day time window that is substantially modulated at a transcriptional level by soluble products of human MSC—particularly when the two cell types were cultured in close proximity. Experimentally, we also show that the combined secretome of RPTEC/MSC co-cultures has the capacity to dampen macrophage inflammatory response under HG conditions. These results provide a novel platform for better understanding anti-inflammatory mechanisms of action of MSC in in vivo studies and clinical trials of DKD. Our in vitro system also has the potential for identifying new targets of intervention for diabetes-associated proximal tubulopathy and pro-inflammatory epithelial cell/macrophage cross-talk. Further studies are also needed to elucidate the mechanisms whereby HG exposure induces a prolonged increased secretion of cytokines, chemokines and markers of inflammation by RPTEC in the absence of persistent over-activity of NF-κB, MAPK and other expected intracellular signalling pathways.

## Supplementary information

**Additional file 1.** Supplementary methods document.

**Additional file 2:** **Table S1.** List of primer sequences used for qRT-PCR.

**Additional file 3:** **Figure S1.** Effect of High D-Glucose on RPTEC/TERT1 Cells. A. Effect of seeding density on growth of RPTEC-TERT-1 cells. Here, three different cell numbers per square centimeter (cm^2^), labelled as Low (12500 cells/cm^2^), Medium (12500 cells/cm^2^) and High (12500 cells/cm^2^), are represented as Blue, Green and Grey coloured lines respectively. Cells grown in 24 well plate for different time points and number of cells counted by haemocytometer using trypan blue and represented as cells/cm^2^. B. Effect of high glucose on growth of RPTEC-TERT-1. Cells were seeded at 27500/cm^2^. and counted at different time points using haemocytometer. C. Downstream experimental plan. RPTEC-TERT-1 cells cultured at 27500/cm^2^, media replaced every second day. From day 12, 25 mM of D-Glucose or D-Mannitol (osmotic control to glucose) was administered for different time periods Effect of high Glucose on RPTECs for downstream comparisons of glucose versus controls: formation of stable monolayer by microscope; D. Similar cell size confirmed by flow cytometry.

**Additional file 4: Figure S2.** Combined effect of high glucose and albumin on RPTEC/TERT1 inflammatory responses**. A**. Schematic diagram of the experimental protocol. In brief, RPTEC-TERT-1 cells cultured at 27500/cm^2^, medium was replaced every second day. From day 12, cells were grown in high-glucose or control conditions (CTRL/HG/MAN) with or without 100 μg/ml human serum albumin. Mediium was replaced at day 15 for a further two days. **B**. Mean ± SD levels of inflammatory mediators including IL-8 (top left), IL-6 (top right), MCP-1 (bottom left) and NGAL (bottom right) in the supernatants are represented in grey (CTRL), blue (HG) and green (MAN) bars. Bright colours represent the levels in samples when treated without albumin. * denoted unpaired t-tests for CTRL vs HG, HG vs MAN, MAN vs CTRL. ¥ denoted ANOVA to analyse differences between CTRL, HG and MAN. ****^/¥¥¥¥^ *p* <0.0001, ***^/¥¥¥^ *p* <0.001, **^/¥¥^ *p* <0.01, *^/¥^ *p* <0.05.

**Additional file 5: Figure S3.** Combined effect of high glucose and IL-1β as inflammatory cytokine stimuli on RPTEC/TERT1 responses. A. Schematic diagram of the experimental protocol. In brief, RPTEC/TERT1 cells were cultured at 27500/cm^2^, medium was replaced every second day. From day 12, cells were grown in high-glucose or control conditions (CTRL/HG/MAN). Medium was replaced at day-15. In addition to CTRL/HG/MAN, cells were treated with- or without- 1 ng/ml IL-1β for the final two days; B. Mean ± SD levels of inflammatory mediators including IL-8 (top left), IL-6 (top right), MCP-1 (bottom left) and NGAL (bottom right) in the supernatant samples represented in grey (CTRL), blue (HG) and green (MAN) bars. Bright colours represent the levels in samples when treated without IL-1β. * denoted unpaired t-tests for CTRL vs HG, HG vs MAN, and MAN vs CTRL. ¥ denoted ANOVA to test for differences between CTRL, HG and MAN. ****^/¥¥¥¥^ p <0.0001, ***^/¥¥¥^ p <0.001, **^/¥¥^ *p* <0.01, *^/¥^ *p* <0.05.

**Additional file 6: Figure S4.** A*:* Exposure of RPTEC/TERT1 cells to high-Glucose did not alter expression in any common inflammatory signalling molecules. RPTEC-TERT-1 cells were cultured at 27500/cm^2^, medium was replaced every second day. From day 12, cells were grown in high-glucose or control conditions (CTRL/HG/MAN) for 24, 48 and 96 hours. Using western blotting, cell pellets were harvested for investigating the expressions of different signalling proteins including: total and phosphorylated forms of p65 NFkB (nuclear factor kappa B – p65 sub unit), p38 MAPK (P38 mitogen-activated protein kinase), ERK-1/2 (extracellular signal–regulated kinase 1/2), STAT-1 (Signal transducer and activator of transcription 1), PKCα (Protein kinase C alpha) and total PPAR-γ (Peroxisome proliferator-activated receptor gamma ) as well as housekeeping protein β-Actin (Beta Actin).

**Additional file 7: Figure S4.** B: Semi-quantitative analyses of the western blots as in Figure S4A*.* ImageJ software was used to perform semi-quantitative analysis of the blots. The area and its corresponding percentage of blots were calculated. Densitometric data were then normalized for the housekeeping protein followed by further normalization relative to the control. Statistical analyses were performed using GraphPad prism. Results were expressed as the Mean±SD for three technical replicates per condition. *p* values ≤0.05 were considered significant at: *p<0.05, **p<0.01, ***p<0.001.

**Additional file 8:** **Figure S5.** Human bone-marrow derived MSCs showed its cell surface characteristics and were able to differentiate. A. Immunophenotyping of cultured human BM-MSCs for different specific cell surface markers by flow cytometry. B. Osteogenic and adipogenic differentiation capacity of BM-MSCs. Unpaired t test with Welch's correction *p<0.05; **p<0.01; ***p<0.001.

**Additional file 9:** **Table S2.** List of DEGs with significant Fold Changes in High-Glucose vs Mannitol.

**Additional file 10:** **Table S3.** List of DEGs with significant Fold Changes in High-Glucose vs Control.

**Additional file 11:** **Table S4.** List of DEGs with significant Fold Changes in Mannitol vs Control.

**Additional file 12:** **Figure S6.** Involvement of biological processes and signalling pathways associated with differentially expressed genes in high-glucose exposed RPTEC/TERT1 cells. (A) Pathway Enrichment analysis of differentially expressed genes (DEGs) performed based on KEGG database; (B) Scatter plot for the top 20 pathways of KEGG enrichment results. Here, X axis represents number of DEGs. Y axis represents second KEGG pathway terms. All second pathway terms were grouped in top pathway terms indicated in different colour.

**Additional file 13:** **Figure S7.** Up-regulation of genes for inflammatory cytokines following exposure of RPTEC/TERT1 cells to high-glucose (TNF-Signalling pathway is a representative example). Up-regulated genes are marked with red borders. Unchanged genes are marked with black borders.

**Additional file 14:** **Table S5.** List of DEGs with significant Fold Changes in RPTEC/TERT1 cells without and with MSC co-culture in high glucose (HG) condition.

**Additional file 15:** **Table S6.** List of DEGs with significant Fold Changes in RPTEC/TERT1 cells without and with MSC co-culture in mannitol (MAN) condition.

**Additional file 16:** **Figure S8.** Differential Expression of Genes in Presence of Indirect Contact of MSCs. Pathway Enrichment Analysis of Differentially Expressed Genes (DEGs) performed based on KEGG database by pairwise analysis. Here, X axis represents number of DEGs. Y axis represents second KEGG pathway terms. All second pathway terms were grouped in top pathway terms indicated in different colour.

**Additional file 17:** **Figure S9.** TNF-Signalling pathway as a representative example of the signalling pathways involved in MSC mediated anti-inflammatory effects (in high-glucose environment). Here, down-regulated genes are marked with green borders and unchanged genes are marked with black borders.

## Data Availability

RNA-seq data will be made publically available through GEO upon publication. All other datasets used and/or analysed during the current study are available from the corresponding author on reasonable request.

## Abbreviations

AGE: Advanced glycation end-products
BM-MSC: Bone marrow-derived mesenchymal stromal cells
CM: Conditioned medium
CTRL: Control
DEG: Differentially expressed gene
DKD: Diabetic kidney disease
DM: Diabetes mellitus
DMEM: Dulbecco’s modified Eagle medium
ESRD: End-stage renal disease
EV: Extracellular vesicle
FCS: Foetal calf serum
GM-CSF: Granulocyte macrophage colony stimulating factor
HCEC: Human corneal endothelial cell
HG: High glucose
HSA: Human serum albumin
hTERT: Human telomerase reverse transcriptase
IFNγ: Interferon gamma
IL: Interleukin
MAN: Mannitol
MCP-1: Monocyte chemoattractant protein 1
MSC: Mesenchymal stromal cell
NGAL: Neutrophil gelatinase-associated lipocalin
PI: Propidium iodide
RIN: RNA integrity number
RNA-seq: RNA sequencing
ROS: Reactive oxygen species
RPTEC: Renal proximal tubular epithelial cell
TNFα: Tumour necrosis factor alpha
