# Supplementary material for: Human mesenchymal stromal cells broadly modulate high glucose-induced inflammatory responses of renal proximal tubular cell monolayers
Source: Stem Cell Res Ther. 2019 Nov 19;10:329. doi: 10.1186/s13287-019-1424-5 (PMC6862760; doi:10.1186/s13287-019-1424-5)
Supplement: Supplementary file 1 — Additional file 1. Supplementary methods document. [file 13287_2019_1424_MOESM1_ESM.docx]

**SUPPLEMENTARY METHODS**

**Human mesenchymal stromal cells broadly modulate high glucose-induced inflammatory responses of renal proximal tubular cell monolayers**

Md Nahidul Islam^1^, Tomás P. Griffin^1,2^, Elizabeth Sander^1^, Stephanie Rocks^1^, Junaid Qazi^1^, Joana Cabral^1^, Jasmin McCaul^3^, Tara McMorrow^3^, ^†^Matthew D. Griffin^1^

^1^ Regenerative Medicine Institute (REMEDI) at CÚRAM Centre for Research in Medical Devices, School of Medicine, National University of Ireland Galway, Galway, Ireland

^2^ Centre for Endocrinology, Diabetes and Metabolism, Galway University Hospitals, Galway, Ireland.

^3^ School of Biomolecular and Biomedical Science, Conway Institute, University College Dublin, Ireland.

**Mesenchymal stromal cell differentiation assays**

Differentiation of MSC to osteogenic and adipogenic phenotypes was performed using StemPro differentiation kits (Gibco) according to the manufacturer’s instructions. After 21 days of incubation, differentiation of MSC into osteoblasts was confirmed by Alizarin Red S staining (Sigma) and differentiation into adipocytes was confirmed by by Oil-Red-O staining (Sigma) using well-described protocols.

**Preparation of conditioned media**

Mesenchymal stromal cells were plated at 1x10^6^ cells/ml into a T175 flask (Sarstedt) and incubated at 37^o^C in 5% CO_2_. After 3 days, MSC conditioned medium (MSC-CM) was collected and centrifuged at 400 x g for 5 minutes to remove floating cells and debris. The MSC-CM was then centrifuged at 2000 x g for 20 minutes to be cleared of apoptotic bodies and filtered through a filtropur 0.2µm vacuum filter bottle. Half of the total volume of MSC-CM was then ultra-centrifuged at 100,000 x g for 1 hour and 15 minutes to remove MSC-derived EVs. Both Total- and EV-depleted MSC-CM were then concentrated 10-times by spin concentrator (Agilent Technologies, Santa Clara, CA, USA) with cut-off of 5kDa following manufacturer’s instructions.

**ELISA protocol**

Capture antibody was coated onto 96-well ELISA plate (Nunc Maxisorp^TM^, Roskilde, Denmark) for 18 hours at room temperature (RT) followed by blocking with 1% BSA (Sigma) for 2 hours at RT. Samples/standards were added and incubated overnight at 4^0^C. Samples were diluted as necessary for accurate quantification. The plates were washed prior to the addition of biotin-conjugated detection antibody for 2 hours at RT. Streptavidin-HRP was added and incubated for 30 minutes at RT following which TMB-substrate (EMD Millipore Corp, Billerica, MA, USA) was added to observe blue colour. Enzyme-substrate reaction was stopped by 1M sulfuric acid (Sigma) and optical density was measured at 450nm and subtracted from the background at 630nm using microplate reader (Varioskan Flash, Thermo Scientific, UK). Concentrations of the various analytes were calculated from standard curves.

**Western Blotting**

The rabbit anti-human primary antibodies for NF-κB p65 (monoclonal: D14E12), Phospho-NF-kB p65 (Ser536; monoclonal: 93H1), p38 MAPK (polyclonal), Phospho-p38 MAPK (Thr180/Tyr182; monoclonal: D3F9), p44/42 MAPK (Erk1/2; polyclonal), Phospho-p44/42 MAPK (Erk1/2; Thr202/Tyr204; monoclonal: D13.14.4E), Stat1 (polyclonal), Phospho-Stat1 (Tyr701; monoclonal D4A7), , PKCα (polyclonal), Phospho-PKCα/β II (Thr638/641; polyclonal), PPAR-γ (monoclonal: 81B8), and the secondary antibody (goat anti-rabbit IgG; polyclonal) were purchased from Cell Signalling Technology, MA, USA. Anti-beta Actin antibody (HRP conjugated; mouse monoclonal AC-15) was purchased from Abcam, Cambridge, MA, USA. Cell pellets were treated with lysis buffer containing 0.5M HEPES (pH 7.5; Sigma), 1M NaCl (Sigma), 150mM MgCl2 (Sigma), 100mM EDTA (Sigma), 100mM EGTA (Sigma), 10% IGEPAL (Sigma), 10mM Sodium fluoride (Sigma), 1mM Sodium orthovanadate (Sigma) and 1% protease inhibitor cocktail (Sigma). Protein concentrations were determined by Bradford assay (Thermofisher Scientific) according to the manufacturer’s protocol. 15 μg of total protein were diluted in Laemmli buffer containing 2% SDS (Sigma), 4% Glycerol (Sigma), 0.05% Bromophenol Blue (Sigma), 50 mM Tris (Sigma; pH 6.8), 1mM phenyl methyl sulfonyl fluoride (Sigma) and 5% Beta-Mercaptoethanol (Sigma). Protein samples were loaded onto 10% SDS–PAGE (Sigma) gels, separated by electrophoresis and then transferred to nitrocellulose membranes using [Mini Trans-Blot® Cell](http://www.bio-rad.com/en-ie/product/mini-trans-blot-cell?ID=589ca8f7-5751-487a-a453-571ee8cc8b7e) (BioRad, CA, USA). Membranes were incubated in blocking buffer (5% BSA in TBS with 0.05% Tween-20; Sigma) for 1 hour at RT and then incubated overnight at 4^o^C with primary antibodies. The membranes were washed 3 times with 0.05% Tween-20 in TBS (Sigma) and incubated with a horseradish peroxidase- conjugated secondary antibody at 1:2000 for 1.5 hours then washed a further 3 times. Finally, the membranes were developed by electrochemical luminescence by addition of ECL Plus Substrate (GE Healthcare Life Sciences, Pittsburgh, PA, USA). After suitable development times, bands were imaged using chemiluminescence reader (Uvitec Essential V6, Cambridge, UK).

**RNA isolation by TRIzol**

Cells were initially suspended in TRIzol reagent (Sigma) and stored at –80^o^C until use. Samples were then thawed and chloroform (Sigma) was added at 0.2 ml/1 ml TRIzol. Samples were mixed gently by inversion then allowed to stand at room temperature for 10 minutes followed by centrifugation at 12,000xg for 15 minutes at 4^o^C. The RNA-containing clear upper aqueous phase was transferred to 1.5ml microtubes (Sarstedt) and RNA was precipitated by adding isopropanol (Sigma) at 0.75 ml/1 ml Trizol, mixing vigorously and storing overnight at -20^o^C. The samples were then centrifuged at 12,000xg for 10 minutes at 4^o^C, the isopropanol was discarded and the RNA pellets were washed by vortexing in 75% ethanol (Sigma) followed by centrifugation at 12,000xg for 15 minutes at 4^o^C. Finally, the ethanol was discarded and the RNA pellets air dried for 10 minutes prior to re-suspending in the appropriate volume of RNase-free water (Qiagen) and warming up tubes for 10-15 minutes at 50^0^C for complete dissolving.

**Preparation and initial culture of human peripheral blood mononuclear**

Samples of anticoagulated blood were collected from healthy adult volunteers by informed consent according to a protocol approved by the Research Ethics Committee of the National University of Ireland Galway (Ref:14/MAR/01). Peripheral blood mononuclear cells were separated by density gradient centrifugation using ficoll-hypaque solution (GE Healthcare) as previously described ^1^ and cultured in a T175 flask (Sarstedt) in macrophage medium consisting of RPMI-1640 (Gibco) containing 10% heat-inactivated human AB serum (Sigma), 1% penicillin/streptomycin (Gibco) and 1% L-Glutamine (Gibco). Floating cells were discarded the next day and adherent cells were washed with ice cold PBS (Gibco) then lifted by adding 0.25% Trypsin-EDTA (Gibco) to the flask for 3 minutes, counted using a hematocytometer.

**References**

1. Naicker SD, Cormican S, Griffin TP, et al. Chronic kidney disease severity is associated with selective expansion of a distinctive intermediate monocyte subpopulation. Front Immunol 2018;9.
