# Supplementary figures and images for: Human mesenchymal stromal cells broadly modulate high glucose-induced inflammatory responses of renal proximal tubular cell monolayers
Source: Stem Cell Res Ther. 2019 Nov 19;10:329. doi: 10.1186/s13287-019-1424-5 (PMC6862760; doi:10.1186/s13287-019-1424-5)

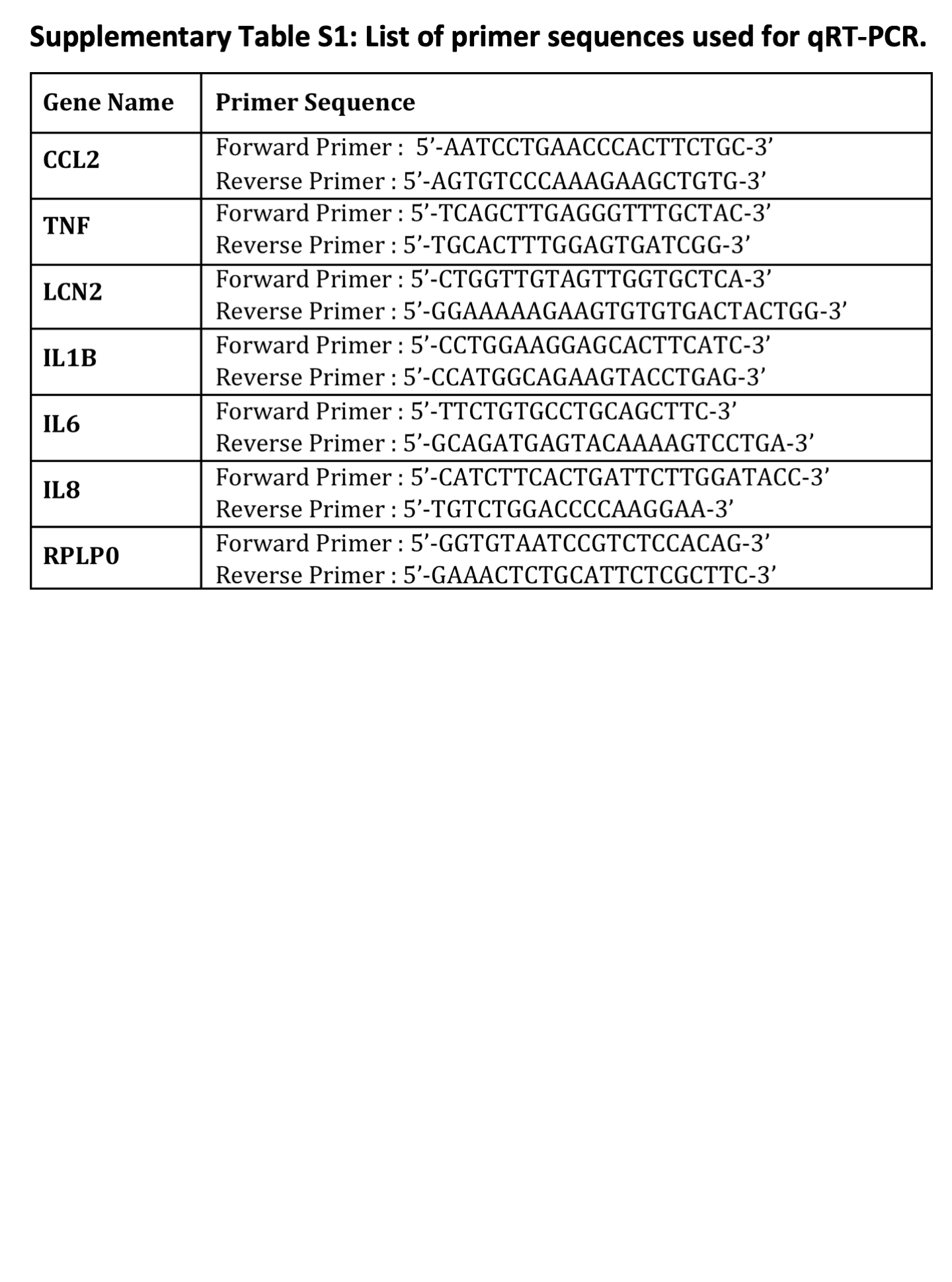

Supplement: Supplementary file 2 — Additional file 2: Table S1. List of primer sequences used for qRT-PCR. [file 13287_2019_1424_MOESM2_ESM.tiff]

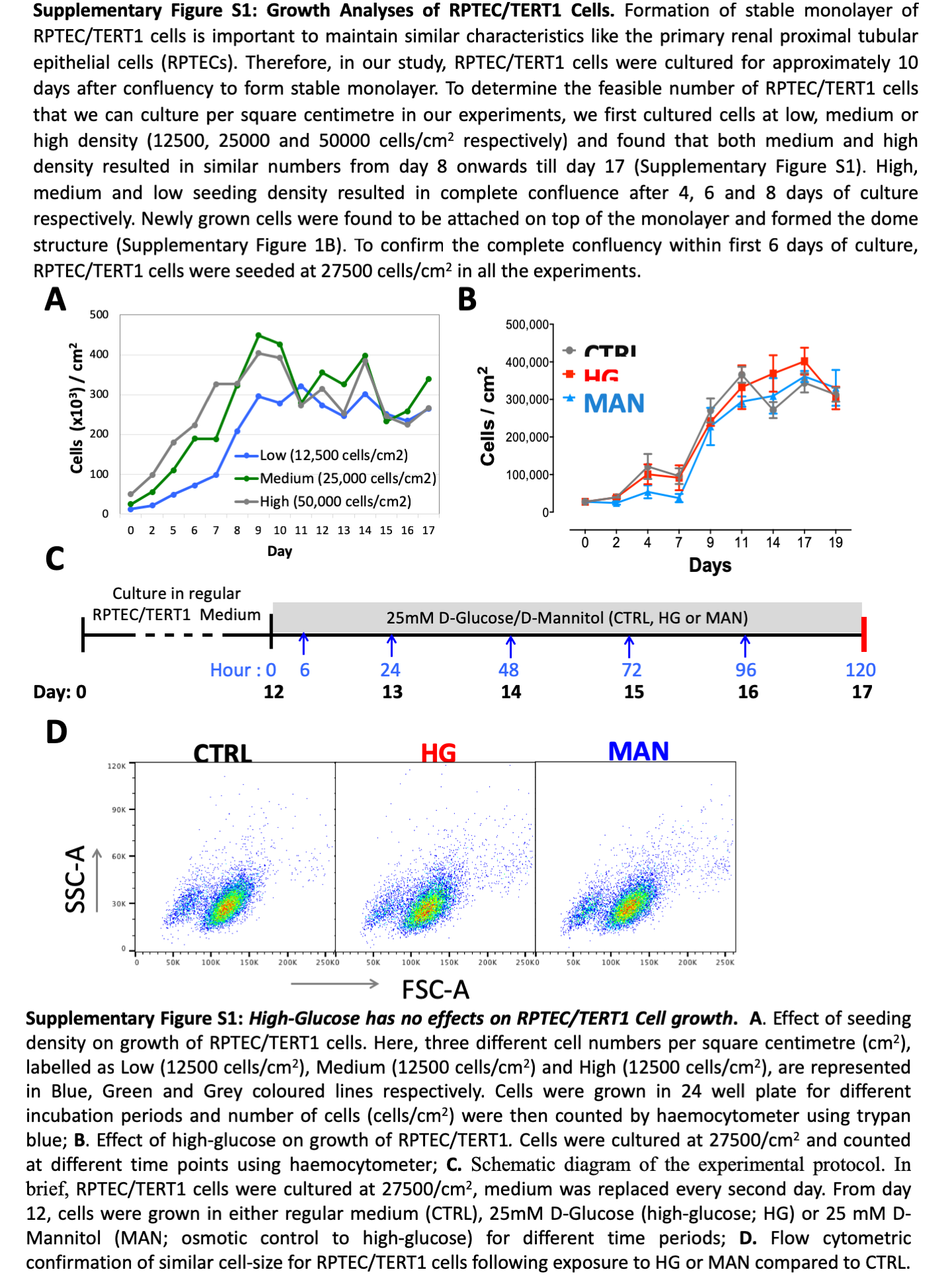

Supplement: Supplementary file 3 — Additional file 3: Figure S1. Effect of High D-Glucose on RPTEC/TERT1 Cells. A. Effect of seeding density on growth of RPTEC-TERT-1 cells. Here, three different cell numbers per square centimeter (cm2), labelled as Low (12500 cells/cm2), Medium (12500 cells/cm2) and High (12500 cells/cm2), are represented as Blue, Green and Grey coloured lines respectively. Cells grown in 24 well plate for different time points and number of cells counted by haemocytometer using trypan blue and represented as cells/cm2. B. Effect of high glucose on growth of RPTEC-TERT-1. Cells were seeded at 27500/cm2. and counted at different time points using haemocytometer. C. Downstream experimental plan. RPTEC-TERT-1 cells cultured at 27500/cm2, media replaced every second day. From day 12, 25 mM of D-Glucose or D-Mannitol (osmotic control to glucose) was administered for different time periods Effect of high Glucose on RPTECs for downstream comparisons of glucose versus controls: formation of stable monolayer by microscope; D. Similar cell size confirmed by flow cytometry. [file 13287_2019_1424_MOESM3_ESM.tiff]

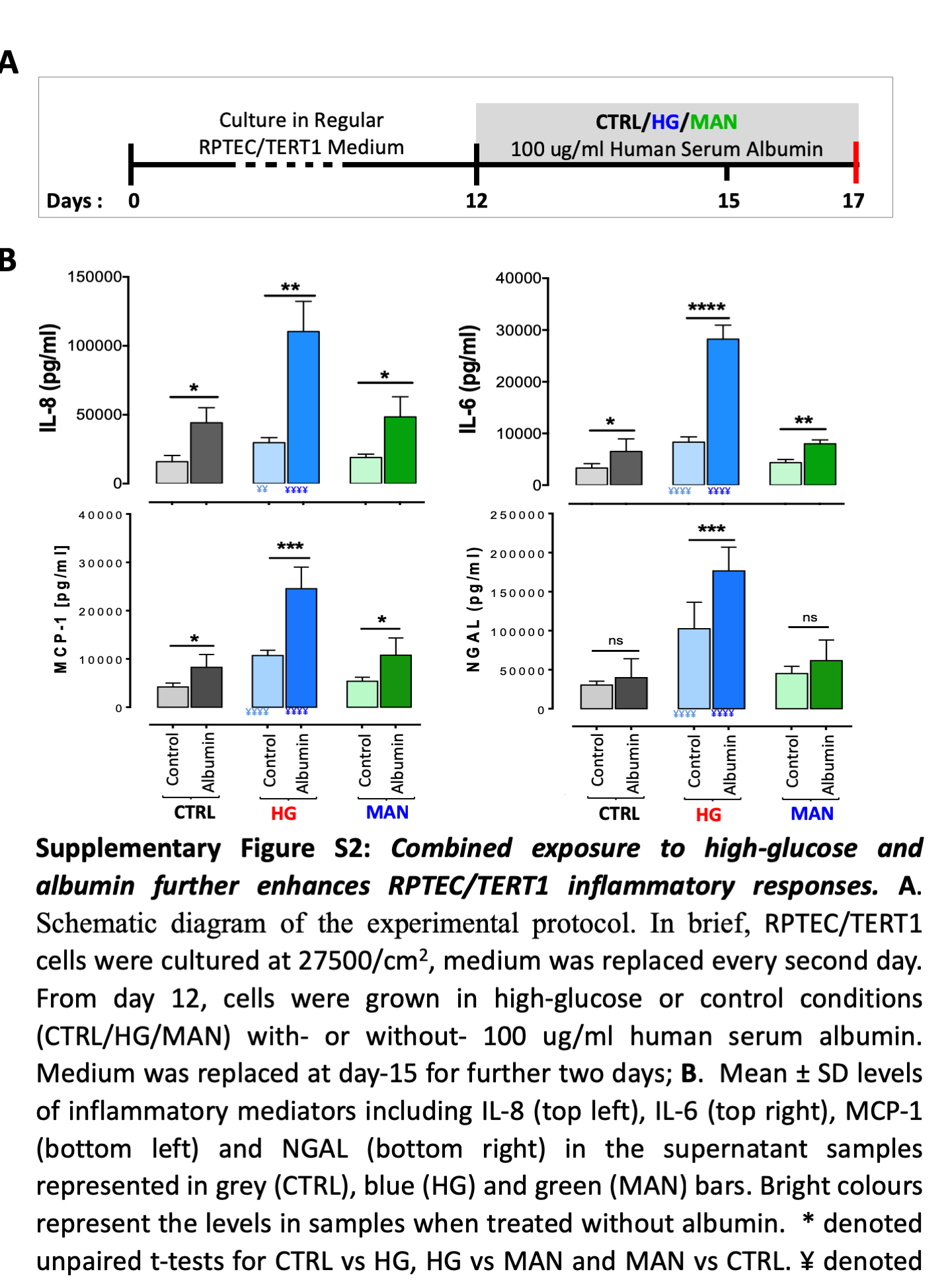

Supplement: Supplementary file 4 — Additional file 4: Figure S2. Combined effect of high glucose and albumin on RPTEC/TERT1 inflammatory responses. A. Schematic diagram of the experimental protocol. In brief, RPTEC-TERT-1 cells cultured at 27500/cm2, medium was replaced every second day. From day 12, cells were grown in high-glucose or control conditions (CTRL/HG/MAN) with or without 100 μg/ml human serum albumin. Mediium was replaced at day 15 for a further two days. B. Mean ± SD levels of inflammatory mediators including IL-8 (top left), IL-6 (top right), MCP-1 (bottom left) and NGAL (bottom right) in the supernatants are represented in grey (CTRL), blue (HG) and green (MAN) bars. Bright colours represent the levels in samples when treated without albumin. * denoted unpaired t-tests for CTRL vs HG, HG vs MAN, MAN vs CTRL. ¥ denoted ANOVA to analyse differences between CTRL, HG and MAN. ****/¥¥¥¥ p <0.0001, ***/¥¥¥ p <0.001, **/¥¥ p <0.01, */¥ p <0.05. [file 13287_2019_1424_MOESM4_ESM.tiff]

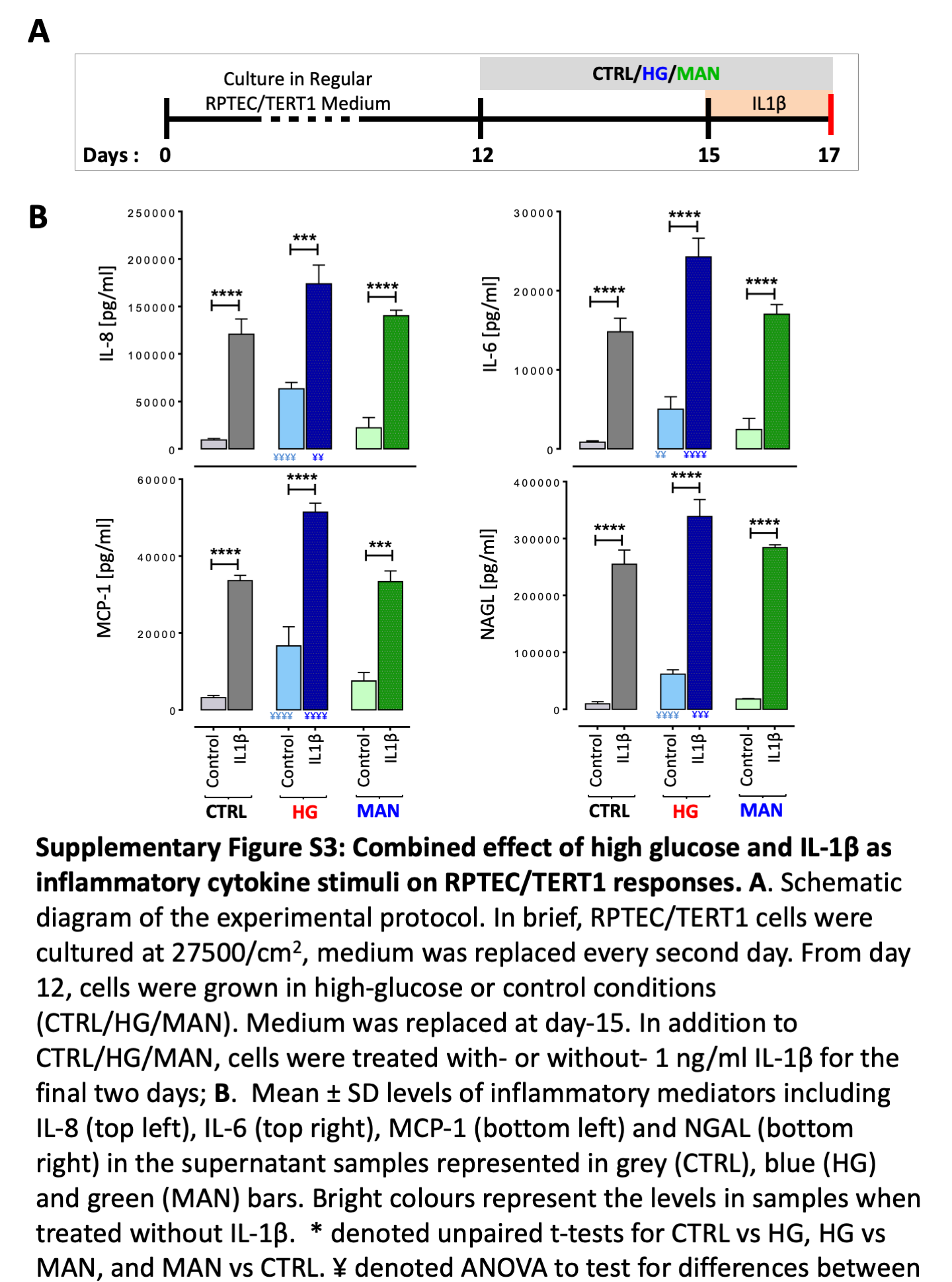

Supplement: Supplementary file 5 — Additional file 5: Figure S3. Combined effect of high glucose and IL-1β as inflammatory cytokine stimuli on RPTEC/TERT1 responses. A. Schematic diagram of the experimental protocol. In brief, RPTEC/TERT1 cells were cultured at 27500/cm2, medium was replaced every second day. From day 12, cells were grown in high-glucose or control conditions (CTRL/HG/MAN). Medium was replaced at day-15. In addition to CTRL/HG/MAN, cells were treated with- or without- 1 ng/ml IL-1β for the final two days; B. Mean ± SD levels of inflammatory mediators including IL-8 (top left), IL-6 (top right), MCP-1 (bottom left) and NGAL (bottom right) in the supernatant samples represented in grey (CTRL), blue (HG) and green (MAN) bars. Bright colours represent the levels in samples when treated without IL-1β. * denoted unpaired t-tests for CTRL vs HG, HG vs MAN, and MAN vs CTRL. ¥ denoted ANOVA to test for differences between CTRL, HG and MAN. ****/¥¥¥¥ p <0.0001, ***/¥¥¥ p <0.001, **/¥¥ p <0.01, */¥ p <0.05. [file 13287_2019_1424_MOESM5_ESM.tiff]

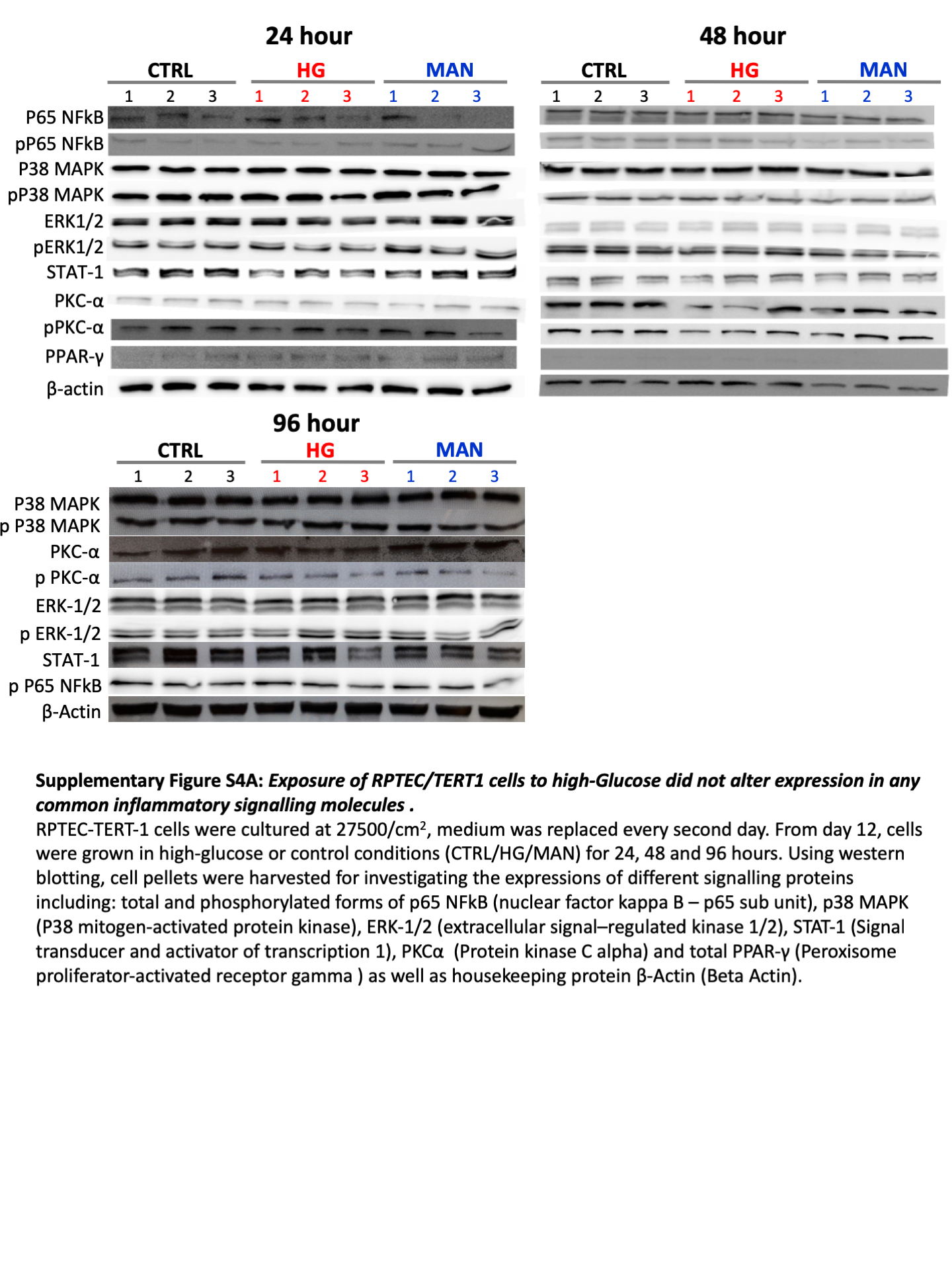

Supplement: Supplementary file 6 — Additional file 6: Figure S4. A: Exposure of RPTEC/TERT1 cells to high-Glucose did not alter expression in any common inflammatory signalling molecules. RPTEC-TERT-1 cells were cultured at 27500/cm2, medium was replaced every second day. From day 12, cells were grown in high-glucose or control conditions (CTRL/HG/MAN) for 24, 48 and 96 hours. Using western blotting, cell pellets were harvested for investigating the expressions of different signalling proteins including: total and phosphorylated forms of p65 NFkB (nuclear factor kappa B – p65 sub unit), p38 MAPK (P38 mitogen-activated protein kinase), ERK-1/2 (extracellular signal–regulated kinase 1/2), STAT-1 (Signal transducer and activator of transcription 1), PKCα (Protein kinase C alpha) and total PPAR-γ (Peroxisome proliferator-activated receptor gamma ) as well as housekeeping protein β-Actin (Beta Actin). [file 13287_2019_1424_MOESM6_ESM.tiff]

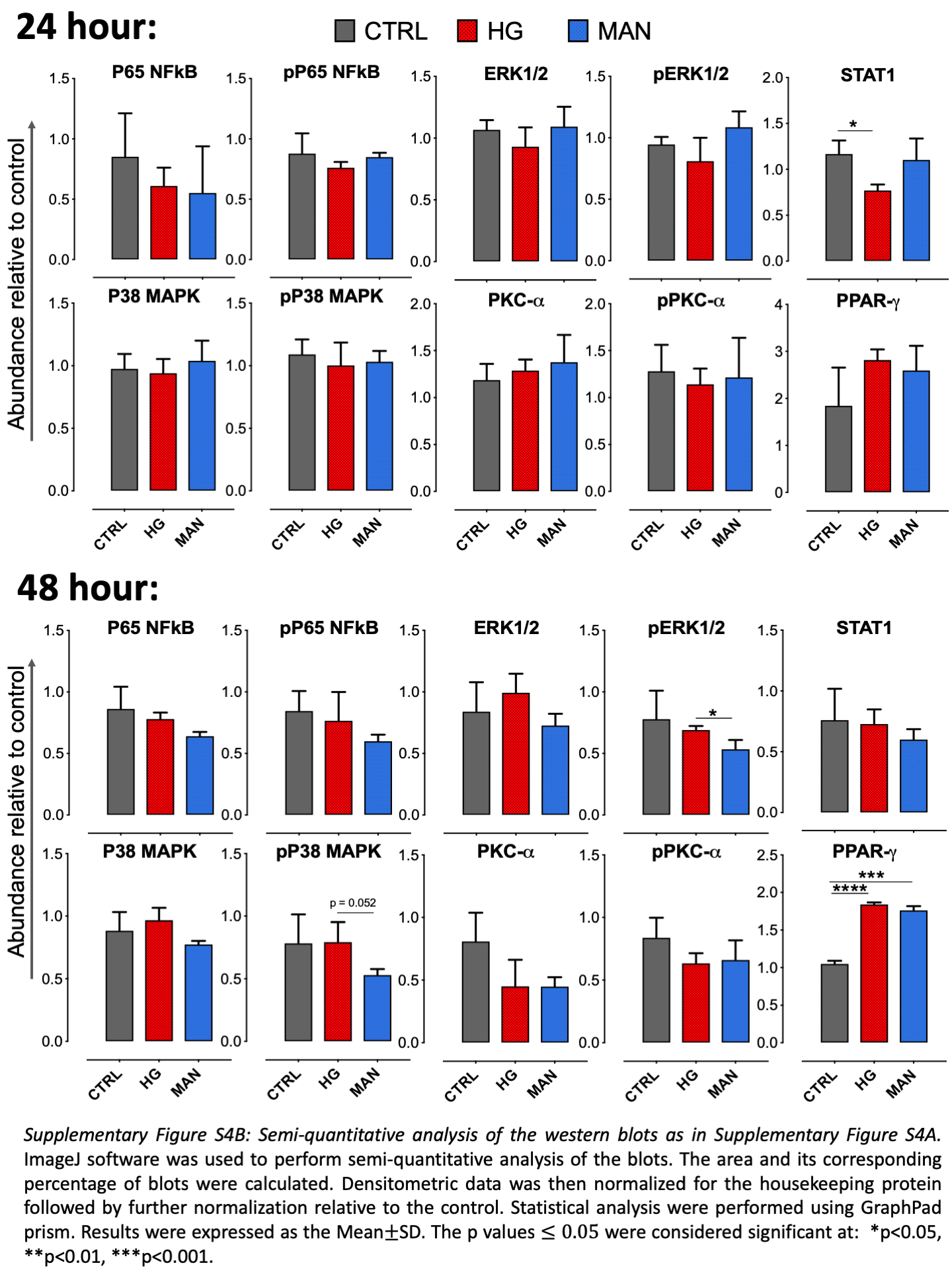

Supplement: Supplementary file 7 — Additional file 7: Figure S4. B: Semi-quantitative analyses of the western blots as in Figure S4A. ImageJ software was used to perform semi-quantitative analysis of the blots. The area and its corresponding percentage of blots were calculated. Densitometric data were then normalized for the housekeeping protein followed by further normalization relative to the control. Statistical analyses were performed using GraphPad prism. Results were expressed as the Mean±SD for three technical replicates per condition. p values ≤0.05 were considered significant at: *p<0.05, **p<0.01, ***p<0.001. [file 13287_2019_1424_MOESM7_ESM.tiff]

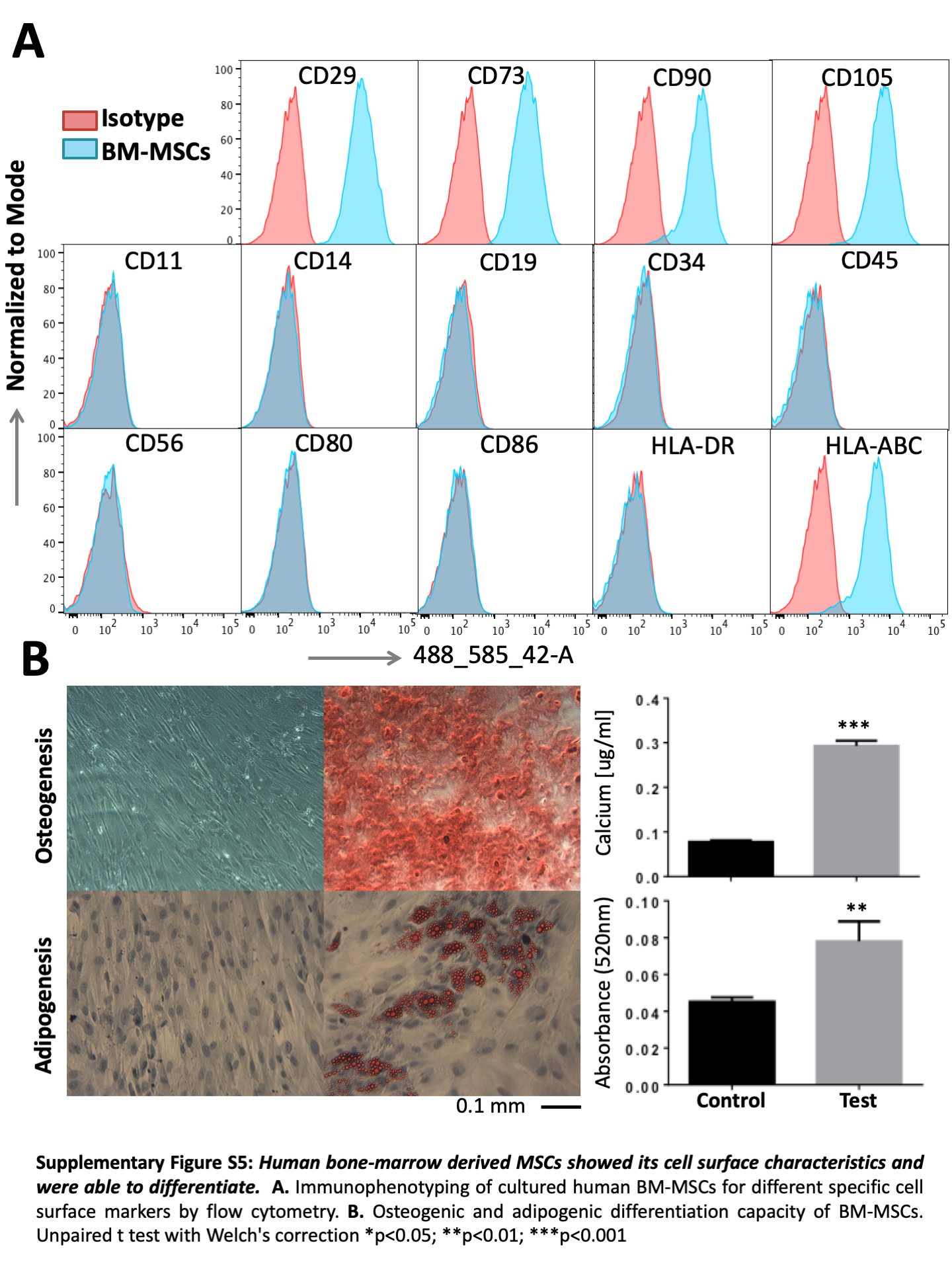

Supplement: Supplementary file 8 — Additional file 8: Figure S5. Human bone-marrow derived MSCs showed its cell surface characteristics and were able to differentiate. A. Immunophenotyping of cultured human BM-MSCs for different specific cell surface markers by flow cytometry. B. Osteogenic and adipogenic differentiation capacity of BM-MSCs. Unpaired t test with Welch's correction *p<0.05; **p<0.01; ***p<0.001. [file 13287_2019_1424_MOESM8_ESM.tiff]

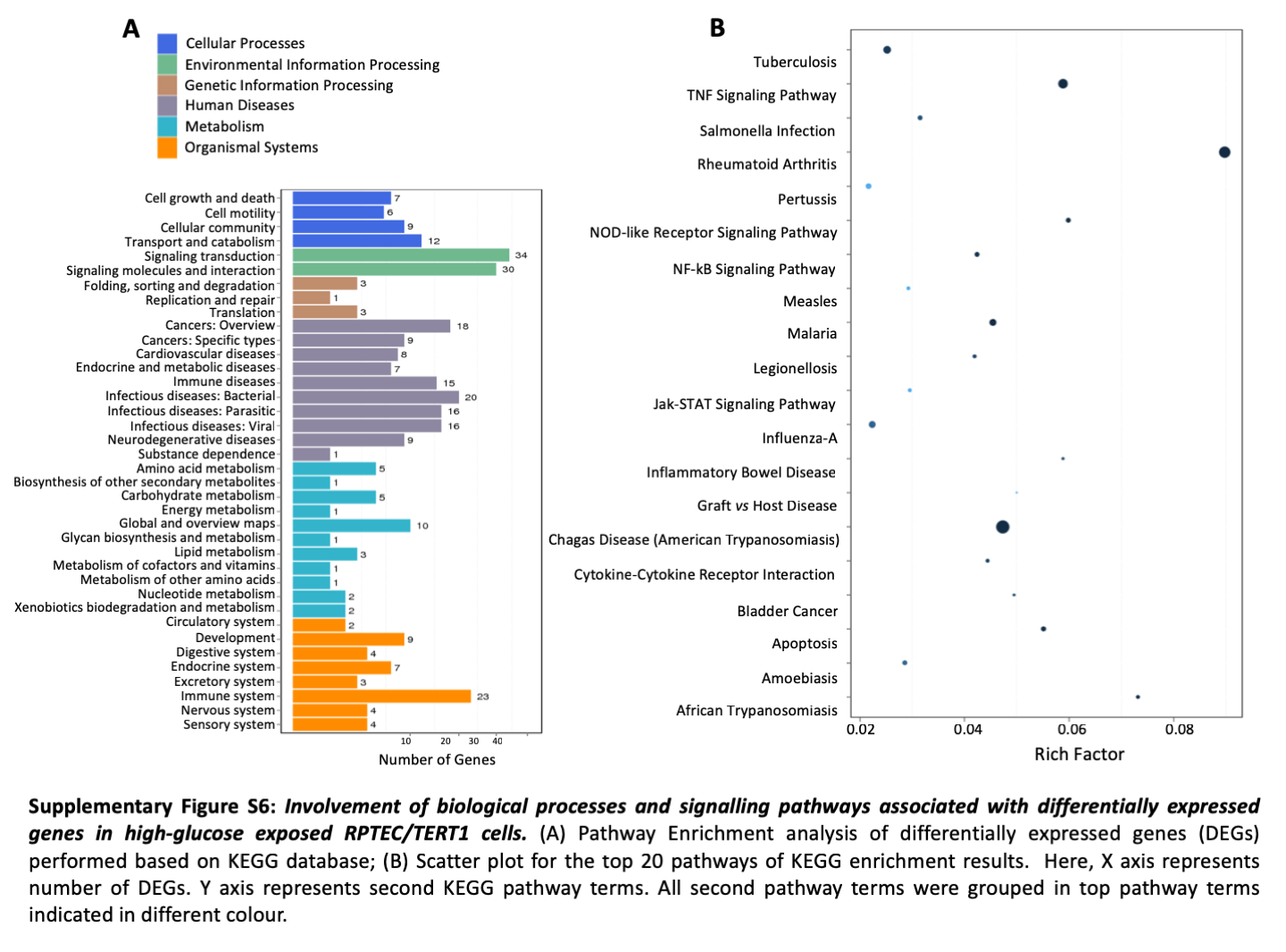

Supplement: Supplementary file 12 — Additional file 12: Figure S6. Involvement of biological processes and signalling pathways associated with differentially expressed genes in high-glucose exposed RPTEC/TERT1 cells. (A) Pathway Enrichment analysis of differentially expressed genes (DEGs) performed based on KEGG database; (B) Scatter plot for the top 20 pathways of KEGG enrichment results. Here, X axis represents number of DEGs. Y axis represents second KEGG pathway terms. All second pathway terms were grouped in top pathway terms indicated in different colour. [file 13287_2019_1424_MOESM12_ESM.tiff]

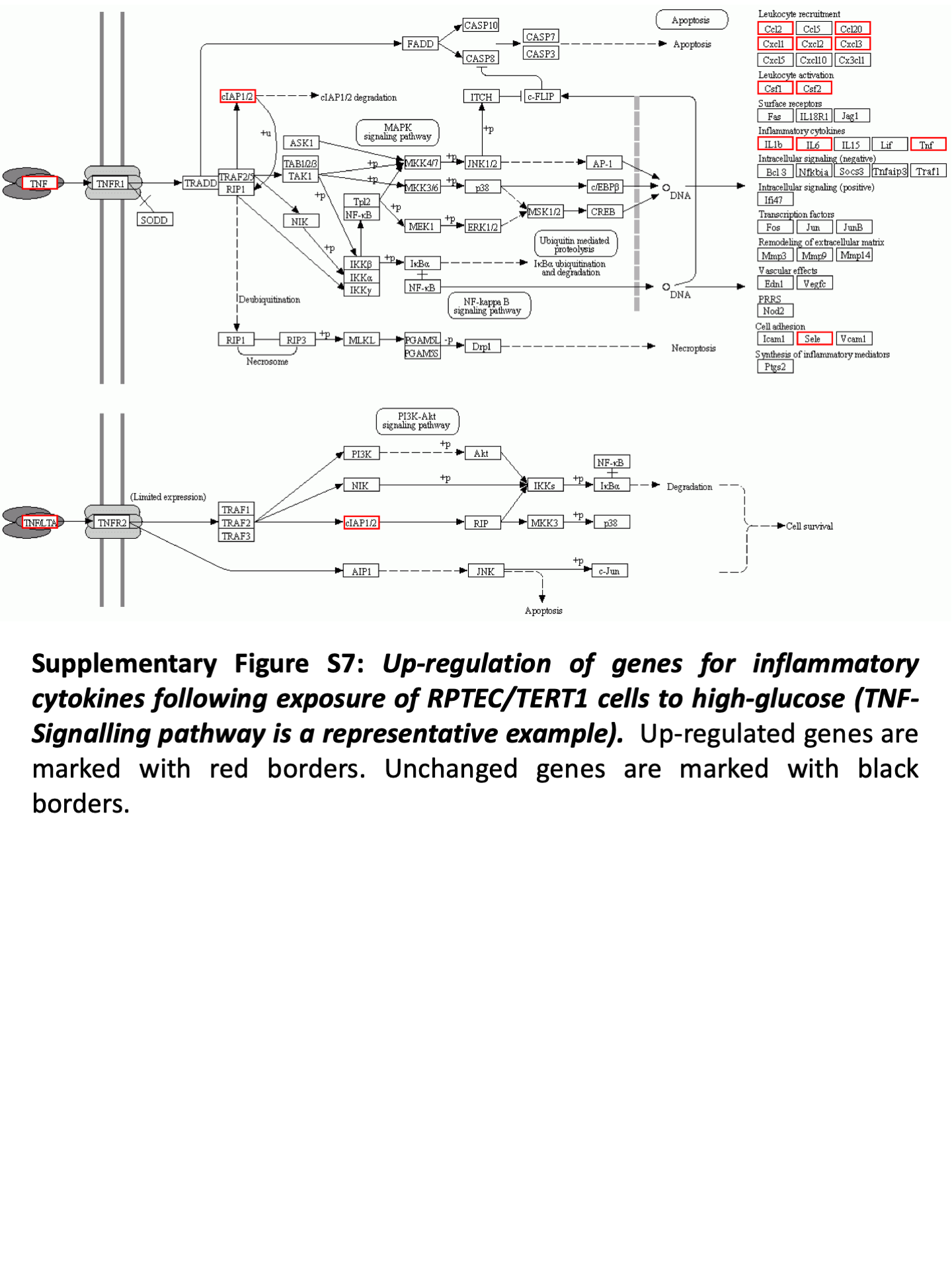

Supplement: Supplementary file 13 — Additional file 13: Figure S7. Up-regulation of genes for inflammatory cytokines following exposure of RPTEC/TERT1 cells to high-glucose (TNF-Signalling pathway is a representative example). Up-regulated genes are marked with red borders. Unchanged genes are marked with black borders. [file 13287_2019_1424_MOESM13_ESM.tiff]

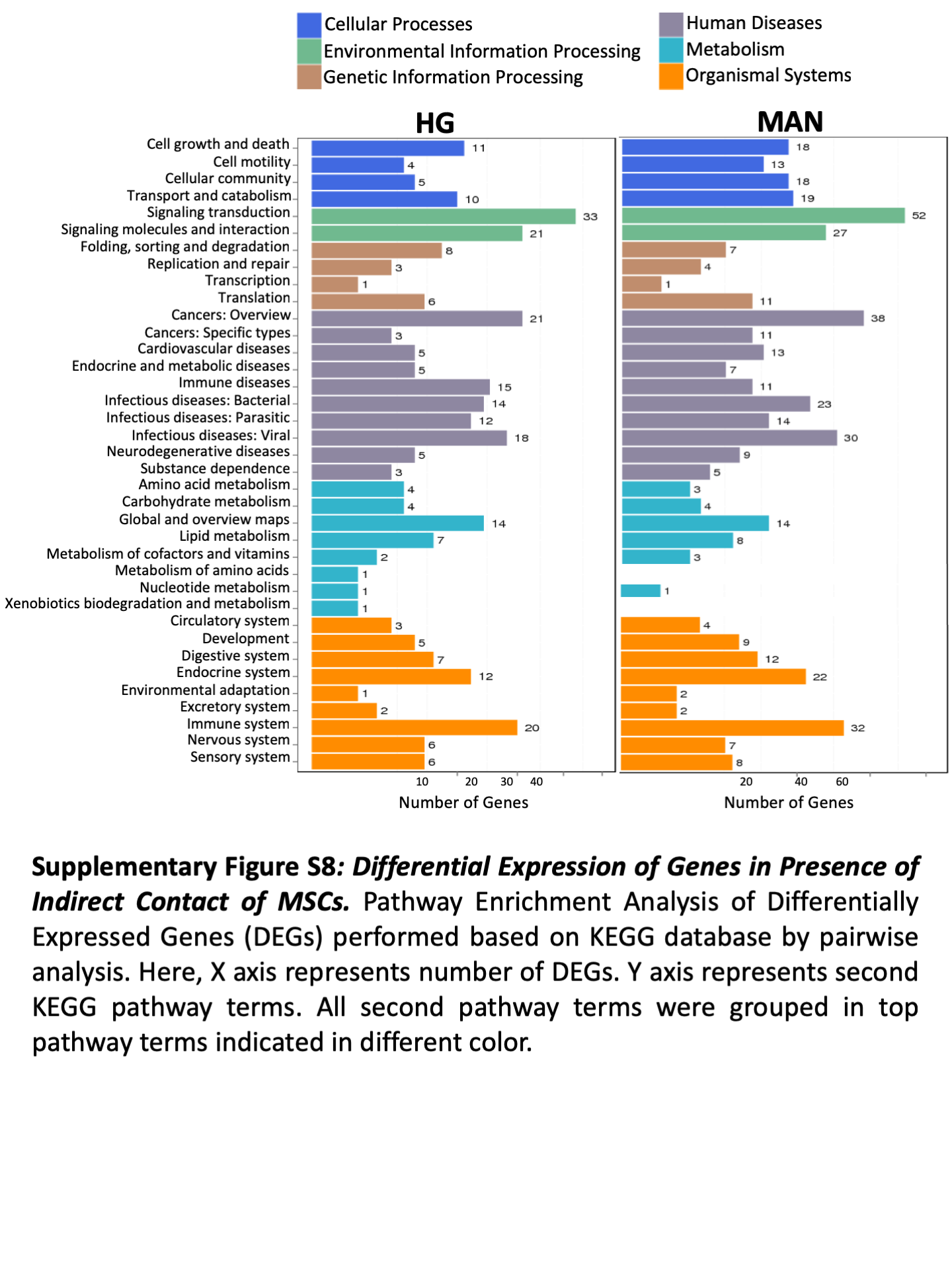

Supplement: Supplementary file 16 — Additional file 16: Figure S8. Differential Expression of Genes in Presence of Indirect Contact of MSCs. Pathway Enrichment Analysis of Differentially Expressed Genes (DEGs) performed based on KEGG database by pairwise analysis. Here, X axis represents number of DEGs. Y axis represents second KEGG pathway terms. All second pathway terms were grouped in top pathway terms indicated in different colour. [file 13287_2019_1424_MOESM16_ESM.tiff]

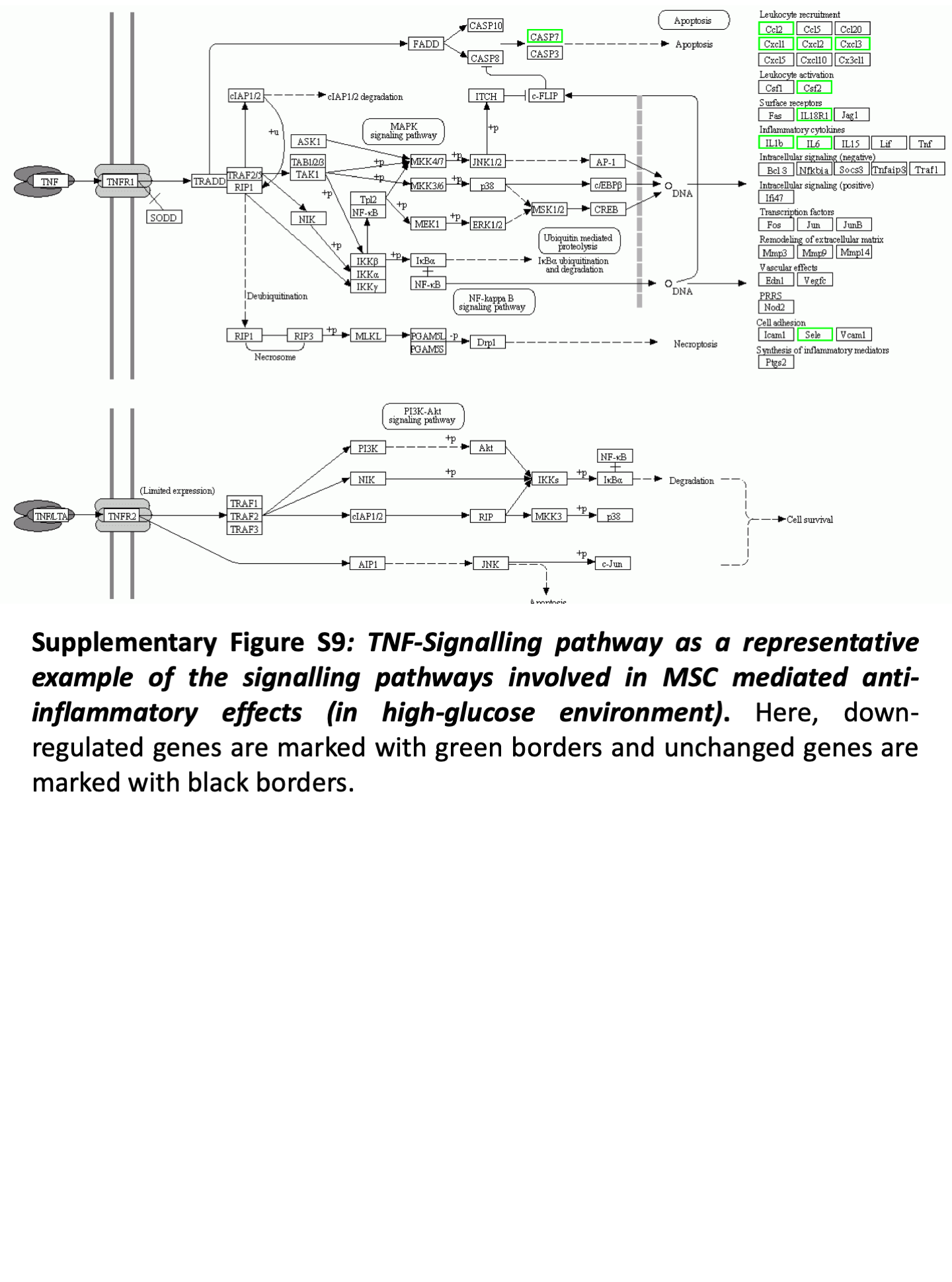

Supplement: Supplementary file 17 — Additional file 17: Figure S9. TNF-Signalling pathway as a representative example of the signalling pathways involved in MSC mediated anti-inflammatory effects (in high-glucose environment). Here, down-regulated genes are marked with green borders and unchanged genes are marked with black borders. [file 13287_2019_1424_MOESM17_ESM.tiff]
