# Supplementary material for: Human mesenchymal stromal cells broadly modulate high glucose-induced inflammatory responses of renal proximal tubular cell monolayers
Source: Stem Cell Res Ther. 2019 Nov 19;10:329. doi: 10.1186/s13287-019-1424-5 (PMC6862760; doi:10.1186/s13287-019-1424-5)
Supplement: Supplementary file 9 — Additional file 9: Table S2. List of DEGs with significant Fold Changes in High-Glucose vs Mannitol. [file 13287_2019_1424_MOESM9_ESM.docx]

| **Supplementary Table S2: List of DEGs with significant Fold Changes in High-Glucose vs Mannitol** | | | |
| --- | --- | --- | --- |
|  | Upregulated by HG |  |  |
|  | Downregulated by HG |  |  |
|  |  |  |  |
| **Gene Symbol** | **Gene Description** | **Fold Changes in HG** | **P Value** |
| PRAMEF17 | PRAME family member 17 | 20.33 | 0.047 |
| TEKT4 | tektin 4 | 16.00 | 0.013 |
| CECR7 | cat eye syndrome chromosome region, candidate 7 (non-protein coding) | 12.00 | 0.008 |
| IL18RAP | interleukin 18 receptor accessory protein | 12.00 | 0.008 |
| CACNA1S | calcium channel, voltage-dependent, L type, alpha 1S subunit | 11.00 | 0.010 |
| SCN1B | sodium channel, voltage-gated, type I, beta subunit | 9.14 | 0.045 |
| FNDC8 | fibronectin type III domain containing 8 | 9.13 | 0.05 |
| SNORD76 | small nucleolar RNA, C/D box 76 | 8.55 | 0.049 |
| IGSF9B | immunoglobulin superfamily, member 9B | 8.00 | 0.020 |
| SH3GL1P1 | SH3-domain GRB2-like 1 pseudogene 1 | 7.91 | 0.040 |
| FGF1 | fibroblast growth factor 1 (acidic) | 6.78 | 0.018 |
| CEP128 | centrosomal protein 128kDa | 6.55 | 0.011 |
| PIK3CG | phosphatidylinositol-4,5-bisphosphate 3-kinase, catalytic subunit gamma | 6.17 | 0.047 |
| TGM1 | transglutaminase 1 (K polypeptide epidermal type I, protein-glutamine-gamma-glutamyltransferase) | 6.08 | 0.002 |
| LHFPL3 | lipoma HMGIC fusion partner-like 3 | 5.83 | 0.041 |
| KRT25 | keratin 25 | 5.67 | 0.002 |
| AOC3 | amine oxidase, copper containing 3 (vascular adhesion protein 1) | 5.20 | 0.020 |
| RUNDC3A | RUN domain containing 3A | 5.17 | 0.025 |
| FAM179A | family with sequence similarity 179, member A | 4.80 | 0.049 |
| NPFF | neuropeptide FF-amide peptide precursor | 4.50 | 0.049 |
| CRYGS | crystallin, gamma S | 4.33 | 0.008 |
| ACSBG1 | acyl-CoA synthetase bubblegum family member 1 | 4.25 | 0.023 |
| COL16A1 | collagen, type XVI, alpha 1 | 3.41 | 0.028 |
| NR2E1 | nuclear receptor subfamily 2, group E, member 1 | 3.33 | 0.020 |
| SELL | selectin L | 3.31 | 0.037 |
| NUTM2G | NUT family member 2G | 3.25 | 0.035 |
| NEK10 | NIMA-related kinase 10 | 3.25 | 0.035 |
| PDE2A | phosphodiesterase 2A, cGMP-stimulated | 3.24 | 0.019 |
| C7orf63 | chromosome 7 open reading frame 63 | 3.13 | 0.008 |
| SNORD10 | small nucleolar RNA, C/D box 10 | 3.11 | 0.031 |
| CD160 | CD160 molecule | 3.11 | 0.019 |
| TNNC1 | troponin C type 1 (slow) | 3.11 | 0.003 |
| LINC00310 | long intergenic non-protein coding RNA 310 | 3.05 | 0.035 |
| GJB5 | gap junction protein, beta 5, 31.1kDa | 3.04 | 0.002 |
| CYP1B1 | cytochrome P450, family 1, subfamily B, polypeptide 1 | 2.96 | 0.018 |
| LOC283856 | uncharacterized LOC283856 | 2.88 | 0.034 |
| SLC25A18 | solute carrier family 25 (glutamate carrier), member 18 | 2.87 | 0.013 |
| LINC00634 | long intergenic non-protein coding RNA 634 | 2.84 | 0.015 |
| LOC100506071 | uncharacterized LOC100506071 | 2.76 | 0.026 |
| POF1B | premature ovarian failure, 1B | 2.71 | 0.020 |
| FGF11 | fibroblast growth factor 11 | 2.69 | 0.035 |
| SNORA40 | small nucleolar RNA, H/ACA box 40 | 2.68 | 0.016 |
| FAM198B | family with sequence similarity 198, member B | 2.67 | 0.038 |
| ESPL1 | extra spindle pole bodies homolog 1 (S. cerevisiae) | 2.65 | 0.004 |
| FLJ37035 | uncharacterized LOC399821 | 2.63 | 0.017 |
| CENPE | centromere protein E, 312kDa | 2.61 | 0.034 |
| ALDH1A3 | aldehyde dehydrogenase 1 family, member A3 | 2.60 | 0.002 |
| POLE2 | polymerase (DNA directed), epsilon 2, accessory subunit | 2.57 | 0.033 |
| IQCD | IQ motif containing D | 2.48 | 0.039 |
| TTC14 | tetratricopeptide repeat domain 14 | 2.39 | 0.044 |
| DNER | delta/notch-like EGF repeat containing | 2.37 | 0.038 |
| DAPK2 | death-associated protein kinase 2 | 2.33 | 0.015 |
| LY6G5B | lymphocyte antigen 6 complex, locus G5B | 2.33 | 0.006 |
| SLC9C1 | solute carrier family 9, subfamily C (Na+-transporting carboxylic acid decarboxylase), member 1 | 2.31 | 0.042 |
| ZNF432 | zinc finger protein 432 | 2.31 | 0.049 |
| TREML2 | triggering receptor expressed on myeloid cells-like 2 | 2.29 | 0.035 |
| ANKS1B | ankyrin repeat and sterile alpha motif domain containing 1B | 2.28 | 0.026 |
| ASB2 | ankyrin repeat and SOCS box containing 2 | 2.26 | 0.037 |
| USP2 | ubiquitin specific peptidase 2 | 2.23 | 0.043 |
| FBLN5 | fibulin 5 | 2.21 | 0.003 |
| TPTE2 | transmembrane phosphoinositide 3-phosphatase and tensin homolog 2 | 2.21 | 0.008 |
| GJB2 | gap junction protein, beta 2, 26kDa | 2.18 | 0.047 |
| TNFRSF11B | tumor necrosis factor receptor superfamily, member 11b | 2.17 | 0.006 |
| RFC3 | replication factor C (activator 1) 3, 38kDa | 2.17 | 0.013 |
| GCSAM | germinal center-associated, signaling and motility | 2.16 | 0.025 |
| KCND1 | potassium voltage-gated channel, Shal-related subfamily, member 1 | 2.15 | 0.049 |
| ALPK2 | alpha-kinase 2 | 2.15 | 0.011 |
| EFCAB10 | EF-hand calcium binding domain 10 | 2.14 | 0.003 |
| FLJ45079 | FLJ45079 protein | 2.14 | 0.015 |
| FBXW10 | F-box and WD repeat domain containing 10 | 2.13 | 0.035 |
| PRRT1 | proline-rich transmembrane protein 1 | 2.13 | 0.035 |
| AHRR | aryl-hydrocarbon receptor repressor | 2.10 | 0.041 |
| AGER | advanced glycosylation end product-specific receptor | 2.09 | 0.020 |
| ALX3 | ALX homeobox 3 | 2.08 | 0.044 |
| IL1B | interleukin 1, beta | 2.07 | 0.05 |
| GPC2 | glypican 2 | 2.06 | 0.042 |
| CENPQ | centromere protein Q | 2.04 | 0.040 |
| GALNT9 | UDP-N-acetyl-alpha-D-galactosamine:polypeptide N-acetylgalactosaminyltransferase 9 (GalNAc-T9) | 2.03 | 0.018 |
| LINC00909 | uncharacterized LOC400657 | 2.01 | 0.037 |
| TRIM34 | tripartite motif containing 34 | 2.01 | 0.043 |
| ZNF239 | zinc finger protein 239 | 2.00 | 0.030 |
| SNORD22 | small nucleolar RNA, C/D box 22 | 2.00 | 0.031 |
| EMX2 | empty spiracles homeobox 2 | 1.96 | 0.016 |
| SLC43A2 | solute carrier family 43, member 2 | 1.96 | 0.034 |
| LINC01239 | uncharacterized LOC441389 | 1.95 | 0.049 |
| CXCL5 | chemokine (C-X-C motif) ligand 5 | 1.95 | 0.029 |
| MTBP | Mdm2, transformed 3T3 cell double minute 2, p53 binding protein (mouse) binding protein, 104kDa | 1.94 | 0.016 |
| PAX6 | paired box 6 | 1.93 | 0.014 |
| DISP2 | dispatched homolog 2 (Drosophila) | 1.91 | 0.004 |
| NOL8 | nucleolar protein 8 | 1.91 | 0.048 |
| LOC284865 | uncharacterized LOC284865 | 1.89 | 0.021 |
| DCLRE1A | DNA cross-link repair 1A | 1.89 | 0.012 |
| SCRN3 | secernin 3 | 1.86 | 0.045 |
| MBTD1 | mbt domain containing 1 | 1.86 | 0.008 |
| MATN2 | matrilin 2 | 1.86 | 0.033 |
| NEURL3 | neuralized homolog 3 (Drosophila) pseudogene | 1.85 | 0.011 |
| WDR4 | WD repeat domain 4 | 1.83 | 0.010 |
| ULK4 | unc-51-like kinase 4 (C. elegans) | 1.83 | 0.011 |
| BTN2A3P | butyrophilin, subfamily 2, member A3, pseudogene | 1.83 | 0.035 |
| CDCA2 | cell division cycle associated 2 | 1.83 | 0.025 |
| SLCO2B1 | solute carrier organic anion transporter family, member 2B1 | 1.81 | 0.004 |
| CDC14A | cell division cycle 14A | 1.80 | 0.027 |
| EHHADH-AS1 | EHHADH antisense RNA 1 | 1.80 | 0.047 |
| ASF1A | ASF1 anti-silencing function 1 homolog A (S. cerevisiae) | 1.80 | 0.009 |
| MBLAC2 | metallo-beta-lactamase domain containing 2 | 1.78 | 0.012 |
| ZNF30 | zinc finger protein 30 | 1.78 | 0.001 |
| DPF3 | D4, zinc and double PHD fingers, family 3 | 1.77 | 0.013 |
| LNP1 | leukemia NUP98 fusion partner 1 | 1.74 | 0.003 |
| ZNF300 | zinc finger protein 300 | 1.74 | 0.044 |
| ZPLD1 | zona pellucida-like domain containing 1 | 1.73 | 0.012 |
| MALT1 | mucosa associated lymphoid tissue lymphoma translocation gene 1 | 1.71 | 0.006 |
| HIST2H2BC | histone cluster 2, H2bc (pseudogene) | 1.71 | 0.004 |
| SLC6A6 | solute carrier family 6 (neurotransmitter transporter, taurine), member 6 | 1.70 | 0.003 |
| ZNF75A | zinc finger protein 75a | 1.70 | 0.010 |
| RECK | reversion-inducing-cysteine-rich protein with kazal motifs | 1.69 | 0.022 |
| KCTD12 | potassium channel tetramerisation domain containing 12 | 1.68 | 0.043 |
| ADAMTSL4 | ADAMTS-like 4 | 1.68 | 0.05 |
| SLC7A5 | solute carrier family 7 (amino acid transporter light chain, L system), member 5 | 1.68 | 0.017 |
| TMEM169 | transmembrane protein 169 | 1.68 | 0.008 |
| DEPDC5 | DEP domain containing 5 | 1.67 | 0.048 |
| TLR6 | toll-like receptor 6 | 1.66 | 0.028 |
| CCZ1B | CCZ1 vacuolar protein trafficking and biogenesis associated homolog B (S. cerevisiae) | 1.66 | 0.048 |
| IZUMO4 | IZUMO family member 4 | 1.65 | 0.05 |
| NALCN-AS1 | NALCN antisense RNA 1 | 1.65 | 0.030 |
| LINC00888 | uncharacterized LOC100505687 | 1.65 | 0.026 |
| TGFBI | transforming growth factor, beta-induced, 68kDa | 1.65 | 0.049 |
| MTFR2 | mitochondrial fission regulator 2 | 1.65 | 0.038 |
| LOC100288637 | OTU domain containing 7A pseudogene | 1.65 | 0.044 |
| CCDC146 | coiled-coil domain containing 146 | 1.64 | 0.015 |
| NOL11 | nucleolar protein 11 | 1.64 | 0.021 |
| RABL2B | RAB, member of RAS oncogene family-like 2B | 1.64 | 0.019 |
| C9orf84 | chromosome 9 open reading frame 84 | 1.64 | 0.020 |
| ENOX2 | ecto-NOX disulfide-thiol exchanger 2 | 1.64 | 0.015 |
| SH3BP5-AS1 | SH3BP5 antisense RNA 1 | 1.63 | 0.0008 |
| FBXO9 | F-box protein 9 | 1.63 | 0.021 |
| ODC1 | ornithine decarboxylase 1 | 1.63 | 0.045 |
| FUT8 | fucosyltransferase 8 (alpha (1,6) fucosyltransferase) | 1.63 | 0.021 |
| ACR | acrosin | 1.63 | 0.038 |
| LAMA3 | laminin, alpha 3 | 1.62 | 0.042 |
| KLHL20 | kelch-like family member 20 | 1.62 | 0.046 |
| DNA2 | DNA replication helicase 2 homolog (yeast) | 1.62 | 0.017 |
| LTB | lymphotoxin beta (TNF superfamily, member 3) | 1.62 | 0.027 |
| CNIH3 | cornichon homolog 3 (Drosophila) | 1.61 | 0.035 |
| FERMT1 | fermitin family member 1 | 1.61 | 0.037 |
| ARHGEF10 | Rho guanine nucleotide exchange factor (GEF) 10 | 1.61 | 0.022 |
| RNF219-AS1 | RNF219 antisense RNA 1 | 1.61 | 0.023 |
| AZI2 | 5-azacytidine induced 2 | 1.60 | 0.015 |
| CEP162 | KIAA1009 | 1.60 | 0.049 |
| DOCK4 | dedicator of cytokinesis 4 | 1.59 | 0.007 |
| NUP37 | nucleoporin 37kDa | 1.59 | 0.023 |
| LOC100132352 | FSHD region gene 1 pseudogene | 1.59 | 0.046 |
| RBM4B | RNA binding motif protein 4B | 1.59 | 0.026 |
| KCTD4 | potassium channel tetramerisation domain containing 4 | 1.59 | 0.0004 |
| DYSF | dysferlin, limb girdle muscular dystrophy 2B (autosomal recessive) | 1.59 | 0.024 |
| HMGA2 | high mobility group AT-hook 2 | 1.59 | 0.038 |
| FAM135A | family with sequence similarity 135, member A | 1.59 | 0.008 |
| ZNF34 | zinc finger protein 34 | 1.58 | 0.05 |
| LOH12CR1 | loss of heterozygosity, 12, chromosomal region 1 | 1.58 | 0.003 |
| SYP | synaptophysin | 1.58 | 0.032 |
| EFCAB7 | EF-hand calcium binding domain 7 | 1.57 | 0.038 |
| ADCK3 | aarF domain containing kinase 3 | 1.57 | 0.036 |
| GOLGA7B | golgin A7 family, member B | 1.56 | 0.028 |
| RCOR3 | REST corepressor 3 | 1.56 | 0.017 |
| AP4B1 | adaptor-related protein complex 4, beta 1 subunit | 1.56 | 0.009 |
| ITGAE | integrin, alpha E (antigen CD103, human mucosal lymphocyte antigen 1; alpha polypeptide) | 1.55 | 0.036 |
| ZNF18 | zinc finger protein 18 | 1.55 | 0.015 |
| KCNJ15 | potassium inwardly-rectifying channel, subfamily J, member 15 | 1.55 | 0.006 |
| MPP3 | membrane protein, palmitoylated 3 (MAGUK p55 subfamily member 3) | 1.55 | 0.033 |
| LOC613037 | nuclear pore complex interacting protein pseudogene | 1.55 | 0.049 |
| ANKRD26 | ankyrin repeat domain 26 | 1.54 | 0.011 |
| ZNHIT6 | zinc finger, HIT-type containing 6 | 1.54 | 0.023 |
| GPAT2 | glycerol-3-phosphate acyltransferase 2, mitochondrial | 1.54 | 0.017 |
| FREM2 | FRAS1 related extracellular matrix protein 2 | 1.53 | 0.002 |
| ENC1 | ectodermal-neural cortex 1 (with BTB domain) | 1.53 | 0.018 |
| PPAP2B | phosphatidic acid phosphatase type 2B | 1.53 | 0.044 |
| RFK | riboflavin kinase | 1.53 | 0.041 |
| BATF3 | basic leucine zipper transcription factor, ATF-like 3 | 1.53 | 0.027 |
| ALG1L | ALG1, chitobiosyldiphosphodolichol beta-mannosyltransferase-like | 1.53 | 0.004 |
| ALKBH1 | alkB, alkylation repair homolog 1 (E. coli) | 1.53 | 0.013 |
| FAM86C2P | family with sequence similarity 86, member A pseudogene | 1.53 | 0.037 |
| SLCO4C1 | solute carrier organic anion transporter family, member 4C1 | 1.53 | 0.001 |
| PPM1M | protein phosphatase, Mg2+/Mn2+ dependent, 1M | 1.52 | 0.030 |
| LSM5 | LSM5 homolog, U6 small nuclear RNA associated (S. cerevisiae) | 1.52 | 0.005 |
| DHX57 | DEAH (Asp-Glu-Ala-Asp/His) box polypeptide 57 | 1.52 | 0.026 |
| PDGFB | platelet-derived growth factor beta polypeptide | 1.52 | 0.002 |
| CDH6 | cadherin 6, type 2, K-cadherin (fetal kidney) | 1.52 | 0.020 |
| WDR12 | WD repeat domain 12 | 1.52 | 0.031 |
| CD58 | CD58 molecule | 1.52 | 0.002 |
| C9orf41 | chromosome 9 open reading frame 41 | 1.52 | 0.045 |
| TRAM2 | translocation associated membrane protein 2 | 1.52 | 0.008 |
| PIK3R1 | phosphoinositide-3-kinase, regulatory subunit 1 (alpha) | 1.51 | 0.024 |
| HHAT | hedgehog acyltransferase | 1.51 | 0.023 |
| CCDC175 | coiled-coil domain containing 175 | 1.51 | 0.042 |
| PDCD2L | programmed cell death 2-like | 1.51 | 0.007 |
| KANSL1-AS1 | KANSL1 antisense RNA 1 | 1.51 | 0.048 |
| ARHGAP22 | Rho GTPase activating protein 22 | 1.50 | 0.042 |
| APC2 | adenomatosis polyposis coli 2 | 1.50 | 0.023 |
| LOC728743 | zinc finger protein pseudogene | 1.50 | 0.035 |
| PRR15L | proline rich 15-like | 0.05 | 0.012 |
| FSCN2 | fascin homolog 2, actin-bundling protein, retinal (Strongylocentrotus purpuratus) | 0.09 | 0.038 |
| OR7E91P | olfactory receptor, family 7, subfamily E, member 91 pseudogene | 0.10 | 0.009 |
| PLA2G4F | phospholipase A2, group IVF | 0.11 | 0.035 |
| RENBP | renin binding protein | 0.11 | 0.028 |
| BBOX1 | butyrobetaine (gamma), 2-oxoglutarate dioxygenase (gamma-butyrobetaine hydroxylase) 1 | 0.12 | 0.0009 |
| LOC90246 | uncharacterized LOC90246 | 0.15 | 0.008 |
| NAPSA | napsin A aspartic peptidase | 0.16 | 0.004 |
| UBE2Q2P2 | ubiquitin-conjugating enzyme E2Q family member 2 pseudogene 2 | 0.16 | 0.046 |
| MYO7B | myosin VIIB | 0.16 | 0.002 |
| PLA1A | phospholipase A1 member A | 0.20 | 0.020 |
| NME9 | NME/NM23 family member 9 | 0.21 | 0.049 |
| GPNMB | glycoprotein (transmembrane) nmb | 0.21 | 0.010 |
| ADH4 | alcohol dehydrogenase 4 (class II), pi polypeptide | 0.22 | 0.029 |
| SPX | chromosome 12 open reading frame 39 | 0.24 | 0.032 |
| ACP5 | acid phosphatase 5, tartrate resistant | 0.24 | 0.009 |
| LINC00323 | long intergenic non-protein coding RNA 323 | 0.26 | 0.046 |
| C4orf47 | chromosome 4 open reading frame 47 | 0.28 | 0.013 |
| GPM6B | glycoprotein M6B | 0.28 | 0.039 |
| RORC | RAR-related orphan receptor C | 0.28 | 0.001 |
| SMARCD3 | SWI/SNF related, matrix associated, actin dependent regulator of chromatin, subfamily d, member 3 | 0.28 | 0.009 |
| SCARNA2 | small Cajal body-specific RNA 2 | 0.28 | 0.012 |
| ABI3BP | ABI family, member 3 (NESH) binding protein | 0.28 | 0.012 |
| MLXIPL | MLX interacting protein-like | 0.29 | 0.003 |
| HRASLS5 | HRAS-like suppressor family, member 5 | 0.29 | 0.034 |
| FXYD6 | FXYD domain containing ion transport regulator 6 | 0.29 | 0.007 |
| PCDHGA9 | protocadherin gamma subfamily A, 9 | 0.30 | 0.020 |
| HSD17B14 | hydroxysteroid (17-beta) dehydrogenase 14 | 0.30 | 0.002 |
| ADH6 | alcohol dehydrogenase 6 (class V) | 0.31 | 0.0002 |
| CLCA3P | chloride channel accessory 3, pseudogene | 0.31 | 0.034 |
| GPX3 | glutathione peroxidase 3 (plasma) | 0.32 | 0.018 |
| ATP6V1B1 | ATPase, H+ transporting, lysosomal 56/58kDa, V1 subunit B1 | 0.33 | 0.008 |
| NR1H4 | nuclear receptor subfamily 1, group H, member 4 | 0.34 | 0.032 |
| FAM131C | family with sequence similarity 131, member C | 0.34 | 0.002 |
| PANX2 | pannexin 2 | 0.34 | 0.028 |
| CCDC81 | coiled-coil domain containing 81 | 0.34 | 0.046 |
| PBX1 | pre-B-cell leukemia homeobox 1 | 0.35 | 0.039 |
| IFIT3 | interferon-induced protein with tetratricopeptide repeats 3 | 0.35 | 0.016 |
| BDKRB2 | bradykinin receptor B2 | 0.36 | 0.048 |
| STC1 | stanniocalcin 1 | 0.36 | 0.022 |
| RN7SL1 | RNA, 7SL, cytoplasmic 1 | 0.36 | 0.041 |
| LTF | lactotransferrin | 0.37 | 0.014 |
| NRAP | nebulin-related anchoring protein | 0.37 | 0.013 |
| TNNI3 | troponin I type 3 (cardiac) | 0.38 | 0.049 |
| IFIT1 | interferon-induced protein with tetratricopeptide repeats 1 | 0.38 | 0.009 |
| HABP2 | hyaluronan binding protein 2 | 0.38 | 0.008 |
| MAP2 | microtubule-associated protein 2 | 0.38 | 0.010 |
| SECTM1 | secreted and transmembrane 1 | 0.39 | 0.020 |
| PSCA | prostate stem cell antigen | 0.39 | 0.016 |
| KCNIP4 | Kv channel interacting protein 4 | 0.40 | 0.047 |
| CAPN14 | calpain 14 | 0.40 | 0.002 |
| NPR1 | natriuretic peptide receptor A/guanylate cyclase A (atrionatriuretic peptide receptor A) | 0.40 | 0.012 |
| NTRK2 | neurotrophic tyrosine kinase, receptor, type 2 | 0.40 | 0.029 |
| TTYH2 | tweety homolog 2 (Drosophila) | 0.41 | 0.014 |
| LOC100130705 | uncharacterized LOC100130705 | 0.41 | 0.035 |
| DEFB1 | defensin, beta 1 | 0.41 | 0.049 |
| HILPDA | hypoxia inducible lipid droplet-associated | 0.42 | 0.004 |
| RTBDN | retbindin | 0.42 | 0.014 |
| IL12A | interleukin 12A (natural killer cell stimulatory factor 1, cytotoxic lymphocyte maturation factor 1, p35) | 0.43 | 0.023 |
| CASP1 | caspase 1, apoptosis-related cysteine peptidase | 0.44 | 0.05 |
| HPSE | heparanase | 0.44 | 0.018 |
| CRYAB | crystallin, alpha B | 0.44 | 0.031 |
| TMEM91 | transmembrane protein 91 | 0.44 | 0.045 |
| FKBP9P1 | FK506 binding protein 9-like | 0.44 | 0.042 |
| KRTAP5-1 | keratin associated protein 5-1 | 0.44 | 0.0005 |
| CLDN14 | claudin 14 | 0.44 | 0.020 |
| ENO2 | enolase 2 (gamma, neuronal) | 0.44 | 0.001 |
| LOC100133669 | uncharacterized LOC100133669 | 0.44 | 0.006 |
| LAMA2 | laminin, alpha 2 | 0.45 | 0.004 |
| PRSS22 | protease, serine, 22 | 0.45 | 0.004 |
| CTSF | cathepsin F | 0.45 | 0.026 |
| FRAT1 | frequently rearranged in advanced T-cell lymphomas | 0.46 | 0.008 |
| ANKRD37 | ankyrin repeat domain 37 | 0.47 | 0.019 |
| NRN1L | neuritin 1-like | 0.47 | 0.028 |
| VTRNA2-1 | vault RNA 2-1 | 0.47 | 0.038 |
| WFDC2 | WAP four-disulfide core domain 2 | 0.47 | 0.013 |
| FOXJ1 | forkhead box J1 | 0.48 | 0.002 |
| PFKFB4 | 6-phosphofructo-2-kinase/fructose-2,6-biphosphatase 4 | 0.48 | 0.045 |
| EHD3 | EH-domain containing 3 | 0.48 | 0.025 |
| MIR210HG | MIR210 host gene (non-protein coding) | 0.48 | 0.022 |
| BIK | BCL2-interacting killer (apoptosis-inducing) | 0.49 | 0.037 |
| SEPP1 | selenoprotein P, plasma, 1 | 0.49 | 0.013 |
| PALM2-AKAP2 | PALM2-AKAP2 readthrough | 0.49 | 0.040 |
| ENDOD1 | endonuclease domain containing 1 | 0.49 | 0.018 |
| METAP1D | methionyl aminopeptidase type 1D (mitochondrial) | 0.49 | 0.011 |
| SLC3A1 | solute carrier family 3 (cystine, dibasic and neutral amino acid transporters, activator of cystine, dibasic and neutral amino acid transport), member 1 | 0.49 | 0.029 |
| SH3D21 | SH3 domain containing 21 | 0.50 | 0.010 |
| ANKRD22 | ankyrin repeat domain 22 | 0.50 | 0.038 |
| CFB | complement factor B | 0.51 | 0.020 |
| NDRG1 | N-myc downstream regulated 1 | 0.51 | 0.005 |
| CDIP1 | cell death-inducing p53 target 1 | 0.51 | 0.031 |
| CLU | clusterin | 0.52 | 0.001 |
| TMC4 | transmembrane channel-like 4 | 0.52 | 0.017 |
| SHC4 | SHC (Src homology 2 domain containing) family, member 4 | 0.52 | 0.036 |
| SCGB2A1 | secretoglobin, family 2A, member 1 | 0.53 | 0.006 |
| PTH1R | parathyroid hormone 1 receptor | 0.53 | 0.022 |
| MIR3064 | microRNA 3064 | 0.53 | 0.048 |
| ACSS1 | acyl-CoA synthetase short-chain family member 1 | 0.53 | 0.016 |
| CNTNAP1 | contactin associated protein 1 | 0.53 | 0.036 |
| CARD16 | caspase recruitment domain family, member 16 | 0.53 | 0.018 |
| LRAT | lecithin retinol acyltransferase (phosphatidylcholine--retinol O-acyltransferase) | 0.54 | 0.043 |
| BNIP3 | BCL2/adenovirus E1B 19kDa interacting protein 3 | 0.55 | 0.014 |
| RAP1GAP | RAP1 GTPase activating protein | 0.55 | 0.031 |
| IFITM1 | interferon induced transmembrane protein 1 | 0.55 | 0.003 |
| S100A1 | S100 calcium binding protein A1 | 0.55 | 0.019 |
| BCAM | basal cell adhesion molecule (Lutheran blood group) | 0.56 | 0.005 |
| NCKIPSD | NCK interacting protein with SH3 domain | 0.56 | 0.011 |
| CDH16 | cadherin 16, KSP-cadherin | 0.57 | 0.018 |
| ADAMTS9-AS2 | ADAMTS9 antisense RNA 2 | 0.57 | 0.011 |
| HGD | homogentisate 1,2-dioxygenase | 0.57 | 0.029 |
| MEGF11 | multiple EGF-like-domains 11 | 0.57 | 0.012 |
| IFITM10 | interferon induced transmembrane protein 10 | 0.57 | 0.035 |
| SLC2A12 | solute carrier family 2 (facilitated glucose transporter), member 12 | 0.57 | 0.031 |
| SLC6A13 | solute carrier family 6 (neurotransmitter transporter, GABA), member 13 | 0.58 | 0.041 |
| SEMA4A | sema domain, immunoglobulin domain (Ig), transmembrane domain (TM) and short cytoplasmic domain, (semaphorin) 4A | 0.58 | 0.023 |
| C4orf3 | chromosome 4 open reading frame 3 | 0.58 | 0.007 |
| CDK18 | cyclin-dependent kinase 18 | 0.58 | 0.012 |
| HK2 | hexokinase 2 | 0.58 | 0.006 |
| GBP2 | guanylate binding protein 2, interferon-inducible | 0.59 | 0.015 |
| MUC16 | mucin 16, cell surface associated | 0.59 | 0.009 |
| APOL1 | apolipoprotein L, 1 | 0.59 | 0.001 |
| GBE1 | glucan (1,4-alpha-), branching enzyme 1 | 0.59 | 0.009 |
| AGR2 | anterior gradient 2 homolog (Xenopus laevis) | 0.59 | 0.010 |
| OGDHL | oxoglutarate dehydrogenase-like | 0.59 | 0.028 |
| LINC00478 | long intergenic non-protein coding RNA 478 | 0.60 | 0.013 |
| RAP2B | RAP2B, member of RAS oncogene family | 0.60 | 0.024 |
| PBLD | phenazine biosynthesis-like protein domain containing | 0.60 | 0.026 |
| CFI | complement factor I | 0.60 | 0.032 |
| IRS1 | insulin receptor substrate 1 | 0.60 | 0.008 |
| ZIM3 | zinc finger, imprinted 3 | 0.61 | 0.037 |
| TGM5 | transglutaminase 5 | 0.61 | 0.016 |
| COL11A1 | collagen, type XI, alpha 1 | 0.61 | 0.049 |
| ST3GAL4 | ST3 beta-galactoside alpha-2,3-sialyltransferase 4 | 0.61 | 0.040 |
| GPLD1 | glycosylphosphatidylinositol specific phospholipase D1 | 0.61 | 0.041 |
| ZNF559 | zinc finger protein 559 | 0.61 | 0.027 |
| NEURL1 | neuralized homolog (Drosophila) | 0.61 | 0.029 |
| IFITM2 | interferon induced transmembrane protein 2 | 0.61 | 0.012 |
| HRASLS | HRAS-like suppressor | 0.62 | 0.017 |
| SIAE | sialic acid acetylesterase | 0.62 | 0.022 |
| MPI | mannose phosphate isomerase | 0.62 | 0.026 |
| ACSM3 | acyl-CoA synthetase medium-chain family member 3 | 0.62 | 0.029 |
| CKB | creatine kinase, brain | 0.62 | 0.005 |
| LINC00663 | long intergenic non-protein coding RNA 663 | 0.62 | 0.024 |
| MIR17HG | miR-17-92 cluster host gene (non-protein coding) | 0.63 | 0.041 |
| LINC00327 | long intergenic non-protein coding RNA 327 | 0.63 | 0.001 |
| FXYD2 | FXYD domain containing ion transport regulator 2 | 0.63 | 0.012 |
| MAP1LC3B2 | microtubule-associated protein 1 light chain 3 beta 2 | 0.63 | 0.031 |
| PFKL | phosphofructokinase, liver | 0.63 | 0.008 |
| CLDN4 | claudin 4 | 0.63 | 0.011 |
| P4HA1 | prolyl 4-hydroxylase, alpha polypeptide I | 0.64 | 0.002 |
| MIOX | myo-inositol oxygenase | 0.64 | 0.012 |
| CTSD | cathepsin D | 0.64 | <0.0001 |
| CECR5 | cat eye syndrome chromosome region, candidate 5 | 0.64 | 0.041 |
| CA11 | carbonic anhydrase XI | 0.64 | 0.011 |
| DNAJB7 | DnaJ (Hsp40) homolog, subfamily B, member 7 | 0.64 | 0.025 |
| GAS2L3 | growth arrest-specific 2 like 3 | 0.64 | 0.036 |
| SHC2 | SHC (Src homology 2 domain containing) transforming protein 2 | 0.65 | 0.043 |
| ONECUT3 | one cut homeobox 3 | 0.65 | 0.021 |
| LOC100130872 | uncharacterized LOC100130872 | 0.65 | 0.008 |
| BCKDK | branched chain ketoacid dehydrogenase kinase | 0.65 | 0.037 |
| MACC1 | metastasis associated in colon cancer 1 | 0.66 | 0.007 |
| SNX33 | sorting nexin 33 | 0.66 | 0.010 |
| ERO1LB | ERO1-like beta (S. cerevisiae) | 0.66 | 0.047 |
| TSPAN33 | tetraspanin 33 | 0.66 | 0.017 |
| FLT4 | fms-related tyrosine kinase 4 | 0.66 | 0.014 |
| IFITM3 | interferon induced transmembrane protein 3 | 0.66 | 0.002 |
| FAM162A | family with sequence similarity 162, member A | 0.66 | 0.015 |
| KLHDC9 | kelch domain containing 9 | 0.66 | 0.003 |
| FLOT1 | flotillin 1 | 0.66 | 0.001 |
| PREPL | prolyl endopeptidase-like | 0.66 | 0.008 |
| PGK1 | phosphoglycerate kinase 1 | 0.66 | 0.027 |
| STBD1 | starch binding domain 1 | 0.66 | 0.039 |
| SLC16A3 | solute carrier family 16, member 3 (monocarboxylic acid transporter 4) | 0.66 | 0.05 |
| ITM2C | integral membrane protein 2C | 0.67 | 0.012 |
| TTC39A | tetratricopeptide repeat domain 39A | 0.67 | 0.025 |
| CELF2 | CUGBP, Elav-like family member 2 | 0.67 | 0.038 |
