# Supplementary material for: Human mesenchymal stromal cells broadly modulate high glucose-induced inflammatory responses of renal proximal tubular cell monolayers
Source: Stem Cell Res Ther. 2019 Nov 19;10:329. doi: 10.1186/s13287-019-1424-5 (PMC6862760; doi:10.1186/s13287-019-1424-5)
Supplement: Supplementary file 10 — Additional file 10: Table S3. List of DEGs with significant Fold Changes in High-Glucose vs Control. [file 13287_2019_1424_MOESM10_ESM.docx]

| **Supplementary Table S3: List of DEGs with significant Fold Changes in High-Glucose vs Control** | | | |
| --- | --- | --- | --- |
|  | Upregulated by HG |  |  |
|  | Downregulated by HG |  |  |
|  |  |  |  |
| **Gene Symbol** | **Gene Description** | **Fold Changes in HG** | **P Value** |
| SEMA3E | sema domain, immunoglobulin domain (Ig), short basic domain, secreted, (semaphorin) 3E | 16.00 | 0.038 |
| PHOSPHO2-KLHL23 | PHOSPHO2-KLHL23 readthrough | 15.65 | 0.0002 |
| IL18RAP | interleukin 18 receptor accessory protein | 12.00 | 0.032 |
| LINC00293 | long intergenic non-protein coding RNA 293 | 9.50 | 0.023 |
| AOC3 | amine oxidase, copper containing 3 (vascular adhesion protein 1) | 8.67 | 0.034 |
| BAGE | B melanoma antigen | 7.67 | 0.039 |
| SNORD76 | small nucleolar RNA, C/D box 76 | 6.49 | 0.013 |
| HCG22 | HLA complex group 22 (non-protein coding) | 6.05 | 0.034 |
| OSR1 | odd-skipped related 1 (Drosophila) | 6.00 | 0.038 |
| ZP3 | zona pellucida glycoprotein 3 (sperm receptor) | 5.76 | 0.017 |
| GLYATL1 | glycine-N-acyltransferase-like 1 | 5.50 | 0.035 |
| KIF7 | kinesin family member 7 | 5.00 | 0.015 |
| FAM179A | family with sequence similarity 179, member A | 4.80 | 0.003 |
| LHFPL3 | lipoma HMGIC fusion partner-like 3 | 4.38 | 0.016 |
| HCN2 | hyperpolarization activated cyclic nucleotide-gated potassium channel 2 | 4.00 | <0.0001 |
| SNORD47 | small nucleolar RNA, C/D box 47 | 3.93 | 0.003 |
| IQCD | IQ motif containing D | 3.79 | 0.041 |
| AQP5 | aquaporin 5 | 3.71 | 0.034 |
| PKD1L3 | polycystic kidney disease 1-like 3 | 3.70 | 0.046 |
| CYP1B1 | cytochrome P450, family 1, subfamily B, polypeptide 1 | 3.46 | 0.039 |
| LOC654342 | lymphocyte-specific protein 1 pseudogene | 3.41 | 0.0004 |
| SCN1B | sodium channel, voltage-gated, type I, beta subunit | 3.37 | 0.027 |
| KNG1 | kininogen 1 | 3.24 | 0.011 |
| SLC12A3 | solute carrier family 12 (sodium/chloride transporters), member 3 | 3.17 | 0.006 |
| IAPP | islet amyloid polypeptide | 3.11 | 0.039 |
| SNCG | synuclein, gamma (breast cancer-specific protein 1) | 3.09 | 0.048 |
| RSU1P2 | Ras suppressor protein 1 pseudogene 2 | 3.07 | 0.008 |
| POLE2 | polymerase (DNA directed), epsilon 2, accessory subunit | 3.01 | 0.049 |
| RRS1-AS1 | uncharacterized LOC100505676 | 2.87 | 0.034 |
| FAM151B | family with sequence similarity 151, member B | 2.71 | 0.029 |
| GDAP1 | ganglioside induced differentiation associated protein 1 | 2.58 | 0.019 |
| C3orf35 | chromosome 3 open reading frame 35 | 2.52 | 0.013 |
| AGER | advanced glycosylation end product-specific receptor | 2.51 | 0.035 |
| EMCN | endomucin | 2.50 | 0.038 |
| RNU2-1 | RNA, U2 small nuclear 1 | 2.48 | 0.028 |
| KRT42P | keratin 42 pseudogene | 2.46 | 0.011 |
| ASB2 | ankyrin repeat and SOCS box containing 2 | 2.46 | 0.002 |
| TUBB1 | tubulin, beta 1 class VI | 2.43 | 0.038 |
| BAI2 | brain-specific angiogenesis inhibitor 2 | 2.42 | 0.030 |
| SLC35G1 | solute carrier family 35, member G1 | 2.38 | 0.037 |
| PAX9 | paired box 9 | 2.35 | 0.010 |
| SFMBT2 | Scm-like with four mbt domains 2 | 2.33 | 0.045 |
| PROB1 | proline-rich basic protein 1 | 2.29 | 0.008 |
| ALDH1A3 | aldehyde dehydrogenase 1 family, member A3 | 2.26 | 0.049 |
| AKAP3 | A kinase (PRKA) anchor protein 3 | 2.25 | 0.013 |
| SH3GL1P1 | SH3-domain GRB2-like 1 pseudogene 1 | 2.23 | 0.017 |
| GRIP2 | glutamate receptor interacting protein 2 | 2.22 | 0.014 |
| C17orf53 | chromosome 17 open reading frame 53 | 2.21 | 0.007 |
| TSACC | TSSK6 activating co-chaperone | 2.20 | 0.025 |
| SSTR2 | somatostatin receptor 2 | 2.19 | 0.037 |
| LINC00921 | uncharacterized protein FLJ39639 | 2.17 | 0.034 |
| LOC100131347 | RAD52 motif 1 pseudogene | 2.16 | 0.002 |
| OR7D2 | olfactory receptor, family 7, subfamily D, member 2 | 2.16 | 0.050 |
| LOC100506469 | uncharacterized LOC100506469 | 2.15 | 0.035 |
| IPCEF1 | interaction protein for cytohesin exchange factors 1 | 2.14 | 0.028 |
| C19orf40 | chromosome 19 open reading frame 40 | 2.14 | 0.041 |
| NLRP8 | NLR family, pyrin domain containing 8 | 2.13 | 0.040 |
| NCRUPAR | non-protein coding RNA, upstream of F2R/PAR1 | 2.13 | 0.025 |
| RLTPR | RGD motif, leucine rich repeats, tropomodulin domain and proline-rich containing | 2.12 | 0.041 |
| FBXL13 | F-box and leucine-rich repeat protein 13 | 2.11 | 0.019 |
| USP2 | ubiquitin specific peptidase 2 | 2.10 | 0.035 |
| C9orf66 | chromosome 9 open reading frame 66 | 2.09 | 0.021 |
| MS4A2 | membrane-spanning 4-domains, subfamily A, member 2 | 2.09 | 0.021 |
| STXBP5-AS1 | STXBP5 antisense RNA 1 | 2.08 | 0.012 |
| ZNF551 | zinc finger protein 551 | 2.07 | 0.022 |
| KRT15 | keratin 15 | 2.06 | 0.039 |
| ZRANB2-AS2 | ZRANB2 antisense RNA 2 (head to head) | 2.05 | 0.044 |
| RAB33A | RAB33A, member RAS oncogene family | 2.05 | 0.024 |
| PPP1R1A | protein phosphatase 1, regulatory (inhibitor) subunit 1A | 2.03 | 0.011 |
| MCF2L-AS1 | MCF2L antisense RNA 1 | 2.00 | 0.025 |
| FIRRE | family with sequence similarity 195, member A pseudogene | 1.93 | 0.010 |
| BMF | Bcl2 modifying factor | 1.92 | 0.003 |
| DBF4B | DBF4 homolog B (S. cerevisiae) | 1.91 | 0.028 |
| ZNF716 | zinc finger protein 716 | 1.86 | 0.039 |
| CCDC28B | coiled-coil domain containing 28B | 1.84 | 0.039 |
| LOC283693 | actin, alpha 2, smooth muscle, aorta pseudogene | 1.84 | 0.036 |
| INGX | inhibitor of growth family, X-linked, pseudogene | 1.83 | 0.004 |
| KLHL14 | kelch-like family member 14 | 1.83 | 0.013 |
| COCH | coagulation factor C homolog, cochlin (Limulus polyphemus) | 1.82 | 0.015 |
| LINC01140 | uncharacterized LOC339524 | 1.82 | 0.012 |
| LHFPL3-AS2 | LHFPL3 antisense RNA 2 | 1.81 | 0.036 |
| GABRE | gamma-aminobutyric acid (GABA) A receptor, epsilon | 1.81 | 0.044 |
| GGT6 | gamma-glutamyltransferase 6 | 1.81 | 0.022 |
| LAIR1 | leukocyte-associated immunoglobulin-like receptor 1 | 1.80 | 0.044 |
| MT1E | metallothionein 1E | 1.80 | 0.012 |
| EMR2 | egf-like module containing, mucin-like, hormone receptor-like 2 | 1.80 | 0.041 |
| RGPD4-AS1 | uncharacterized LOC729121 | 1.79 | 0.041 |
| SLC7A5 | solute carrier family 7 (amino acid transporter light chain, L system), member 5 | 1.78 | 0.022 |
| LINC00574 | long intergenic non-protein coding RNA 574 | 1.77 | 0.043 |
| PKN3 | protein kinase N3 | 1.77 | 0.047 |
| TRIM59 | tripartite motif containing 59 | 1.75 | 0.031 |
| ODC1 | ornithine decarboxylase 1 | 1.74 | 0.0004 |
| KCNQ4 | potassium voltage-gated channel, KQT-like subfamily, member 4 | 1.74 | 0.009 |
| PROSER2-AS1 | PROSER2 antisense RNA 1 | 1.74 | 0.034 |
| ATAT1 | alpha tubulin acetyltransferase 1 | 1.74 | 0.042 |
| TPCN1 | two pore segment channel 1 | 1.73 | 0.039 |
| HMGA2 | high mobility group AT-hook 2 | 1.73 | 0.030 |
| ZNF341 | zinc finger protein 341 | 1.72 | 0.002 |
| H1FX-AS1 | H1FX antisense RNA 1 | 1.71 | 0.007 |
| DNM1P46 | DNM1 pseudogene 46 | 1.71 | 0.014 |
| SMTN | smoothelin | 1.71 | 0.014 |
| KCND1 | potassium voltage-gated channel, Shal-related subfamily, member 1 | 1.69 | 0.035 |
| TEKT4P2 | tektin 4 pseudogene 2 | 1.68 | 0.003 |
| BIN3-IT1 | uncharacterized LOC80094 | 1.67 | 0.029 |
| LTB | lymphotoxin beta (TNF superfamily, member 3) | 1.67 | 0.014 |
| GATSL3 | GATS protein-like 3 | 1.67 | 0.006 |
| TDGF1 | teratocarcinoma-derived growth factor 1 | 1.67 | 0.050 |
| AFG3L1P | AFG3 ATPase family member 3-like 1 (S. cerevisiae), pseudogene | 1.66 | 0.009 |
| KCNH3 | potassium voltage-gated channel, subfamily H (eag-related), member 3 | 1.66 | 0.004 |
| PLSCR3 | phospholipid scramblase 3 | 1.65 | 0.038 |
| PDGFB | platelet-derived growth factor beta polypeptide | 1.65 | 0.003 |
| GRB14 | growth factor receptor-bound protein 14 | 1.63 | 0.008 |
| TPRA1 | transmembrane protein, adipocyte asscociated 1 | 1.63 | 0.005 |
| MLLT4-AS1 | MLLT4 antisense RNA 1 (head to head) | 1.63 | 0.031 |
| SLCO2B1 | solute carrier organic anion transporter family, member 2B1 | 1.62 | <0.0001 |
| SLC4A11 | solute carrier family 4, sodium borate transporter, member 11 | 1.62 | 0.004 |
| VARS2 | valyl-tRNA synthetase 2, mitochondrial | 1.61 | 0.007 |
| LDLRAD2 | low density lipoprotein receptor class A domain containing 2 | 1.61 | 0.032 |
| SSH1 | slingshot protein phosphatase 1 | 1.61 | 0.030 |
| BMP8A | bone morphogenetic protein 8a | 1.61 | 0.040 |
| PRR23C | proline rich 23C | 1.61 | 0.003 |
| TXNRD1 | thioredoxin reductase 1 | 1.60 | 0.045 |
| RDH10 | retinol dehydrogenase 10 (all-trans) | 1.60 | 0.010 |
| FBXL22 | F-box and leucine-rich repeat protein 22 | 1.60 | 0.003 |
| GOLGA7B | golgin A7 family, member B | 1.59 | 0.008 |
| SIX5 | SIX homeobox 5 | 1.58 | 0.029 |
| SLC6A6 | solute carrier family 6 (neurotransmitter transporter, taurine), member 6 | 1.58 | 0.006 |
| STC2 | stanniocalcin 2 | 1.58 | 0.013 |
| PIN4P1 | protein (peptidylprolyl cis/trans isomerase) NIMA-interacting, 4 pseudogene 1 | 1.58 | 0.034 |
| MAL | mal, T-cell differentiation protein | 1.58 | 0.034 |
| CITED4 | Cbp/p300-interacting transactivator, with Glu/Asp-rich carboxy-terminal domain, 4 | 1.58 | 0.012 |
| KCNJ5 | potassium inwardly-rectifying channel, subfamily J, member 5 | 1.58 | 0.035 |
| ULK4 | unc-51-like kinase 4 (C. elegans) | 1.57 | 0.025 |
| PABPC1P2 | poly(A) binding protein, cytoplasmic 1 pseudogene 2 | 1.57 | 0.029 |
| SCIMP | SLP adaptor and CSK interacting membrane protein | 1.57 | 0.041 |
| AP4B1 | adaptor-related protein complex 4, beta 1 subunit | 1.56 | 0.049 |
| DPH7 | WD repeat domain 85 | 1.55 | 0.048 |
| EXOC3L2 | exocyst complex component 3-like 2 | 1.55 | 0.011 |
| TNFAIP8L3 | tumor necrosis factor, alpha-induced protein 8-like 3 | 1.55 | 0.039 |
| NANOG | Nanog homeobox | 1.55 | 0.011 |
| SLC43A1 | solute carrier family 43, member 1 | 1.55 | 0.015 |
| SNORA10 | small nucleolar RNA, H/ACA box 10 | 1.55 | 0.0014 |
| BYSL | bystin-like | 1.55 | 0.034 |
| MPL | myeloproliferative leukemia virus oncogene | 1.55 | 0.028 |
| NRGN | neurogranin (protein kinase C substrate, RC3) | 1.54 | 0.045 |
| TBXAS1 | thromboxane A synthase 1 (platelet) | 1.54 | 0.040 |
| KCNIP3 | Kv channel interacting protein 3, calsenilin | 1.54 | 0.010 |
| DSCAML1 | Down syndrome cell adhesion molecule like 1 | 1.54 | 0.038 |
| RRP1 | ribosomal RNA processing 1 homolog (S. cerevisiae) | 1.54 | 0.033 |
| FAM167A | family with sequence similarity 167, member A | 1.54 | 0.038 |
| FASN | fatty acid synthase | 1.54 | 0.039 |
| NME1-NME2 | NME1-NME2 readthrough | 1.53 | 0.002 |
| IL1RN | interleukin 1 receptor antagonist | 1.53 | 0.040 |
| ST8SIA1 | ST8 alpha-N-acetyl-neuraminide alpha-2,8-sialyltransferase 1 | 1.52 | 0.044 |
| SOX7 | SRY (sex determining region Y)-box 7 | 1.52 | 0.017 |
| HOXB5 | homeobox B5 | 1.52 | 0.026 |
| CTU2 | cytosolic thiouridylase subunit 2 homolog (S. pombe) | 1.52 | 0.043 |
| MT2A | metallothionein 2A | 1.52 | 0.025 |
| ARMC9 | armadillo repeat containing 9 | 1.51 | 0.016 |
| DGKA | diacylglycerol kinase, alpha 80kDa | 1.51 | 0.017 |
| ASMTL-AS1 | ASMTL antisense RNA 1 | 1.51 | 0.034 |
| TCEANC2 | transcription elongation factor A (SII) N-terminal and central domain containing 2 | 1.51 | 0.050 |
| CRYM | crystallin, mu | 1.51 | 0.004 |
| ADRA1B | adrenoceptor alpha 1B | 1.50 | 0.005 |
| SP6 | Sp6 transcription factor | 1.50 | 0.034 |
| PRR15L | proline rich 15-like | 0.04 | 0.001 |
| ADAM7 | ADAM metallopeptidase domain 7 | 0.07 | 0.0002 |
| PLA2G4F | phospholipase A2, group IVF | 0.07 | 0.025 |
| PPFIA4 | protein tyrosine phosphatase, receptor type, f polypeptide (PTPRF), interacting protein (liprin), alpha 4 | 0.07 | 0.043 |
| LOC90246 | uncharacterized LOC90246 | 0.07 | 0.029 |
| PLA1A | phospholipase A1 member A | 0.07 | 0.026 |
| WAS | Wiskott-Aldrich syndrome | 0.08 | 0.039 |
| LIMS3 | LIM and senescent cell antigen-like domains 3 | 0.09 | 0.007 |
| PRR22 | proline rich 22 | 0.09 | 0.046 |
| HIST1H4E | histone cluster 1, H4e | 0.11 | 0.030 |
| BBOX1 | butyrobetaine (gamma), 2-oxoglutarate dioxygenase (gamma-butyrobetaine hydroxylase) 1 | 0.11 | 0.006 |
| SPX | chromosome 12 open reading frame 39 | 0.14 | 0.043 |
| LOC100133920 | uncharacterized LOC100133920 | 0.14 | 0.049 |
| LOC154761 | family with sequence similarity 115, member C pseudogene | 0.14 | 0.004 |
| MYO7B | myosin VIIB | 0.16 | 0.015 |
| C1QTNF4 | C1q and tumor necrosis factor related protein 4 | 0.16 | 0.005 |
| C4BPA | complement component 4 binding protein, alpha | 0.16 | 0.005 |
| PLCH2 | phospholipase C, eta 2 | 0.17 | 0.025 |
| GPNMB | glycoprotein (transmembrane) nmb | 0.17 | 0.0009 |
| TMEM45A | transmembrane protein 45A | 0.17 | 0.032 |
| DNAJC12 | DnaJ (Hsp40) homolog, subfamily C, member 12 | 0.18 | 0.031 |
| ANKRD37 | ankyrin repeat domain 37 | 0.18 | 0.008 |
| LAMA1 | laminin, alpha 1 | 0.19 | 0.014 |
| ATP6V1B1 | ATPase, H+ transporting, lysosomal 56/58kDa, V1 subunit B1 | 0.19 | 0.018 |
| GPR146 | G protein-coupled receptor 146 | 0.19 | 0.0008 |
| HILPDA | hypoxia inducible lipid droplet-associated | 0.20 | 0.006 |
| C4orf47 | chromosome 4 open reading frame 47 | 0.20 | 0.05 |
| DHRS2 | dehydrogenase/reductase (SDR family) member 2 | 0.20 | 0.015 |
| C21orf49 | chromosome 21 open reading frame 49 | 0.20 | 0.030 |
| SCGB1D2 | secretoglobin, family 1D, member 2 | 0.20 | 0.022 |
| STC1 | stanniocalcin 1 | 0.20 | 0.002 |
| GRK4 | G protein-coupled receptor kinase 4 | 0.21 | 0.028 |
| USP51 | ubiquitin specific peptidase 51 | 0.21 | 0.034 |
| ESPN | espin | 0.21 | 0.017 |
| DLL1 | delta-like 1 (Drosophila) | 0.21 | 0.030 |
| ADH6 | alcohol dehydrogenase 6 (class V) | 0.22 | 0.023 |
| PGF | placental growth factor | 0.22 | 0.023 |
| ANGPTL4 | angiopoietin-like 4 | 0.23 | 0.038 |
| PCDHB11 | protocadherin beta 11 | 0.24 | 0.004 |
| CDH17 | cadherin 17, LI cadherin (liver-intestine) | 0.25 | 0.018 |
| FLJ44635 | TPT1-like protein | 0.25 | 0.040 |
| NAPSA | napsin A aspartic peptidase | 0.25 | 0.011 |
| SLC8A1 | solute carrier family 8 (sodium/calcium exchanger), member 1 | 0.26 | 0.025 |
| BLNK | B-cell linker | 0.26 | 0.006 |
| DMGDH | dimethylglycine dehydrogenase | 0.27 | 0.019 |
| AMZ2P1 | archaelysin family metallopeptidase 2 pseudogene 1 | 0.27 | 0.013 |
| RTDR1 | rhabdoid tumor deletion region gene 1 | 0.28 | 0.026 |
| ASTN1 | astrotactin 1 | 0.28 | 0.034 |
| VEPH1 | ventricular zone expressed PH domain-containing 1 | 0.28 | 0.014 |
| LOC286359 | uncharacterized LOC286359 | 0.29 | 0.019 |
| IFIT1 | interferon-induced protein with tetratricopeptide repeats 1 | 0.29 | 0.0007 |
| ABI3BP | ABI family, member 3 (NESH) binding protein | 0.29 | 0.015 |
| SCGB2A2 | secretoglobin, family 2A, member 2 | 0.29 | 0.033 |
| FAM13A-AS1 | FAM13A antisense RNA 1 | 0.29 | 0.0005 |
| GNN | Grp94 neighboring nucleotidase pseudogene | 0.29 | 0.014 |
| MAP7D2 | MAP7 domain containing 2 | 0.29 | 0.015 |
| ADM | adrenomedullin | 0.30 | 0.032 |
| SCGB2A1 | secretoglobin, family 2A, member 1 | 0.30 | 0.008 |
| NDRG1 | N-myc downstream regulated 1 | 0.31 | 0.005 |
| LRRIQ3 | leucine-rich repeats and IQ motif containing 3 | 0.31 | 0.022 |
| ENO2 | enolase 2 (gamma, neuronal) | 0.32 | 0.002 |
| FXYD6 | FXYD domain containing ion transport regulator 6 | 0.32 | 0.05 |
| PFKFB4 | 6-phosphofructo-2-kinase/fructose-2,6-biphosphatase 4 | 0.32 | 0.005 |
| PBX1 | pre-B-cell leukemia homeobox 1 | 0.32 | 0.038 |
| TLR4 | toll-like receptor 4 | 0.32 | 0.049 |
| CLEC2B | C-type lectin domain family 2, member B | 0.32 | 0.008 |
| NTRK2 | neurotrophic tyrosine kinase, receptor, type 2 | 0.33 | 0.002 |
| WBSCR28 | Williams-Beuren syndrome chromosome region 28 | 0.34 | 0.05 |
| TMEM91 | transmembrane protein 91 | 0.34 | 0.007 |
| BEST3 | bestrophin 3 | 0.34 | 0.008 |
| IFIT3 | interferon-induced protein with tetratricopeptide repeats 3 | 0.35 | 0.029 |
| TBC1D3P5 | TBC1 domain family, member 3 pseudogene 5 | 0.35 | 0.032 |
| IGFBP3 | insulin-like growth factor binding protein 3 | 0.36 | 0.001 |
| TSLP | thymic stromal lymphopoietin | 0.36 | 0.040 |
| HLA-DQA1 | major histocompatibility complex, class II, DQ alpha 1 | 0.36 | 0.035 |
| TMEM37 | transmembrane protein 37 | 0.36 | 0.0004 |
| TUBA8 | tubulin, alpha 8 | 0.36 | 0.009 |
| GPX3 | glutathione peroxidase 3 (plasma) | 0.36 | 0.013 |
| NR1H4 | nuclear receptor subfamily 1, group H, member 4 | 0.36 | 0.030 |
| TMED10P1 | transmembrane emp24-like trafficking protein 10 (yeast) pseudogene 1 | 0.37 | 0.028 |
| ENDOD1 | endonuclease domain containing 1 | 0.37 | 0.007 |
| DEFB1 | defensin, beta 1 | 0.37 | 0.004 |
| GBE1 | glucan (1,4-alpha-), branching enzyme 1 | 0.37 | 0.002 |
| SH3D21 | SH3 domain containing 21 | 0.37 | 0.018 |
| HABP2 | hyaluronan binding protein 2 | 0.37 | 0.002 |
| MAP2 | microtubule-associated protein 2 | 0.37 | 0.008 |
| RRN3P2 | RNA polymerase I transcription factor homolog (S. cerevisiae) pseudogene 2 | 0.37 | 0.042 |
| ZBTB37 | zinc finger and BTB domain containing 37 | 0.37 | 0.035 |
| RHOU | ras homolog family member U | 0.37 | 0.022 |
| SMARCD3 | SWI/SNF related, matrix associated, actin dependent regulator of chromatin, subfamily d, member 3 | 0.37 | 0.017 |
| FCGR2C | Fc fragment of IgG, low affinity IIc, receptor for (CD32) (gene/pseudogene) | 0.38 | 0.038 |
| PODNL1 | podocan-like 1 | 0.38 | 0.036 |
| NNAT | neuronatin | 0.38 | 0.007 |
| GPR113 | G protein-coupled receptor 113 | 0.38 | 0.026 |
| RABL2A | RAB, member of RAS oncogene family-like 2A | 0.38 | 0.018 |
| SYNGR4 | synaptogyrin 4 | 0.38 | 0.044 |
| NEBL | nebulette | 0.39 | 0.023 |
| KHDC1 | KH homology domain containing 1 | 0.39 | 0.014 |
| IFI44L | interferon-induced protein 44-like | 0.39 | 0.013 |
| GPBAR1 | G protein-coupled bile acid receptor 1 | 0.39 | 0.024 |
| LTF | lactotransferrin | 0.40 | 0.032 |
| WDR66 | WD repeat domain 66 | 0.40 | 0.034 |
| GOLGA8S | golgin A8 family, member S | 0.41 | 0.013 |
| SEL1L | sel-1 suppressor of lin-12-like (C. elegans) | 0.41 | 0.005 |
| HPSE | heparanase | 0.41 | 0.003 |
| SMIM24 | chromosome 19 open reading frame 77 | 0.41 | 0.045 |
| IL12A | interleukin 12A (natural killer cell stimulatory factor 1, cytotoxic lymphocyte maturation factor 1, p35) | 0.41 | 0.015 |
| MSH5 | mutS homolog 5 (E. coli) | 0.41 | 0.042 |
| NRAP | nebulin-related anchoring protein | 0.42 | 0.024 |
| C6orf222 | chromosome 6 open reading frame 222 | 0.42 | 0.025 |
| HK2 | hexokinase 2 | 0.42 | 0.001 |
| ERO1LB | ERO1-like beta (S. cerevisiae) | 0.42 | 0.004 |
| GIMAP2 | GTPase, IMAP family member 2 | 0.42 | 0.041 |
| SYNM | synemin, intermediate filament protein | 0.42 | 0.045 |
| CRELD2 | cysteine-rich with EGF-like domains 2 | 0.42 | 0.019 |
| ERO1L | ERO1-like (S. cerevisiae) | 0.43 | 0.008 |
| DHDH | dihydrodiol dehydrogenase (dimeric) | 0.43 | 0.022 |
| CP | ceruloplasmin (ferroxidase) | 0.43 | 0.012 |
| LUM | lumican | 0.43 | 0.020 |
| RNASE4 | ribonuclease, RNase A family, 4 | 0.43 | 0.013 |
| BIK | BCL2-interacting killer (apoptosis-inducing) | 0.43 | 0.048 |
| C2CD4C | C2 calcium-dependent domain containing 4C | 0.43 | 0.012 |
| HLA-DPA1 | major histocompatibility complex, class II, DP alpha 1 | 0.43 | 0.027 |
| RASAL2-AS1 | RASAL2 antisense RNA 1 | 0.43 | 0.023 |
| RGMA | RGM domain family, member A | 0.44 | 0.035 |
| HSPA5 | heat shock 70kDa protein 5 (glucose-regulated protein, 78kDa) | 0.44 | 0.022 |
| PTGS1 | prostaglandin-endoperoxide synthase 1 (prostaglandin G/H synthase and cyclooxygenase) | 0.44 | 0.002 |
| CASP1 | caspase 1, apoptosis-related cysteine peptidase | 0.44 | 0.002 |
| TTC18 | tetratricopeptide repeat domain 18 | 0.44 | 0.035 |
| ZNF502 | zinc finger protein 502 | 0.44 | 0.028 |
| CNTNAP1 | contactin associated protein 1 | 0.45 | 0.016 |
| CKB | creatine kinase, brain | 0.45 | 0.007 |
| ESRRG | estrogen-related receptor gamma | 0.45 | 0.030 |
| MIR210HG | MIR210 host gene (non-protein coding) | 0.45 | 0.013 |
| RASD1 | RAS, dexamethasone-induced 1 | 0.45 | 0.002 |
| BNIP3 | BCL2/adenovirus E1B 19kDa interacting protein 3 | 0.45 | 0.002 |
| CFB | complement factor B | 0.45 | 0.002 |
| TDRG1 | testis development related 1 (non-protein coding) | 0.45 | 0.0008 |
| SAPCD1 | suppressor APC domain containing 1 | 0.45 | 0.033 |
| RSG1 | REM2 and RAB-like small GTPase 1 | 0.45 | 0.003 |
| PDK1 | pyruvate dehydrogenase kinase, isozyme 1 | 0.45 | 0.008 |
| PDIA4 | protein disulfide isomerase family A, member 4 | 0.46 | 0.011 |
| TMC4 | transmembrane channel-like 4 | 0.46 | 0.018 |
| ACSS1 | acyl-CoA synthetase short-chain family member 1 | 0.46 | 0.010 |
| NUPR1 | nuclear protein, transcriptional regulator, 1 | 0.46 | 0.020 |
| PTH1R | parathyroid hormone 1 receptor | 0.46 | 0.010 |
| C4orf3 | chromosome 4 open reading frame 3 | 0.46 | 0.006 |
| SEPP1 | selenoprotein P, plasma, 1 | 0.46 | 0.043 |
| NEU3 | sialidase 3 (membrane sialidase) | 0.46 | 0.021 |
| CTSF | cathepsin F | 0.46 | 0.007 |
| RAB27B | RAB27B, member RAS oncogene family | 0.46 | 0.016 |
| RNF217 | ring finger protein 217 | 0.46 | 0.026 |
| SPEF1 | sperm flagellar 1 | 0.46 | 0.039 |
| SLC3A1 | solute carrier family 3 (cystine, dibasic and neutral amino acid transporters, activator of cystine, dibasic and neutral amino acid transport), member 1 | 0.47 | 0.006 |
| BTC | betacellulin | 0.47 | 0.035 |
| NCKIPSD | NCK interacting protein with SH3 domain | 0.47 | 0.010 |
| P4HA1 | prolyl 4-hydroxylase, alpha polypeptide I | 0.47 | 0.032 |
| SLMO1 | slowmo homolog 1 (Drosophila) | 0.47 | 0.044 |
| PHYHIP | phytanoyl-CoA 2-hydroxylase interacting protein | 0.48 | 0.032 |
| RAP2B | RAP2B, member of RAS oncogene family | 0.48 | 0.012 |
| ZNF808 | zinc finger protein 808 | 0.48 | 0.05 |
| PGK1 | phosphoglycerate kinase 1 | 0.48 | 0.008 |
| KLHDC7A | kelch domain containing 7A | 0.48 | 0.049 |
| C2 | complement component 2 | 0.48 | 0.027 |
| FUT11 | fucosyltransferase 11 (alpha (1,3) fucosyltransferase) | 0.48 | 0.040 |
| NXNL2 | nucleoredoxin-like 2 | 0.48 | 0.002 |
| PLOD2 | procollagen-lysine, 2-oxoglutarate 5-dioxygenase 2 | 0.48 | 0.030 |
| LIX1 | Lix1 homolog (chicken) | 0.49 | 0.032 |
| NBEAL1 | neurobeachin-like 1 | 0.49 | 0.05 |
| LINC-PINT | uncharacterized LOC378805 | 0.49 | 0.020 |
| STBD1 | starch binding domain 1 | 0.49 | 0.017 |
| CCL28 | chemokine (C-C motif) ligand 28 | 0.49 | 0.006 |
| MACROD2 | MACRO domain containing 2 | 0.49 | 0.021 |
| MORN3 | MORN repeat containing 3 | 0.50 | 0.028 |
| SOX8 | SRY (sex determining region Y)-box 8 | 0.50 | 0.005 |
| DYNAP | dynactin associated protein | 0.50 | 0.022 |
| CRYAB | crystallin, alpha B | 0.50 | 0.028 |
| PRSS22 | protease, serine, 22 | 0.50 | 0.028 |
| RNF150 | ring finger protein 150 | 0.50 | 0.019 |
| ZEB2 | zinc finger E-box binding homeobox 2 | 0.50 | 0.006 |
| AATK | apoptosis-associated tyrosine kinase | 0.50 | 0.023 |
| NFATC2 | nuclear factor of activated T-cells, cytoplasmic, calcineurin-dependent 2 | 0.50 | 0.034 |
| ITGA4 | integrin, alpha 4 (antigen CD49D, alpha 4 subunit of VLA-4 receptor) | 0.50 | 0.035 |
| C5 | complement component 5 | 0.50 | 0.044 |
| BEX2 | brain expressed X-linked 2 | 0.50 | 0.042 |
| CCDC153 | coiled-coil domain containing 153 | 0.50 | 0.042 |
| HSP90B1 | heat shock protein 90kDa beta (Grp94), member 1 | 0.50 | 0.009 |
| AGR2 | anterior gradient 2 homolog (Xenopus laevis) | 0.50 | 0.004 |
| PRKAR2A | protein kinase, cAMP-dependent, regulatory, type II, alpha | 0.50 | 0.046 |
| SNX33 | sorting nexin 33 | 0.51 | 0.008 |
| IL1RL1 | interleukin 1 receptor-like 1 | 0.51 | 0.044 |
| SLC2A1 | solute carrier family 2 (facilitated glucose transporter), member 1 | 0.51 | 0.026 |
| CGREF1 | cell growth regulator with EF-hand domain 1 | 0.51 | 0.009 |
| EHD3 | EH-domain containing 3 | 0.51 | 0.017 |
| THAP8 | THAP domain containing 8 | 0.51 | 0.039 |
| IFIT2 | interferon-induced protein with tetratricopeptide repeats 2 | 0.52 | 0.005 |
| C16orf74 | chromosome 16 open reading frame 74 | 0.52 | 0.002 |
| LOX | lysyl oxidase | 0.52 | 0.005 |
| TMEM182 | transmembrane protein 182 | 0.52 | 0.003 |
| SNHG12 | small nucleolar RNA host gene 12 (non-protein coding) | 0.52 | 0.022 |
| ULBP1 | UL16 binding protein 1 | 0.53 | 0.017 |
| RRAGB | Ras-related GTP binding B | 0.53 | 0.035 |
| INTU | inturned planar cell polarity protein | 0.53 | 0.0008 |
| ZNF12 | zinc finger protein 12 | 0.53 | 0.027 |
| TVP23C-CDRT4 | TVP23C-CDRT4 readthrough | 0.53 | 0.046 |
| SLC6A13 | solute carrier family 6 (neurotransmitter transporter, GABA), member 13 | 0.53 | 0.023 |
| SEC11C | SEC11 homolog C (S. cerevisiae) | 0.54 | 0.026 |
| GMDS-AS1 | uncharacterized LOC100508120 | 0.54 | 0.034 |
| GAS2L3 | growth arrest-specific 2 like 3 | 0.54 | 0.020 |
| APOL1 | apolipoprotein L, 1 | 0.54 | 0.018 |
| FABP3 | fatty acid binding protein 3, muscle and heart (mammary-derived growth inhibitor) | 0.54 | 0.031 |
| NUCB2 | nucleobindin 2 | 0.54 | 0.023 |
| MFSD2A | major facilitator superfamily domain containing 2A | 0.54 | 0.040 |
| RAP1GAP | RAP1 GTPase activating protein | 0.54 | 0.012 |
| ANG | angiogenin, ribonuclease, RNase A family, 5 | 0.54 | 0.038 |
| HLA-DRB5 | major histocompatibility complex, class II, DR beta 5 | 0.54 | 0.030 |
| TRIB2 | tribbles homolog 2 (Drosophila) | 0.54 | 0.023 |
| FAM162A | family with sequence similarity 162, member A | 0.54 | 0.001 |
| IFITM10 | interferon induced transmembrane protein 10 | 0.54 | 0.033 |
| ZNF165 | zinc finger protein 165 | 0.55 | 0.014 |
| MALL | mal, T-cell differentiation protein-like | 0.55 | 0.001 |
| FAM110C | family with sequence similarity 110, member C | 0.55 | 0.028 |
| ZNF860 | zinc finger protein 860 | 0.55 | 0.012 |
| DENND5B-AS1 | DENND5B antisense RNA 1 | 0.55 | 0.010 |
| PPFIBP2 | PTPRF interacting protein, binding protein 2 (liprin beta 2) | 0.55 | 0.023 |
| CFI | complement factor I | 0.55 | 0.022 |
| CDK18 | cyclin-dependent kinase 18 | 0.55 | 0.015 |
| CACNA1H | calcium channel, voltage-dependent, T type, alpha 1H subunit | 0.55 | 0.028 |
| ARRDC3 | arrestin domain containing 3 | 0.56 | 0.017 |
| HIST2H2BE | histone cluster 2, H2be | 0.56 | 0.024 |
| SIAE | sialic acid acetylesterase | 0.56 | 0.022 |
| BBS1 | Bardet-Biedl syndrome 1 | 0.56 | 0.010 |
| HLA-DRA | major histocompatibility complex, class II, DR alpha | 0.56 | 0.016 |
| HGD | homogentisate 1,2-dioxygenase | 0.56 | 0.006 |
| SERPINI1 | serpin peptidase inhibitor, clade I (neuroserpin), member 1 | 0.56 | 0.022 |
| LOC728323 | uncharacterized LOC728323 | 0.56 | 0.040 |
| LGI2 | leucine-rich repeat LGI family, member 2 | 0.56 | 0.038 |
| ZNF23 | zinc finger protein 23 | 0.56 | 0.009 |
| PDXP | pyridoxal (pyridoxine, vitamin B6) phosphatase | 0.56 | 0.009 |
| PIGR | polymeric immunoglobulin receptor | 0.56 | 0.012 |
| FAM84A | family with sequence similarity 84, member A | 0.57 | 0.002 |
| HYOU1 | hypoxia up-regulated 1 | 0.57 | 0.017 |
| DNM1 | dynamin 1 | 0.57 | 0.031 |
| SHC4 | SHC (Src homology 2 domain containing) family, member 4 | 0.57 | 0.009 |
| GALNT1 | UDP-N-acetyl-alpha-D-galactosamine:polypeptide N-acetylgalactosaminyltransferase 1 (GalNAc-T1) | 0.57 | 0.026 |
| NME7 | NME/NM23 family member 7 | 0.58 | 0.010 |
| LTC4S | leukotriene C4 synthase | 0.58 | 0.029 |
| NPR1 | natriuretic peptide receptor A/guanylate cyclase A (atrionatriuretic peptide receptor A) | 0.58 | 0.007 |
| C14orf132 | chromosome 14 open reading frame 132 | 0.58 | 0.008 |
| AK4 | adenylate kinase 4 | 0.58 | 0.015 |
| HMGN3-AS1 | uncharacterized LOC100288198 | 0.58 | 0.008 |
| SCN9A | sodium channel, voltage-gated, type IX, alpha subunit | 0.58 | 0.039 |
| SLC25A36 | solute carrier family 25 (pyrimidine nucleotide carrier ), member 36 | 0.58 | 0.002 |
| INSIG2 | insulin induced gene 2 | 0.58 | 0.044 |
| DKK1 | dickkopf 1 homolog (Xenopus laevis) | 0.58 | 0.035 |
| IFITM1 | interferon induced transmembrane protein 1 | 0.59 | 0.044 |
| ATP1B1 | ATPase, Na+/K+ transporting, beta 1 polypeptide | 0.59 | 0.020 |
| CRELD1 | cysteine-rich with EGF-like domains 1 | 0.59 | 0.008 |
| CCT6P1 | chaperonin containing TCP1, subunit 6 (zeta) pseudogene 1 | 0.59 | 0.047 |
| VWA7 | von Willebrand factor A domain containing 7 | 0.59 | 0.046 |
| DNAJB7 | DnaJ (Hsp40) homolog, subfamily B, member 7 | 0.59 | 0.003 |
| CHIC2 | cysteine-rich hydrophobic domain 2 | 0.59 | 0.041 |
| MXI1 | MAX interactor 1, dimerization protein | 0.59 | 0.010 |
| NUP62CL | nucleoporin 62kDa C-terminal like | 0.59 | 0.040 |
| ZEB1-AS1 | ZEB1 antisense RNA 1 | 0.60 | 0.048 |
| CFH | complement factor H | 0.60 | 0.011 |
| SECTM1 | secreted and transmembrane 1 | 0.60 | 0.010 |
| FAM222A | family with sequence similarity 222, member A | 0.60 | 0.018 |
| LGMN | legumain | 0.60 | 0.007 |
| PREX2 | phosphatidylinositol-3,4,5-trisphosphate-dependent Rac exchange factor 2 | 0.60 | 0.024 |
| BCKDK | branched chain ketoacid dehydrogenase kinase | 0.60 | 0.029 |
| NAMPT | nicotinamide phosphoribosyltransferase | 0.60 | 0.017 |
| MYH7B | myosin, heavy chain 7B, cardiac muscle, beta | 0.60 | 0.015 |
| PROS1 | protein S (alpha) | 0.60 | 0.027 |
| PBLD | phenazine biosynthesis-like protein domain containing | 0.60 | 0.034 |
| CLU | clusterin | 0.60 | 0.038 |
| ATP8B3 | ATPase, aminophospholipid transporter, class I, type 8B, member 3 | 0.60 | 0.032 |
| FRK | fyn-related kinase | 0.60 | 0.028 |
| TTC39A | tetratricopeptide repeat domain 39A | 0.61 | 0.023 |
| TMTC2 | transmembrane and tetratricopeptide repeat containing 2 | 0.61 | 0.047 |
| DNAJB11 | DnaJ (Hsp40) homolog, subfamily B, member 11 | 0.61 | 0.025 |
| TMEM213 | transmembrane protein 213 | 0.61 | 0.004 |
| C2orf88 | chromosome 2 open reading frame 88 | 0.61 | 0.013 |
| TCN2 | transcobalamin II | 0.61 | 0.026 |
| EGLN1 | egl nine homolog 1 (C. elegans) | 0.61 | 0.011 |
| RHOQ | ras homolog family member Q | 0.61 | 0.002 |
| FAM210A | family with sequence similarity 210, member A | 0.61 | 0.019 |
| MME | membrane metallo-endopeptidase | 0.61 | 0.019 |
| TPI1 | triosephosphate isomerase 1 | 0.61 | 0.025 |
| ABHD16B | abhydrolase domain containing 16B | 0.61 | 0.025 |
| ZFAND2A | zinc finger, AN1-type domain 2A | 0.62 | 0.032 |
| PREPL | prolyl endopeptidase-like | 0.62 | 0.047 |
| ZNF559 | zinc finger protein 559 | 0.62 | 0.019 |
| CSRNP1 | cysteine-serine-rich nuclear protein 1 | 0.62 | 0.039 |
| ENPP5 | ectonucleotide pyrophosphatase/phosphodiesterase 5 (putative) | 0.62 | 0.018 |
| TIGD2 | tigger transposable element derived 2 | 0.62 | 0.004 |
| TRABD2B | TraB domain containing 2B | 0.62 | 0.035 |
| MAN2B2 | mannosidase, alpha, class 2B, member 2 | 0.62 | 0.020 |
| GPRC5A | G protein-coupled receptor, family C, group 5, member A | 0.62 | 0.004 |
| IPMK | inositol polyphosphate multikinase | 0.62 | 0.044 |
| HPS3 | Hermansky-Pudlak syndrome 3 | 0.62 | 0.040 |
| PTPN20B | protein tyrosine phosphatase, non-receptor type 20B | 0.62 | 0.008 |
| EEPD1 | endonuclease/exonuclease/phosphatase family domain containing 1 | 0.62 | 0.039 |
| ETV3 | ets variant 3 | 0.62 | 0.019 |
| CDH16 | cadherin 16, KSP-cadherin | 0.62 | 0.004 |
| MTFP1 | mitochondrial fission process 1 | 0.63 | 0.018 |
| GAB1 | GRB2-associated binding protein 1 | 0.63 | 0.031 |
| GBP2 | guanylate binding protein 2, interferon-inducible | 0.63 | 0.018 |
| FLJ23867 | uncharacterized protein FLJ23867 | 0.63 | 0.032 |
| FOLR2 | folate receptor 2 (fetal) | 0.63 | 0.002 |
| PFKFB3 | 6-phosphofructo-2-kinase/fructose-2,6-biphosphatase 3 | 0.63 | 0.016 |
| SH3RF1 | SH3 domain containing ring finger 1 | 0.63 | 0.0003 |
| IRS2 | insulin receptor substrate 2 | 0.63 | 0.006 |
| EPB41L4B | erythrocyte membrane protein band 4.1 like 4B | 0.63 | 0.014 |
| BNIP3L | BCL2/adenovirus E1B 19kDa interacting protein 3-like | 0.64 | 0.003 |
| MILR1 | mast cell immunoglobulin-like receptor 1 | 0.64 | 0.019 |
| RGS17 | regulator of G-protein signaling 17 | 0.64 | 0.022 |
| METTL10 | methyltransferase like 10 | 0.64 | 0.012 |
| HLA-DQB1 | major histocompatibility complex, class II, DQ beta 1 | 0.64 | 0.009 |
| ARHGEF19 | Rho guanine nucleotide exchange factor (GEF) 19 | 0.64 | 0.041 |
| PLEKHA8P1 | pleckstrin homology domain containing, family A member 8 pseudogene 1 | 0.64 | 0.031 |
| HSF2BP | heat shock transcription factor 2 binding protein | 0.64 | 0.014 |
| ZNF543 | zinc finger protein 543 | 0.64 | 0.0007 |
| NOV | nephroblastoma overexpressed | 0.64 | 0.019 |
| ERMARD | chromosome 6 open reading frame 70 | 0.64 | 0.008 |
| NEK6 | NIMA-related kinase 6 | 0.64 | 0.011 |
| ALDH6A1 | aldehyde dehydrogenase 6 family, member A1 | 0.64 | 0.042 |
| SYTL2 | synaptotagmin-like 2 | 0.64 | 0.028 |
| HERC3 | HECT and RLD domain containing E3 ubiquitin protein ligase 3 | 0.64 | 0.021 |
| ITGB2-AS1 | ITGB2 antisense RNA 1 | 0.64 | 0.035 |
| PKIB | protein kinase (cAMP-dependent, catalytic) inhibitor beta | 0.64 | 0.010 |
| RC3H2 | ring finger and CCCH-type domains 2 | 0.64 | 0.013 |
| MALAT1 | metastasis associated lung adenocarcinoma transcript 1 (non-protein coding) | 0.65 | 0.006 |
| SPIRE1 | spire homolog 1 (Drosophila) | 0.65 | 0.0006 |
| ZNF33B | zinc finger protein 33B | 0.65 | 0.030 |
| MANF | mesencephalic astrocyte-derived neurotrophic factor | 0.65 | 0.030 |
| PGBD5 | piggyBac transposable element derived 5 | 0.65 | 0.002 |
| OR7E37P | olfactory receptor, family 7, subfamily E, member 37 pseudogene | 0.65 | 0.003 |
| TMEM47 | transmembrane protein 47 | 0.65 | 0.004 |
| PDIA3 | protein disulfide isomerase family A, member 3 | 0.65 | 0.003 |
| CD74 | CD74 molecule, major histocompatibility complex, class II invariant chain | 0.65 | 0.041 |
| IL7 | interleukin 7 | 0.65 | 0.041 |
| FEM1C | fem-1 homolog c (C. elegans) | 0.65 | 0.018 |
| BACE1 | beta-site APP-cleaving enzyme 1 | 0.65 | 0.017 |
| MAOA | monoamine oxidase A | 0.65 | 0.036 |
| LOC399715 | uncharacterized LOC399715 | 0.66 | 0.020 |
| SLC35A3 | solute carrier family 35 (UDP-N-acetylglucosamine (UDP-GlcNAc) transporter), member A3 | 0.66 | 0.031 |
| GNGT1 | guanine nucleotide binding protein (G protein), gamma transducing activity polypeptide 1 | 0.66 | 0.014 |
| USP27X | ubiquitin specific peptidase 27, X-linked | 0.66 | 0.008 |
| KLHDC10 | kelch domain containing 10 | 0.66 | 0.027 |
| CTSA | cathepsin A | 0.66 | 0.002 |
| ASAH1 | N-acylsphingosine amidohydrolase (acid ceramidase) 1 | 0.66 | 0.005 |
| LYRM9 | LYR motif containing 9 | 0.66 | 0.002 |
| PFKL | phosphofructokinase, liver | 0.66 | 0.003 |
| BATF2 | basic leucine zipper transcription factor, ATF-like 2 | 0.66 | 0.009 |
| LINC01158 | uncharacterized LOC100506421 | 0.66 | 0.021 |
| CAMSAP1 | calmodulin regulated spectrin-associated protein 1 | 0.66 | 0.0002 |
| SYNE1 | spectrin repeat containing, nuclear envelope 1 | 0.66 | 0.020 |
| RNF24 | ring finger protein 24 | 0.66 | 0.036 |
| BRAF | v-raf murine sarcoma viral oncogene homolog B1 | 0.66 | 0.025 |
| PPIP5K1 | diphosphoinositol pentakisphosphate kinase 1 | 0.66 | 0.029 |
| RHBDL1 | rhomboid, veinlet-like 1 (Drosophila) | 0.67 | 0.021 |
