# Supplementary material for: Human mesenchymal stromal cells broadly modulate high glucose-induced inflammatory responses of renal proximal tubular cell monolayers
Source: Stem Cell Res Ther. 2019 Nov 19;10:329. doi: 10.1186/s13287-019-1424-5 (PMC6862760; doi:10.1186/s13287-019-1424-5)
Supplement: Supplementary file 11 — Additional file 11: Table S4. List of DEGs with significant Fold Changes in Mannitol vs Control. [file 13287_2019_1424_MOESM11_ESM.docx]

| **Supplementary Table S4: List of DEGs with significant Fold Changes in Mannitol vs Control** | | | |
| --- | --- | --- | --- |
|  | Upregulated by MAN |  |  |
|  | Downregulated by MAN |  |  |
|  |  |  |  |
| **Gene Symbol** | **Gene Description** | **Fold Changes in MAN** | **P Value** |
| CECR7 | cat eye syndrome chromosome region, candidate 7 (non-protein coding) | 17.00 | 0.015 |
| NELL2 | NEL-like 2 (chicken) | 12.00 | 0.032 |
| S1PR1 | sphingosine-1-phosphate receptor 1 | 11.50 | 0.044 |
| CCDC110 | coiled-coil domain containing 110 | 11.33 | 0.05 |
| RTN4RL2 | reticulon 4 receptor-like 2 | 9.33 | 0.028 |
| GRIA3 | glutamate receptor, ionotropic, AMPA 3 | 9.00 | 0.015 |
| LOC100287072 | ribosomal protein S6 kinase, 70kDa, polypeptide 1 pseudogene | 8.00 | 0.049 |
| TOP1P1 | topoisomerase (DNA) I pseudogene 1 | 7.75 | 0.05 |
| LOC440461 | Rho GTPase activating protein 27 pseudogene | 7.75 | 0.05 |
| PIK3CG | phosphatidylinositol-4,5-bisphosphate 3-kinase, catalytic subunit gamma | 7.00 | 0.016 |
| MST1P2 | macrophage stimulating 1 (hepatocyte growth factor-like) pseudogene 2 | 6.86 | 0.025 |
| PCDHB11 | protocadherin beta 11 | 6.83 | 0.033 |
| ZNF625-ZNF20 | ZNF625-ZNF20 readthrough | 6.32 | 0.037 |
| DCAF8L2 | DDB1 and CUL4 associated factor 8-like 2 | 6.00 | 0.013 |
| LIMS3 | LIM and senescent cell antigen-like domains 3 | 5.69 | 0.036 |
| FAM178B | family with sequence similarity 178, member B | 5.63 | 0.005 |
| OR7E12P | olfactory receptor, family 7, subfamily E, member 12 pseudogene | 5.47 | 0.045 |
| TGM1 | transglutaminase 1 (K polypeptide epidermal type I, protein-glutamine-gamma-glutamyltransferase) | 5.31 | 0.0003 |
| KCNK4 | potassium channel, subfamily K, member 4 | 5.25 | 0.003 |
| USP27X-AS1 | uncharacterized LOC158572 | 5.20 | 0.031 |
| LINC00488 | long intergenic non-protein coding RNA 488 | 5.18 | 0.037 |
| ANXA2R | annexin A2 receptor | 4.91 | 0.046 |
| TIGD4 | tigger transposable element derived 4 | 4.77 | 0.011 |
| DGCR9 | DiGeorge syndrome critical region gene 9 | 4.63 | 0.018 |
| PGAM4 | phosphoglycerate mutase family member 4 | 4.50 | 0.044 |
| MGARP | mitochondria-localized glutamic acid-rich protein | 4.43 | 0.015 |
| MCIDAS | multiciliate differentiation and DNA synthesis associated cell cycle protein | 4.33 | 0.035 |
| KHDC1 | KH homology domain containing 1 | 3.81 | 0.018 |
| AANAT | aralkylamine N-acetyltransferase | 3.78 | 0.002 |
| TMEM45A | transmembrane protein 45A | 3.61 | 0.045 |
| SNORA45A | small nucleolar RNA, H/ACA box 3 | 3.59 | 0.008 |
| CCDC171 | coiled-coil domain containing 171 | 3.50 | 0.038 |
| FGF14-AS2 | FGF14 antisense RNA 2 | 3.34 | 0.025 |
| ZFPM2 | zinc finger protein, FOG family member 2 | 3.25 | 0.05 |
| FAM13A | family with sequence similarity 13, member A | 3.24 | 0.033 |
| LOC100506071 | uncharacterized LOC100506071 | 3.16 | 0.019 |
| LOC100288123 | uncharacterized LOC100288123 | 3.16 | 0.048 |
| LINC01144 | uncharacterized LOC400752 | 3.08 | 0.038 |
| ARC | activity-regulated cytoskeleton-associated protein | 3.00 | 0.012 |
| CIB2 | calcium and integrin binding family member 2 | 3.00 | 0.047 |
| TTC25 | tetratricopeptide repeat domain 25 | 2.95 | 0.008 |
| CLEC2B | C-type lectin domain family 2, member B | 2.85 | 0.047 |
| RAX2 | retina and anterior neural fold homeobox 2 | 2.82 | 0.010 |
| LEP | leptin | 2.77 | 0.024 |
| PGBD4 | piggyBac transposable element derived 4 | 2.75 | 0.015 |
| BASP1P1 | brain abundant, membrane attached signal protein 1 pseudogene 1 | 2.71 | 0.014 |
| GMDS-AS1 | uncharacterized LOC100508120 | 2.69 | 0.020 |
| PLA1A | phospholipase A1 member A | 2.67 | 0.033 |
| DNAH7 | dynein, axonemal, heavy chain 7 | 2.65 | 0.019 |
| ACER2 | alkaline ceramidase 2 | 2.57 | 0.0006 |
| ANKRD37 | ankyrin repeat domain 37 | 2.56 | 0.026 |
| SNORD99 | small nucleolar RNA, C/D box 99 | 2.55 | 0.012 |
| DMGDH | dimethylglycine dehydrogenase | 2.53 | 0.031 |
| CUZD1 | CUB and zona pellucida-like domains 1 | 2.52 | 0.012 |
| IGFBP3 | insulin-like growth factor binding protein 3 | 2.48 | 0.004 |
| TMEM232 | transmembrane protein 232 | 2.47 | 0.046 |
| GEMIN8P4 | gem (nuclear organelle) associated protein 8 pseudogene 4 | 2.47 | 0.044 |
| CNTNAP2 | contactin associated protein-like 2 | 2.46 | 0.034 |
| CRMP1 | collapsin response mediator protein 1 | 2.43 | 0.021 |
| PGF | placental growth factor | 2.39 | 0.028 |
| LINC01003 | uncharacterized LOC100128822 | 2.39 | 0.009 |
| CCL26 | chemokine (C-C motif) ligand 26 | 2.36 | 0.026 |
| LOC441461 | uncharacterized LOC441461 | 2.36 | 0.039 |
| ZCWPW1 | zinc finger, CW type with PWWP domain 1 | 2.33 | 0.018 |
| CACHD1 | cache domain containing 1 | 2.32 | 0.020 |
| ACADL | acyl-CoA dehydrogenase, long chain | 2.31 | 0.016 |
| COL7A1 | collagen, type VII, alpha 1 | 2.30 | 0.033 |
| ATL1 | atlastin GTPase 1 | 2.30 | 0.006 |
| ADM | adrenomedullin | 2.29 | 0.036 |
| AGAP5 | ArfGAP with GTPase domain, ankyrin repeat and PH domain 5 | 2.28 | 0.014 |
| TMEM182 | transmembrane protein 182 | 2.27 | 0.002 |
| MYH7B | myosin, heavy chain 7B, cardiac muscle, beta | 2.22 | 0.008 |
| ACTRT3 | actin-related protein T3 | 2.22 | 0.020 |
| CBWD6 | COBW domain containing 6 | 2.21 | 0.023 |
| LOC440311 | glioma tumor suppressor candidate region gene 2 pseudogene | 2.20 | 0.020 |
| TPPP3 | tubulin polymerization-promoting protein family member 3 | 2.20 | 0.029 |
| NHLRC1 | NHL repeat containing 1 | 2.18 | 0.023 |
| LRRC34 | leucine rich repeat containing 34 | 2.18 | 0.023 |
| LINC00410 | long intergenic non-protein coding RNA 410 | 2.18 | 0.033 |
| NAALADL2 | N-acetylated alpha-linked acidic dipeptidase-like 2 | 2.17 | 0.028 |
| HILPDA | hypoxia inducible lipid droplet-associated | 2.16 | 0.021 |
| C10orf99 | chromosome 10 open reading frame 99 | 2.15 | 0.034 |
| CEP83 | coiled-coil domain containing 41 | 2.15 | 0.013 |
| APBB3 | amyloid beta (A4) precursor protein-binding, family B, member 3 | 2.12 | 0.009 |
| ZNF334 | zinc finger protein 334 | 2.11 | 0.010 |
| FOXN3-AS1 | FOXN3 antisense RNA 1 | 2.11 | 0.034 |
| LAMA1 | laminin, alpha 1 | 2.10 | 0.008 |
| DNAH12 | dynein, axonemal, heavy chain 12 | 2.10 | 0.032 |
| C7orf63 | chromosome 7 open reading frame 63 | 2.10 | 0.026 |
| SNORA25 | small nucleolar RNA, H/ACA box 25 | 2.07 | 0.028 |
| DENND5B-AS1 | DENND5B antisense RNA 1 | 2.06 | 0.008 |
| MADCAM1 | mucosal vascular addressin cell adhesion molecule 1 | 2.06 | 0.019 |
| ZBTB37 | zinc finger and BTB domain containing 37 | 2.02 | 0.007 |
| TTC14 | tetratricopeptide repeat domain 14 | 2.02 | 0.007 |
| SOX8 | SRY (sex determining region Y)-box 8 | 2.01 | 0.049 |
| ABCC6 | ATP-binding cassette, sub-family C (CFTR/MRP), member 6 | 2.01 | 0.019 |
| ERO1L | ERO1-like (S. cerevisiae) | 1.98 | 0.031 |
| RAD51AP1 | RAD51 associated protein 1 | 1.97 | 0.026 |
| XIRP1 | xin actin-binding repeat containing 1 | 1.96 | 0.027 |
| LOC643770 | uncharacterized LOC643770 | 1.96 | 0.003 |
| ARSF | arylsulfatase F | 1.95 | 0.027 |
| IL1RL1 | interleukin 1 receptor-like 1 | 1.94 | 0.032 |
| C20orf203 | chromosome 20 open reading frame 203 | 1.93 | 0.001 |
| LINC-PINT | uncharacterized LOC378805 | 1.92 | 0.005 |
| ABCA5 | ATP-binding cassette, sub-family A (ABC1), member 5 | 1.92 | 0.041 |
| SEL1L | sel-1 suppressor of lin-12-like (C. elegans) | 1.92 | 0.028 |
| SOX6 | SRY (sex determining region Y)-box 6 | 1.91 | 0.038 |
| N4BP2L2-IT2 | N4BPL2 intronic transcript 2 (non-protein coding) | 1.91 | 0.044 |
| UBA6-AS1 | uncharacterized LOC550112 | 1.91 | 0.014 |
| HIST1H2BG | histone cluster 1, H2bg | 1.90 | 0.049 |
| ADAM11 | ADAM metallopeptidase domain 11 | 1.89 | 0.042 |
| SEC11C | SEC11 homolog C (S. cerevisiae) | 1.89 | 0.018 |
| ARRDC4 | arrestin domain containing 4 | 1.88 | 0.024 |
| LRRC49 | leucine rich repeat containing 49 | 1.88 | 0.027 |
| PDE5A | phosphodiesterase 5A, cGMP-specific | 1.87 | 0.007 |
| GEN1 | Gen endonuclease homolog 1 (Drosophila) | 1.86 | 0.016 |
| PP2D1 | protein phosphatase 2C-like domain containing 1 | 1.84 | 0.013 |
| RNF217 | ring finger protein 217 | 1.83 | 0.014 |
| CGREF1 | cell growth regulator with EF-hand domain 1 | 1.83 | 0.015 |
| FBXO5 | F-box protein 5 | 1.82 | 0.0009 |
| TIGD2 | tigger transposable element derived 2 | 1.82 | 0.047 |
| PIK3IP1 | phosphoinositide-3-kinase interacting protein 1 | 1.82 | 0.028 |
| DCAF17 | DDB1 and CUL4 associated factor 17 | 1.81 | 0.009 |
| ZNF284 | zinc finger protein 284 | 1.80 | 0.049 |
| PARD6G-AS1 | PARD6G antisense RNA 1 | 1.79 | 0.048 |
| HIST1H2BD | histone cluster 1, H2bd | 1.79 | 0.035 |
| LINC01011 | uncharacterized LOC401232 | 1.78 | 0.018 |
| PCGF6 | polycomb group ring finger 6 | 1.78 | 0.016 |
| EFCAB12 | EF-hand calcium binding domain 12 | 1.77 | 0.038 |
| COL2A1 | collagen, type II, alpha 1 | 1.76 | 0.006 |
| HIST2H2BE | histone cluster 2, H2be | 1.76 | 0.040 |
| STC1 | stanniocalcin 1 | 1.76 | 0.035 |
| SMG1P5 | smg-1 homolog, phosphatidylinositol 3-kinase-related kinase (C. elegans) pseudogene | 1.75 | 0.017 |
| ASB14 | ankyrin repeat and SOCS box containing 14 | 1.75 | 0.034 |
| SCGB2A1 | secretoglobin, family 2A, member 1 | 1.75 | 0.014 |
| PDK1 | pyruvate dehydrogenase kinase, isozyme 1 | 1.74 | 0.0003 |
| ATP6V1B1 | ATPase, H+ transporting, lysosomal 56/58kDa, V1 subunit B1 | 1.73 | 0.035 |
| HSPA5 | heat shock 70kDa protein 5 (glucose-regulated protein, 78kDa) | 1.72 | 0.037 |
| APITD1 | apoptosis-inducing, TAF9-like domain 1 | 1.72 | 0.001 |
| CCDC150 | coiled-coil domain containing 150 | 1.72 | 0.009 |
| CDH17 | cadherin 17, LI cadherin (liver-intestine) | 1.72 | 0.035 |
| FAM171B | family with sequence similarity 171, member B | 1.71 | 0.004 |
| TRPC1 | transient receptor potential cation channel, subfamily C, member 1 | 1.70 | 0.030 |
| SDCBP2-AS1 | SDCBP2 antisense RNA 1 | 1.70 | 0.028 |
| MPP6 | membrane protein, palmitoylated 6 (MAGUK p55 subfamily member 6) | 1.70 | 0.038 |
| NCOA2 | nuclear receptor coactivator 2 | 1.70 | 0.003 |
| PLOD2 | procollagen-lysine, 2-oxoglutarate 5-dioxygenase 2 | 1.70 | 0.007 |
| LINC00472 | long intergenic non-protein coding RNA 472 | 1.69 | 0.030 |
| UBR1 | ubiquitin protein ligase E3 component n-recognin 1 | 1.69 | 0.049 |
| SPTBN4 | spectrin, beta, non-erythrocytic 4 | 1.68 | 0.006 |
| USP27X | ubiquitin specific peptidase 27, X-linked | 1.68 | 0.046 |
| ZNF81 | zinc finger protein 81 | 1.68 | 0.028 |
| C15orf38 | chromosome 15 open reading frame 38 | 1.67 | 0.012 |
| KIAA1715 | KIAA1715 | 1.66 | 0.002 |
| PIH1D2 | PIH1 domain containing 2 | 1.66 | 0.019 |
| CEP78 | centrosomal protein 78kDa | 1.66 | 0.038 |
| RAP2A | RAP2A, member of RAS oncogene family | 1.65 | 0.016 |
| ZNF585A | zinc finger protein 585A | 1.65 | 0.021 |
| WLS | wntless homolog (Drosophila) | 1.65 | 0.014 |
| PTGS1 | prostaglandin-endoperoxide synthase 1 (prostaglandin G/H synthase and cyclooxygenase) | 1.65 | 0.010 |
| LOX | lysyl oxidase | 1.65 | 0.006 |
| NDRG1 | N-myc downstream regulated 1 | 1.65 | 0.022 |
| C2orf88 | chromosome 2 open reading frame 88 | 1.64 | 0.001 |
| SHPRH | SNF2 histone linker PHD RING helicase, E3 ubiquitin protein ligase | 1.64 | 0.037 |
| LINC00888 | uncharacterized LOC100505687 | 1.64 | 0.045 |
| ARL5B | ADP-ribosylation factor-like 5B | 1.64 | 0.043 |
| RNASE4 | ribonuclease, RNase A family, 4 | 1.64 | 0.007 |
| DOCK10 | dedicator of cytokinesis 10 | 1.63 | 0.038 |
| LINC00847 | long intergenic non-protein coding RNA 847 | 1.62 | 0.046 |
| FLJ12825 | uncharacterized LOC440101 | 1.62 | 0.026 |
| C15orf41 | chromosome 15 open reading frame 41 | 1.62 | 0.028 |
| RAD50 | RAD50 homolog (S. cerevisiae) | 1.61 | 0.044 |
| STAC | SH3 and cysteine rich domain | 1.60 | 0.034 |
| GBE1 | glucan (1,4-alpha-), branching enzyme 1 | 1.60 | 0.0005 |
| SH3TC2 | SH3 domain and tetratricopeptide repeats 2 | 1.60 | 0.020 |
| CDCA2 | cell division cycle associated 2 | 1.60 | 0.040 |
| HSD17B12 | hydroxysteroid (17-beta) dehydrogenase 12 | 1.59 | 0.017 |
| ZNF77 | zinc finger protein 77 | 1.59 | 0.030 |
| TRIM23 | tripartite motif containing 23 | 1.59 | 0.045 |
| GOLGA1 | golgin A1 | 1.58 | 0.014 |
| CCDC69 | coiled-coil domain containing 69 | 1.58 | 0.042 |
| PMS2P5 | postmeiotic segregation increased 2 pseudogene 5 | 1.58 | 0.027 |
| DNAJC18 | DnaJ (Hsp40) homolog, subfamily C, member 18 | 1.58 | 0.025 |
| HERC2P7 | hect domain and RLD 2 pseudogene 7 | 1.58 | 0.048 |
| CHML | choroideremia-like (Rab escort protein 2) | 1.58 | 0.031 |
| SLC14A1 | solute carrier family 14 (urea transporter), member 1 (Kidd blood group) | 1.58 | 0.049 |
| HOMER1 | homer homolog 1 (Drosophila) | 1.57 | 0.014 |
| C4orf46 | chromosome 4 open reading frame 46 | 1.57 | 0.007 |
| ZNF32-AS2 | ZNF32 antisense RNA 2 | 1.57 | 0.023 |
| ERO1LB | ERO1-like beta (S. cerevisiae) | 1.57 | 0.002 |
| TMEM64 | transmembrane protein 64 | 1.56 | 0.048 |
| MOB3B | MOB kinase activator 3B | 1.56 | 0.016 |
| SLC22A15 | solute carrier family 22, member 15 | 1.56 | 0.023 |
| ORC5 | origin recognition complex, subunit 5 | 1.55 | 0.033 |
| WDR5B | WD repeat domain 5B | 1.55 | 0.042 |
| LOC100133985 | uncharacterized LOC100133985 | 1.55 | 0.020 |
| UPRT | uracil phosphoribosyltransferase (FUR1) homolog (S. cerevisiae) | 1.54 | 0.042 |
| PEX3 | peroxisomal biogenesis factor 3 | 1.54 | 0.049 |
| ZBTB24 | zinc finger and BTB domain containing 24 | 1.54 | 0.025 |
| BHLHB9 | basic helix-loop-helix domain containing, class B, 9 | 1.54 | 0.027 |
| CAMK4 | calcium/calmodulin-dependent protein kinase IV | 1.54 | 0.031 |
| C1orf109 | chromosome 1 open reading frame 109 | 1.53 | 0.045 |
| NUCB2 | nucleobindin 2 | 1.53 | 0.043 |
| ZBTB25 | zinc finger and BTB domain containing 25 | 1.53 | 0.019 |
| DNAJB9 | DnaJ (Hsp40) homolog, subfamily B, member 9 | 1.51 | 0.027 |
| DCK | deoxycytidine kinase | 1.51 | 0.039 |
| PPP2R5E | protein phosphatase 2, regulatory subunit B', epsilon isoform | 1.51 | 0.041 |
| TROAP | trophinin associated protein | 1.51 | 0.046 |
| RBM44 | RNA binding motif protein 44 | 1.51 | 0.049 |
| SNAPC5 | small nuclear RNA activating complex, polypeptide 5, 19kDa | 1.51 | 0.005 |
| PFKFB4 | 6-phosphofructo-2-kinase/fructose-2,6-biphosphatase 4 | 1.50 | 0.022 |
| ULBP1 | UL16 binding protein 1 | 1.50 | 0.043 |
| ULBP2 | UL16 binding protein 2 | 1.50 | 0.005 |
| CPA6 | carboxypeptidase A6 | 1.50 | 0.032 |
| MSL2 | male-specific lethal 2 homolog (Drosophila) | 1.50 | 0.022 |
| RNA5SP244 | RNA, 5S ribosomal pseudogene 244 | 0.05 | 0.037 |
| LOC100507351 | uncharacterized LOC100507351 | 0.11 | 0.015 |
| CCDC81 | coiled-coil domain containing 81 | 0.12 | 0.009 |
| STKLD1 | chromosome 9 open reading frame 96 | 0.13 | 0.006 |
| DKFZp686K1684 | uncharacterized LOC440034 | 0.16 | 0.001 |
| XCL1 | chemokine (C motif) ligand 1 | 0.18 | 0.017 |
| STMN4 | stathmin-like 4 | 0.20 | 0.020 |
| SRGAP2D | SLIT-ROBO Rho GTPase activating protein 2D | 0.20 | 0.039 |
| SLC47A1 | solute carrier family 47, member 1 | 0.28 | 0.038 |
| LINC00578 | long intergenic non-protein coding RNA 578 | 0.29 | 0.031 |
| TEDDM1 | transmembrane epididymal protein 1 | 0.30 | 0.041 |
| HYMAI | hydatidiform mole associated and imprinted (non-protein coding) | 0.30 | 0.034 |
| AKR7L | aldo-keto reductase family 7-like | 0.32 | 0.049 |
| FAM151B | family with sequence similarity 151, member B | 0.32 | 0.043 |
| GNGT2 | guanine nucleotide binding protein (G protein), gamma transducing activity polypeptide 2 | 0.32 | 0.035 |
| NLRP8 | NLR family, pyrin domain containing 8 | 0.34 | 0.012 |
| EMCN | endomucin | 0.34 | 0.019 |
| SIX2 | SIX homeobox 2 | 0.35 | 0.003 |
| SIGLEC14 | sialic acid binding Ig-like lectin 14 | 0.35 | 0.036 |
| SCML2 | sex comb on midleg-like 2 (Drosophila) | 0.39 | 0.013 |
| GPR157 | G protein-coupled receptor 157 | 0.39 | 0.003 |
| LINC00852 | long intergenic non-protein coding RNA 852 | 0.40 | 0.043 |
| MCF2L2 | MCF.2 cell line derived transforming sequence-like 2 | 0.40 | 0.027 |
| LOC100506469 | uncharacterized LOC100506469 | 0.41 | 0.033 |
| IL11 | interleukin 11 | 0.42 | 0.042 |
| LOC286177 | uncharacterized LOC286177 | 0.43 | 0.014 |
| ACSL6 | acyl-CoA synthetase long-chain family member 6 | 0.44 | 0.043 |
| NPAP1 | nuclear pore associated protein 1 | 0.44 | 0.021 |
| SCARF2 | scavenger receptor class F, member 2 | 0.45 | 0.026 |
| LOC646999 | akirin 1 pseudogene | 0.45 | 0.038 |
| PROB1 | proline-rich basic protein 1 | 0.45 | 0.005 |
| TSC22D1-AS1 | TSC22D1 antisense RNA 1 | 0.46 | 0.036 |
| FKBP1AP1 | FK506 binding protein 1A, 12kDa pseudogene 1 | 0.47 | 0.05 |
| LOC283683 | uncharacterized LOC283683 | 0.48 | 0.015 |
| IRAK3 | interleukin-1 receptor-associated kinase 3 | 0.48 | 0.003 |
| VTRNA1-3 | vault RNA 1-3 | 0.48 | 0.041 |
| KANK4 | KN motif and ankyrin repeat domains 4 | 0.49 | 0.038 |
| PART1 | prostate androgen-regulated transcript 1 (non-protein coding) | 0.49 | 0.048 |
| KCNH6 | potassium voltage-gated channel, subfamily H (eag-related), member 6 | 0.50 | 0.026 |
| LIMS2 | LIM and senescent cell antigen-like domains 2 | 0.50 | 0.031 |
| LINC00907 | uncharacterized LOC284260 | 0.50 | 0.028 |
| ADCY1 | adenylate cyclase 1 (brain) | 0.51 | 0.019 |
| SOX7 | SRY (sex determining region Y)-box 7 | 0.52 | 0.045 |
| CELF2 | CUGBP, Elav-like family member 2 | 0.53 | 0.020 |
| PBOV1 | prostate and breast cancer overexpressed 1 | 0.54 | 0.035 |
| RFPL1S | RFPL1 antisense RNA 1 | 0.54 | 0.045 |
| ZNF850 | zinc finger protein 850 | 0.54 | 0.05 |
| SLC25A53 | solute carrier family 25, member 53 | 0.55 | 0.035 |
| ZNF665 | zinc finger protein 665 | 0.56 | 0.008 |
| SP6 | Sp6 transcription factor | 0.56 | 0.037 |
| NME1-NME2 | NME1-NME2 readthrough | 0.56 | 0.003 |
| LOC100288181 | uncharacterized LOC100288181 | 0.57 | 0.017 |
| KIAA0087 | KIAA0087 | 0.57 | 0.0011 |
| GVINP1 | GTPase, very large interferon inducible pseudogene 1 | 0.57 | <0.0001 |
| NWD1 | NACHT and WD repeat domain containing 1 | 0.57 | 0.019 |
| BREA2 | breast cancer estrogen-induced apoptosis 2 | 0.57 | 0.026 |
| LINC01089 | uncharacterized LOC338799 | 0.58 | 0.019 |
| C1orf229 | chromosome 1 open reading frame 229 | 0.58 | 0.036 |
| MYOZ3 | myozenin 3 | 0.58 | 0.043 |
| PPP1R1A | protein phosphatase 1, regulatory (inhibitor) subunit 1A | 0.59 | 0.031 |
| A1BG-AS1 | A1BG antisense RNA 1 | 0.59 | 0.049 |
| NRN1L | neuritin 1-like | 0.59 | 0.031 |
| FAM153C | family with sequence similarity 153, member C, pseudogene | 0.59 | 0.035 |
| OTUD6A | OTU domain containing 6A | 0.59 | 0.034 |
| GPR63 | G protein-coupled receptor 63 | 0.59 | 0.014 |
| LOC100287225 | uncharacterized LOC100287225 | 0.59 | 0.021 |
| AUNIP | aurora kinase A and ninein interacting protein | 0.60 | 0.0002 |
| VWCE | von Willebrand factor C and EGF domains | 0.60 | 0.020 |
| ZBTB20 | zinc finger and BTB domain containing 20 | 0.60 | 0.017 |
| BIN3-IT1 | uncharacterized LOC80094 | 0.60 | 0.026 |
| LOC100289019 | uncharacterized LOC100289019 | 0.60 | 0.010 |
| GPLD1 | glycosylphosphatidylinositol specific phospholipase D1 | 0.60 | 0.014 |
| SLC35F1 | solute carrier family 35, member F1 | 0.61 | 0.030 |
| NAPSA | napsin A aspartic peptidase | 0.61 | <0.0001 |
| PCYT1B | phosphate cytidylyltransferase 1, choline, beta | 0.62 | 0.025 |
| POU2F2 | POU class 2 homeobox 2 | 0.62 | 0.022 |
| PRDM7 | PR domain containing 7 | 0.63 | 0.007 |
| C9orf172 | chromosome 9 open reading frame 172 | 0.63 | 0.025 |
| POM121L10P | POM121 transmembrane nucleoporin-like 10, pseudogene | 0.64 | 0.021 |
| LOC284950 | uncharacterized LOC284950 | 0.64 | 0.006 |
| PRG2 | proteoglycan 2, bone marrow (natural killer cell activator, eosinophil granule major basic protein) | 0.64 | 0.046 |
| TRIM66 | tripartite motif containing 66 | 0.64 | 0.023 |
| KCNH3 | potassium voltage-gated channel, subfamily H (eag-related), member 3 | 0.64 | 0.048 |
| TRIM58 | tripartite motif containing 58 | 0.64 | 0.038 |
| NEK5 | NIMA-related kinase 5 | 0.65 | 0.008 |
| ZNF341 | zinc finger protein 341 | 0.65 | 0.005 |
| PPM1K | protein phosphatase, Mg2+/Mn2+ dependent, 1K | 0.65 | 0.039 |
| TVP23C | trans-golgi network vesicle protein 23 homolog C (S. cerevisiae) | 0.65 | 0.018 |
| RPGR | retinitis pigmentosa GTPase regulator | 0.65 | 0.023 |
| LOC440600 | uncharacterized LOC440600 | 0.65 | 0.048 |
| ADORA2A | adenosine A2a receptor | 0.65 | 0.003 |
| LINC00598 | long intergenic non-protein coding RNA 598 | 0.65 | 0.049 |
| CXorf36 | chromosome X open reading frame 36 | 0.65 | 0.039 |
| IQCD | IQ motif containing D | 0.65 | 0.048 |
| COLCA1 | chromosome 11 open reading frame 92 | 0.65 | 0.014 |
| EFNB3 | ephrin-B3 | 0.66 | 0.046 |
| PPM1J | protein phosphatase, Mg2+/Mn2+ dependent, 1J | 0.66 | 0.025 |
| IGF1 | insulin-like growth factor 1 (somatomedin C) | 0.67 | 0.013 |
| C8orf86 | chromosome 8 open reading frame 86 | 0.67 | 0.030 |
