# Supplementary material for: Human mesenchymal stromal cells broadly modulate high glucose-induced inflammatory responses of renal proximal tubular cell monolayers
Source: Stem Cell Res Ther. 2019 Nov 19;10:329. doi: 10.1186/s13287-019-1424-5 (PMC6862760; doi:10.1186/s13287-019-1424-5)
Supplement: Supplementary file 14 — Additional file 14: Table S5. List of DEGs with significant Fold Changes in RPTEC/TERT1 cells without and with MSC co-culture in high glucose (HG) condition. [file 13287_2019_1424_MOESM14_ESM.docx]

| **Supplementary Table S5: List of DEGs with significant Fold Changes in RPTEC/TERT1 cells without and with MSC co-culture in high glucose (HG) condition** | | | |
| --- | --- | --- | --- |
|  | Higher expression in No-MSC versus MSC co-culture (HG) |  |  |
|  | Lower expression in No-MSC versus MSC co-culture (HG) |  |  |
|  |  |  |  |
| **Gene Symbol** | **Gene Description** | **Fold change No-MSC vs MSC** | **P Value** |
| DLX1 | distal-less homeobox 1 | 14.00 | 0.0015 |
| TEX11 | testis expressed 11 | 9.67 | 0.035 |
| GRK7 | G protein-coupled receptor kinase 7 | 8.67 | 0.0019 |
| FCAMR | Fc receptor, IgA, IgM, high affinity | 7.67 | 0.05 |
| GATM | glycine amidinotransferase (L-arginine:glycine amidinotransferase) | 6.75 | 0.007 |
| ALS2CR12 | amyotrophic lateral sclerosis 2 (juvenile) chromosome region, candidate 12 | 6.60 | 0.023 |
| SCARNA27 | small Cajal body-specific RNA 27 | 6.34 | 0.040 |
| SMAD5-AS1 | SMAD5 antisense RNA 1 | 6.33 | 0.026 |
| AOX1 | aldehyde oxidase 1 | 6.17 | 0.028 |
| HIST1H2AG | histone cluster 1, H2ag | 6.10 | 0.012 |
| DGAT2 | diacylglycerol O-acyltransferase 2 | 5.93 | 0.05 |
| LINC00525 | long intergenic non-protein coding RNA 525 | 5.80 | 0.026 |
| MAMDC2-AS1 | uncharacterized LOC100507244 | 5.67 | 0.020 |
| PHGDH | phosphoglycerate dehydrogenase | 5.46 | 0.005 |
| NPPA | natriuretic peptide A | 5.18 | 0.003 |
| DYX1C1-CCPG1 | DYX1C1-CCPG1 readthrough (non-protein coding) | 4.98 | 0.002 |
| SYT1 | synaptotagmin I | 4.83 | 0.009 |
| SNORD18C | small nucleolar RNA, C/D box 18C | 4.79 | 0.006 |
| FGF1 | fibroblast growth factor 1 (acidic) | 4.50 | 0.020 |
| BAALC | brain and acute leukemia, cytoplasmic | 4.50 | 0.020 |
| CCDC169 | coiled-coil domain containing 169 | 4.44 | 0.013 |
| MIR4767 | microRNA 4767 | 4.25 | 0.05 |
| ISM1 | isthmin 1 homolog (zebrafish) | 4.13 | 0.049 |
| TAS2R3 | taste receptor, type 2, member 3 | 4.08 | 0.036 |
| IL19 | interleukin 19 | 4.05 | 0.022 |
| PHEX | phosphate regulating endopeptidase homolog, X-linked | 4.00 | 0.020 |
| REP15 | RAB15 effector protein | 4.00 | 0.027 |
| LOC646938 | TBC1 domain family, member 2B pseudogene | 3.91 | 0.047 |
| RAPSN | receptor-associated protein of the synapse | 3.75 | 0.032 |
| LOC100506655 | uncharacterized LOC100506655 | 3.75 | 0.05 |
| KRTAP5-9 | keratin associated protein 5-9 | 3.75 | 0.05 |
| SELL | selectin L | 3.75 | 0.008 |
| TRPM3 | transient receptor potential cation channel, subfamily M, member 3 | 3.74 | 0.026 |
| CST2 | cystatin SA | 3.69 | 0.033 |
| KRTDAP | keratinocyte differentiation-associated protein | 3.63 | 0.043 |
| RRN3P2 | RNA polymerase I transcription factor homolog (S. cerevisiae) pseudogene 2 | 3.63 | 0.044 |
| ABCA12 | ATP-binding cassette, sub-family A (ABC1), member 12 | 3.62 | 0.018 |
| C11orf82 | chromosome 11 open reading frame 82 | 3.25 | 0.008 |
| HEY1 | hairy/enhancer-of-split related with YRPW motif 1 | 3.17 | 0.021 |
| IGDCC4 | immunoglobulin superfamily, DCC subclass, member 4 | 3.17 | 0.019 |
| TSACC | TSSK6 activating co-chaperone | 3.05 | 0.037 |
| CHAC1 | ChaC, cation transport regulator homolog 1 (E. coli) | 3.05 | 0.009 |
| FLJ22447 | uncharacterized LOC400221 | 3.03 | 0.012 |
| IL1B | interleukin 1, beta | 2.93 | 0.005 |
| LCN2 | lipocalin 2 | 2.91 | 0.016 |
| PLEKHS1 | pleckstrin homology domain containing, family S member 1 | 2.88 | 0.05 |
| Mar-03 | membrane-associated ring finger (C3HC4) 3, E3 ubiquitin protein ligase | 2.83 | 0.032 |
| EXO1 | exonuclease 1 | 2.81 | 0.015 |
| ZBED3-AS1 | ZBED3 antisense RNA 1 | 2.80 | 0.005 |
| HIST1H2BG | histone cluster 1, H2bg | 2.79 | 0.05 |
| C17orf51 | chromosome 17 open reading frame 51 | 2.73 | 0.019 |
| PCDHB3 | protocadherin beta 3 | 2.67 | 0.038 |
| MMP10 | matrix metallopeptidase 10 (stromelysin 2) | 2.67 | 0.009 |
| CCL2 | chemokine (C-C motif) ligand 2 | 2.64 | 0.039 |
| SPTBN5 | spectrin, beta, non-erythrocytic 5 | 2.63 | 0.040 |
| BLM | Bloom syndrome, RecQ helicase-like | 2.63 | 0.020 |
| FAM111B | family with sequence similarity 111, member B | 2.61 | 0.0019 |
| CECR7 | cat eye syndrome chromosome region, candidate 7 (non-protein coding) | 2.58 | 0.041 |
| FST | follistatin | 2.57 | 0.024 |
| PTCD1 | pentatricopeptide repeat domain 1 | 2.56 | 0.011 |
| ESCO2 | establishment of cohesion 1 homolog 2 (S. cerevisiae) | 2.54 | 0.009 |
| CXCL2 | chemokine (C-X-C motif) ligand 2 | 2.53 | 0.042 |
| SKA3 | spindle and kinetochore associated complex subunit 3 | 2.51 | 0.023 |
| THSD7B | thrombospondin, type I, domain containing 7B | 2.50 | 0.012 |
| SCUBE3 | signal peptide, CUB domain, EGF-like 3 | 2.50 | 0.035 |
| CENPI | centromere protein I | 2.49 | 0.020 |
| CDC45 | cell division cycle 45 | 2.44 | 0.013 |
| LINC00634 | long intergenic non-protein coding RNA 634 | 2.43 | 0.032 |
| RFTN1 | raftlin, lipid raft linker 1 | 2.43 | 0.05 |
| MIR181A1HG | familial acute myelogenous leukemia related factor | 2.42 | 0.0010 |
| ZNF367 | zinc finger protein 367 | 2.41 | 0.031 |
| SNORD76 | small nucleolar RNA, C/D box 76 | 2.40 | 0.039 |
| NUDT9P1 | nudix (nucleoside diphosphate linked moiety X)-type motif 9 pseudogene 1 | 2.38 | 0.009 |
| HRK | harakiri, BCL2 interacting protein (contains only BH3 domain) | 2.38 | 0.041 |
| TREX2 | three prime repair exonuclease 2 | 2.38 | 0.05 |
| FAIM3 | Fas apoptotic inhibitory molecule 3 | 2.36 | 0.031 |
| MCM10 | minichromosome maintenance complex component 10 | 2.35 | 0.0005 |
| GINS2 | GINS complex subunit 2 (Psf2 homolog) | 2.33 | 0.004 |
| IL18R1 | interleukin 18 receptor 1 | 2.33 | 0.022 |
| LINC00626 | long intergenic non-protein coding RNA 626 | 2.30 | 0.0011 |
| NOP56 | NOP56 ribonucleoprotein | 2.29 | 0.045 |
| SP5 | Sp5 transcription factor | 2.28 | 0.020 |
| HIST2H3D | histone cluster 2, H3d | 2.27 | 0.036 |
| E2F1 | E2F transcription factor 1 | 2.23 | 0.007 |
| ZWINT | ZW10 interactor, kinetochore protein | 2.23 | 0.040 |
| CDC6 | cell division cycle 6 | 2.22 | 0.013 |
| MKI67 | antigen identified by monoclonal antibody Ki-67 | 2.22 | 0.007 |
| BUB1B | BUB1 mitotic checkpoint serine/threonine kinase B | 2.22 | 0.008 |
| FAM71F2 | family with sequence similarity 71, member F2 | 2.21 | 0.031 |
| MYBL2 | v-myb myeloblastosis viral oncogene homolog (avian)-like 2 | 2.21 | 0.010 |
| CENPF | centromere protein F, 350/400kDa | 2.18 | 0.019 |
| ASF1B | ASF1 anti-silencing function 1 homolog B (S. cerevisiae) | 2.16 | 0.007 |
| ACTBL2 | actin, beta-like 2 | 2.13 | 0.0016 |
| ASPM | asp (abnormal spindle) homolog, microcephaly associated (Drosophila) | 2.11 | 0.013 |
| KIF26B | kinesin family member 26B | 2.11 | 0.022 |
| CDCA2 | cell division cycle associated 2 | 2.11 | 0.012 |
| CDH5 | cadherin 5, type 2 (vascular endothelium) | 2.10 | 0.032 |
| RAPGEFL1 | Rap guanine nucleotide exchange factor (GEF)-like 1 | 2.09 | 0.049 |
| CDCA5 | cell division cycle associated 5 | 2.09 | 0.010 |
| ASNS | asparagine synthetase (glutamine-hydrolyzing) | 2.09 | 0.023 |
| C16orf59 | chromosome 16 open reading frame 59 | 2.09 | 0.014 |
| PBK | PDZ binding kinase | 2.08 | 0.021 |
| SLC1A4 | solute carrier family 1 (glutamate/neutral amino acid transporter), member 4 | 2.06 | 0.028 |
| KIF15 | kinesin family member 15 | 2.06 | 0.007 |
| DNER | delta/notch-like EGF repeat containing | 2.05 | 0.038 |
| PINX1 | PIN2/TERF1 interacting, telomerase inhibitor 1 | 2.04 | 0.028 |
| DTL | denticleless E3 ubiquitin protein ligase homolog (Drosophila) | 2.04 | 0.013 |
| CENPM | centromere protein M | 2.02 | 0.020 |
| NUF2 | NUF2, NDC80 kinetochore complex component, homolog (S. cerevisiae) | 2.00 | 0.018 |
| SERPINB7 | serpin peptidase inhibitor, clade B (ovalbumin), member 7 | 2.00 | 0.004 |
| NOP2 | NOP2 nucleolar protein | 2.00 | 0.037 |
| CCNE2 | cyclin E2 | 1.99 | 0.0006 |
| CHAC2 | ChaC, cation transport regulator homolog 2 (E. coli) | 1.98 | 0.012 |
| CXCL5 | chemokine (C-X-C motif) ligand 5 | 1.97 | 0.012 |
| GTSE1 | G-2 and S-phase expressed 1 | 1.96 | 0.0014 |
| CENPW | centromere protein W | 1.96 | 0.029 |
| HMMR | hyaluronan-mediated motility receptor (RHAMM) | 1.95 | 0.041 |
| VNN1 | vanin 1 | 1.95 | 0.0012 |
| PSAT1 | phosphoserine aminotransferase 1 | 1.94 | 0.038 |
| RFC3 | replication factor C (activator 1) 3, 38kDa | 1.94 | 0.021 |
| LOC152225 | uncharacterized LOC152225 | 1.94 | 0.026 |
| CDCA3 | cell division cycle associated 3 | 1.93 | 0.005 |
| CENPA | centromere protein A | 1.93 | 0.006 |
| CKAP2L | cytoskeleton associated protein 2-like | 1.93 | 0.007 |
| SLC6A9 | solute carrier family 6 (neurotransmitter transporter, glycine), member 9 | 1.92 | 0.017 |
| FEN1 | flap structure-specific endonuclease 1 | 1.92 | 0.012 |
| FNTB | farnesyltransferase, CAAX box, beta | 1.91 | 0.023 |
| KYNU | kynureninase | 1.91 | 0.049 |
| NKX3-1 | NK3 homeobox 1 | 1.91 | 0.004 |
| LOC554206 | leucine carboxyl methyltransferase 1 pseudogene | 1.91 | 0.044 |
| IL23A | interleukin 23, alpha subunit p19 | 1.91 | 0.036 |
| HBQ1 | hemoglobin, theta 1 | 1.91 | 0.05 |
| KIF2C | kinesin family member 2C | 1.90 | 0.007 |
| SP140 | SP140 nuclear body protein | 1.89 | 0.05 |
| AURKB | aurora kinase B | 1.88 | 0.0013 |
| DSCC1 | defective in sister chromatid cohesion 1 homolog (S. cerevisiae) | 1.88 | 0.013 |
| NCAPH | non-SMC condensin I complex, subunit H | 1.88 | 0.002 |
| MYBL1 | v-myb myeloblastosis viral oncogene homolog (avian)-like 1 | 1.88 | 0.017 |
| CEP55 | centrosomal protein 55kDa | 1.88 | 0.006 |
| DIAPH3 | diaphanous homolog 3 (Drosophila) | 1.87 | 0.0004 |
| FAM83D | family with sequence similarity 83, member D | 1.87 | 0.002 |
| CDC25C | cell division cycle 25C | 1.87 | 0.039 |
| TOP2A | topoisomerase (DNA) II alpha 170kDa | 1.87 | 0.020 |
| PLK1 | polo-like kinase 1 | 1.86 | 0.0004 |
| IFI16 | interferon, gamma-inducible protein 16 | 1.86 | 0.019 |
| MTHFD2 | methylenetetrahydrofolate dehydrogenase (NADP+ dependent) 2, methenyltetrahydrofolate cyclohydrolase | 1.86 | 0.033 |
| NEIL3 | nei endonuclease VIII-like 3 (E. coli) | 1.85 | 0.05 |
| MCM4 | minichromosome maintenance complex component 4 | 1.85 | 0.022 |
| CCM2L | cerebral cavernous malformation 2-like | 1.85 | 0.014 |
| BIRC5 | baculoviral IAP repeat containing 5 | 1.84 | 0.004 |
| KIF23 | kinesin family member 23 | 1.84 | 0.0018 |
| KIFC1 | kinesin family member C1 | 1.84 | 0.006 |
| URB2 | URB2 ribosome biogenesis 2 homolog (S. cerevisiae) | 1.84 | 0.044 |
| STX11 | syntaxin 11 | 1.84 | 0.011 |
| TPX2 | TPX2, microtubule-associated, homolog (Xenopus laevis) | 1.83 | 0.003 |
| ZFP69B | ZFP69 zinc finger protein B | 1.83 | 0.035 |
| XDH | xanthine dehydrogenase | 1.83 | 0.025 |
| RRM2 | ribonucleotide reductase M2 | 1.83 | 0.020 |
| SNPH | syntaphilin | 1.82 | 0.047 |
| CCNA2 | cyclin A2 | 1.82 | 0.009 |
| SPAG5 | sperm associated antigen 5 | 1.82 | 0.012 |
| OAS3 | 2'-5'-oligoadenylate synthetase 3, 100kDa | 1.81 | 0.0008 |
| ANLN | anillin, actin binding protein | 1.81 | 0.025 |
| KIF20A | kinesin family member 20A | 1.81 | 0.012 |
| ZIC5 | Zic family member 5 | 1.81 | 0.023 |
| CSPG5 | chondroitin sulfate proteoglycan 5 (neuroglycan C) | 1.81 | 0.003 |
| SFMBT1 | Scm-like with four mbt domains 1 | 1.81 | 0.015 |
| PIGR | polymeric immunoglobulin receptor | 1.80 | 0.002 |
| DLGAP5 | discs, large (Drosophila) homolog-associated protein 5 | 1.80 | 0.042 |
| TNIP3 | TNFAIP3 interacting protein 3 | 1.80 | 0.005 |
| DEPDC1 | DEP domain containing 1 | 1.80 | 0.032 |
| CENPE | centromere protein E, 312kDa | 1.80 | 0.041 |
| NUSAP1 | nucleolar and spindle associated protein 1 | 1.80 | 0.010 |
| SHCBP1 | SHC SH2-domain binding protein 1 | 1.79 | 0.029 |
| GPATCH4 | G patch domain containing 4 | 1.79 | 0.044 |
| KIAA1524 | KIAA1524 | 1.79 | 0.008 |
| TRIM31 | tripartite motif containing 31 | 1.79 | 0.024 |
| AURKA | aurora kinase A | 1.79 | 0.0016 |
| SLC36A1 | solute carrier family 36 (proton/amino acid symporter), member 1 | 1.79 | 0.042 |
| PTGDR2 | prostaglandin D2 receptor 2 | 1.78 | 0.041 |
| HBB | hemoglobin, beta | 1.78 | 0.0012 |
| DHRS3 | dehydrogenase/reductase (SDR family) member 3 | 1.77 | 0.003 |
| CLEC4E | C-type lectin domain family 4, member E | 1.77 | 0.029 |
| CTH | cystathionase (cystathionine gamma-lyase) | 1.77 | 0.045 |
| KIF4A | kinesin family member 4A | 1.77 | 0.0009 |
| SH3RF3-AS1 | SH3RF3 antisense RNA 1 | 1.77 | 0.008 |
| E2F8 | E2F transcription factor 8 | 1.77 | 0.005 |
| FOXM1 | forkhead box M1 | 1.77 | 0.002 |
| PCNA | proliferating cell nuclear antigen | 1.76 | 0.014 |
| WDR62 | WD repeat domain 62 | 1.76 | 0.016 |
| C1orf112 | chromosome 1 open reading frame 112 | 1.76 | 0.023 |
| CDK1 | cyclin-dependent kinase 1 | 1.76 | 0.028 |
| STAB1 | stabilin 1 | 1.76 | 0.010 |
| CCDC15 | coiled-coil domain containing 15 | 1.75 | 0.046 |
| ALPK2 | alpha-kinase 2 | 1.75 | 0.014 |
| C10orf2 | chromosome 10 open reading frame 2 | 1.75 | 0.030 |
| PNO1 | partner of NOB1 homolog (S. cerevisiae) | 1.75 | 0.023 |
| MARS | methionyl-tRNA synthetase | 1.75 | 0.008 |
| CENPU | MLF1 interacting protein | 1.74 | 0.027 |
| TICRR | TOPBP1-interacting checkpoint and replication regulator | 1.74 | 0.005 |
| LY6G5C | lymphocyte antigen 6 complex, locus G5C | 1.74 | 0.015 |
| NCF2 | neutrophil cytosolic factor 2 | 1.73 | 0.005 |
| FAM169A | family with sequence similarity 169, member A | 1.72 | 0.049 |
| SYT16 | synaptotagmin XVI | 1.72 | 0.011 |
| KPNA2 | karyopherin alpha 2 (RAG cohort 1, importin alpha 1) | 1.72 | 0.011 |
| HAUS8 | HAUS augmin-like complex, subunit 8 | 1.72 | 0.011 |
| PODXL2 | podocalyxin-like 2 | 1.72 | 0.05 |
| CMSS1 | cms1 ribosomal small subunit homolog (yeast) | 1.72 | 0.007 |
| XPOT | exportin, tRNA | 1.72 | 0.035 |
| BYSL | bystin-like | 1.72 | 0.021 |
| LYAR | Ly1 antibody reactive | 1.72 | 0.0003 |
| TRIB3 | tribbles homolog 3 (Drosophila) | 1.71 | 0.05 |
| CNNM1 | cyclin M1 | 1.71 | 0.0013 |
| CIITA | class II, major histocompatibility complex, transactivator | 1.71 | 0.033 |
| HAUS7 | HAUS augmin-like complex, subunit 7 | 1.71 | 0.045 |
| FAM169B | family with sequence similarity 169, member B | 1.71 | 0.0009 |
| DDX21 | DEAD (Asp-Glu-Ala-Asp) box helicase 21 | 1.70 | 0.045 |
| KIF20B | kinesin family member 20B | 1.70 | 0.05 |
| CDCA8 | cell division cycle associated 8 | 1.70 | 0.009 |
| PTCHD3 | patched domain containing 3 | 1.69 | 0.042 |
| SLFN5 | schlafen family member 5 | 1.69 | 0.033 |
| ADAMTS3 | ADAM metallopeptidase with thrombospondin type 1 motif, 3 | 1.69 | 0.016 |
| TCF19 | transcription factor 19 | 1.69 | 0.016 |
| CEBPG | CCAAT/enhancer binding protein (C/EBP), gamma | 1.69 | 0.035 |
| FAM64A | family with sequence similarity 64, member A | 1.69 | 0.008 |
| NOLC1 | nucleolar and coiled-body phosphoprotein 1 | 1.68 | 0.028 |
| ZNF239 | zinc finger protein 239 | 1.68 | 0.018 |
| UTP14A | UTP14, U3 small nucleolar ribonucleoprotein, homolog A (yeast) | 1.68 | 0.027 |
| OGFRL1 | opioid growth factor receptor-like 1 | 1.68 | 0.016 |
| PAK1IP1 | PAK1 interacting protein 1 | 1.68 | 0.014 |
| PKMYT1 | protein kinase, membrane associated tyrosine/threonine 1 | 1.68 | 0.006 |
| ANTXR1 | anthrax toxin receptor 1 | 1.68 | 0.011 |
| CHRNA10 | cholinergic receptor, nicotinic, alpha 10 (neuronal) | 1.68 | 0.05 |
| FBXO5 | F-box protein 5 | 1.68 | 0.05 |
| RRS1 | RRS1 ribosome biogenesis regulator homolog (S. cerevisiae) | 1.67 | 0.021 |
| NR1D2 | nuclear receptor subfamily 1, group D, member 2 | 1.67 | 0.021 |
| SOD2 | superoxide dismutase 2, mitochondrial | 1.67 | 0.018 |
| IQCD | IQ motif containing D | 1.67 | 0.048 |
| COL12A1 | collagen, type XII, alpha 1 | 1.67 | 0.003 |
| NCAPG2 | non-SMC condensin II complex, subunit G2 | 1.67 | 0.025 |
| CDT1 | chromatin licensing and DNA replication factor 1 | 1.67 | 0.0016 |
| UBE2C | ubiquitin-conjugating enzyme E2C | 1.66 | 0.022 |
| MCM3 | minichromosome maintenance complex component 3 | 1.66 | 0.006 |
| TUBA1B | tubulin, alpha 1b | 1.66 | 0.012 |
| FUT8-AS1 | FUT8 antisense RNA 1 | 1.66 | 0.05 |
| MARS2 | methionyl-tRNA synthetase 2, mitochondrial | 1.66 | 0.037 |
| PTPRB | protein tyrosine phosphatase, receptor type, B | 1.66 | 0.0018 |
| PRKCH | protein kinase C, eta | 1.66 | 0.014 |
| DNAJC6 | DnaJ (Hsp40) homolog, subfamily C, member 6 | 1.66 | 0.030 |
| PRC1 | protein regulator of cytokinesis 1 | 1.66 | 0.0008 |
| MTFR2 | mitochondrial fission regulator 2 | 1.66 | 0.008 |
| ELOVL3 | ELOVL fatty acid elongase 3 | 1.66 | 0.007 |
| GTPBP4 | GTP binding protein 4 | 1.66 | 0.031 |
| TACC3 | transforming, acidic coiled-coil containing protein 3 | 1.66 | 0.0004 |
| TNFRSF11B | tumor necrosis factor receptor superfamily, member 11b | 1.66 | 0.0008 |
| TRIM6 | tripartite motif containing 6 | 1.65 | 0.011 |
| ATF5 | activating transcription factor 5 | 1.65 | 0.038 |
| CENPJ | centromere protein J | 1.65 | 0.023 |
| VTI1A | vesicle transport through interaction with t-SNAREs 1A | 1.65 | 0.016 |
| CDC20 | cell division cycle 20 | 1.64 | 0.003 |
| WDR3 | WD repeat domain 3 | 1.64 | 0.046 |
| C3 | complement component 3 | 1.64 | 0.025 |
| FANCI | Fanconi anemia, complementation group I | 1.64 | 0.017 |
| IARS | isoleucyl-tRNA synthetase | 1.64 | 0.036 |
| AIMP2 | aminoacyl tRNA synthetase complex-interacting multifunctional protein 2 | 1.64 | 0.032 |
| FICD | FIC domain containing | 1.63 | 0.035 |
| PIF1 | PIF1 5'-to-3' DNA helicase homolog (S. cerevisiae) | 1.63 | 0.033 |
| CCNB1 | cyclin B1 | 1.63 | 0.034 |
| HJURP | Holliday junction recognition protein | 1.63 | 0.011 |
| ARHGDIB | Rho GDP dissociation inhibitor (GDI) beta | 1.63 | 0.008 |
| NLRC4 | NLR family, CARD domain containing 4 | 1.63 | 0.05 |
| CDH4 | cadherin 4, type 1, R-cadherin (retinal) | 1.62 | 0.012 |
| SPAG1 | sperm associated antigen 1 | 1.62 | 0.008 |
| TERT | telomerase reverse transcriptase | 1.62 | 0.002 |
| H2AFX | H2A histone family, member X | 1.62 | 0.022 |
| NEXN | nexilin (F actin binding protein) | 1.62 | 0.042 |
| PRKAR1B | protein kinase, cAMP-dependent, regulatory, type I, beta | 1.62 | 0.047 |
| ORC5 | origin recognition complex, subunit 5 | 1.62 | 0.042 |
| RAD51 | RAD51 homolog (S. cerevisiae) | 1.62 | 0.047 |
| ZNF678 | zinc finger protein 678 | 1.61 | 0.008 |
| LTV1 | LTV1 homolog (S. cerevisiae) | 1.61 | 0.003 |
| KNSTRN | kinetochore-localized astrin/SPAG5 binding protein | 1.61 | 0.003 |
| KIF18A | kinesin family member 18A | 1.61 | 0.026 |
| ZWILCH | zwilch kinetochore protein | 1.61 | 0.010 |
| LARS | leucyl-tRNA synthetase | 1.61 | 0.034 |
| SLC22A18AS | solute carrier family 22 (organic cation transporter), member 18 antisense | 1.61 | 0.037 |
| CHORDC1 | cysteine and histidine-rich domain (CHORD) containing 1 | 1.61 | 0.038 |
| MON1A | MON1 homolog A (yeast) | 1.61 | 0.042 |
| NUP35 | nucleoporin 35kDa | 1.60 | 0.012 |
| ZNF319 | zinc finger protein 319 | 1.60 | 0.013 |
| MAK16 | MAK16 homolog (S. cerevisiae) | 1.60 | 0.018 |
| DHFR | dihydrofolate reductase | 1.60 | 0.036 |
| RPP25 | ribonuclease P/MRP 25kDa subunit | 1.60 | 0.026 |
| IDI2-AS1 | IDI2 antisense RNA 1 | 1.60 | 0.035 |
| UTP20 | UTP20, small subunit (SSU) processome component, homolog (yeast) | 1.60 | 0.007 |
| TARS | threonyl-tRNA synthetase | 1.59 | 0.011 |
| HLA-DRA | major histocompatibility complex, class II, DR alpha | 1.59 | 0.011 |
| UHRF1 | ubiquitin-like with PHD and ring finger domains 1 | 1.59 | 0.0012 |
| FAM208B | family with sequence similarity 208, member B | 1.59 | 0.036 |
| TRIP13 | thyroid hormone receptor interactor 13 | 1.58 | 0.007 |
| TEX10 | testis expressed 10 | 1.58 | 0.026 |
| KLHDC7A | kelch domain containing 7A | 1.58 | 0.007 |
| POLA2 | polymerase (DNA directed), alpha 2, accessory subunit | 1.58 | 0.023 |
| BICC1 | bicaudal C homolog 1 (Drosophila) | 1.58 | 0.009 |
| POLR3B | polymerase (RNA) III (DNA directed) polypeptide B | 1.58 | 0.002 |
| SPRTN | SprT-like N-terminal domain | 1.58 | 0.036 |
| LYPD6B | LY6/PLAUR domain containing 6B | 1.58 | 0.017 |
| LRRIQ1 | leucine-rich repeats and IQ motif containing 1 | 1.58 | 0.005 |
| SLC12A2 | solute carrier family 12 (sodium/potassium/chloride transporters), member 2 | 1.58 | 0.015 |
| PRIM2 | primase, DNA, polypeptide 2 (58kDa) | 1.58 | 0.030 |
| SUV39H1 | suppressor of variegation 3-9 homolog 1 (Drosophila) | 1.58 | 0.013 |
| CA2 | carbonic anhydrase II | 1.57 | 0.013 |
| CIRH1A | cirrhosis, autosomal recessive 1A (cirhin) | 1.57 | 0.032 |
| HLA-DRB1 | major histocompatibility complex, class II, DR beta 1 | 1.57 | 0.013 |
| PRKAR2B | protein kinase, cAMP-dependent, regulatory, type II, beta | 1.57 | 0.019 |
| CAPN13 | calpain 13 | 1.56 | 0.028 |
| CCDC18 | coiled-coil domain containing 18 | 1.56 | 0.028 |
| IQGAP3 | IQ motif containing GTPase activating protein 3 | 1.56 | 0.007 |
| COA7 | Sel1 repeat containing 1 | 1.56 | 0.039 |
| TUBA1C | tubulin, alpha 1c | 1.56 | 0.032 |
| SLC1A5 | solute carrier family 1 (neutral amino acid transporter), member 5 | 1.56 | 0.021 |
| POLR1A | polymerase (RNA) I polypeptide A, 194kDa | 1.56 | 0.014 |
| HEATR3 | HEAT repeat containing 3 | 1.56 | 0.040 |
| TNFSF15 | tumor necrosis factor (ligand) superfamily, member 15 | 1.56 | 0.031 |
| SPRN | shadow of prion protein homolog (zebrafish) | 1.56 | 0.005 |
| EXOSC8 | exosome component 8 | 1.56 | 0.010 |
| NCOA5 | nuclear receptor coactivator 5 | 1.56 | 0.032 |
| TYMS | thymidylate synthetase | 1.55 | 0.003 |
| AMER1 | APC membrane recruitment protein 1 | 1.55 | 0.003 |
| MCM2 | minichromosome maintenance complex component 2 | 1.55 | 0.044 |
| POLR1B | polymerase (RNA) I polypeptide B, 128kDa | 1.55 | 0.039 |
| PRKDC | protein kinase, DNA-activated, catalytic polypeptide | 1.55 | 0.039 |
| C4orf19 | chromosome 4 open reading frame 19 | 1.55 | 0.040 |
| WARS | tryptophanyl-tRNA synthetase | 1.55 | 0.027 |
| ESF1 | ESF1, nucleolar pre-rRNA processing protein, homolog (S. cerevisiae) | 1.55 | 0.007 |
| TNC | tenascin C | 1.55 | 0.0010 |
| RECQL4 | RecQ protein-like 4 | 1.55 | 0.016 |
| POLR3K | polymerase (RNA) III (DNA directed) polypeptide K, 12.3 kDa | 1.54 | 0.025 |
| NOP14 | NOP14 nucleolar protein | 1.54 | 0.010 |
| NCAPG | non-SMC condensin I complex, subunit G | 1.54 | 0.05 |
| GEMIN5 | gem (nuclear organelle) associated protein 5 | 1.54 | 0.016 |
| PLK2 | polo-like kinase 2 | 1.54 | 0.039 |
| PCK2 | phosphoenolpyruvate carboxykinase 2 (mitochondrial) | 1.54 | 0.017 |
| BRCC3 | BRCA1/BRCA2-containing complex, subunit 3 | 1.54 | 0.042 |
| RHCE | Rh blood group, CcEe antigens | 1.54 | 0.019 |
| PYCR1 | pyrroline-5-carboxylate reductase 1 | 1.54 | 0.035 |
| TOMM40 | translocase of outer mitochondrial membrane 40 homolog (yeast) | 1.54 | 0.021 |
| CCT5 | chaperonin containing TCP1, subunit 5 (epsilon) | 1.54 | 0.019 |
| ARHGAP31 | Rho GTPase activating protein 31 | 1.54 | 0.029 |
| UBIAD1 | UbiA prenyltransferase domain containing 1 | 1.54 | 0.011 |
| DNAJC9 | DnaJ (Hsp40) homolog, subfamily C, member 9 | 1.54 | 0.009 |
| SH2D4A | SH2 domain containing 4A | 1.54 | 0.006 |
| CLN6 | ceroid-lipofuscinosis, neuronal 6, late infantile, variant | 1.54 | 0.024 |
| LRR1 | leucine rich repeat protein 1 | 1.54 | 0.039 |
| NOP58 | NOP58 ribonucleoprotein | 1.53 | 0.018 |
| EBNA1BP2 | EBNA1 binding protein 2 | 1.53 | 0.0008 |
| GRHL1 | grainyhead-like 1 (Drosophila) | 1.53 | 0.038 |
| KIAA0020 | KIAA0020 | 1.53 | 0.007 |
| PMAIP1 | phorbol-12-myristate-13-acetate-induced protein 1 | 1.53 | 0.012 |
| ETV3 | ets variant 3 | 1.53 | 0.004 |
| NUFIP1 | nuclear fragile X mental retardation protein interacting protein 1 | 1.53 | 0.026 |
| ORC1 | origin recognition complex, subunit 1 | 1.53 | 0.036 |
| ZNF407 | zinc finger protein 407 | 1.53 | 0.014 |
| TK1 | thymidine kinase 1, soluble | 1.53 | 0.0013 |
| SLC7A5 | solute carrier family 7 (amino acid transporter light chain, L system), member 5 | 1.53 | 0.025 |
| TOMM34 | translocase of outer mitochondrial membrane 34 | 1.53 | 0.037 |
| ST5 | suppression of tumorigenicity 5 | 1.53 | 0.019 |
| PPIL1 | peptidylprolyl isomerase (cyclophilin)-like 1 | 1.53 | <0.0001 |
| FAM129A | family with sequence similarity 129, member A | 1.53 | 0.007 |
| ZFAND4 | zinc finger, AN1-type domain 4 | 1.53 | 0.009 |
| C6orf99 | chromosome 6 open reading frame 99 | 1.52 | 0.006 |
| FANCM | Fanconi anemia, complementation group M | 1.52 | 0.024 |
| GBP5 | guanylate binding protein 5 | 1.52 | 0.009 |
| USP2-AS1 | uncharacterized LOC100499227 | 1.52 | 0.027 |
| SPC24 | SPC24, NDC80 kinetochore complex component, homolog (S. cerevisiae) | 1.52 | 0.033 |
| SYNCRIP | synaptotagmin binding, cytoplasmic RNA interacting protein | 1.52 | 0.018 |
| AMD1 | adenosylmethionine decarboxylase 1 | 1.52 | 0.025 |
| SLC3A2 | solute carrier family 3 (activators of dibasic and neutral amino acid transport), member 2 | 1.52 | 0.032 |
| CCDC150 | coiled-coil domain containing 150 | 1.52 | 0.013 |
| FABP5 | fatty acid binding protein 5 (psoriasis-associated) | 1.51 | 0.013 |
| NCAPD2 | non-SMC condensin I complex, subunit D2 | 1.51 | 0.0003 |
| BUB1 | BUB1 mitotic checkpoint serine/threonine kinase | 1.51 | 0.041 |
| PANK3 | pantothenate kinase 3 | 1.51 | 0.042 |
| TROAP | trophinin associated protein | 1.51 | 0.023 |
| SHMT2 | serine hydroxymethyltransferase 2 (mitochondrial) | 1.51 | 0.013 |
| ACER3 | alkaline ceramidase 3 | 1.51 | 0.013 |
| FADS1 | fatty acid desaturase 1 | 1.51 | 0.004 |
| FAM86A | family with sequence similarity 86, member A | 1.51 | 0.05 |
| UTP15 | UTP15, U3 small nucleolar ribonucleoprotein, homolog (S. cerevisiae) | 1.51 | 0.025 |
| SIGMAR1 | sigma non-opioid intracellular receptor 1 | 1.50 | 0.027 |
| TP53 | tumor protein p53 | 1.50 | 0.034 |
| BRIX1 | BRX1, biogenesis of ribosomes, homolog (S. cerevisiae) | 1.50 | 0.028 |
| CDIP1 | cell death-inducing p53 target 1 | 1.50 | 0.023 |
| MCM7 | minichromosome maintenance complex component 7 | 1.50 | 0.003 |
| ALPK3 | alpha-kinase 3 | 1.50 | 0.05 |
| PRTN3 | proteinase 3 | 0.04 | 0.005 |
| CLEC2B | C-type lectin domain family 2, member B | 0.06 | 0.015 |
| KISS1R | KISS1 receptor | 0.07 | 0.05 |
| ISM2 | isthmin 2 homolog (zebrafish) | 0.08 | 0.017 |
| LINC00535 | long intergenic non-protein coding RNA 535 | 0.09 | 0.002 |
| PMP2 | peripheral myelin protein 2 | 0.10 | 0.035 |
| PDPN | podoplanin | 0.11 | 0.05 |
| CYP26A1 | cytochrome P450, family 26, subfamily A, polypeptide 1 | 0.11 | 0.008 |
| HLA-G | major histocompatibility complex, class I, G | 0.11 | 0.012 |
| TRIL | TLR4 interactor with leucine-rich repeats | 0.13 | 0.020 |
| DEFB109P1B | defensin, beta 109, pseudogene 1B | 0.13 | 0.018 |
| MIR2861 | microRNA 2861 | 0.14 | 0.026 |
| DTNA | dystrobrevin, alpha | 0.15 | 0.003 |
| LMO1 | LIM domain only 1 (rhombotin 1) | 0.15 | 0.022 |
| DCAF12L2 | DDB1 and CUL4 associated factor 12-like 2 | 0.15 | 0.008 |
| LGI4 | leucine-rich repeat LGI family, member 4 | 0.15 | 0.008 |
| OLFM2 | olfactomedin 2 | 0.16 | 0.047 |
| TTLL9 | tubulin tyrosine ligase-like family, member 9 | 0.17 | 0.013 |
| DRP2 | dystrophin related protein 2 | 0.17 | 0.038 |
| ALK | anaplastic lymphoma receptor tyrosine kinase | 0.17 | 0.038 |
| LOC145837 | uncharacterized LOC145837 | 0.17 | 0.008 |
| SNORD116-4 | small nucleolar RNA, C/D box 116-4 | 0.18 | 0.020 |
| PTGS1 | prostaglandin-endoperoxide synthase 1 (prostaglandin G/H synthase and cyclooxygenase) | 0.19 | 0.0002 |
| NACAP1 | nascent-polypeptide-associated complex alpha polypeptide pseudogene 1 | 0.19 | 0.038 |
| LOC100129617 | uncharacterized LOC100129617 | 0.19 | 0.021 |
| RASIP1 | Ras interacting protein 1 | 0.19 | 0.026 |
| AMZ1 | archaelysin family metallopeptidase 1 | 0.21 | 0.019 |
| SLC2A5 | solute carrier family 2 (facilitated glucose/fructose transporter), member 5 | 0.23 | 0.025 |
| ORM2 | orosomucoid 2 | 0.23 | 0.016 |
| CPXM2 | carboxypeptidase X (M14 family), member 2 | 0.23 | 0.010 |
| EEF1A2 | eukaryotic translation elongation factor 1 alpha 2 | 0.24 | 0.020 |
| VILL | villin-like | 0.24 | 0.003 |
| NKAIN1 | Na+/K+ transporting ATPase interacting 1 | 0.24 | 0.030 |
| S1PR4 | sphingosine-1-phosphate receptor 4 | 0.25 | 0.050 |
| HCG27 | HLA complex group 27 (non-protein coding) | 0.25 | 0.020 |
| MRPL23-AS1 | MRPL23 antisense RNA 1 | 0.25 | 0.023 |
| ANGPTL4 | angiopoietin-like 4 | 0.26 | 0.003 |
| FLJ43315 | asparagine synthetase pseudogene | 0.26 | 0.042 |
| DHDH | dihydrodiol dehydrogenase (dimeric) | 0.26 | 0.044 |
| AQP2 | aquaporin 2 (collecting duct) | 0.26 | 0.020 |
| RGS11 | regulator of G-protein signaling 11 | 0.27 | 0.05 |
| G0S2 | G0/G1switch 2 | 0.27 | 0.008 |
| H2BFM | H2B histone family, member M | 0.27 | 0.007 |
| FAM83A | family with sequence similarity 83, member A | 0.28 | 0.044 |
| GIPR | gastric inhibitory polypeptide receptor | 0.28 | 0.003 |
| SERPINE1 | serpin peptidase inhibitor, clade E (nexin, plasminogen activator inhibitor type 1), member 1 | 0.28 | 0.020 |
| LINC00312 | long intergenic non-protein coding RNA 312 | 0.29 | 0.038 |
| AKR1C2 | aldo-keto reductase family 1, member C2 | 0.29 | 0.042 |
| CSPG4 | chondroitin sulfate proteoglycan 4 | 0.29 | 0.036 |
| LGALS4 | lectin, galactoside-binding, soluble, 4 | 0.29 | 0.008 |
| HCG4B | HLA complex group 4B (non-protein coding) | 0.29 | 0.041 |
| SUSD3 | sushi domain containing 3 | 0.30 | 0.0015 |
| KCNK15 | potassium channel, subfamily K, member 15 | 0.30 | 0.004 |
| CRYAB | crystallin, alpha B | 0.30 | 0.05 |
| SNORD69 | small nucleolar RNA, C/D box 69 | 0.30 | 0.002 |
| TCTE3 | t-complex-associated-testis-expressed 3 | 0.31 | 0.004 |
| EFEMP2 | EGF containing fibulin-like extracellular matrix protein 2 | 0.31 | 0.019 |
| TMEM191C | transmembrane protein 191C | 0.31 | 0.028 |
| RNASE4 | ribonuclease, RNase A family, 4 | 0.31 | 0.003 |
| DSCR9 | Down syndrome critical region gene 9 (non-protein coding) | 0.31 | 0.037 |
| RASA4B | RAS p21 protein activator 4B | 0.32 | 0.020 |
| TFF3 | trefoil factor 3 (intestinal) | 0.32 | 0.029 |
| CNDP1 | carnosine dipeptidase 1 (metallopeptidase M20 family) | 0.32 | 0.034 |
| NDRG1 | N-myc downstream regulated 1 | 0.32 | 0.005 |
| TEX19 | testis expressed 19 | 0.33 | 0.050 |
| PLCH2 | phospholipase C, eta 2 | 0.33 | 0.012 |
| NDUFAF4P1 | NADH dehydrogenase (ubiquinone) complex I, assembly factor 4 pseudogene 1 | 0.33 | 0.020 |
| ENPEP | glutamyl aminopeptidase (aminopeptidase A) | 0.33 | 0.038 |
| SPTBN4 | spectrin, beta, non-erythrocytic 4 | 0.33 | 0.020 |
| DPEP1 | dipeptidase 1 (renal) | 0.34 | 0.034 |
| ANG | angiogenin, ribonuclease, RNase A family, 5 | 0.34 | 0.0013 |
| PPP1R3C | protein phosphatase 1, regulatory subunit 3C | 0.34 | 0.0009 |
| ASGR1 | asialoglycoprotein receptor 1 | 0.35 | 0.029 |
| ADM | adrenomedullin | 0.35 | 0.020 |
| CLCNKB | chloride channel, voltage-sensitive Kb | 0.35 | 0.010 |
| CDK18 | cyclin-dependent kinase 18 | 0.36 | 0.014 |
| FAM25A | family with sequence similarity 25, member A | 0.36 | 0.039 |
| GPER1 | G protein-coupled estrogen receptor 1 | 0.36 | 0.038 |
| PCOLCE2 | procollagen C-endopeptidase enhancer 2 | 0.36 | 0.023 |
| TREH | trehalase (brush-border membrane glycoprotein) | 0.37 | 0.025 |
| INSC | inscuteable homolog (Drosophila) | 0.38 | 0.038 |
| MAPT | microtubule-associated protein tau | 0.38 | 0.023 |
| LINC00202-1 | long intergenic non-protein coding RNA 202-1 | 0.38 | 0.023 |
| NRN1L | neuritin 1-like | 0.38 | 0.042 |
| WFDC3 | WAP four-disulfide core domain 3 | 0.38 | 0.007 |
| TRIM29 | tripartite motif containing 29 | 0.38 | 0.012 |
| ADAM11 | ADAM metallopeptidase domain 11 | 0.38 | 0.005 |
| C1orf213 | chromosome 1 open reading frame 213 | 0.38 | 0.005 |
| LEMD1 | LEM domain containing 1 | 0.38 | 0.019 |
| TMEM74B | transmembrane protein 74B | 0.38 | 0.017 |
| USP6 | ubiquitin specific peptidase 6 (Tre-2 oncogene) | 0.39 | 0.007 |
| SEMA5B | sema domain, seven thrombospondin repeats (type 1 and type 1-like), transmembrane domain (TM) and short cytoplasmic domain, (semaphorin) 5B | 0.39 | 0.032 |
| CGB7 | chorionic gonadotropin, beta polypeptide 7 | 0.39 | 0.013 |
| IL2RG | interleukin 2 receptor, gamma | 0.39 | 0.007 |
| RAET1G | retinoic acid early transcript 1G | 0.39 | 0.030 |
| HILPDA | hypoxia inducible lipid droplet-associated | 0.39 | 0.009 |
| ANKRD37 | ankyrin repeat domain 37 | 0.39 | 0.017 |
| FLJ44511 | uncharacterized LOC441307 | 0.39 | 0.022 |
| SNORD89 | small nucleolar RNA, C/D box 89 | 0.40 | 0.012 |
| SLC38A11 | solute carrier family 38, member 11 | 0.40 | 0.010 |
| NAPSA | napsin A aspartic peptidase | 0.40 | 0.010 |
| S100A14 | S100 calcium binding protein A14 | 0.40 | 0.013 |
| NR4A1 | nuclear receptor subfamily 4, group A, member 1 | 0.40 | 0.0015 |
| LINC00482 | long intergenic non-protein coding RNA 482 | 0.40 | 0.044 |
| ODF3L1 | outer dense fiber of sperm tails 3-like 1 | 0.40 | 0.022 |
| LINC00466 | long intergenic non-protein coding RNA 466 | 0.40 | 0.035 |
| AKAP12 | A kinase (PRKA) anchor protein 12 | 0.41 | 0.0008 |
| ADAMTS16 | ADAM metallopeptidase with thrombospondin type 1 motif, 16 | 0.41 | 0.011 |
| FGF9 | fibroblast growth factor 9 (glia-activating factor) | 0.41 | 0.011 |
| ENO2 | enolase 2 (gamma, neuronal) | 0.41 | 0.006 |
| POM121L1P | POM121 transmembrane nucleoporin-like 1, pseudogene | 0.41 | 0.05 |
| CD40LG | CD40 ligand | 0.41 | 0.038 |
| BEND5 | BEN domain containing 5 | 0.41 | 0.028 |
| TMEM178A | transmembrane protein 178A | 0.41 | 0.035 |
| CES4A | carboxylesterase 4A | 0.42 | 0.020 |
| CASR | calcium-sensing receptor | 0.42 | 0.0006 |
| NANOGNB | NANOG neighbor homeobox | 0.43 | 0.026 |
| LINC00887 | uncharacterized LOC100131551 | 0.43 | 0.008 |
| MMP28 | matrix metallopeptidase 28 | 0.43 | 0.018 |
| AQP7P3 | aquaporin 7 pseudogene 3 | 0.43 | 0.019 |
| AOC1 | amiloride binding protein 1 (amine oxidase (copper-containing)) | 0.43 | 0.006 |
| AQP3 | aquaporin 3 (Gill blood group) | 0.43 | 0.025 |
| AIM1L | absent in melanoma 1-like | 0.43 | 0.018 |
| MIR614 | microRNA 614 | 0.43 | 0.012 |
| SNORA44 | small nucleolar RNA, H/ACA box 44 | 0.43 | 0.049 |
| LINC00035 | long intergenic non-protein coding RNA 35 | 0.43 | 0.025 |
| ENOX1 | ecto-NOX disulfide-thiol exchanger 1 | 0.43 | 0.033 |
| ESPN | espin | 0.43 | 0.005 |
| TGM5 | transglutaminase 5 | 0.44 | 0.005 |
| SLC9A3 | solute carrier family 9, subfamily A (NHE3, cation proton antiporter 3), member 3 | 0.44 | 0.019 |
| C19orf71 | chromosome 19 open reading frame 71 | 0.44 | 0.020 |
| TRIM60 | tripartite motif containing 60 | 0.44 | 0.036 |
| SNHG12 | small nucleolar RNA host gene 12 (non-protein coding) | 0.44 | 0.014 |
| ICAM5 | intercellular adhesion molecule 5, telencephalin | 0.44 | 0.015 |
| MEF2C | myocyte enhancer factor 2C | 0.44 | 0.037 |
| OPRL1 | opiate receptor-like 1 | 0.44 | 0.038 |
| MYO7B | myosin VIIB | 0.45 | 0.007 |
| TEKT3 | tektin 3 | 0.45 | 0.05 |
| ZNF571-AS1 | uncharacterized LOC100507433 | 0.45 | 0.027 |
| RORC | RAR-related orphan receptor C | 0.45 | 0.021 |
| LINC-PINT | uncharacterized LOC378805 | 0.46 | 0.019 |
| TNXB | tenascin XB | 0.46 | 0.048 |
| NKAIN4 | Na+/K+ transporting ATPase interacting 4 | 0.46 | 0.012 |
| PLXNB3 | plexin B3 | 0.46 | 0.005 |
| C9orf173 | chromosome 9 open reading frame 173 | 0.46 | 0.010 |
| DCDC5 | doublecortin domain containing 5 | 0.46 | 0.047 |
| LOC100270804 | uncharacterized LOC100270804 | 0.46 | 0.025 |
| VTN | vitronectin | 0.47 | 0.015 |
| C4orf47 | chromosome 4 open reading frame 47 | 0.47 | 0.021 |
| C2CD4C | C2 calcium-dependent domain containing 4C | 0.47 | 0.002 |
| UPP2 | uridine phosphorylase 2 | 0.47 | 0.019 |
| CD68 | CD68 molecule | 0.47 | 0.014 |
| CTAGE7P | CTAGE family, member 7, pseudogene | 0.47 | 0.011 |
| SCN4B | sodium channel, voltage-gated, type IV, beta subunit | 0.47 | 0.010 |
| SIGLEC1 | sialic acid binding Ig-like lectin 1, sialoadhesin | 0.47 | 0.038 |
| IGFBP3 | insulin-like growth factor binding protein 3 | 0.47 | 0.0008 |
| WAS | Wiskott-Aldrich syndrome | 0.48 | 0.022 |
| SMIM24 | chromosome 19 open reading frame 77 | 0.48 | 0.038 |
| FAM110C | family with sequence similarity 110, member C | 0.48 | 0.005 |
| LINC00996 | uncharacterized LOC285972 | 0.48 | 0.015 |
| SNORA16A | small nucleolar RNA, H/ACA box 16A | 0.49 | 0.006 |
| SPAG8 | sperm associated antigen 8 | 0.49 | 0.043 |
| PPFIA4 | protein tyrosine phosphatase, receptor type, f polypeptide (PTPRF), interacting protein (liprin), alpha 4 | 0.49 | 0.012 |
| SEMA3B | sema domain, immunoglobulin domain (Ig), short basic domain, secreted, (semaphorin) 3B | 0.49 | 0.010 |
| FGL2 | fibrinogen-like 2 | 0.49 | 0.014 |
| NPR1 | natriuretic peptide receptor A/guanylate cyclase A (atrionatriuretic peptide receptor A) | 0.49 | 0.019 |
| RRAGD | Ras-related GTP binding D | 0.50 | 0.005 |
| SUGT1P1 | suppressor of G2 allele of SKP1 (S. cerevisiae) pseudogene 1 | 0.50 | 0.003 |
| NKAPP1 | NFKB activating protein pseudogene 1 | 0.50 | 0.019 |
| ACSL6 | acyl-CoA synthetase long-chain family member 6 | 0.50 | 0.021 |
| ISLR2 | immunoglobulin superfamily containing leucine-rich repeat 2 | 0.50 | 0.022 |
| MDK | midkine (neurite growth-promoting factor 2) | 0.50 | 0.034 |
| OSCAR | osteoclast associated, immunoglobulin-like receptor | 0.50 | 0.041 |
| BBOX1 | butyrobetaine (gamma), 2-oxoglutarate dioxygenase (gamma-butyrobetaine hydroxylase) 1 | 0.50 | 0.013 |
| TMEM110-MUSTN1 | TMEM110-MUSTN1 readthrough | 0.50 | 0.009 |
| SCART1 | scavenger receptor protein family member | 0.50 | 0.013 |
| PRSS53 | protease, serine, 53 | 0.50 | 0.029 |
| DUSP1 | dual specificity phosphatase 1 | 0.50 | 0.013 |
| LTC4S | leukotriene C4 synthase | 0.50 | 0.016 |
| ANK1 | ankyrin 1, erythrocytic | 0.50 | 0.007 |
| AKR1C1 | aldo-keto reductase family 1, member C1 | 0.50 | 0.029 |
| LINC00939 | uncharacterized LOC400084 | 0.50 | 0.009 |
| CKB | creatine kinase, brain | 0.51 | 0.012 |
| DCN | decorin | 0.51 | 0.019 |
| SNORD104 | small nucleolar RNA, C/D box 104 | 0.51 | 0.016 |
| SERPINF2 | serpin peptidase inhibitor, clade F (alpha-2 antiplasmin, pigment epithelium derived factor), member 2 | 0.51 | 0.05 |
| ENTPD8 | ectonucleoside triphosphate diphosphohydrolase 8 | 0.51 | 0.029 |
| LMCD1-AS1 | LMCD1 antisense RNA 1 (head to head) | 0.51 | 0.028 |
| KIAA1549L | KIAA1549-like | 0.51 | 0.010 |
| LINC01018 | uncharacterized LOC255167 | 0.51 | 0.002 |
| SH3D21 | SH3 domain containing 21 | 0.51 | 0.017 |
| LINC01021 | uncharacterized LOC643401 | 0.51 | 0.049 |
| ADAMTSL4 | ADAMTS-like 4 | 0.51 | 0.003 |
| SUSD2 | sushi domain containing 2 | 0.52 | 0.033 |
| BTBD16 | BTB (POZ) domain containing 16 | 0.52 | 0.026 |
| CA9 | carbonic anhydrase IX | 0.52 | 0.030 |
| PRRT1 | proline-rich transmembrane protein 1 | 0.52 | 0.009 |
| ZNF404 | zinc finger protein 404 | 0.52 | 0.0005 |
| FZD10 | frizzled family receptor 10 | 0.52 | 0.003 |
| MIR155HG | MIR155 host gene (non-protein coding) | 0.52 | 0.009 |
| M1AP | meiosis 1 associated protein | 0.52 | 0.037 |
| FRZB | frizzled-related protein | 0.52 | 0.05 |
| JAG2 | jagged 2 | 0.52 | 0.002 |
| MXRA8 | matrix-remodelling associated 8 | 0.52 | 0.020 |
| C2 | complement component 2 | 0.53 | 0.05 |
| LOC154761 | family with sequence similarity 115, member C pseudogene | 0.53 | 0.006 |
| P4HA1 | prolyl 4-hydroxylase, alpha polypeptide I | 0.53 | 0.0015 |
| ZNF208 | zinc finger protein 208 | 0.53 | 0.015 |
| PADI1 | peptidyl arginine deiminase, type I | 0.53 | 0.021 |
| MLXIPL | MLX interacting protein-like | 0.53 | 0.034 |
| FZD10-AS1 | uncharacterized LOC440119 | 0.53 | 0.015 |
| ENG | endoglin | 0.53 | 0.049 |
| IGFBP1 | insulin-like growth factor binding protein 1 | 0.54 | 0.020 |
| PTH1R | parathyroid hormone 1 receptor | 0.54 | 0.009 |
| LINC00271 | long intergenic non-protein coding RNA 271 | 0.54 | 0.048 |
| PIM1 | pim-1 oncogene | 0.54 | 0.017 |
| CLIP3 | CAP-GLY domain containing linker protein 3 | 0.54 | 0.037 |
| TENM1 | teneurin transmembrane protein 1 | 0.54 | 0.041 |
| GAPDHS | glyceraldehyde-3-phosphate dehydrogenase, spermatogenic | 0.54 | 0.008 |
| PROCA1 | protein interacting with cyclin A1 | 0.54 | 0.039 |
| C10orf10 | chromosome 10 open reading frame 10 | 0.54 | 0.014 |
| BDKRB2 | bradykinin receptor B2 | 0.54 | 0.037 |
| LRP1 | low density lipoprotein receptor-related protein 1 | 0.54 | 0.0003 |
| LOC284454 | uncharacterized LOC284454 | 0.54 | 0.029 |
| VAMP5 | vesicle-associated membrane protein 5 | 0.54 | 0.009 |
| S100A1 | S100 calcium binding protein A1 | 0.54 | 0.020 |
| LOC100129722 | uncharacterized LOC100129722 | 0.54 | 0.017 |
| FAM26F | family with sequence similarity 26, member F | 0.54 | 0.044 |
| IHH | indian hedgehog | 0.54 | 0.026 |
| MFNG | MFNG O-fucosylpeptide 3-beta-N-acetylglucosaminyltransferase | 0.55 | 0.05 |
| DGKK | diacylglycerol kinase, kappa | 0.55 | 0.038 |
| PMS2L2 | postmeiotic segregation increased 2-like 2 pseudogene | 0.55 | 0.020 |
| C7orf65 | chromosome 7 open reading frame 65 | 0.55 | 0.035 |
| B3GNT6 | UDP-GlcNAc:betaGal beta-1,3-N-acetylglucosaminyltransferase 6 (core 3 synthase) | 0.55 | 0.05 |
| INGX | inhibitor of growth family, X-linked, pseudogene | 0.55 | 0.050 |
| C10orf99 | chromosome 10 open reading frame 99 | 0.55 | 0.017 |
| CAPN14 | calpain 14 | 0.55 | 0.031 |
| PRODH | proline dehydrogenase (oxidase) 1 | 0.56 | 0.022 |
| TRIM17 | tripartite motif containing 17 | 0.56 | 0.026 |
| ATP6V1G2 | ATPase, H+ transporting, lysosomal 13kDa, V1 subunit G2 | 0.56 | 0.05 |
| SSTR5-AS1 | SSTR5 antisense RNA 1 | 0.56 | 0.006 |
| ACKR4 | chemokine (C-C motif) receptor-like 1 | 0.56 | 0.049 |
| MSLN | mesothelin | 0.56 | 0.020 |
| TMEM229B | transmembrane protein 229B | 0.56 | 0.006 |
| MT1X | metallothionein 1X | 0.56 | 0.017 |
| SLC26A4 | solute carrier family 26, member 4 | 0.56 | 0.046 |
| LOC100132062 | uncharacterized LOC100132062 | 0.56 | 0.014 |
| FAM83E | family with sequence similarity 83, member E | 0.56 | 0.013 |
| LINC00473 | long intergenic non-protein coding RNA 473 | 0.56 | 0.019 |
| THEMIS2 | thymocyte selection associated family member 2 | 0.57 | 0.007 |
| UNC13A | unc-13 homolog A (C. elegans) | 0.57 | 0.038 |
| ATG16L2 | autophagy related 16-like 2 (S. cerevisiae) | 0.57 | 0.0011 |
| COX6B2 | cytochrome c oxidase subunit VIb polypeptide 2 (testis) | 0.57 | 0.039 |
| ARL11 | ADP-ribosylation factor-like 11 | 0.57 | 0.030 |
| N4BP2L2-IT2 | N4BPL2 intronic transcript 2 (non-protein coding) | 0.57 | 0.008 |
| NEAT1 | nuclear paraspeckle assembly transcript 1 (non-protein coding) | 0.57 | 0.013 |
| WSB1 | WD repeat and SOCS box containing 1 | 0.57 | 0.0011 |
| THBS4 | thrombospondin 4 | 0.57 | 0.037 |
| C20orf203 | chromosome 20 open reading frame 203 | 0.57 | 0.027 |
| FAM115C | family with sequence similarity 115, member C | 0.57 | 0.043 |
| SSPO | SCO-spondin homolog (Bos taurus) | 0.57 | 0.027 |
| LINC00950 | uncharacterized LOC92973 | 0.57 | 0.036 |
| TSSK3 | testis-specific serine kinase 3 | 0.57 | 0.014 |
| GS1-259H13.2 | transmembrane protein 225-like | 0.57 | 0.036 |
| UCN | urocortin | 0.57 | 0.004 |
| KRT42P | keratin 42 pseudogene | 0.57 | 0.034 |
| SLC6A10P | solute carrier family 6 (neurotransmitter transporter, creatine), member 10, pseudogene | 0.57 | 0.031 |
| Sep-01 | septin 1 | 0.58 | 0.029 |
| LOC729683 | uncharacterized LOC729683 | 0.58 | 0.046 |
| LOC155060 | AI894139 pseudogene | 0.58 | 0.021 |
| RND2 | Rho family GTPase 2 | 0.58 | 0.037 |
| SSX5 | synovial sarcoma, X breakpoint 5 | 0.58 | 0.020 |
| KCNIP2-AS1 | uncharacterized LOC100289509 | 0.58 | 0.031 |
| RTBDN | retbindin | 0.59 | 0.010 |
| LINC00936 | uncharacterized LOC338758 | 0.59 | 0.022 |
| SLPI | secretory leukocyte peptidase inhibitor | 0.59 | 0.006 |
| N4BP2L1 | NEDD4 binding protein 2-like 1 | 0.59 | 0.014 |
| DIRAS2 | DIRAS family, GTP-binding RAS-like 2 | 0.59 | 0.010 |
| KIAA0087 | KIAA0087 | 0.59 | 0.029 |
| CFD | complement factor D (adipsin) | 0.59 | 0.004 |
| MST1 | macrophage stimulating 1 (hepatocyte growth factor-like) | 0.59 | 0.007 |
| SFMBT2 | Scm-like with four mbt domains 2 | 0.59 | 0.006 |
| LOX | lysyl oxidase | 0.59 | 0.006 |
| ST7-OT4 | ST7 overlapping transcript 4 | 0.59 | 0.008 |
| C1QL4 | complement component 1, q subcomponent-like 4 | 0.59 | 0.05 |
| PRSS8 | protease, serine, 8 | 0.59 | 0.033 |
| GSDMC | gasdermin C | 0.60 | 0.05 |
| ITM2C | integral membrane protein 2C | 0.60 | 0.006 |
| GABRE | gamma-aminobutyric acid (GABA) A receptor, epsilon | 0.60 | 0.004 |
| SLC2A1 | solute carrier family 2 (facilitated glucose transporter), member 1 | 0.60 | 0.014 |
| CYP4F3 | cytochrome P450, family 4, subfamily F, polypeptide 3 | 0.60 | 0.005 |
| KRT15 | keratin 15 | 0.60 | 0.014 |
| TNNI3 | troponin I type 3 (cardiac) | 0.60 | 0.007 |
| PRSS22 | protease, serine, 22 | 0.60 | 0.05 |
| C6orf141 | chromosome 6 open reading frame 141 | 0.60 | 0.032 |
| FBXL16 | F-box and leucine-rich repeat protein 16 | 0.60 | 0.028 |
| SNORA8 | small nucleolar RNA, H/ACA box 8 | 0.60 | 0.041 |
| F3 | coagulation factor III (thromboplastin, tissue factor) | 0.60 | 0.010 |
| HHLA2 | HERV-H LTR-associating 2 | 0.60 | 0.009 |
| CEL | carboxyl ester lipase | 0.60 | 0.046 |
| VKORC1 | vitamin K epoxide reductase complex, subunit 1 | 0.60 | 0.005 |
| VSIG10L | V-set and immunoglobulin domain containing 10 like | 0.60 | 0.05 |
| LOC286297 | uncharacterized LOC286297 | 0.60 | 0.048 |
| SAPCD1 | suppressor APC domain containing 1 | 0.60 | 0.009 |
| TINAG | tubulointerstitial nephritis antigen | 0.60 | 0.008 |
| PCAT6 | KDM5B antisense RNA 1 (head to head) | 0.60 | 0.034 |
| SYT13 | synaptotagmin XIII | 0.60 | 0.012 |
| LMNTD2 | chromosome 11 open reading frame 35 | 0.61 | 0.011 |
| C1R | complement component 1, r subcomponent | 0.61 | 0.020 |
| PDE6B | phosphodiesterase 6B, cGMP-specific, rod, beta | 0.61 | 0.048 |
| MYO15B | myosin XVB pseudogene | 0.61 | 0.0001 |
| PSCA | prostate stem cell antigen | 0.61 | 0.031 |
| MTMR11 | myotubularin related protein 11 | 0.61 | 0.002 |
| FTLP10 | ferritin, light polypeptide pseudogene 10 | 0.61 | 0.0014 |
| RASA4 | RAS p21 protein activator 4 | 0.61 | 0.05 |
| FOS | FBJ murine osteosarcoma viral oncogene homolog | 0.61 | 0.021 |
| GSDMA | gasdermin A | 0.61 | 0.003 |
| HLA-F-AS1 | HLA-F antisense RNA 1 | 0.61 | 0.016 |
| FGFR3 | fibroblast growth factor receptor 3 | 0.61 | 0.011 |
| KCTD7 | potassium channel tetramerisation domain containing 7 | 0.61 | 0.042 |
| PNPLA7 | patatin-like phospholipase domain containing 7 | 0.61 | 0.042 |
| RHBDL1 | rhomboid, veinlet-like 1 (Drosophila) | 0.61 | 0.022 |
| SNHG18 | uncharacterized LOC100505806 | 0.61 | 0.025 |
| PAK6 | p21 protein (Cdc42/Rac)-activated kinase 6 | 0.61 | 0.020 |
| LOC100131564 | uncharacterized LOC100131564 | 0.61 | 0.034 |
| BMP7 | bone morphogenetic protein 7 | 0.61 | 0.029 |
| SNX33 | sorting nexin 33 | 0.61 | 0.009 |
| HTR2A | 5-hydroxytryptamine (serotonin) receptor 2A, G protein-coupled | 0.62 | 0.010 |
| CLU | clusterin | 0.62 | 0.009 |
| TMEM70 | transmembrane protein 70 | 0.62 | 0.005 |
| C4A | complement component 4A (Rodgers blood group) | 0.62 | 0.017 |
| PTPRS | protein tyrosine phosphatase, receptor type, S | 0.62 | 0.015 |
| CDKN1C | cyclin-dependent kinase inhibitor 1C (p57, Kip2) | 0.62 | 0.026 |
| CEACAM5 | carcinoembryonic antigen-related cell adhesion molecule 5 | 0.62 | 0.0006 |
| LINC00515 | long intergenic non-protein coding RNA 515 | 0.62 | 0.030 |
| TMC4 | transmembrane channel-like 4 | 0.62 | 0.002 |
| PLAUR | plasminogen activator, urokinase receptor | 0.62 | 0.025 |
| APOE | apolipoprotein E | 0.62 | 0.011 |
| TSLP | thymic stromal lymphopoietin | 0.62 | 0.006 |
| EPOR | erythropoietin receptor | 0.62 | 0.016 |
| LMBR1L | limb region 1 homolog (mouse)-like | 0.62 | 0.005 |
| LDLRAD1 | low density lipoprotein receptor class A domain containing 1 | 0.63 | 0.033 |
| CTSF | cathepsin F | 0.63 | 0.039 |
| KRTCAP3 | keratinocyte associated protein 3 | 0.63 | 0.05 |
| LINC01004 | uncharacterized LOC100216546 | 0.63 | 0.021 |
| GNRH1 | gonadotropin-releasing hormone 1 (luteinizing-releasing hormone) | 0.63 | 0.024 |
| IRF8 | interferon regulatory factor 8 | 0.63 | 0.003 |
| ZNF674-AS1 | ZNF674 antisense RNA 1 (head to head) | 0.63 | 0.05 |
| SLC3A1 | solute carrier family 3 (cystine, dibasic and neutral amino acid transporters, activator of cystine, dibasic and neutral amino acid transport), member 1 | 0.63 | 0.011 |
| C10orf95 | chromosome 10 open reading frame 95 | 0.63 | 0.032 |
| TBC1D3F | TBC1 domain family, member 3F | 0.63 | 0.020 |
| SH3YL1 | SH3 domain containing, Ysc84-like 1 (S. cerevisiae) | 0.63 | 0.017 |
| BIN3-IT1 | uncharacterized LOC80094 | 0.63 | 0.032 |
| CYP2B7P | cytochrome P450, family 2, subfamily B, polypeptide 7 pseudogene 1 | 0.63 | 0.031 |
| LOC440434 | aminopeptidase puromycin sensitive pseudogene | 0.63 | 0.039 |
| INSR | insulin receptor | 0.63 | 0.05 |
| ZNF540 | zinc finger protein 540 | 0.63 | 0.042 |
| F2RL2 | coagulation factor II (thrombin) receptor-like 2 | 0.63 | 0.048 |
| CACNB4 | calcium channel, voltage-dependent, beta 4 subunit | 0.63 | 0.009 |
| SEC14L6 | SEC14-like 6 (S. cerevisiae) | 0.63 | 0.006 |
| PDK4 | pyruvate dehydrogenase kinase, isozyme 4 | 0.63 | 0.039 |
| SLC25A29 | solute carrier family 25 (mitochondrial carnitine/acylcarnitine carrier), member 29 | 0.64 | 0.011 |
| YPEL4 | yippee-like 4 (Drosophila) | 0.64 | 0.015 |
| DICER1-AS1 | DICER1 antisense RNA 1 | 0.64 | 0.023 |
| TMEM61 | transmembrane protein 61 | 0.64 | 0.022 |
| VAC14-AS1 | uncharacterized LOC100130894 | 0.64 | 0.018 |
| DGCR5 | DiGeorge syndrome critical region gene 5 (non-protein coding) | 0.64 | 0.003 |
| ASS1 | argininosuccinate synthase 1 | 0.64 | 0.005 |
| RSRP1 | chromosome 1 open reading frame 63 | 0.64 | 0.002 |
| LINC00937 | uncharacterized LOC389634 | 0.64 | 0.018 |
| CYP21A1P | cytochrome P450, family 21, subfamily A, polypeptide 1 pseudogene | 0.64 | 0.031 |
| MAPK8IP3 | mitogen-activated protein kinase 8 interacting protein 3 | 0.64 | 0.037 |
| RNASET2 | ribonuclease T2 | 0.64 | 0.004 |
| AMT | aminomethyltransferase | 0.64 | 0.009 |
| UGT1A6 | UDP glucuronosyltransferase 1 family, polypeptide A6 | 0.64 | 0.003 |
| GPRC5A | G protein-coupled receptor, family C, group 5, member A | 0.64 | 0.002 |
| KLHL6 | kelch-like family member 6 | 0.64 | 0.003 |
| THBS3 | thrombospondin 3 | 0.64 | 0.004 |
| SNORA6 | small nucleolar RNA, H/ACA box 6 | 0.64 | 0.014 |
| PLXNA3 | plexin A3 | 0.64 | 0.024 |
| CLDN14 | claudin 14 | 0.64 | 0.034 |
| LETMD1 | LETM1 domain containing 1 | 0.64 | 0.009 |
| PAQR6 | progestin and adipoQ receptor family member VI | 0.65 | 0.003 |
| TCN2 | transcobalamin II | 0.65 | 0.019 |
| SEC14L5 | SEC14-like 5 (S. cerevisiae) | 0.65 | 0.025 |
| CD55 | CD55 molecule, decay accelerating factor for complement (Cromer blood group) | 0.65 | 0.012 |
| CLEC19A | C-type lectin domain family 19, member A | 0.65 | 0.028 |
| SYTL3 | synaptotagmin-like 3 | 0.65 | 0.0005 |
| SCARF1 | scavenger receptor class F, member 1 | 0.65 | 0.005 |
| PPP1R3E | protein phosphatase 1, regulatory subunit 3E | 0.65 | 0.044 |
| LGALS1 | lectin, galactoside-binding, soluble, 1 | 0.65 | 0.013 |
| LRAT | lecithin retinol acyltransferase (phosphatidylcholine--retinol O-acyltransferase) | 0.65 | 0.025 |
| CCDC114 | coiled-coil domain containing 114 | 0.65 | 0.026 |
| ADAMTS14 | ADAM metallopeptidase with thrombospondin type 1 motif, 14 | 0.65 | 0.041 |
| SLC25A27 | solute carrier family 25, member 27 | 0.65 | 0.038 |
| PAXIP1-AS1 | uncharacterized LOC202781 | 0.65 | 0.043 |
| ARNT2 | aryl-hydrocarbon receptor nuclear translocator 2 | 0.65 | 0.026 |
| LOC728743 | zinc finger protein pseudogene | 0.65 | 0.015 |
| PAG1 | phosphoprotein associated with glycosphingolipid microdomains 1 | 0.65 | 0.034 |
| GPR146 | G protein-coupled receptor 146 | 0.65 | 0.035 |
| FANK1 | fibronectin type III and ankyrin repeat domains 1 | 0.65 | 0.014 |
| MC1R | melanocortin 1 receptor (alpha melanocyte stimulating hormone receptor) | 0.66 | 0.008 |
| VWA7 | von Willebrand factor A domain containing 7 | 0.66 | 0.025 |
| EMILIN3 | elastin microfibril interfacer 3 | 0.66 | 0.006 |
| CCDC88B | coiled-coil domain containing 88B | 0.66 | 0.011 |
| LOC100506469 | uncharacterized LOC100506469 | 0.66 | 0.015 |
| CD36 | CD36 molecule (thrombospondin receptor) | 0.66 | 0.025 |
| LENG8 | leukocyte receptor cluster (LRC) member 8 | 0.66 | 0.011 |
| ATP6V1B1 | ATPase, H+ transporting, lysosomal 56/58kDa, V1 subunit B1 | 0.66 | 0.032 |
| KIZ | polo-like kinase 1 substrate 1 | 0.66 | 0.023 |
| ADHFE1 | alcohol dehydrogenase, iron containing, 1 | 0.66 | 0.018 |
| LOC400655 | uncharacterized LOC400655 | 0.66 | 0.007 |
| PEX6 | peroxisomal biogenesis factor 6 | 0.66 | 0.003 |
| IFNE | interferon, epsilon | 0.67 | 0.024 |
| CYP2E1 | cytochrome P450, family 2, subfamily E, polypeptide 1 | 0.67 | <0.0001 |
| KCND1 | potassium voltage-gated channel, Shal-related subfamily, member 1 | 0.67 | 0.045 |
