# Supplementary material for: Human mesenchymal stromal cells broadly modulate high glucose-induced inflammatory responses of renal proximal tubular cell monolayers
Source: Stem Cell Res Ther. 2019 Nov 19;10:329. doi: 10.1186/s13287-019-1424-5 (PMC6862760; doi:10.1186/s13287-019-1424-5)
Supplement: Supplementary file 15 — Additional file 15: Table S6. List of DEGs with significant Fold Changes in RPTEC/TERT1 cells without and with MSC co-culture in mannitol (MAN) condition. [file 13287_2019_1424_MOESM15_ESM.docx]

| **Supplementary Table S6: List of DEGs with significant Fold Changes in RPTEC/TERT1 cells without and with MSC co-culture in mannitol (MAN) condition** | | | |
| --- | --- | --- | --- |
|  | Higher expression in No-MSC versus MSC co-culture (MAN) |  |  |
|  | Lower expression in No-MSC versus MSC co-culture (MAN) |  |  |
|  |  |  |  |
| **Gene Symbol** | **Gene Description** | **Fold change No-MSC vs MSC** | **P Value** |
| KBTBD8 | kelch repeat and BTB (POZ) domain containing 8 | 26.00 | 0.045 |
| GAL | galanin/GMAP prepropeptide | 12.40 | 0.042 |
| BEGAIN | brain-enriched guanylate kinase-associated | 11.00 | 0.044 |
| MYRIP | myosin VIIA and Rab interacting protein | 10.00 | 0.035 |
| ZFP91-CNTF | ZFP91-CNTF readthrough (non-protein coding) | 8.63 | 0.002 |
| IL1RL1 | interleukin 1 receptor-like 1 | 8.14 | 0.045 |
| ATOH8 | atonal homolog 8 (Drosophila) | 8.00 | 0.020 |
| AOX1 | aldehyde oxidase 1 | 7.75 | 0.004 |
| CABYR | calcium binding tyrosine-(Y)-phosphorylation regulated | 6.67 | 0.011 |
| TMEFF1 | transmembrane protein with EGF-like and two follistatin-like domains 1 | 6.36 | 0.025 |
| THRSP | thyroid hormone responsive | 6.33 | 0.022 |
| EPN2-IT1 | EPN2 intronic transcript 1 (non-protein coding) | 6.00 | 0.031 |
| B3GALT1 | UDP-Gal:betaGlcNAc beta 1,3-galactosyltransferase, polypeptide 1 | 5.45 | 0.003 |
| RAB40AL | RAB40A, member RAS oncogene family-like | 5.44 | 0.044 |
| PCDHA11 | protocadherin alpha 11 | 5.00 | 0.015 |
| UBD | ubiquitin D | 4.66 | 0.033 |
| - | BMS1 pseudogene 2 | 4.63 | 0.018 |
| HIST1H4H | histone cluster 1, H4h | 4.55 | 0.019 |
| MIR4683 | microRNA 4683 | 4.48 | 0.021 |
| AVPR1B | arginine vasopressin receptor 1B | 4.47 | 0.018 |
| ASIC5 | acid-sensing (proton-gated) ion channel family member 5 | 4.33 | 0.038 |
| LOC339240 | keratin 17 pseudogene | 4.20 | 0.047 |
| NAV3 | neuron navigator 3 | 4.05 | 0.017 |
| PLEKHS1 | pleckstrin homology domain containing, family S member 1 | 4.04 | 0.033 |
| SYT1 | synaptotagmin I | 4.00 | 0.020 |
| BEND6 | BEN domain containing 6 | 4.00 | 0.027 |
| ALKBH3-AS1 | uncharacterized LOC100507300 | 4.00 | 0.035 |
| SNORA76C | small nucleolar RNA, H/ACA box 76 | 3.71 | 0.023 |
| C6orf7 | chromosome 6 open reading frame 7 | 3.65 | 0.05 |
| FLJ22447 | uncharacterized LOC400221 | 3.55 | 0.003 |
| UNC45B | unc-45 homolog B (C. elegans) | 3.50 | 0.038 |
| TMEM72 | transmembrane protein 72 | 3.25 | 0.006 |
| OR12D2 | olfactory receptor, family 12, subfamily D, member 2 | 3.20 | 0.017 |
| LINC00518 | long intergenic non-protein coding RNA 518 | 3.20 | 0.032 |
| MIR3960 | microRNA 3960 | 3.12 | 0.013 |
| BMPR1B | bone morphogenetic protein receptor, type IB | 3.11 | 0.011 |
| CCL2 | chemokine (C-C motif) ligand 2 | 3.11 | 0.019 |
| PPM1L | protein phosphatase, Mg2+/Mn2+ dependent, 1L | 3.02 | 0.001 |
| APOL3 | apolipoprotein L, 3 | 3.00 | 0.010 |
| NLRP9 | NLR family, pyrin domain containing 9 | 3.00 | 0.010 |
| EPPK1 | epiplakin 1 | 3.00 | 0.038 |
| LINC00313 | long intergenic non-protein coding RNA 313 | 3.00 | 0.040 |
| STEAP1B | STEAP family member 1B | 3.00 | 0.026 |
| FAM66B | family with sequence similarity 66, member B | 2.95 | 0.021 |
| CXCL2 | chemokine (C-X-C motif) ligand 2 | 2.94 | 0.027 |
| PLAC8L1 | PLAC8-like 1 | 2.93 | 0.008 |
| MIR200A | microRNA 200a | 2.92 | 0.042 |
| PHGDH | phosphoglycerate dehydrogenase | 2.91 | 0.021 |
| BEST3 | bestrophin 3 | 2.89 | 0.042 |
| ERCC6L | excision repair cross-complementing rodent repair deficiency, complementation group 6-like | 2.86 | 0.006 |
| MCM10 | minichromosome maintenance complex component 10 | 2.86 | 0.004 |
| ABCA12 | ATP-binding cassette, sub-family A (ABC1), member 12 | 2.84 | 0.037 |
| EXO1 | exonuclease 1 | 2.82 | 0.005 |
| SKA3 | spindle and kinetochore associated complex subunit 3 | 2.82 | 0.008 |
| CASC5 | cancer susceptibility candidate 5 | 2.78 | 0.022 |
| SCUBE3 | signal peptide, CUB domain, EGF-like 3 | 2.78 | 0.004 |
| HIST1H2AG | histone cluster 1, H2ag | 2.77 | 0.045 |
| ASPM | asp (abnormal spindle) homolog, microcephaly associated (Drosophila) | 2.77 | 0.005 |
| EGFR-AS1 | EGFR antisense RNA 1 | 2.76 | 0.028 |
| ESCO2 | establishment of cohesion 1 homolog 2 (S. cerevisiae) | 2.76 | 0.014 |
| C17orf51 | chromosome 17 open reading frame 51 | 2.74 | 0.033 |
| SYT16 | synaptotagmin XVI | 2.74 | 0.049 |
| POLQ | polymerase (DNA directed), theta | 2.73 | 0.0006 |
| HIST1H2BC | histone cluster 1, H2bc | 2.72 | 0.038 |
| GREM1 | gremlin 1, DAN family BMP antagonist | 2.71 | 0.045 |
| KIF14 | kinesin family member 14 | 2.70 | 0.007 |
| SSR4P1 | signal sequence receptor, delta pseudogene 1 | 2.69 | 0.048 |
| SERTAD4 | SERTA domain containing 4 | 2.68 | 0.012 |
| LNPEP | leucyl/cystinyl aminopeptidase | 2.68 | 0.040 |
| DEPDC1 | DEP domain containing 1 | 2.61 | 0.004 |
| NCR3LG1 | natural killer cell cytotoxicity receptor 3 ligand 1 | 2.61 | 0.039 |
| LINC00701 | long intergenic non-protein coding RNA 701 | 2.60 | 0.015 |
| TP53AIP1 | tumor protein p53 regulated apoptosis inducing protein 1 | 2.60 | 0.048 |
| CKAP2L | cytoskeleton associated protein 2-like | 2.59 | 0.0009 |
| FAM111B | family with sequence similarity 111, member B | 2.58 | 0.010 |
| GDAP1 | ganglioside induced differentiation associated protein 1 | 2.57 | <0.0001 |
| SELL | selectin L | 2.57 | 0.011 |
| MLLT10P1 | myeloid/lymphoid or mixed-lineage leukemia (trithorax homolog, Drosophila); translocated to, 10 pseudogene 1 | 2.57 | 0.041 |
| ZIC5 | Zic family member 5 | 2.56 | 0.011 |
| BRCA2 | breast cancer 2, early onset | 2.54 | 0.0009 |
| ANLN | anillin, actin binding protein | 2.53 | 0.007 |
| TGFB2 | transforming growth factor, beta 2 | 2.52 | 0.004 |
| SLFN11 | schlafen family member 11 | 2.50 | 0.026 |
| SNORD26 | small nucleolar RNA, C/D box 26 | 2.50 | 0.047 |
| ACTL10 | actin-like 10 | 2.49 | 0.003 |
| DDO | D-aspartate oxidase | 2.49 | 0.001 |
| SOD2 | superoxide dismutase 2, mitochondrial | 2.49 | 0.038 |
| KIF26B | kinesin family member 26B | 2.48 | 0.05 |
| PIGR | polymeric immunoglobulin receptor | 2.45 | 0.013 |
| RFX3 | regulatory factor X, 3 (influences HLA class II expression) | 2.45 | 0.030 |
| NR1D2 | nuclear receptor subfamily 1, group D, member 2 | 2.44 | 0.05 |
| CEP97 | centrosomal protein 97kDa | 2.44 | 0.049 |
| HMCN1 | hemicentin 1 | 2.43 | 0.019 |
| GTSE1 | G-2 and S-phase expressed 1 | 2.42 | 0.007 |
| THSD7B | thrombospondin, type I, domain containing 7B | 2.41 | 0.047 |
| PTGS2 | prostaglandin-endoperoxide synthase 2 (prostaglandin G/H synthase and cyclooxygenase) | 2.40 | 0.034 |
| CDC45 | cell division cycle 45 | 2.39 | 0.004 |
| LRGUK | leucine-rich repeats and guanylate kinase domain containing | 2.38 | 0.029 |
| IL1B | interleukin 1, beta | 2.37 | <0.0001 |
| IL6 | interleukin 6 (interferon, beta 2) | 2.37 | 0.050 |
| C11orf82 | chromosome 11 open reading frame 82 | 2.37 | 0.015 |
| TERT | telomerase reverse transcriptase | 2.37 | 0.038 |
| HPD | 4-hydroxyphenylpyruvate dioxygenase | 2.36 | 0.038 |
| CENPA | centromere protein A | 2.36 | 0.001 |
| GNAZ | guanine nucleotide binding protein (G protein), alpha z polypeptide | 2.36 | 0.024 |
| CENPF | centromere protein F, 350/400kDa | 2.36 | 0.003 |
| PBK | PDZ binding kinase | 2.35 | 0.022 |
| ACTBL2 | actin, beta-like 2 | 2.34 | 0.032 |
| BUB1B | BUB1 mitotic checkpoint serine/threonine kinase B | 2.33 | 0.006 |
| DTL | denticleless E3 ubiquitin protein ligase homolog (Drosophila) | 2.33 | 0.020 |
| TOP2A | topoisomerase (DNA) II alpha 170kDa | 2.33 | 0.003 |
| DLL3 | delta-like 3 (Drosophila) | 2.32 | 0.019 |
| BLM | Bloom syndrome, RecQ helicase-like | 2.32 | 0.037 |
| SLFN5 | schlafen family member 5 | 2.32 | 0.049 |
| CDKL5 | cyclin-dependent kinase-like 5 | 2.30 | 0.05 |
| DYNC1I1 | dynein, cytoplasmic 1, intermediate chain 1 | 2.30 | 0.033 |
| ALPK2 | alpha-kinase 2 | 2.28 | 0.013 |
| ERCC6L2 | excision repair cross-complementing rodent repair deficiency, complementation group 6-like 2 | 2.26 | 0.008 |
| CDCA5 | cell division cycle associated 5 | 2.26 | 0.004 |
| EDN2 | endothelin 2 | 2.26 | 0.023 |
| FAM71F1 | family with sequence similarity 71, member F1 | 2.25 | 0.038 |
| KIF15 | kinesin family member 15 | 2.25 | 0.005 |
| PTPLB | protein tyrosine phosphatase-like (proline instead of catalytic arginine), member b | 2.24 | 0.032 |
| NDC80 | NDC80 kinetochore complex component | 2.23 | 0.013 |
| ITIH2 | inter-alpha-trypsin inhibitor heavy chain 2 | 2.23 | 0.0007 |
| TMPO-AS1 | TMPO antisense RNA 1 | 2.23 | 0.012 |
| SHCBP1 | SHC SH2-domain binding protein 1 | 2.22 | 0.016 |
| TRIM6 | tripartite motif containing 6 | 2.22 | 0.006 |
| PCNA-AS1 | PCNA antisense RNA 1 | 2.20 | 0.001 |
| COL21A1 | collagen, type XXI, alpha 1 | 2.19 | 0.046 |
| RC3H2 | ring finger and CCCH-type domains 2 | 2.18 | 0.039 |
| DLGAP5 | discs, large (Drosophila) homolog-associated protein 5 | 2.18 | 0.009 |
| MIR4321 | microRNA 4321 | 2.18 | 0.044 |
| TLL1 | tolloid-like 1 | 2.18 | 0.017 |
| NUF2 | NUF2, NDC80 kinetochore complex component, homolog (S. cerevisiae) | 2.17 | 0.025 |
| PMFBP1 | polyamine modulated factor 1 binding protein 1 | 2.17 | 0.005 |
| ZGRF1 | chromosome 4 open reading frame 21 | 2.16 | 0.012 |
| RRAD | Ras-related associated with diabetes | 2.16 | 0.010 |
| MKI67 | antigen identified by monoclonal antibody Ki-67 | 2.16 | 0.007 |
| HELLS | helicase, lymphoid-specific | 2.14 | <0.0001 |
| KIF4A | kinesin family member 4A | 2.14 | 0.001 |
| PTPRB | protein tyrosine phosphatase, receptor type, B | 2.14 | 0.0004 |
| BARD1 | BRCA1 associated RING domain 1 | 2.12 | 0.05 |
| SPC24 | SPC24, NDC80 kinetochore complex component, homolog (S. cerevisiae) | 2.12 | 0.009 |
| CEP55 | centrosomal protein 55kDa | 2.12 | 0.031 |
| LDHAL6B | lactate dehydrogenase A-like 6B | 2.11 | 0.041 |
| KCNAB3 | potassium voltage-gated channel, shaker-related subfamily, beta member 3 | 2.11 | 0.041 |
| SNORD16 | small nucleolar RNA, C/D box 16 | 2.11 | 0.007 |
| RAD51AP1 | RAD51 associated protein 1 | 2.11 | 0.007 |
| NIPAL1 | NIPA-like domain containing 1 | 2.10 | 0.024 |
| SYNE1 | spectrin repeat containing, nuclear envelope 1 | 2.10 | 0.037 |
| TNF | tumor necrosis factor | 2.09 | 0.017 |
| C3orf36 | chromosome 3 open reading frame 36 | 2.09 | 0.0007 |
| RFC3 | replication factor C (activator 1) 3, 38kDa | 2.08 | 0.016 |
| ANTXR1 | anthrax toxin receptor 1 | 2.07 | 0.0009 |
| ULBP3 | UL16 binding protein 3 | 2.07 | 0.016 |
| XYLB | xylulokinase homolog (H. influenzae) | 2.07 | 0.040 |
| FRK | fyn-related kinase | 2.07 | 0.037 |
| BIRC5 | baculoviral IAP repeat containing 5 | 2.06 | 0.012 |
| CLIC5 | chloride intracellular channel 5 | 2.06 | 0.024 |
| ZNF891 | zinc finger protein 891 | 2.04 | 0.05 |
| GPR89B | G protein-coupled receptor 89B | 2.03 | 0.042 |
| PCLO | piccolo presynaptic cytomatrix protein | 2.03 | 0.016 |
| WDR43 | WD repeat domain 43 | 2.03 | 0.037 |
| PSAT1 | phosphoserine aminotransferase 1 | 2.03 | 0.031 |
| LRRC31 | leucine rich repeat containing 31 | 2.03 | 0.035 |
| LCN2 | lipocalin 2 | 2.02 | 0.044 |
| ENC1 | ectodermal-neural cortex 1 (with BTB domain) | 2.01 | 0.014 |
| IKZF2 | IKAROS family zinc finger 2 (Helios) | 2.01 | 0.028 |
| TGM4 | transglutaminase 4 (prostate) | 2.00 | 0.035 |
| NUSAP1 | nucleolar and spindle associated protein 1 | 2.00 | 0.033 |
| IL6ST | interleukin 6 signal transducer (gp130, oncostatin M receptor) | 2.00 | 0.042 |
| NEBL | nebulette | 1.99 | 0.041 |
| BDNF | brain-derived neurotrophic factor | 1.99 | 0.024 |
| CIITA | class II, major histocompatibility complex, transactivator | 1.99 | 0.023 |
| CDC6 | cell division cycle 6 | 1.98 | 0.016 |
| ALKBH8 | alkB, alkylation repair homolog 8 (E. coli) | 1.98 | 0.014 |
| LARP4 | La ribonucleoprotein domain family, member 4 | 1.98 | 0.049 |
| VTI1A | vesicle transport through interaction with t-SNAREs 1A | 1.98 | 0.012 |
| CAPN13 | calpain 13 | 1.98 | 0.0007 |
| DHRS3 | dehydrogenase/reductase (SDR family) member 3 | 1.98 | 0.047 |
| TICRR | TOPBP1-interacting checkpoint and replication regulator | 1.97 | 0.018 |
| GPATCH11 | G patch domain containing 11 | 1.97 | 0.049 |
| TTK | TTK protein kinase | 1.97 | 0.007 |
| CENPE | centromere protein E, 312kDa | 1.97 | 0.010 |
| NTM | neurotrimin | 1.97 | 0.015 |
| ASAH2 | N-acylsphingosine amidohydrolase (non-lysosomal ceramidase) 2 | 1.97 | 0.033 |
| NCAPG2 | non-SMC condensin II complex, subunit G2 | 1.97 | 0.008 |
| CXCL1 | chemokine (C-X-C motif) ligand 1 (melanoma growth stimulating activity, alpha) | 1.95 | 0.025 |
| PCDHB13 | protocadherin beta 13 | 1.95 | 0.016 |
| SOSTDC1 | sclerostin domain containing 1 | 1.95 | 0.003 |
| CDK1 | cyclin-dependent kinase 1 | 1.95 | 0.019 |
| HEG1 | heart development protein with EGF-like domains 1 | 1.94 | 0.005 |
| ZWINT | ZW10 interactor, kinetochore protein | 1.94 | 0.005 |
| MIR1260B | microRNA 1260b | 1.94 | 0.018 |
| NCAPH | non-SMC condensin I complex, subunit H | 1.94 | 0.036 |
| SERPINB8 | serpin peptidase inhibitor, clade B (ovalbumin), member 8 | 1.94 | 0.003 |
| DNAJC6 | DnaJ (Hsp40) homolog, subfamily C, member 6 | 1.93 | 0.023 |
| FBXO5 | F-box protein 5 | 1.93 | 0.013 |
| MYBL1 | v-myb myeloblastosis viral oncogene homolog (avian)-like 1 | 1.93 | 0.017 |
| DDX11-AS1 | DDX11 antisense RNA 1 | 1.93 | 0.013 |
| CHAC2 | ChaC, cation transport regulator homolog 2 (E. coli) | 1.92 | 0.039 |
| TAS1R3 | taste receptor, type 1, member 3 | 1.92 | 0.029 |
| KIFC1 | kinesin family member C1 | 1.92 | 0.015 |
| SLFN12 | schlafen family member 12 | 1.92 | 0.026 |
| EBLN2 | endogenous Bornavirus-like nucleoprotein 2 | 1.91 | 0.001 |
| BICC1 | bicaudal C homolog 1 (Drosophila) | 1.91 | 0.034 |
| LATS1 | LATS, large tumor suppressor, homolog 1 (Drosophila) | 1.91 | 0.019 |
| SPAG5 | sperm associated antigen 5 | 1.91 | 0.009 |
| KIF20B | kinesin family member 20B | 1.90 | 0.002 |
| MCM4 | minichromosome maintenance complex component 4 | 1.90 | 0.004 |
| C1orf101 | chromosome 1 open reading frame 101 | 1.89 | 0.020 |
| IL9R | interleukin 9 receptor | 1.89 | 0.020 |
| POLR3B | polymerase (RNA) III (DNA directed) polypeptide B | 1.89 | 0.012 |
| COL12A1 | collagen, type XII, alpha 1 | 1.89 | 0.042 |
| AMD1 | adenosylmethionine decarboxylase 1 | 1.89 | 0.036 |
| SOX6 | SRY (sex determining region Y)-box 6 | 1.88 | 0.049 |
| HMMR | hyaluronan-mediated motility receptor (RHAMM) | 1.88 | 0.009 |
| GINS4 | GINS complex subunit 4 (Sld5 homolog) | 1.88 | 0.009 |
| KRTAP5-4 | keratin associated protein 5-4 | 1.88 | 0.020 |
| CHAC1 | ChaC, cation transport regulator homolog 1 (E. coli) | 1.88 | 0.05 |
| ATAD2 | ATPase family, AAA domain containing 2 | 1.87 | 0.033 |
| TWISTNB | TWIST neighbor | 1.86 | 0.049 |
| ZFAND4 | zinc finger, AN1-type domain 4 | 1.86 | 0.015 |
| GAN | gigaxonin | 1.86 | 0.024 |
| CXCL3 | chemokine (C-X-C motif) ligand 3 | 1.86 | 0.037 |
| MAFB | v-maf musculoaponeurotic fibrosarcoma oncogene homolog B (avian) | 1.86 | 0.005 |
| GRHL1 | grainyhead-like 1 (Drosophila) | 1.86 | 0.012 |
| RRM2 | ribonucleotide reductase M2 | 1.86 | 0.021 |
| RAPGEF6 | Rap guanine nucleotide exchange factor (GEF) 6 | 1.86 | 0.040 |
| MARS2 | methionyl-tRNA synthetase 2, mitochondrial | 1.86 | 0.041 |
| SFMBT1 | Scm-like with four mbt domains 1 | 1.86 | 0.011 |
| FBXO43 | F-box protein 43 | 1.86 | 0.023 |
| FITM2 | fat storage-inducing transmembrane protein 2 | 1.86 | 0.047 |
| LTB | lymphotoxin beta (TNF superfamily, member 3) | 1.86 | 0.033 |
| NRP1 | neuropilin 1 | 1.86 | 0.0001 |
| NRIP1 | nuclear receptor interacting protein 1 | 1.85 | 0.042 |
| SMG1P5 | smg-1 homolog, phosphatidylinositol 3-kinase-related kinase (C. elegans) pseudogene | 1.85 | 0.028 |
| PCNA | proliferating cell nuclear antigen | 1.85 | 0.023 |
| CMTR2 | FtsJ methyltransferase domain containing 1 | 1.85 | 0.05 |
| SMC4 | structural maintenance of chromosomes 4 | 1.85 | 0.022 |
| ZWILCH | zwilch kinetochore protein | 1.85 | 0.006 |
| ZNF407 | zinc finger protein 407 | 1.84 | 0.033 |
| KIF11 | kinesin family member 11 | 1.84 | 0.003 |
| TCF19 | transcription factor 19 | 1.84 | 0.010 |
| RCAN2 | regulator of calcineurin 2 | 1.84 | 0.029 |
| KIF23 | kinesin family member 23 | 1.83 | 0.022 |
| PIK3AP1 | phosphoinositide-3-kinase adaptor protein 1 | 1.83 | 0.020 |
| C21orf67 | chromosome 21 open reading frame 67 | 1.83 | 0.0004 |
| CCNA2 | cyclin A2 | 1.82 | 0.020 |
| ORC1 | origin recognition complex, subunit 1 | 1.82 | 0.007 |
| ZNF300 | zinc finger protein 300 | 1.82 | 0.020 |
| FAM83D | family with sequence similarity 83, member D | 1.82 | 0.026 |
| DHFR | dihydrofolate reductase | 1.81 | 0.008 |
| CDCA8 | cell division cycle associated 8 | 1.81 | 0.016 |
| TBC1D30 | TBC1 domain family, member 30 | 1.80 | 0.003 |
| LCTL | lactase-like | 1.80 | 0.009 |
| ENOX2 | ecto-NOX disulfide-thiol exchanger 2 | 1.80 | 0.044 |
| PHLPP2 | PH domain and leucine rich repeat protein phosphatase 2 | 1.80 | 0.037 |
| ZNF827 | zinc finger protein 827 | 1.80 | 0.018 |
| KIF20A | kinesin family member 20A | 1.80 | 0.018 |
| ASCC3 | activating signal cointegrator 1 complex subunit 3 | 1.80 | 0.046 |
| MEGF11 | multiple EGF-like-domains 11 | 1.80 | 0.0007 |
| PTPRN2 | protein tyrosine phosphatase, receptor type, N polypeptide 2 | 1.80 | 0.031 |
| FAM126A | family with sequence similarity 126, member A | 1.80 | 0.027 |
| PLK2 | polo-like kinase 2 | 1.79 | 0.042 |
| PCDH20 | protocadherin 20 | 1.79 | 0.044 |
| BMP2K | BMP2 inducible kinase | 1.79 | 0.043 |
| KLHL23 | kelch-like family member 23 | 1.79 | 0.005 |
| ZNF365 | zinc finger protein 365 | 1.79 | 0.006 |
| ITGA4 | integrin, alpha 4 (antigen CD49D, alpha 4 subunit of VLA-4 receptor) | 1.79 | 0.032 |
| ACER3 | alkaline ceramidase 3 | 1.79 | 0.003 |
| PLK4 | polo-like kinase 4 | 1.78 | 0.004 |
| DPY19L2P1 | dpy-19-like 2 pseudogene 1 (C. elegans) | 1.78 | 0.048 |
| MFAP3L | microfibrillar-associated protein 3-like | 1.78 | 0.001 |
| DSCC1 | defective in sister chromatid cohesion 1 homolog (S. cerevisiae) | 1.78 | 0.012 |
| MAD2L1 | MAD2 mitotic arrest deficient-like 1 (yeast) | 1.77 | 0.006 |
| DIAPH3 | diaphanous homolog 3 (Drosophila) | 1.77 | 0.034 |
| CTGF | connective tissue growth factor | 1.77 | 0.013 |
| LIMCH1 | LIM and calponin homology domains 1 | 1.77 | 0.009 |
| INCENP | inner centromere protein antigens 135/155kDa | 1.77 | 0.016 |
| SETD9 | SET domain containing 9 | 1.77 | 0.015 |
| UHRF1 | ubiquitin-like with PHD and ring finger domains 1 | 1.76 | 0.028 |
| GRIP1 | glutamate receptor interacting protein 1 | 1.76 | 0.0007 |
| PVRL4 | poliovirus receptor-related 4 | 1.76 | 0.011 |
| DHX9 | DEAH (Asp-Glu-Ala-His) box polypeptide 9 | 1.76 | 0.021 |
| FAM64A | family with sequence similarity 64, member A | 1.76 | 0.033 |
| MBNL2 | muscleblind-like splicing regulator 2 | 1.75 | 0.048 |
| KIF18A | kinesin family member 18A | 1.75 | 0.012 |
| HJURP | Holliday junction recognition protein | 1.75 | 0.002 |
| EFNB2 | ephrin-B2 | 1.74 | 0.014 |
| LMNB1 | lamin B1 | 1.74 | 0.013 |
| LHFP | lipoma HMGIC fusion partner | 1.74 | 0.040 |
| PKHD1 | polycystic kidney and hepatic disease 1 (autosomal recessive) | 1.74 | 0.038 |
| MYCBP2 | MYC binding protein 2, E3 ubiquitin protein ligase | 1.74 | 0.044 |
| MYO5A | myosin VA (heavy chain 12, myoxin) | 1.74 | 0.034 |
| TMPO | thymopoietin | 1.74 | 0.041 |
| RSC1A1 | regulatory solute carrier protein, family 1, member 1 | 1.74 | 0.012 |
| BHLHE40-AS1 | BHLHE40 antisense RNA 1 | 1.74 | 0.049 |
| NTRK2 | neurotrophic tyrosine kinase, receptor, type 2 | 1.73 | 0.006 |
| TLR3 | toll-like receptor 3 | 1.73 | 0.007 |
| DCBLD1 | discoidin, CUB and LCCL domain containing 1 | 1.73 | 0.039 |
| RAB11FIP2 | RAB11 family interacting protein 2 (class I) | 1.73 | 0.026 |
| PELI1 | pellino E3 ubiquitin protein ligase 1 | 1.73 | 0.043 |
| ESRRG | estrogen-related receptor gamma | 1.73 | 0.013 |
| SLMO2-ATP5E | SLMO2-ATP5E readthrough | 1.72 | 0.047 |
| SGOL2 | shugoshin-like 2 (S. pombe) | 1.72 | 0.007 |
| VNN1 | vanin 1 | 1.72 | 0.049 |
| NTN4 | netrin 4 | 1.72 | 0.033 |
| HIST1H2AC | histone cluster 1, H2ac | 1.72 | 0.018 |
| EPC2 | enhancer of polycomb homolog 2 (Drosophila) | 1.72 | 0.022 |
| CHORDC1 | cysteine and histidine-rich domain (CHORD) containing 1 | 1.72 | 0.048 |
| CCAR1 | cell division cycle and apoptosis regulator 1 | 1.72 | 0.05 |
| HIPK2 | homeodomain interacting protein kinase 2 | 1.72 | 0.046 |
| FNDC3B | fibronectin type III domain containing 3B | 1.72 | 0.040 |
| CEP76 | centrosomal protein 76kDa | 1.72 | 0.009 |
| CYR61 | cysteine-rich, angiogenic inducer, 61 | 1.72 | 0.003 |
| FOXM1 | forkhead box M1 | 1.71 | 0.018 |
| EFCAB5 | EF-hand calcium binding domain 5 | 1.71 | 0.024 |
| CRIM1 | cysteine rich transmembrane BMP regulator 1 (chordin-like) | 1.71 | 0.05 |
| STAG1 | stromal antigen 1 | 1.71 | 0.016 |
| ERO1LB | ERO1-like beta (S. cerevisiae) | 1.71 | 0.005 |
| CENPK | centromere protein K | 1.71 | 0.041 |
| KIAA1024 | KIAA1024 | 1.71 | 0.042 |
| ECE2 | endothelin converting enzyme 2 | 1.70 | 0.027 |
| PIGW | phosphatidylinositol glycan anchor biosynthesis, class W | 1.70 | 0.021 |
| CTTNBP2 | cortactin binding protein 2 | 1.70 | 0.010 |
| GPC2 | glypican 2 | 1.70 | 0.016 |
| SLC26A2 | solute carrier family 26 (sulfate transporter), member 2 | 1.70 | 0.049 |
| KPNA2 | karyopherin alpha 2 (RAG cohort 1, importin alpha 1) | 1.70 | 0.041 |
| UHRF1BP1L | UHRF1 binding protein 1-like | 1.70 | 0.031 |
| CCNB1 | cyclin B1 | 1.70 | 0.007 |
| SEMA3A | sema domain, immunoglobulin domain (Ig), short basic domain, secreted, (semaphorin) 3A | 1.70 | 0.011 |
| ACVR1C | activin A receptor, type IC | 1.70 | 0.004 |
| F11-AS1 | uncharacterized LOC285441 | 1.70 | 0.001 |
| NIPAL2 | NIPA-like domain containing 2 | 1.69 | 0.048 |
| LCA5 | Leber congenital amaurosis 5 | 1.69 | 0.045 |
| SPPL2A | signal peptide peptidase like 2A | 1.69 | 0.048 |
| FAM102B | family with sequence similarity 102, member B | 1.69 | 0.047 |
| DCUN1D1 | DCN1, defective in cullin neddylation 1, domain containing 1 (S. cerevisiae) | 1.69 | 0.049 |
| ALMS1 | Alstrom syndrome 1 | 1.69 | 0.006 |
| FBXL4 | F-box and leucine-rich repeat protein 4 | 1.69 | 0.045 |
| INPP4B | inositol polyphosphate-4-phosphatase, type II, 105kDa | 1.69 | 0.021 |
| PHACTR2 | phosphatase and actin regulator 2 | 1.69 | 0.028 |
| LZTS1 | leucine zipper, putative tumor suppressor 1 | 1.68 | 0.019 |
| TRIP13 | thyroid hormone receptor interactor 13 | 1.68 | 0.033 |
| CKAP2 | cytoskeleton associated protein 2 | 1.68 | 0.034 |
| MID1 | midline 1 (Opitz/BBB syndrome) | 1.68 | 0.002 |
| CHUK | conserved helix-loop-helix ubiquitous kinase | 1.68 | 0.028 |
| TENM4 | teneurin transmembrane protein 4 | 1.68 | 0.026 |
| IQGAP3 | IQ motif containing GTPase activating protein 3 | 1.68 | 0.011 |
| ZBTB6 | zinc finger and BTB domain containing 6 | 1.68 | 0.042 |
| HIF1A | hypoxia inducible factor 1, alpha subunit (basic helix-loop-helix transcription factor) | 1.68 | 0.019 |
| TROAP | trophinin associated protein | 1.68 | 0.034 |
| NCAPG | non-SMC condensin I complex, subunit G | 1.68 | 0.012 |
| KLHDC7A | kelch domain containing 7A | 1.68 | 0.002 |
| UBTD2 | ubiquitin domain containing 2 | 1.68 | 0.013 |
| CDC25A | cell division cycle 25A | 1.68 | 0.004 |
| G3BP1 | GTPase activating protein (SH3 domain) binding protein 1 | 1.67 | 0.023 |
| ARHGAP11A | Rho GTPase activating protein 11A | 1.67 | 0.001 |
| PDF | peptide deformylase (mitochondrial) | 1.67 | 0.003 |
| SLC31A2 | solute carrier family 31 (copper transporters), member 2 | 1.67 | 0.011 |
| CDC73 | cell division cycle 73 | 1.67 | 0.05 |
| CDCA2 | cell division cycle associated 2 | 1.67 | 0.005 |
| PINX1 | PIN2/TERF1 interacting, telomerase inhibitor 1 | 1.67 | 0.032 |
| VANGL1 | VANGL planar cell polarity protein 1 | 1.67 | 0.014 |
| DNAH5 | dynein, axonemal, heavy chain 5 | 1.66 | 0.044 |
| TNC | tenascin C | 1.66 | 0.006 |
| TMEM38B | transmembrane protein 38B | 1.66 | 0.049 |
| TEX10 | testis expressed 10 | 1.66 | 0.017 |
| LTV1 | LTV1 homolog (S. cerevisiae) | 1.66 | 0.028 |
| SETBP1 | SET binding protein 1 | 1.66 | 0.040 |
| HCFC2 | host cell factor C2 | 1.66 | 0.016 |
| HHEX | hematopoietically expressed homeobox | 1.65 | 0.015 |
| EPHA4 | EPH receptor A4 | 1.65 | 0.010 |
| PARPBP | PARP1 binding protein | 1.65 | 0.036 |
| GPD2 | glycerol-3-phosphate dehydrogenase 2 (mitochondrial) | 1.65 | 0.026 |
| ENTPD7 | ectonucleoside triphosphate diphosphohydrolase 7 | 1.65 | 0.035 |
| APC | adenomatous polyposis coli | 1.65 | 0.011 |
| WDR3 | WD repeat domain 3 | 1.65 | 0.05 |
| FEN1 | flap structure-specific endonuclease 1 | 1.65 | 0.022 |
| STS | steroid sulfatase (microsomal), isozyme S | 1.65 | 0.026 |
| QSER1 | glutamine and serine rich 1 | 1.64 | 0.030 |
| B3GALT5 | UDP-Gal:betaGlcNAc beta 1,3-galactosyltransferase, polypeptide 5 | 1.64 | 0.025 |
| MELK | maternal embryonic leucine zipper kinase | 1.64 | 0.004 |
| LRCH3 | leucine-rich repeats and calponin homology (CH) domain containing 3 | 1.64 | 0.031 |
| GPAM | glycerol-3-phosphate acyltransferase, mitochondrial | 1.64 | 0.032 |
| NOP56 | NOP56 ribonucleoprotein | 1.64 | 0.010 |
| ZNF778 | zinc finger protein 778 | 1.64 | 0.015 |
| ZC2HC1C | zinc finger, C2HC-type containing 1C | 1.64 | 0.021 |
| DCAF13 | DDB1 and CUL4 associated factor 13 | 1.64 | 0.024 |
| MAP3K1 | mitogen-activated protein kinase kinase kinase 1, E3 ubiquitin protein ligase | 1.64 | 0.029 |
| ZDHHC20 | zinc finger, DHHC-type containing 20 | 1.64 | 0.027 |
| CALCRL | calcitonin receptor-like | 1.63 | 0.008 |
| DCK | deoxycytidine kinase | 1.63 | 0.046 |
| SYNCRIP | synaptotagmin binding, cytoplasmic RNA interacting protein | 1.63 | 0.043 |
| EFR3A | EFR3 homolog A (S. cerevisiae) | 1.63 | 0.038 |
| CDC20 | cell division cycle 20 | 1.63 | 0.040 |
| PRIM2 | primase, DNA, polypeptide 2 (58kDa) | 1.63 | 0.048 |
| MGA | MGA, MAX dimerization protein | 1.63 | 0.045 |
| HS2ST1 | heparan sulfate 2-O-sulfotransferase 1 | 1.63 | 0.028 |
| SGMS1 | sphingomyelin synthase 1 | 1.62 | 0.010 |
| CCNF | cyclin F | 1.62 | 0.039 |
| ERAP2 | endoplasmic reticulum aminopeptidase 2 | 1.62 | 0.027 |
| BCLAF1 | BCL2-associated transcription factor 1 | 1.62 | 0.029 |
| MCM2 | minichromosome maintenance complex component 2 | 1.62 | 0.025 |
| MKL2 | MKL/myocardin-like 2 | 1.62 | 0.028 |
| ZNF326 | zinc finger protein 326 | 1.62 | 0.032 |
| EXOC6 | exocyst complex component 6 | 1.62 | 0.044 |
| POP1 | processing of precursor 1, ribonuclease P/MRP subunit (S. cerevisiae) | 1.62 | 0.029 |
| NDST1 | N-deacetylase/N-sulfotransferase (heparan glucosaminyl) 1 | 1.62 | 0.025 |
| KIAA1462 | KIAA1462 | 1.61 | 0.005 |
| NOP2 | NOP2 nucleolar protein | 1.61 | 0.022 |
| KGFLP1 | fibroblast growth factor 7 pseudogene | 1.61 | 0.026 |
| NSUN3 | NOP2/Sun domain family, member 3 | 1.61 | 0.017 |
| AP5M1 | adaptor-related protein complex 5, mu 1 subunit | 1.61 | 0.020 |
| DHX36 | DEAH (Asp-Glu-Ala-His) box polypeptide 36 | 1.61 | 0.033 |
| OAS3 | 2'-5'-oligoadenylate synthetase 3, 100kDa | 1.61 | 0.043 |
| NRG1 | neuregulin 1 | 1.61 | 0.026 |
| TGFBR2 | transforming growth factor, beta receptor II (70/80kDa) | 1.61 | 0.012 |
| ITPR2 | inositol 1,4,5-trisphosphate receptor, type 2 | 1.61 | 0.020 |
| TRANK1 | tetratricopeptide repeat and ankyrin repeat containing 1 | 1.61 | 0.0004 |
| PEG10 | paternally expressed 10 | 1.61 | 0.025 |
| ZNF530 | zinc finger protein 530 | 1.61 | 0.012 |
| CEP57L1 | centrosomal protein 57kDa-like 1 | 1.61 | 0.023 |
| NIN | ninein (GSK3B interacting protein) | 1.61 | 0.046 |
| DOCK3 | dedicator of cytokinesis 3 | 1.61 | 0.010 |
| MBNL3 | muscleblind-like splicing regulator 3 | 1.61 | 0.029 |
| CSRNP3 | cysteine-serine-rich nuclear protein 3 | 1.60 | 0.044 |
| KLHL14 | kelch-like family member 14 | 1.60 | 0.010 |
| RECQL4 | RecQ protein-like 4 | 1.60 | 0.028 |
| KIAA1468 | KIAA1468 | 1.60 | 0.031 |
| SMC1A | structural maintenance of chromosomes 1A | 1.60 | 0.042 |
| BIRC3 | baculoviral IAP repeat containing 3 | 1.60 | 0.040 |
| BAZ1A | bromodomain adjacent to zinc finger domain, 1A | 1.60 | 0.040 |
| ERVMER34-1 | endogenous retrovirus group MER34, member 1 | 1.60 | 0.020 |
| RNF144B | ring finger protein 144B | 1.60 | 0.005 |
| CBX2 | chromobox homolog 2 | 1.60 | 0.024 |
| ZNF485 | zinc finger protein 485 | 1.60 | 0.032 |
| ABCB1 | ATP-binding cassette, sub-family B (MDR/TAP), member 1 | 1.60 | 0.020 |
| THBS1 | thrombospondin 1 | 1.60 | 0.045 |
| IFI16 | interferon, gamma-inducible protein 16 | 1.60 | 0.008 |
| GINS3 | GINS complex subunit 3 (Psf3 homolog) | 1.60 | 0.004 |
| TMEM2 | transmembrane protein 2 | 1.60 | 0.038 |
| INHBB | inhibin, beta B | 1.60 | 0.030 |
| MBNL1 | muscleblind-like splicing regulator 1 | 1.60 | 0.048 |
| USP31 | ubiquitin specific peptidase 31 | 1.59 | 0.038 |
| ECT2 | epithelial cell transforming sequence 2 oncogene | 1.59 | 0.031 |
| SCYL2 | SCY1-like 2 (S. cerevisiae) | 1.59 | 0.044 |
| TRIM33 | tripartite motif containing 33 | 1.59 | 0.05 |
| TRIP11 | thyroid hormone receptor interactor 11 | 1.59 | 0.039 |
| ATL3 | atlastin GTPase 3 | 1.59 | 0.039 |
| TNFAIP2 | tumor necrosis factor, alpha-induced protein 2 | 1.58 | 0.006 |
| PLEK | pleckstrin | 1.58 | 0.021 |
| BUB1 | BUB1 mitotic checkpoint serine/threonine kinase | 1.58 | 0.003 |
| ITGA1 | integrin, alpha 1 | 1.58 | 0.003 |
| TMEM178B | transmembrane protein 178B | 1.58 | 0.037 |
| SAMHD1 | SAM domain and HD domain 1 | 1.58 | 0.040 |
| ZNF319 | zinc finger protein 319 | 1.58 | 0.007 |
| LIN54 | lin-54 homolog (C. elegans) | 1.58 | 0.023 |
| SLC38A1 | solute carrier family 38, member 1 | 1.58 | 0.034 |
| RAPGEF2 | Rap guanine nucleotide exchange factor (GEF) 2 | 1.58 | 0.044 |
| TCF12 | transcription factor 12 | 1.58 | 0.036 |
| TNS3 | tensin 3 | 1.58 | 0.030 |
| PLXNC1 | plexin C1 | 1.58 | 0.007 |
| SFTA1P | surfactant associated 1, pseudogene | 1.58 | 0.028 |
| SLC25A46 | solute carrier family 25, member 46 | 1.57 | 0.022 |
| LIN9 | lin-9 homolog (C. elegans) | 1.57 | 0.018 |
| ANKRD44 | ankyrin repeat domain 44 | 1.57 | 0.010 |
| ABHD5 | abhydrolase domain containing 5 | 1.57 | 0.003 |
| NRDE2 | NRDE-2, necessary for RNA interference, domain containing | 1.57 | 0.041 |
| TTC30A | tetratricopeptide repeat domain 30A | 1.57 | 0.034 |
| ING3 | inhibitor of growth family, member 3 | 1.57 | 0.028 |
| PKMYT1 | protein kinase, membrane associated tyrosine/threonine 1 | 1.57 | 0.018 |
| KCTD12 | potassium channel tetramerisation domain containing 12 | 1.57 | 0.003 |
| LYPD6 | LY6/PLAUR domain containing 6 | 1.57 | 0.014 |
| ATP9B | ATPase, class II, type 9B | 1.57 | 0.009 |
| MED1 | mediator complex subunit 1 | 1.57 | 0.026 |
| MCM3 | minichromosome maintenance complex component 3 | 1.57 | 0.007 |
| GLIS3 | GLIS family zinc finger 3 | 1.57 | 0.026 |
| IARS | isoleucyl-tRNA synthetase | 1.57 | 0.021 |
| TRIM31 | tripartite motif containing 31 | 1.56 | 0.038 |
| TP53RK | TP53 regulating kinase | 1.56 | 0.035 |
| CYP2U1 | cytochrome P450, family 2, subfamily U, polypeptide 1 | 1.56 | 0.038 |
| TYMS | thymidylate synthetase | 1.56 | 0.005 |
| SPRY3 | sprouty homolog 3 (Drosophila) | 1.56 | 0.042 |
| USP16 | ubiquitin specific peptidase 16 | 1.56 | 0.012 |
| MAP1B | microtubule-associated protein 1B | 1.56 | 0.036 |
| GORAB | golgin, RAB6-interacting | 1.56 | 0.020 |
| LRIG1 | leucine-rich repeats and immunoglobulin-like domains 1 | 1.56 | 0.039 |
| ADAM28 | ADAM metallopeptidase domain 28 | 1.55 | 0.006 |
| NCBP1 | nuclear cap binding protein subunit 1, 80kDa | 1.55 | 0.033 |
| CP | ceruloplasmin (ferroxidase) | 1.55 | 0.008 |
| KDM8 | lysine (K)-specific demethylase 8 | 1.55 | 0.008 |
| ZDHHC13 | zinc finger, DHHC-type containing 13 | 1.55 | 0.043 |
| ZNF345 | zinc finger protein 345 | 1.55 | 0.036 |
| TRAK2 | trafficking protein, kinesin binding 2 | 1.55 | 0.022 |
| SYNE3 | spectrin repeat containing, nuclear envelope family member 3 | 1.55 | 0.032 |
| RACGAP1 | Rac GTPase activating protein 1 | 1.55 | 0.017 |
| RFK | riboflavin kinase | 1.55 | 0.021 |
| PCYT1A | phosphate cytidylyltransferase 1, choline, alpha | 1.55 | 0.028 |
| SPDL1 | spindle apparatus coiled-coil protein 1 | 1.55 | 0.040 |
| CIT | citron (rho-interacting, serine/threonine kinase 21) | 1.55 | 0.019 |
| SDPR | serum deprivation response | 1.55 | 0.005 |
| GALNT10 | UDP-N-acetyl-alpha-D-galactosamine:polypeptide N-acetylgalactosaminyltransferase 10 (GalNAc-T10) | 1.55 | 0.002 |
| EPHB2 | EPH receptor B2 | 1.55 | 0.005 |
| GMFB | glia maturation factor, beta | 1.54 | 0.032 |
| ARID2 | AT rich interactive domain 2 (ARID, RFX-like) | 1.54 | 0.006 |
| SNHG15 | small nucleolar RNA host gene 15 (non-protein coding) | 1.54 | 0.010 |
| LYAR | Ly1 antibody reactive | 1.54 | 0.027 |
| MALT1 | mucosa associated lymphoid tissue lymphoma translocation gene 1 | 1.54 | 0.012 |
| CROT | carnitine O-octanoyltransferase | 1.54 | 0.023 |
| NOLC1 | nucleolar and coiled-body phosphoprotein 1 | 1.54 | 0.049 |
| LRRCC1 | leucine rich repeat and coiled-coil centrosomal protein 1 | 1.54 | 0.032 |
| ZNF624 | zinc finger protein 624 | 1.54 | 0.022 |
| TMEM199 | transmembrane protein 199 | 1.54 | 0.011 |
| PPP6R3 | protein phosphatase 6, regulatory subunit 3 | 1.54 | 0.015 |
| NOL11 | nucleolar protein 11 | 1.54 | 0.036 |
| RNF219 | ring finger protein 219 | 1.54 | 0.042 |
| SPAG1 | sperm associated antigen 1 | 1.54 | 0.0002 |
| PHF20L1 | PHD finger protein 20-like 1 | 1.54 | 0.046 |
| MTOR | mechanistic target of rapamycin (serine/threonine kinase) | 1.54 | 0.031 |
| RNF219-AS1 | RNF219 antisense RNA 1 | 1.54 | 0.027 |
| MIPEPP3 | mitochondrial intermediate peptidase pseudogene 3 | 1.54 | 0.008 |
| PPIP5K2 | diphosphoinositol pentakisphosphate kinase 2 | 1.54 | 0.021 |
| NLGN1 | neuroligin 1 | 1.53 | 0.028 |
| C3 | complement component 3 | 1.53 | 0.027 |
| THAP1 | THAP domain containing, apoptosis associated protein 1 | 1.53 | 0.004 |
| CYTH3 | cytohesin 3 | 1.53 | 0.025 |
| ZNF625 | zinc finger protein 625 | 1.53 | 0.037 |
| LBH | limb bud and heart development | 1.53 | 0.009 |
| ARL15 | ADP-ribosylation factor-like 15 | 1.53 | 0.047 |
| SLX4IP | SLX4 interacting protein | 1.53 | 0.002 |
| PDS5B | PDS5, regulator of cohesion maintenance, homolog B (S. cerevisiae) | 1.53 | 0.023 |
| PPP2R1B | protein phosphatase 2, regulatory subunit A, beta | 1.53 | 0.046 |
| SRSF3 | serine/arginine-rich splicing factor 3 | 1.53 | 0.017 |
| NCEH1 | neutral cholesterol ester hydrolase 1 | 1.53 | 0.029 |
| CGGBP1 | CGG triplet repeat binding protein 1 | 1.53 | 0.036 |
| H2AFX | H2A histone family, member X | 1.52 | 0.030 |
| RAET1K | retinoic acid early transcript 1K pseudogene | 1.52 | 0.002 |
| G2E3 | G2/M-phase specific E3 ubiquitin protein ligase | 1.52 | 0.049 |
| FSTL1 | follistatin-like 1 | 1.52 | 0.007 |
| ST5 | suppression of tumorigenicity 5 | 1.52 | 0.023 |
| OSBPL11 | oxysterol binding protein-like 11 | 1.52 | 0.037 |
| ZCCHC10 | zinc finger, CCHC domain containing 10 | 1.52 | 0.002 |
| TTN-AS1 | TTN antisense RNA 1 | 1.52 | 0.024 |
| LOC100132356 | uncharacterized LOC100132356 | 1.52 | 0.004 |
| EIF4G2 | eukaryotic translation initiation factor 4 gamma, 2 | 1.52 | 0.025 |
| ATF2 | activating transcription factor 2 | 1.52 | 0.014 |
| FAM212B | family with sequence similarity 212, member B | 1.52 | 0.017 |
| TIMELESS | timeless circadian clock | 1.51 | 0.001 |
| TRAM1 | translocation associated membrane protein 1 | 1.51 | 0.004 |
| PPARGC1A | peroxisome proliferator-activated receptor gamma, coactivator 1 alpha | 1.51 | 0.009 |
| ZNF420 | zinc finger protein 420 | 1.51 | 0.016 |
| CDC27 | cell division cycle 27 | 1.51 | 0.046 |
| SNX13 | sorting nexin 13 | 1.51 | 0.028 |
| TWSG1 | twisted gastrulation homolog 1 (Drosophila) | 1.51 | 0.008 |
| MFAP3 | microfibrillar-associated protein 3 | 1.51 | 0.014 |
| NUDT6 | nudix (nucleoside diphosphate linked moiety X)-type motif 6 | 1.51 | 0.05 |
| TAF5 | TAF5 RNA polymerase II, TATA box binding protein (TBP)-associated factor, 100kDa | 1.51 | 0.010 |
| PTCHD3P1 | patched domain containing 3 pseudogene 1 | 1.51 | 0.044 |
| TMEM168 | transmembrane protein 168 | 1.51 | 0.038 |
| ERCC4 | excision repair cross-complementing rodent repair deficiency, complementation group 4 | 1.51 | 0.045 |
| FAM81A | family with sequence similarity 81, member A | 1.51 | 0.028 |
| NETO2 | neuropilin (NRP) and tolloid (TLL)-like 2 | 1.51 | 0.032 |
| TMEM217 | transmembrane protein 217 | 1.51 | 0.043 |
| ZC3H12A | zinc finger CCCH-type containing 12A | 1.51 | 0.006 |
| SORT1 | sortilin 1 | 1.51 | 0.0006 |
| KLHL5 | kelch-like family member 5 | 1.51 | 0.041 |
| PRC1 | protein regulator of cytokinesis 1 | 1.51 | 0.014 |
| TACC3 | transforming, acidic coiled-coil containing protein 3 | 1.51 | 0.020 |
| SLC33A1 | solute carrier family 33 (acetyl-CoA transporter), member 1 | 1.51 | 0.049 |
| RPGRIP1L | RPGRIP1-like | 1.51 | 0.042 |
| ZNFX1 | zinc finger, NFX1-type containing 1 | 1.51 | 0.022 |
| FKRP | fukutin related protein | 1.50 | 0.004 |
| FEZ1 | fasciculation and elongation protein zeta 1 (zygin I) | 1.50 | 0.015 |
| PRTN3 | proteinase 3 | 0.06 | 0.041 |
| DEGS2 | delta(4)-desaturase, sphingolipid 2 | 0.06 | 0.010 |
| LMO1 | LIM domain only 1 (rhombotin 1) | 0.07 | 0.008 |
| NXF3 | nuclear RNA export factor 3 | 0.09 | 0.010 |
| PSD2 | pleckstrin and Sec7 domain containing 2 | 0.10 | 0.035 |
| FCER1G | Fc fragment of IgE, high affinity I, receptor for; gamma polypeptide | 0.11 | 0.044 |
| NXNL1 | nucleoredoxin-like 1 | 0.11 | 0.042 |
| TEX29 | testis expressed 29 | 0.12 | 0.026 |
| TRIM63 | tripartite motif containing 63, E3 ubiquitin protein ligase | 0.12 | 0.038 |
| TXNDC2 | thioredoxin domain containing 2 (spermatozoa) | 0.13 | 0.006 |
| PTGS1 | prostaglandin-endoperoxide synthase 1 (prostaglandin G/H synthase and cyclooxygenase) | 0.14 | 0.025 |
| C11orf86 | chromosome 11 open reading frame 86 | 0.15 | 0.040 |
| ISM2 | isthmin 2 homolog (zebrafish) | 0.15 | 0.003 |
| MRPL23-AS1 | MRPL23 antisense RNA 1 | 0.16 | 0.05 |
| CYP26A1 | cytochrome P450, family 26, subfamily A, polypeptide 1 | 0.17 | 0.012 |
| DUOX2 | dual oxidase 2 | 0.17 | 0.038 |
| MOGAT3 | monoacylglycerol O-acyltransferase 3 | 0.17 | 0.042 |
| GNAT1 | guanine nucleotide binding protein (G protein), alpha transducing activity polypeptide 1 | 0.20 | 0.020 |
| FAM83A | family with sequence similarity 83, member A | 0.20 | 0.040 |
| ARMC12 | armadillo repeat containing 12 | 0.20 | 0.018 |
| ANGPTL4 | angiopoietin-like 4 | 0.20 | 0.019 |
| RNU6-35P | RNA, U6 small nuclear 35 | 0.21 | 0.040 |
| CSPG4 | chondroitin sulfate proteoglycan 4 | 0.21 | 0.028 |
| PSORS1C2 | psoriasis susceptibility 1 candidate 2 | 0.21 | 0.009 |
| ACP5 | acid phosphatase 5, tartrate resistant | 0.21 | 0.0004 |
| DDX11L9 | DEAD/H (Asp-Glu-Ala-Asp/His) box helicase 11 like 9 | 0.21 | 0.037 |
| SLC2A5 | solute carrier family 2 (facilitated glucose/fructose transporter), member 5 | 0.22 | 0.016 |
| EEF1A2 | eukaryotic translation elongation factor 1 alpha 2 | 0.22 | 0.013 |
| CIB4 | calcium and integrin binding family member 4 | 0.22 | 0.006 |
| MIR205 | microRNA 205 | 0.22 | 0.027 |
| IL2RG | interleukin 2 receptor, gamma | 0.22 | 0.015 |
| CHIA | chitinase, acidic | 0.23 | 0.049 |
| DHDH | dihydrodiol dehydrogenase (dimeric) | 0.23 | 0.042 |
| EPX | eosinophil peroxidase | 0.24 | 0.008 |
| PLA2G4B | phospholipase A2, group IVB (cytosolic) | 0.25 | 0.026 |
| NUDT16P1 | nudix (nucleoside diphosphate linked moiety X)-type motif 16 pseudogene 1 | 0.26 | 0.044 |
| TNXB | tenascin XB | 0.26 | 0.047 |
| RNA28S5 | RNA, 28S ribosomal 5 | 0.27 | 0.002 |
| MLPH | melanophilin | 0.28 | 0.042 |
| G0S2 | G0/G1switch 2 | 0.28 | 0.007 |
| CATIP | chromosome 2 open reading frame 62 | 0.28 | 0.039 |
| STRA8 | stimulated by retinoic acid 8 | 0.28 | 0.034 |
| HSPA6 | heat shock 70kDa protein 6 (HSP70B') | 0.29 | 0.038 |
| TMEM74B | transmembrane protein 74B | 0.29 | 0.0002 |
| SNORA44 | small nucleolar RNA, H/ACA box 44 | 0.29 | 0.021 |
| ZPBP2 | zona pellucida binding protein 2 | 0.30 | 0.020 |
| SHANK1 | SH3 and multiple ankyrin repeat domains 1 | 0.30 | 0.020 |
| TRPC7 | transient receptor potential cation channel, subfamily C, member 7 | 0.30 | 0.034 |
| AQP7P3 | aquaporin 7 pseudogene 3 | 0.30 | 0.039 |
| KRTAP5-1 | keratin associated protein 5-1 | 0.31 | 0.0001 |
| WFDC3 | WAP four-disulfide core domain 3 | 0.31 | 0.013 |
| RAB41 | RAB41, member RAS oncogene family | 0.31 | 0.011 |
| LEMD1 | LEM domain containing 1 | 0.31 | 0.017 |
| LINC00158 | long intergenic non-protein coding RNA 158 | 0.32 | 0.009 |
| EFEMP2 | EGF containing fibulin-like extracellular matrix protein 2 | 0.32 | 0.013 |
| NDRG1 | N-myc downstream regulated 1 | 0.32 | 0.023 |
| CACNA2D2 | calcium channel, voltage-dependent, alpha 2/delta subunit 2 | 0.32 | 0.013 |
| ANG | angiogenin, ribonuclease, RNase A family, 5 | 0.33 | 0.015 |
| S100A14 | S100 calcium binding protein A14 | 0.33 | 0.004 |
| S100P | S100 calcium binding protein P | 0.33 | 0.026 |
| TTBK1 | tau tubulin kinase 1 | 0.33 | 0.035 |
| MMP28 | matrix metallopeptidase 28 | 0.34 | 0.035 |
| CDK18 | cyclin-dependent kinase 18 | 0.34 | 0.009 |
| SPOCD1 | SPOC domain containing 1 | 0.34 | 0.004 |
| CYP4F30P | cytochrome P450, family 4, subfamily F, polypeptide 30, pseudogene | 0.34 | 0.029 |
| SYT7 | synaptotagmin VII | 0.35 | 0.013 |
| CFP | complement factor properdin | 0.35 | 0.035 |
| CLDND2 | claudin domain containing 2 | 0.35 | 0.028 |
| SAPCD1 | suppressor APC domain containing 1 | 0.36 | 0.045 |
| PROCA1 | protein interacting with cyclin A1 | 0.36 | 0.041 |
| ENO2 | enolase 2 (gamma, neuronal) | 0.36 | 0.024 |
| HILPDA | hypoxia inducible lipid droplet-associated | 0.37 | 0.005 |
| HCG27 | HLA complex group 27 (non-protein coding) | 0.37 | 0.027 |
| SCGB2A2 | secretoglobin, family 2A, member 2 | 0.38 | 0.047 |
| CAPN14 | calpain 14 | 0.38 | 0.004 |
| TDRD6 | tudor domain containing 6 | 0.38 | 0.023 |
| CD68 | CD68 molecule | 0.39 | 0.003 |
| VILL | villin-like | 0.39 | 0.005 |
| LOC101241902 | chromosome 4 open reading frame 46 pseudogene | 0.39 | 0.039 |
| SLC38A5 | solute carrier family 38, member 5 | 0.39 | 0.005 |
| PRSS53 | protease, serine, 53 | 0.39 | 0.008 |
| FTH1P18 | ferritin, heavy polypeptide 1 pseudogene 18 | 0.39 | 0.040 |
| PLXNB3 | plexin B3 | 0.39 | 0.006 |
| NDRG4 | NDRG family member 4 | 0.39 | 0.035 |
| ANKRD37 | ankyrin repeat domain 37 | 0.39 | 0.008 |
| TRIM29 | tripartite motif containing 29 | 0.40 | 0.005 |
| IQSEC3 | IQ motif and Sec7 domain 3 | 0.40 | 0.049 |
| GIPR | gastric inhibitory polypeptide receptor | 0.40 | 0.044 |
| SPDYE1 | speedy homolog E1 (Xenopus laevis) | 0.41 | 0.036 |
| NR4A1 | nuclear receptor subfamily 4, group A, member 1 | 0.41 | 0.008 |
| LINGO2 | leucine rich repeat and Ig domain containing 2 | 0.41 | 0.010 |
| TMEM178A | transmembrane protein 178A | 0.41 | 0.012 |
| GABRE | gamma-aminobutyric acid (GABA) A receptor, epsilon | 0.41 | 0.009 |
| PRODH | proline dehydrogenase (oxidase) 1 | 0.42 | 0.007 |
| IGFBP3 | insulin-like growth factor binding protein 3 | 0.42 | 0.013 |
| C12orf79 | uncharacterized LOC256021 | 0.42 | 0.019 |
| PLCH2 | phospholipase C, eta 2 | 0.42 | 0.029 |
| SUSD2 | sushi domain containing 2 | 0.42 | 0.022 |
| TREH | trehalase (brush-border membrane glycoprotein) | 0.42 | 0.010 |
| SIDT1 | SID1 transmembrane family, member 1 | 0.43 | 0.035 |
| FAM229A | family with sequence similarity 229, member A | 0.43 | 0.007 |
| PCOLCE2 | procollagen C-endopeptidase enhancer 2 | 0.43 | 0.027 |
| LENG9 | leukocyte receptor cluster (LRC) member 9 | 0.43 | 0.028 |
| CRABP2 | cellular retinoic acid binding protein 2 | 0.43 | 0.011 |
| AIM1L | absent in melanoma 1-like | 0.43 | 0.020 |
| GDPD3 | glycerophosphodiester phosphodiesterase domain containing 3 | 0.43 | 0.044 |
| LINC00202-1 | long intergenic non-protein coding RNA 202-1 | 0.43 | 0.047 |
| LAPTM5 | lysosomal protein transmembrane 5 | 0.44 | 0.001 |
| LOC154761 | family with sequence similarity 115, member C pseudogene | 0.44 | 0.029 |
| HLA-F-AS1 | HLA-F antisense RNA 1 | 0.44 | 0.033 |
| HES7 | hairy and enhancer of split 7 (Drosophila) | 0.44 | 0.006 |
| MYO7B | myosin VIIB | 0.44 | 0.019 |
| GNAS-AS1 | GNAS antisense RNA 1 | 0.44 | 0.0005 |
| FAM132A | family with sequence similarity 132, member A | 0.44 | 0.048 |
| C1S | complement component 1, s subcomponent | 0.44 | 0.008 |
| PKLR | pyruvate kinase, liver and RBC | 0.44 | 0.041 |
| RNASE4 | ribonuclease, RNase A family, 4 | 0.44 | 0.0001 |
| SLPI | secretory leukocyte peptidase inhibitor | 0.44 | 0.046 |
| C16orf74 | chromosome 16 open reading frame 74 | 0.45 | 0.039 |
| C9orf173 | chromosome 9 open reading frame 173 | 0.45 | 0.007 |
| MYH16 | myosin, heavy chain 16 pseudogene | 0.45 | 0.022 |
| SERPINE1 | serpin peptidase inhibitor, clade E (nexin, plasminogen activator inhibitor type 1), member 1 | 0.45 | 0.0005 |
| NANOGNB | NANOG neighbor homeobox | 0.46 | 0.002 |
| NOG | noggin | 0.46 | 0.048 |
| FA2H | fatty acid 2-hydroxylase | 0.46 | 0.015 |
| CRYAB | crystallin, alpha B | 0.46 | 0.008 |
| KIF17 | kinesin family member 17 | 0.46 | 0.015 |
| CNIH2 | cornichon homolog 2 (Drosophila) | 0.46 | 0.043 |
| C1QL4 | complement component 1, q subcomponent-like 4 | 0.47 | 0.004 |
| PLAUR | plasminogen activator, urokinase receptor | 0.47 | 0.002 |
| RBAK-RBAKDN | RBAK-LOC389458 readthrough | 0.47 | 0.021 |
| ZNF404 | zinc finger protein 404 | 0.47 | 0.016 |
| HLA-J | major histocompatibility complex, class I, J (pseudogene) | 0.47 | 0.027 |
| TMEM207 | transmembrane protein 207 | 0.48 | 0.044 |
| PADI1 | peptidyl arginine deiminase, type I | 0.48 | 0.010 |
| AQP7P1 | aquaporin 7 pseudogene 1 | 0.48 | 0.044 |
| RTBDN | retbindin | 0.48 | 0.015 |
| FZD10 | frizzled family receptor 10 | 0.48 | 0.023 |
| ANXA2P2 | annexin A2 pseudogene 2 | 0.49 | 0.021 |
| TMEM255B | transmembrane protein 255B | 0.49 | 0.011 |
| LOC100506469 | uncharacterized LOC100506469 | 0.49 | 0.008 |
| TMEM91 | transmembrane protein 91 | 0.49 | 0.047 |
| PRSS8 | protease, serine, 8 | 0.49 | 0.002 |
| MT1L | metallothionein 1L (gene/pseudogene) | 0.49 | 0.039 |
| SEMA3B | sema domain, immunoglobulin domain (Ig), short basic domain, secreted, (semaphorin) 3B | 0.49 | 0.015 |
| SSTR5 | somatostatin receptor 5 | 0.49 | 0.005 |
| FAM74A1 | family with sequence similarity 74, member A1 | 0.49 | 0.038 |
| MT1X | metallothionein 1X | 0.49 | 0.038 |
| DPEP1 | dipeptidase 1 (renal) | 0.49 | 0.042 |
| TBX6 | T-box 6 | 0.49 | 0.018 |
| LDHD | lactate dehydrogenase D | 0.49 | 0.011 |
| SNORD103A | small nucleolar RNA, C/D box 103A | 0.49 | 0.038 |
| SNORD103B | small nucleolar RNA, C/D box 103B | 0.49 | 0.038 |
| THEMIS2 | thymocyte selection associated family member 2 | 0.50 | 0.006 |
| ADAMTSL4 | ADAMTS-like 4 | 0.50 | 0.005 |
| KCND1 | potassium voltage-gated channel, Shal-related subfamily, member 1 | 0.50 | 0.022 |
| FZD10-AS1 | uncharacterized LOC440119 | 0.50 | 0.022 |
| LINC00607 | long intergenic non-protein coding RNA 607 | 0.50 | 0.006 |
| CYGB | cytoglobin | 0.50 | 0.032 |
| TBC1D3P5 | TBC1 domain family, member 3 pseudogene 5 | 0.50 | 0.034 |
| ZNF385C | zinc finger protein 385C | 0.50 | 0.049 |
| TP53TG5 | TP53 target 5 | 0.50 | 0.002 |
| F3 | coagulation factor III (thromboplastin, tissue factor) | 0.51 | 0.013 |
| DICER1-AS1 | DICER1 antisense RNA 1 | 0.51 | 0.019 |
| MIR155HG | MIR155 host gene (non-protein coding) | 0.51 | 0.018 |
| LOC100129722 | uncharacterized LOC100129722 | 0.51 | 0.024 |
| CKB | creatine kinase, brain | 0.51 | 0.005 |
| JAM2 | junctional adhesion molecule 2 | 0.51 | 0.006 |
| AKR1C1 | aldo-keto reductase family 1, member C1 | 0.51 | 0.017 |
| TGM5 | transglutaminase 5 | 0.51 | 0.0008 |
| C10orf10 | chromosome 10 open reading frame 10 | 0.51 | 0.027 |
| KRT24 | keratin 24 | 0.51 | 0.021 |
| FAM95C | family with sequence similarity 95, member C | 0.51 | 0.036 |
| NAPSA | napsin A aspartic peptidase | 0.51 | 0.035 |
| PLA2G4F | phospholipase A2, group IVF | 0.51 | 0.023 |
| SLCO4A1 | solute carrier organic anion transporter family, member 4A1 | 0.51 | 0.033 |
| OXCT2 | 3-oxoacid CoA transferase 2 | 0.52 | 0.047 |
| DCN | decorin | 0.52 | 0.045 |
| LOC286297 | uncharacterized LOC286297 | 0.52 | 0.035 |
| ESPN | espin | 0.52 | 0.024 |
| HSD17B2 | hydroxysteroid (17-beta) dehydrogenase 2 | 0.52 | 0.029 |
| PPIAP30 | peptidylprolyl isomerase A (cyclophilin A) pseudogene 30 | 0.52 | 0.031 |
| LINC00899 | uncharacterized LOC100271722 | 0.52 | 0.037 |
| DUSP1 | dual specificity phosphatase 1 | 0.52 | 0.021 |
| DUSP5P1 | dual specificity phosphatase 5 pseudogene 1 | 0.52 | 0.012 |
| LGALS1 | lectin, galactoside-binding, soluble, 1 | 0.52 | 0.037 |
| ZNF540 | zinc finger protein 540 | 0.52 | 0.044 |
| NAT6 | N-acetyltransferase 6 (GCN5-related) | 0.52 | 0.017 |
| LINC00282 | long intergenic non-protein coding RNA 282 | 0.53 | 0.049 |
| SAP25 | Sin3A-associated protein, 25kDa | 0.53 | 0.036 |
| INHA | inhibin, alpha | 0.53 | 0.032 |
| SERPINE2 | serpin peptidase inhibitor, clade E (nexin, plasminogen activator inhibitor type 1), member 2 | 0.53 | 0.006 |
| RASA4 | RAS p21 protein activator 4 | 0.53 | 0.041 |
| KIAA1549L | KIAA1549-like | 0.53 | 0.032 |
| KCNK3 | potassium channel, subfamily K, member 3 | 0.53 | 0.036 |
| KCNK7 | potassium channel, subfamily K, member 7 | 0.53 | 0.049 |
| GRIN3B | glutamate receptor, ionotropic, N-methyl-D-aspartate 3B | 0.53 | 0.039 |
| FIBCD1 | fibrinogen C domain containing 1 | 0.53 | 0.002 |
| ROCK1P1 | Rho-associated, coiled-coil containing protein kinase 1 pseudogene 1 | 0.53 | 0.048 |
| ATG16L2 | autophagy related 16-like 2 (S. cerevisiae) | 0.53 | 0.005 |
| NEAT1 | nuclear paraspeckle assembly transcript 1 (non-protein coding) | 0.54 | 0.043 |
| TBC1D3F | TBC1 domain family, member 3F | 0.54 | 0.027 |
| HYPK | huntingtin interacting protein K | 0.54 | 0.021 |
| SLC30A2 | solute carrier family 30 (zinc transporter), member 2 | 0.54 | 0.008 |
| SYP | synaptophysin | 0.54 | 0.024 |
| CLDN10-AS1 | CLDN10 antisense RNA 1 | 0.54 | 0.037 |
| HYKK | aminoglycoside phosphotransferase domain containing 1 | 0.54 | 0.029 |
| PNMAL1 | paraneoplastic Ma antigen family-like 1 | 0.54 | 0.026 |
| SCXB | scleraxis homolog B (mouse) | 0.55 | 0.011 |
| - | scleraxis homolog A (mouse) | 0.55 | 0.011 |
| LINC00685 | long intergenic non-protein coding RNA 685 | 0.55 | 0.009 |
| OLFML3 | olfactomedin-like 3 | 0.55 | 0.038 |
| IZUMO4 | IZUMO family member 4 | 0.55 | 0.002 |
| PPP1R3C | protein phosphatase 1, regulatory subunit 3C | 0.55 | 0.018 |
| HMGN2P46 | high mobility group nucleosomal binding domain 2 pseudogene 46 | 0.55 | 0.025 |
| GSTM2 | glutathione S-transferase mu 2 (muscle) | 0.55 | 0.05 |
| CD55 | CD55 molecule, decay accelerating factor for complement (Cromer blood group) | 0.55 | 0.003 |
| TSLP | thymic stromal lymphopoietin | 0.55 | 0.013 |
| VKORC1 | vitamin K epoxide reductase complex, subunit 1 | 0.55 | 0.025 |
| ADM5 | adrenomedullin 5 (putative) | 0.55 | 0.002 |
| CORO6 | coronin 6 | 0.56 | 0.008 |
| TPM2 | tropomyosin 2 (beta) | 0.56 | 0.008 |
| TSSK3 | testis-specific serine kinase 3 | 0.56 | 0.016 |
| SPATA12 | spermatogenesis associated 12 | 0.56 | 0.044 |
| RNASET2 | ribonuclease T2 | 0.56 | 0.002 |
| LPCAT4 | lysophosphatidylcholine acyltransferase 4 | 0.56 | 0.036 |
| SGSM1 | small G protein signaling modulator 1 | 0.57 | 0.002 |
| MAPK8IP2 | mitogen-activated protein kinase 8 interacting protein 2 | 0.57 | 0.022 |
| CLDN14 | claudin 14 | 0.57 | 0.028 |
| WSB1 | WD repeat and SOCS box containing 1 | 0.57 | 0.017 |
| M1AP | meiosis 1 associated protein | 0.57 | 0.05 |
| SULT1C2P1 | sulfotransferase family, cytosolic, 1C, member 2 pseudogene 1 | 0.57 | 0.025 |
| PEX5L | peroxisomal biogenesis factor 5-like | 0.57 | 0.049 |
| LOC100128076 | protein tyrosine phosphatase pseudogene | 0.58 | 0.044 |
| C8orf31 | chromosome 8 open reading frame 31 | 0.58 | 0.030 |
| RAC2 | ras-related C3 botulinum toxin substrate 2 (rho family, small GTP binding protein Rac2) | 0.58 | 0.048 |
| CCDC88B | coiled-coil domain containing 88B | 0.58 | 0.040 |
| COX6B2 | cytochrome c oxidase subunit VIb polypeptide 2 (testis) | 0.58 | 0.019 |
| PPFIA4 | protein tyrosine phosphatase, receptor type, f polypeptide (PTPRF), interacting protein (liprin), alpha 4 | 0.58 | 0.008 |
| ZNF582-AS1 | ZNF582 antisense RNA 1 (head to head) | 0.58 | 0.032 |
| LINC00624 | long intergenic non-protein coding RNA 624 | 0.58 | 0.038 |
| ZNF83 | zinc finger protein 83 | 0.58 | 0.005 |
| LOC143666 | uncharacterized LOC143666 | 0.58 | 0.021 |
| ZFPM2 | zinc finger protein, FOG family member 2 | 0.58 | 0.033 |
| HOXA4 | homeobox A4 | 0.58 | 0.032 |
| BAIAP2L2 | BAI1-associated protein 2-like 2 | 0.58 | 0.004 |
| C20orf195 | chromosome 20 open reading frame 195 | 0.59 | 0.042 |
| LOC284454 | uncharacterized LOC284454 | 0.59 | 0.010 |
| NYAP2 | neuronal tyrosine-phosphorylated phosphoinositide-3-kinase adaptor 2 | 0.59 | 0.011 |
| P4HA1 | prolyl 4-hydroxylase, alpha polypeptide I | 0.59 | 0.016 |
| FTL | ferritin, light polypeptide | 0.59 | 0.011 |
| CDH17 | cadherin 17, LI cadherin (liver-intestine) | 0.59 | 0.015 |
| PDXDC2P | pyridoxal-dependent decarboxylase domain containing 2, pseudogene | 0.59 | 0.012 |
| KCNQ4 | potassium voltage-gated channel, KQT-like subfamily, member 4 | 0.59 | 0.005 |
| AGER | advanced glycosylation end product-specific receptor | 0.59 | 0.022 |
| PPM1E | protein phosphatase, Mg2+/Mn2+ dependent, 1E | 0.59 | 0.035 |
| LOX | lysyl oxidase | 0.59 | 0.033 |
| DNAAF1 | dynein, axonemal, assembly factor 1 | 0.59 | 0.009 |
| ITM2C | integral membrane protein 2C | 0.59 | 0.009 |
| - | ArfGAP with GTPase domain, ankyrin repeat and PH domain 8 | 0.59 | 0.024 |
| NTF4 | neurotrophin 4 | 0.60 | 0.009 |
| CPLX1 | complexin 1 | 0.60 | 0.044 |
| UNC5A | unc-5 homolog A (C. elegans) | 0.60 | 0.026 |
| MIOX | myo-inositol oxygenase | 0.60 | 0.003 |
| NCRNA00185 | non-protein coding RNA 185 | 0.60 | 0.027 |
| ATP2C2 | ATPase, Ca++ transporting, type 2C, member 2 | 0.60 | 0.027 |
| PTPRH | protein tyrosine phosphatase, receptor type, H | 0.60 | 0.013 |
| ROM1 | retinal outer segment membrane protein 1 | 0.60 | 0.0006 |
| GFAP | glial fibrillary acidic protein | 0.60 | 0.035 |
| RORC | RAR-related orphan receptor C | 0.60 | 0.044 |
| MIR614 | microRNA 614 | 0.60 | 0.042 |
| LOC442028 | uncharacterized LOC442028 | 0.60 | 0.0005 |
| LOC283683 | uncharacterized LOC283683 | 0.60 | 0.034 |
| MC1R | melanocortin 1 receptor (alpha melanocyte stimulating hormone receptor) | 0.60 | 0.020 |
| LINC00663 | long intergenic non-protein coding RNA 663 | 0.60 | 0.010 |
| DCST2 | DC-STAMP domain containing 2 | 0.61 | 0.016 |
| LINC00887 | uncharacterized LOC100131551 | 0.61 | 0.006 |
| MMP19 | matrix metallopeptidase 19 | 0.61 | 0.036 |
| EXD3 | exonuclease 3'-5' domain containing 3 | 0.61 | 0.021 |
| SLC2A1 | solute carrier family 2 (facilitated glucose transporter), member 1 | 0.61 | 0.012 |
| REC8 | REC8 homolog (yeast) | 0.61 | 0.044 |
| HLA-A | major histocompatibility complex, class I, A | 0.61 | 0.004 |
| TRABD2B | TraB domain containing 2B | 0.61 | 0.022 |
| FBXL16 | F-box and leucine-rich repeat protein 16 | 0.61 | 0.002 |
| WBSCR27 | Williams Beuren syndrome chromosome region 27 | 0.61 | 0.0007 |
| TCN2 | transcobalamin II | 0.61 | 0.005 |
| CD72 | CD72 molecule | 0.61 | 0.019 |
| KRT15 | keratin 15 | 0.61 | 0.011 |
| HCFC1R1 | host cell factor C1 regulator 1 (XPO1 dependent) | 0.61 | 0.044 |
| RASA4CP | RAS p21 protein activator 4C, pseudogene | 0.61 | 0.048 |
| SMPD3 | sphingomyelin phosphodiesterase 3, neutral membrane (neutral sphingomyelinase II) | 0.61 | 0.009 |
| VAMP1 | vesicle-associated membrane protein 1 (synaptobrevin 1) | 0.62 | 0.043 |
| PER1 | period circadian clock 1 | 0.62 | 0.040 |
| MFI2-AS1 | MFI2 antisense RNA 1 | 0.62 | 0.0007 |
| LOC115110 | uncharacterized LOC115110 | 0.62 | 0.012 |
| B3GAT2 | beta-1,3-glucuronyltransferase 2 (glucuronosyltransferase S) | 0.62 | 0.046 |
| KCNN4 | potassium intermediate/small conductance calcium-activated channel, subfamily N, member 4 | 0.62 | 0.034 |
| FAM86HP | family with sequence similarity 86, member A pseudogene | 0.62 | 0.047 |
| LOC100506472 | uncharacterized LOC100506472 | 0.62 | 0.045 |
| PRR15L | proline rich 15-like | 0.62 | 0.018 |
| PRKCG | protein kinase C, gamma | 0.62 | 0.05 |
| PHF1 | PHD finger protein 1 | 0.62 | 0.010 |
| ALS2CL | ALS2 C-terminal like | 0.62 | 0.013 |
| TCP10L | t-complex 10-like | 0.62 | 0.002 |
| CD96 | CD96 molecule | 0.63 | 0.046 |
| ARHGAP22 | Rho GTPase activating protein 22 | 0.63 | 0.025 |
| EGFLAM-AS4 | EGFLAM antisense RNA 4 | 0.63 | 0.043 |
| FLJ44511 | uncharacterized LOC441307 | 0.63 | 0.021 |
| PRRT2 | proline-rich transmembrane protein 2 | 0.63 | 0.044 |
| PAXIP1-AS1 | uncharacterized LOC202781 | 0.63 | 0.001 |
| MRPS6 | mitochondrial ribosomal protein S6 | 0.63 | 0.013 |
| PPM1J | protein phosphatase, Mg2+/Mn2+ dependent, 1J | 0.63 | 0.017 |
| CYLC2 | cylicin, basic protein of sperm head cytoskeleton 2 | 0.63 | 0.006 |
| SNORA61 | small nucleolar RNA, H/ACA box 61 | 0.63 | 0.015 |
| HES2 | hairy and enhancer of split 2 (Drosophila) | 0.63 | 0.038 |
| MAPK8IP3 | mitogen-activated protein kinase 8 interacting protein 3 | 0.63 | 0.046 |
| CLEC7A | C-type lectin domain family 7, member A | 0.63 | 0.016 |
| CCBL1 | cysteine conjugate-beta lyase, cytoplasmic | 0.63 | 0.027 |
| IRF8 | interferon regulatory factor 8 | 0.63 | 0.029 |
| PAQR6 | progestin and adipoQ receptor family member VI | 0.63 | <0.0001 |
| MGC16275 | uncharacterized protein MGC16275 | 0.63 | 0.018 |
| GJB3 | gap junction protein, beta 3, 31kDa | 0.64 | 0.017 |
| LENG8 | leukocyte receptor cluster (LRC) member 8 | 0.64 | 0.025 |
| EEPD1 | endonuclease/exonuclease/phosphatase family domain containing 1 | 0.64 | 0.021 |
| PI4KAP1 | phosphatidylinositol 4-kinase, catalytic, alpha pseudogene 1 | 0.64 | 0.023 |
| AMT | aminomethyltransferase | 0.64 | 0.019 |
| LOC100133445 | uncharacterized LOC100133445 | 0.64 | 0.047 |
| DBP | D site of albumin promoter (albumin D-box) binding protein | 0.64 | 0.022 |
| HERC2P7 | hect domain and RLD 2 pseudogene 7 | 0.64 | 0.022 |
| UPK1B | uroplakin 1B | 0.64 | 0.019 |
| SNHG18 | uncharacterized LOC100505806 | 0.64 | 0.042 |
| HMOX1 | heme oxygenase (decycling) 1 | 0.64 | 0.016 |
| SFTPB | surfactant protein B | 0.64 | 0.007 |
| LINC01289 | uncharacterized LOC286184 | 0.64 | 0.049 |
| EPOR | erythropoietin receptor | 0.64 | 0.040 |
| LOXL2 | lysyl oxidase-like 2 | 0.64 | 0.003 |
| BAIAP3 | BAI1-associated protein 3 | 0.64 | 0.016 |
| FAM86JP | family with sequence similarity 86, member A pseudogene | 0.64 | 0.045 |
| GLRX | glutaredoxin (thioltransferase) | 0.65 | 0.019 |
| LY6E | lymphocyte antigen 6 complex, locus E | 0.65 | 0.05 |
| MIR4458HG | uncharacterized LOC100505738 | 0.65 | 0.027 |
| INPP5D | inositol polyphosphate-5-phosphatase, 145kDa | 0.65 | 0.006 |
| LOC100133331 | uncharacterized LOC100133331 | 0.65 | 0.042 |
| NAB2 | NGFI-A binding protein 2 (EGR1 binding protein 2) | 0.65 | 0.010 |
| CALCR | calcitonin receptor | 0.65 | 0.035 |
| MST1 | macrophage stimulating 1 (hepatocyte growth factor-like) | 0.65 | 0.007 |
| C1orf54 | chromosome 1 open reading frame 54 | 0.65 | 0.041 |
| WNK4 | WNK lysine deficient protein kinase 4 | 0.65 | 0.044 |
| C3orf67 | chromosome 3 open reading frame 67 | 0.65 | 0.033 |
| FGFBP1 | fibroblast growth factor binding protein 1 | 0.65 | 0.021 |
| RHCE | Rh blood group, CcEe antigens | 0.65 | 0.035 |
| MTMR11 | myotubularin related protein 11 | 0.65 | 0.008 |
| MOG | myelin oligodendrocyte glycoprotein | 0.65 | 0.029 |
| CPNE7 | copine VII | 0.66 | 0.010 |
| FOXD2-AS1 | FOXD2 antisense RNA 1 (head to head) | 0.66 | 0.035 |
| PRR27 | chromosome 4 open reading frame 40 | 0.66 | 0.006 |
| TNFRSF6B | tumor necrosis factor receptor superfamily, member 6b, decoy | 0.66 | 0.049 |
| SNX33 | sorting nexin 33 | 0.66 | 0.020 |
| LOC729970 | hCG2028352-like | 0.66 | 0.044 |
| 37135 | septin 1 | 0.66 | 0.014 |
| FN3K | fructosamine 3 kinase | 0.66 | 0.014 |
| ARSF | arylsulfatase F | 0.66 | 0.026 |
| ZEB1-AS1 | ZEB1 antisense RNA 1 | 0.66 | 0.030 |
| PDZD7 | PDZ domain containing 7 | 0.66 | 0.043 |
| SUV420H2 | suppressor of variegation 4-20 homolog 2 (Drosophila) | 0.66 | 0.016 |
| MAMDC4 | MAM domain containing 4 | 0.66 | 0.001 |
| LTBP4 | latent transforming growth factor beta binding protein 4 | 0.66 | 0.019 |
| SYTL3 | synaptotagmin-like 3 | 0.67 | 0.006 |
